# Expression-driven genetic dependency reveals targets for precision oncology

**DOI:** 10.1093/gigascience/giag011

**Published:** 2026-01-29

**Authors:** Abdulkadir Elmas, Hillary M Layden, Jacob D Ellis, Luke N Bartlett, Xian Zhao, Reika Kawabata-Iwakawa, Zishan Wang, Hideru Obinata, Scott W Hiebert, Kuan-lin Huang

**Affiliations:** Department of Genetics and Genomic Sciences, Department of Artificial Intelligence and Human Health, Center for Transformative Disease Modeling, Tisch Cancer Institute, Icahn Genomics Institute, Icahn School of Medicine at Mount Sinai, 1 Gustave L. Levy Place, New York, NY 10029, USA; Department of Biochemistry, Vanderbilt University School of Medicine, 2209 Garland Avenue, Nashville, TN 37232, USA; Department of Biochemistry, Vanderbilt University School of Medicine, 2209 Garland Avenue, Nashville, TN 37232, USA; Department of Biochemistry, Vanderbilt University School of Medicine, 2209 Garland Avenue, Nashville, TN 37232, USA; Department of Biochemistry, Gunma University Graduate School of Medicine, 3-39-22 Showa-machi, Maebashi, Gunma 371-8511, Japan; Department of Pharmacy, Nanjing Drum Tower Hospital, Affiliated Hospital of Medical School, Nanjing University, 321 Zhongshan Road, Nanjing, Jiangsu 210008, China; Division of Integrated Oncology Research, Gunma University Initiative for Advanced Research, Gunma University, 3-39-22 Showa-machi, Maebashi, Gunma 371-8511, Japan; Department of Genetics and Genomic Sciences, Department of Artificial Intelligence and Human Health, Center for Transformative Disease Modeling, Tisch Cancer Institute, Icahn Genomics Institute, Icahn School of Medicine at Mount Sinai, 1 Gustave L. Levy Place, New York, NY 10029, USA; Education and Research Support Center, Gunma University Graduate School of Medicine, 3-39-22 Showa-machi, Maebashi, Gunma 371-8511, Japan; Department of Biochemistry, Vanderbilt University School of Medicine, 2209 Garland Avenue, Nashville, TN 37232, USA; Vanderbilt-Ingram Cancer Center, Vanderbilt University School of Medicine, 2220 Pierce Avenue, Nashville, TN 37027, USA; Department of Genetics and Genomic Sciences, Department of Artificial Intelligence and Human Health, Center for Transformative Disease Modeling, Tisch Cancer Institute, Icahn Genomics Institute, Icahn School of Medicine at Mount Sinai, 1 Gustave L. Levy Place, New York, NY 10029, USA

**Keywords:** precision oncology, expression-driven dependency, cancer vulnerability, BEACON, Bayesian statistics, proteomics, transcriptomics, cancer cell lines, drug targets, multi-omics

## Abstract

**Background:**

Cancer cells are heterogeneous, each harboring distinct molecular aberrations and being dependent on different genes for their survival and proliferation. While targeted therapies based on driver DNA mutations have shown success, many tumors lack druggable mutations, limiting treatment options. We hypothesize that new precision oncology targets may be identified through “expression-driven dependency,” where cancer cells with high expression of specific genes are more vulnerable to the knockout of those same genes.

**Results:**

We developed BEACON, a Bayesian approach to identify expression-driven dependency targets by analyzing global transcriptomic and proteomic profiles alongside genetic dependency data from cancer cell lines across 17 tissue lineages. BEACON successfully identified known druggable genes, including *BCL2, ERBB2, EGFR, ESR1*, and *MYC*, while revealing novel targets confirmed by both mRNA- and protein-expression–driven dependency. The identified genes showed a 3.8-fold enrichment for approved drug targets and a 7- to 10-fold enrichment for druggable oncology targets. Experimental validation demonstrated that depletion of *GRHL2, TP63*, and *PAX5* effectively reduced tumor cell growth and survival in their dependent cells.

**Conclusions:**

Our approach provides a systematic method to identify precision oncology targets based on expression-driven dependency patterns. By integrating multi-omics data with genetic dependency screens, we have created a comprehensive catalog of potential therapeutic targets that may expand treatment options for cancer patients lacking druggable mutations. This resource offers new opportunities for precision oncology target discovery beyond mutation-based approaches.

## Introduction

Precision oncology requires accurate identification of molecular aberrations in cancer cells that can serve as biomarkers and therapeutic targets. While some tumors harbor genomic mutations predictive of cancer vulnerability, a large fraction of cancer cells lack such actionable mutations [1–3]. Large-scale genetic dependency screens, including the Cancer Cell Line Encyclopedia (CCLE) [4], Cancer Dependency Map (DepMap) [5], and CancerGD [6], have revealed that cancer cells show different vulnerability upon genetic knockdown or knockout. Across diverse types of molecular alterations—including mutations, copy number alterations, and expression—gene expression biomarkers have been identified as the top biomarkers of genetic dependency, e.g., in 82% of the 501 DepMap cell lines in a genome-scale RNAi screen [5]. We thus reasoned that precision oncology targets might be identified through “expression-driven dependency,” whereby cancer cells with high expression of the targeted genes are more vulnerable to genetic depletion or therapeutic inhibition.

Multiple studies have used genetic and functional screening data to identify cancer vulnerabilities present in a subset of cancer cells, including aneuploid cancer cells [7, 8], pediatric tumor cells [9], and multiple myeloma cells [10]. Notable targets identified include the *WRN* helicase that is essential in cancers with microsatellite instability [11, 12], PKMYT1 kinase in *CCNE1*-amplified tumors, and *BCAR1* in *KRAS* mutant pancreatic cancer, where the suppression of *BCAR1* and *TUBB3* sensitizes cancer cells to *ERK* inhibition by reducing MYC protein levels [13]. Bondeson et al. [14] identified phosphate dysregulation as a therapeutic vulnerability in ovarian cancer through genome-scale CRISPR-Cas9 screens, highlighting the XPR1–KIDINS220 protein complex as crucial for cancer cell survival. Another study [8] identified the ubiquitin ligase complex UBA6/BIRC6/KCMF1/UBR4 as crucial for the survival of aneuploid epithelial tumors. These studies highlight the potential of developing a systematic approach to identify drug targets by linking subsets of cancer cells to genetic dependency based on their aberrant expression.

Expression analyses focusing on only the transcriptome assume that high gene mRNA expression translates into high protein abundance. However, gene expressions show only moderate correlations with protein expression in cancer cell lines and primary tumors [15–20], and protein-level analyses may identify new targets [3, 21–23]. Notably, global proteomic profiles of 375 cell lines in the CCLE/DepMap were recently generated by global mass spectrometry (MS), quantifying a total of 12,399 proteins using multiplexing quantification methods [24]. The combination of these datasets provides unprecedented opportunities to identify new protein biomarkers and therapeutic targets across cancer types.

**Figure 1:**
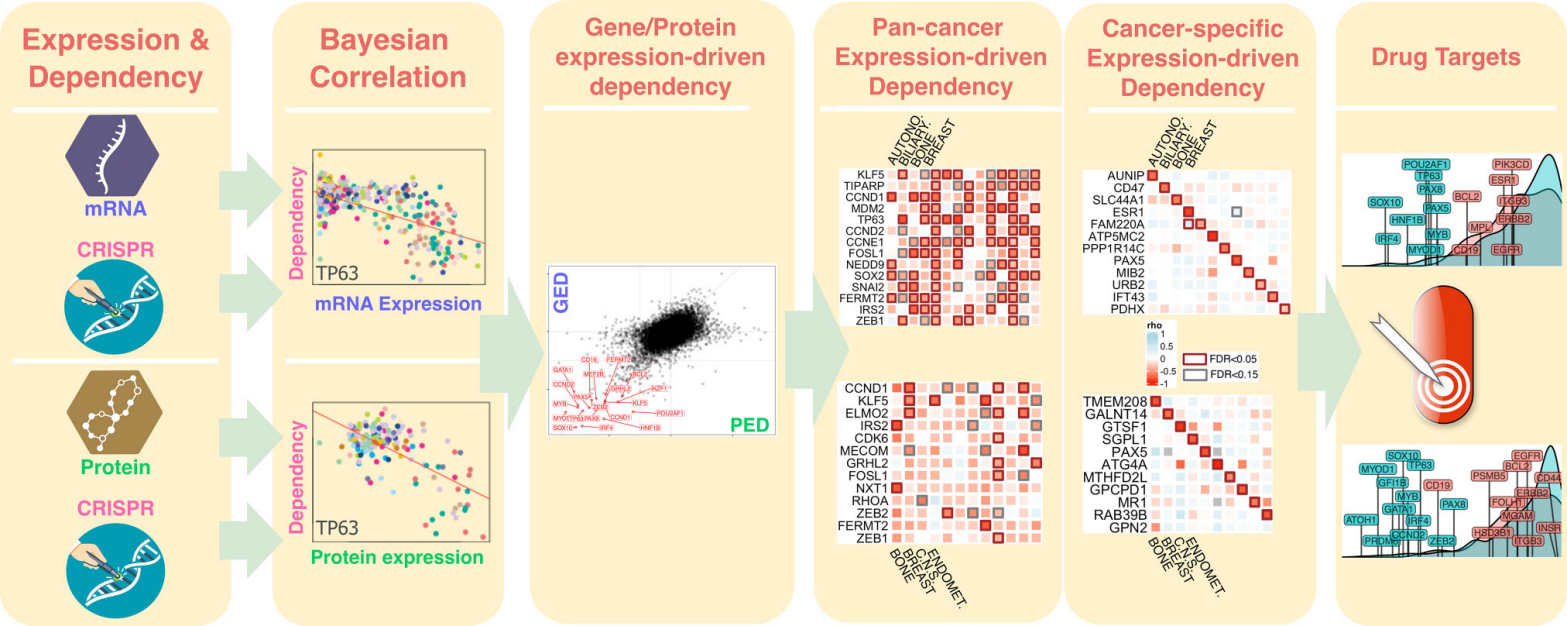
Study overview. (A) The integration of global proteomic and transcriptomic profiles from 375 cancer cell lines across 22 tissue types in the CCLE, with cancer cell dependency scores derived from CRISPR knockout screens (Achilles). (B) BEACON identifies expression-driven dependency (ED) by using a Bayesian estimation of the correlation coefficient between gene/protein expression and cancer cell dependency data across the cell lines for a representative gene (e.g., *TP63*). (C) Comparison of gene/protein EDs revealed potential markers showing consistency at different molecular levels or arising post-transcriptionally. (D) Heatmaps showing pan-cancer expression-driven dependencies, GED (above) and PED (below), revealing dependencies that are common across multiple cancer types. (E) Heatmaps illustrating cancer-specific expression-driven dependencies, GED (above) and PED (below), identifying dependencies unique to specific cancer types. (F) Identification of new potentially actionable targets that are strongly associated with druggable gene lists catalogued in DrugBank [28], highlighting their therapeutic potential.

Herein, we integrated global proteomic and transcriptomic profiles of 855 cancer cell lines across 17 tissue types from DepMap/CCLE [24, 25], and the corresponding cancer cell dependency scores (Achilles) based on the CRISPR knockout screens [25–27]. By developing a new Bayesian correlation approach, BEACON, we identified the expression-driven cancer cell dependencies (ED) for each tissue type at different molecular layers, and revealed new potentially actionable targets that are strongly associated with druggable gene lists [28] (Fig. 1). Our analyses identified the known drug targets *SOX10* and *ESR1* demonstrating strong gene/protein ED linked to their specified cancer type and revealed new potential candidate targets for each cancer type. Experimental validation supported the actionability of the new candidate targets *TP63, GRHL2*, and *PAX5*, exposing a vulnerability in their dependent cancer cells.

## Results

To identify genes showing expression-driven dependency, we first integrated RNA-seq data, global MS proteomics data, and the cell dependency data corresponding to the same cell lines in the DepMap project (see the “Methods” section). We restricted our analyses to lineages where at least 7 cell lines with cancer cell line dependency and corresponding mRNA/protein expression data were available to ensure statistical robustness (Fig. S1A). Overall, 855 cell lines across the 17 lineages shared cancer cell dependency scores and corresponding mRNA and protein expressions (*N* = 854 for mRNA, *N* = 290 for protein, Fig. S1B, Table S1). Based on this limited sample size per cell lineage (Fig. S1C), we noticed that the basic correlation techniques may lead to spurious correlations, particularly for protein expression (Figs S1B and S2). Thus, we developed a Bayesian approach, BEACON (Bayesian EvAluation of expression Correlation-driveN dependency), to model expression levels and dependency scores as the bivariate Gaussians and used Markov Chain Monte Carlo (MCMC) sampling to test the null hypothesis that these 2 are uncorrelated for each given gene (see the “Methods” section). BEACON offers the unique advantage of utilizing prior distributions that are less sensitive to outliers, which is particularly beneficial in lineages where the number of available cell lines is small and thus more vulnerable to the influence of outliers. We benchmarked BEACON’s Bayesian correlation against Pearson correlation, which was used in project DRIVE [29], and against both Pearson and Spearman correlation measures, which were employed in BACON [30]. Simulations were performed on expression and dependency datasets across a range of correlation levels (from −1 to 1, with 0.25 intervals) and sample size (number of cell lines, 5, 7, 10, 20, 30, 60, 100), with different fractions (0.1, 0.3, 0.5, 0.8, 1) of samples corrupted by noise to enable direct comparison of methodological performance (Fig. S2). Based on these simulations, we observed that the Bayesian method is better than Pearson correlation for estimating moderate true correlation (|rho| < 0.75) in small sample size, and preferable in noisy data (noise level ≥ 0.5, i.e., 50% or more of the samples are corrupted by noise to become outliers), regardless of sample size or true correlation level.

To further validate BEACON on real data, we systematically benchmarked its performance against Pearson and Spearman correlations using a curated set of 2,993 druggable genes from drug–gene interaction database (DGIdb) as the reference standard. For each cancer lineage, we calculated the area under the precision–recall curve (AUPRC) for identifying DGIdb genes based on expression–dependency correlation scores. On average across all lineages, BEACON achieved an AUPRC improvement of ∼27–29% over Pearson and ∼27% over Spearman (Fig. S3), based on both mRNA and protein expression data. Specifically, BEACON was the top-performing method in 19 of 24 lineages for GED (mRNA) and in 11 of 17 lineages for PED (protein) (Fig. S3). The advantage was particularly pronounced in lineages with smaller sample sizes (e.g., prostate, pleura, and thyroid in GED; central nervous system, endometrium, and stomach in PED, etc.), where AUPRC gains reached more than 2-fold over Pearson/Spearman.

A complementary benchmarking against the 57 prioritized genes identified by Project DRIVE’s expression-dependency model (Pearson-based) showed an even greater performance advantage for BEACON. Across all lineages, BEACON achieved average AUPRC gains of ∼104–582% over Pearson and ∼150–690% over Spearman (Fig. S3). BEACON was also the top-performing method in 19 of 24 lineages for GED (mRNA) and in 10 of 17 lineages for PED (protein). These results demonstrate that BEACON improves over simpler correlation measures and enhances the recovery of biologically validated dependencies compared to established benchmarks.

### Cancer vulnerability targets showing gene expression-driven dependency

We first applied BEACON to reveal cancer vulnerabilities that show gene expression-driven dependencies (GEDs) at the mRNA level. We first analyzed the pan-lineage GED by using mRNA levels and the corresponding dependency scores from 854 cell lines with available data across 17 lineages and identified 240 genes showing significant association (correlation coefficient, rho < −0.25, FDR < 0.05). The notable genes with strong pan-lineage associations (false discovery rate, FDR < 1e-32) include *SOX10* (correlation coefficient, rho = −0.83), *IRF4* (rho = −0.82), *HNF1B* (rho = −0.76), *MYOD1* (rho = −0.70), and *TP63* (rho = 0.69) (Table S2).

**Figure 2:**
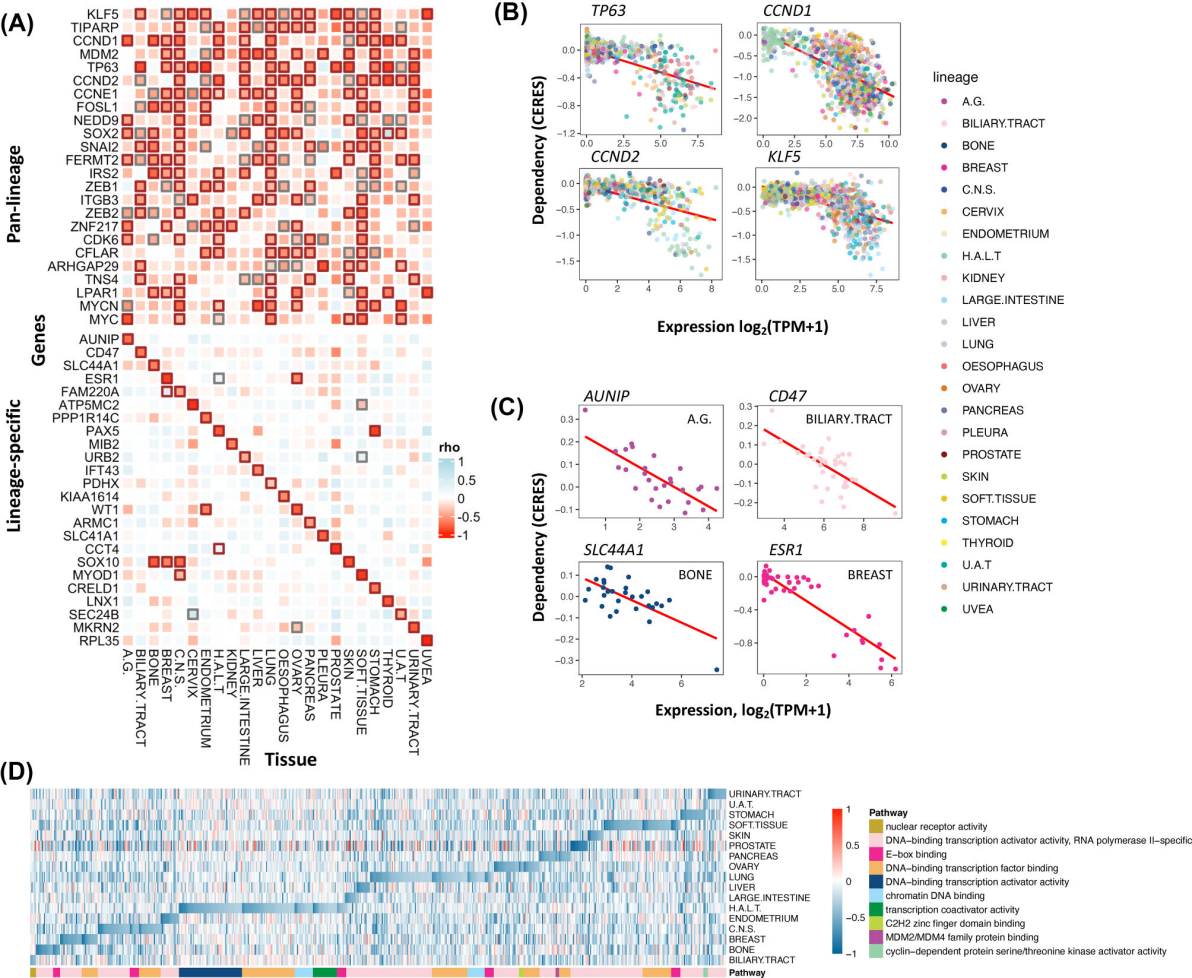
Gene expression-driven dependency (GED). (A) Heatmap illustrating pan-lineage and lineage-specific GEDs across various cancer types. Each square represents the correlation (rho) between gene expression and dependency (CERES scores) in the respective tissue types. Significant dependencies are highlighted with bold outlines (FDR < 0.05 in black, FDR < 0.15 in gray). (B) Scatter plots showing examples of gene expression vs. dependency correlations for selected genes (*TP63, CCND1, CCND2, KLF5*) with significant pan-lineage dependencies. Data points (cell lines) are colored by tissue type. (C) Scatter plots demonstrating lineage-specific dependencies for selected genes (*AUNIP, CD47, SLC44A1, ESR1*). Data points are colored by tissue type, highlighting lineage-specific associations. (D) Pathway enrichment analysis of lineage-specific GEDs, visualized as a heatmap. Each cell indicates the ED score of a particular pathway gene (column) in a specific tissue type (row), with genes grouped (colored) by functional pathways.

Having found many strong GEDs across cancer cells from different tissue types, we then applied BEACON to identify tissue-specific GEDs within each lineage (see the “Methods” section). As expected, several significant pan-lineage GED targets also showed substantial tissue-level GED in multiple lineages, including *TP63, CCND1, CCND2*, and *KLF5* (rho ≤ −0.61, FDR < 1e-32) (Fig. 2A, B). *TP63* showed significant (rho < −0.25, FDR < 0.05) GED across 14 out of 24 lineages of the cancer cell lines. *TP63* is a member of the p53-family transcription factors (TFs) that regulates developmental processes in several organs and tissues, as well as tumorigenesis and tumor progression [31]. Another TF, *KLF5*, also showed significant (rho < −0.25, FDR < 0.05) GED frequently across half of the cell lineages (12/24). This could be explained by its role in the development and progression of various types of cancer, as its expression is essential for cell cycle regulation, apoptosis, migration, and differentiation, impacting a wide array of target genes such as cyclin D1, cyclin B, PDGFα, and FGF-BP [32].

Since multiple lineages were dependent on the expression of some TFs such as *KLF5* and *TP63*, targeting these genes may lead to unintended consequences across tissue types. To minimize potential off-target effects, we further identified the GED targets showing only lineage-specific expression-driven dependency, i.e., exhibiting low correlation (more negative rho) within a given lineage’s cell lines and relatively smaller (near-zero) correlation in other lineages (Methods, Fig. 2A). Among such targets, we found *MYOD1* for soft tissue, *PAX5* for hematopoietic and lymphoid tissue, *SOX10* for skin, and *ESR1* for breast (rho ≤ −0.81, FDR < 1e-32) (Table S2, Fig. 2A, C), the latter of which is an already well-targeted gene through hormonal therapy using selective estrogen receptor modulators (SERMs), such as tamoxifen, and aromatase inhibitors. We next investigated whether the candidate targets showing GED were enriched in distinct molecular pathways. Enrichment analyses using gene ontology (GO) [33] for each lineage GED revealed 34 unique pathways enriched across lineages (Fig. 2D). Although different pathways showed different levels of ED, the 2 GO terms, (i) “DNA-binding transcription activator activity, RNA polymerase II-specific” (GO:0001228), and (ii) “DNA-binding transcription factor binding” (GO:0140297) were the most frequently enriched across the lineages (14 and 7 out of 18 lineages, respectively) (Table S7).

To explore the potential clinical actionability of the identified GEDs, we integrated DGIdb [34], and identified 81 druggable factors out of 240 pan-lineage GEDs (Fig. S4A, Table S3). By analyzing dependencies at each tissue, we identified 927 druggable targets, in total, showing significant (rho < −0.25, FDR < 0.05) lineage-specific GEDs, including 132 targets for hematopoietic and lymphoid tissue, 97 for lung, 77 for soft tissue, 58 for central nervous system, 53 for ovary, 51 for stomach, 41 for autonomic ganglia, and 40 for breast (Fig. S4B).

The most strongly associated (rho ≤ −0.78, FDR < 1e-32) tissue-specific GED targets include *MYOD1* in soft tissue, *ESR1* in breast, *WT1* in ovary, and *SOX10* in skin (Table S3). The skin-specific ED observed for *SOX10* was also concordant with a recent study [35], where the mRNA expression of *SOX10* was found to be associated with *SOX10* hypomethylation and sensitivity to *SOX10* knockdown in melanoma cell lines, while other tissue cell lines showed limited *SOX10* expression and limited dependency to *SOX10* for survival. The strong *ESR1*-driven dependency in breast cancer cell lines supports the established use of SERMs and aromatase inhibitors in ER(+) breast cancers [36]. Several ED genes already have established targeted therapies, and additional gene targets showing strong lineage-specific expression-driven dependencies may also have therapeutic potential.

Based on a set of the most significant GED targets found within lineages (rho < −0.75, FDR < 1e-10), clustering analyses (see the “Methods” section) showed that cancer cells of the pancreatic, large intestine, and biliary tract cancer cells share the most similar expression-driven dependency profiles, with kidney-endometrium and ovary-urinary tract tissue pairs also showing comparable clustering patterns (Fig. S4C). We also conducted a clustering analysis to identify GED-nominated drug targets showing similar tissue-specificities across tissue lineages. For example, the breast-specific *ESR1* TF is clustered with *IRX5* and *GATA3* (Fig. S4D). These TFs showed the strongest GED levels in breast tissue cell lines (rho < −0.5, FDR < 3e-4), where *GATA3* and IRX5 exhibited breast-specific GEDs comparable to *ESR1. GATA3* is a master regulator of luminal breast cancer identity and ER+ differentiation, while *IRX5* co-expressed with luminal transcriptional regulators and suppresses migratory phenotypes in breast cancer cells. Together, this core trio of luminal transcriptional regulators represents a dependency signature that may define targetable vulnerabilities in ER+ breast cancer subtypes. These results identified cross-tissue cancer cells that may share similar targets.

### Cancer vulnerability targets showing protein expression-driven dependency

**Figure 3:**
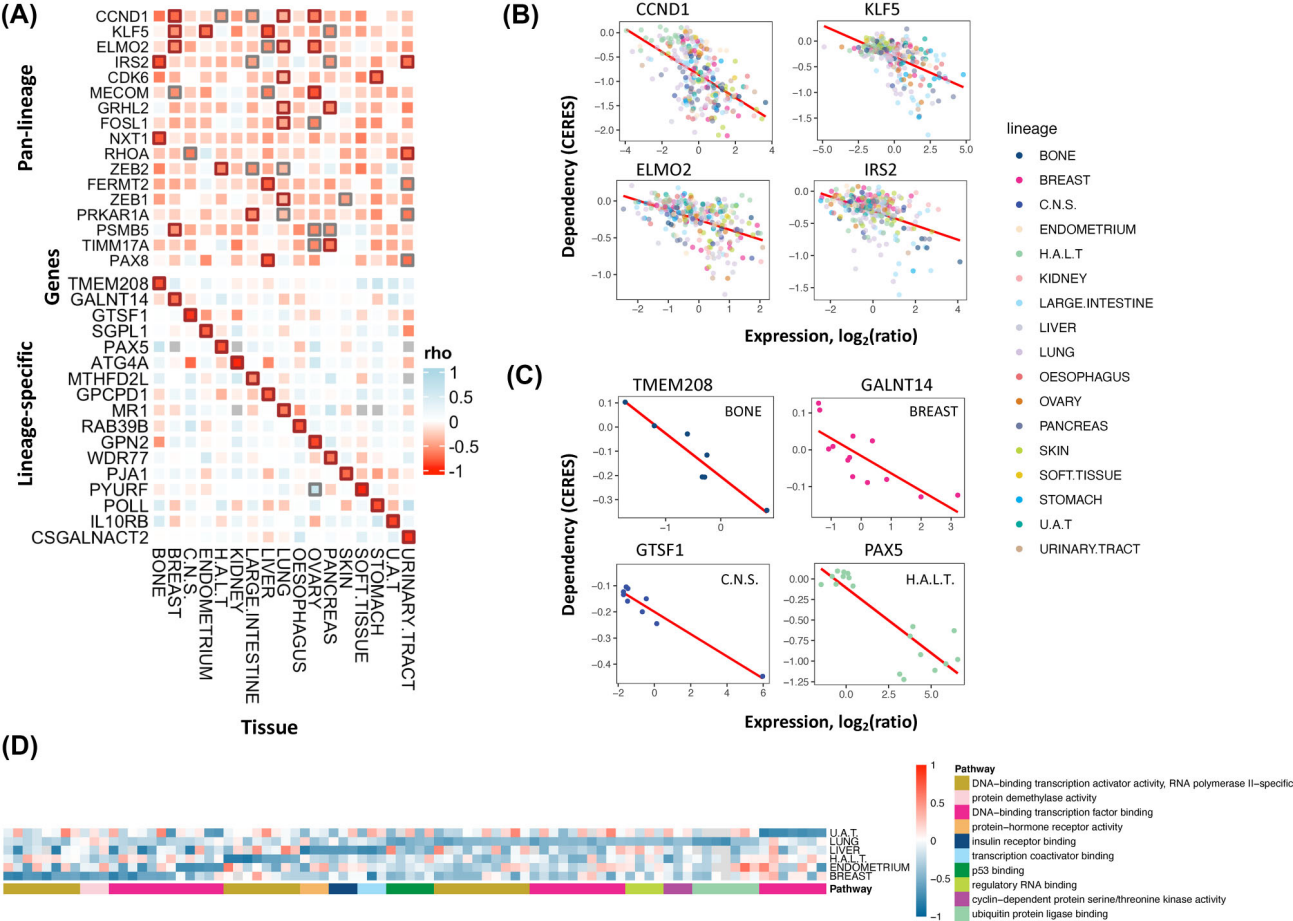
Protein expression-driven dependency (PED). (A) Heatmap illustrating pan-lineage and lineage-specific PEDs across various cancer types. Each square represents the correlation (rho) between protein expression and dependency (CERES scores) in the respective tissue types. Significant dependencies are highlighted with bold outlines (FDR < 0.05 in black, FDR < 0.15 in gray). (B) Scatter plots showing examples of protein expression vs. dependency correlations for selected genes (CCND1, KLF5, ELMO2, and IRS2) with significant pan-lineage dependencies. Data points (cell lines) are colored by tissue type. (C) Scatter plots demonstrating lineage-specific dependencies for selected genes (TMEM208, GALNT14, GTSF1, and PAX5). Data points are colored by tissue type, highlighting lineage-specific associations. (D) Pathway enrichment analysis of lineage-specific PEDs, visualized as a heatmap. Each cell indicates the ED score of a particular pathway gene (column) in a specific tissue type (row), with genes grouped (colored) by functional pathways.

Given that gene mRNA expressions show only moderate correlations with protein abundance in cancer [15–20], we next sought to expand our analyses to identify targets showing protein expression-driven dependency (PED). We applied BEACON to dependency data and protein expression levels in the subset of 290 cell lines with both types of data (see the “Methods” section). BEACON identified 220 proteins showing significant (rho < −0.25, FDR < 0.05) pan-lineage PED. Among the proteins showing pan-lineage PED, just over half (*N* = 123) of the targets also showed significant (rho < –0.25, FDR < 0.05) pan-lineage GED, suggesting general concordance between mRNA and protein while implicating the importance of considering protein expression. ZEB2 was the most strongly associated PED (rho = −0.64), followed by PAX8, GRHL2, CCND1, KLF5, FERMT2, and CDK6 (rho ≤ −0.52), all of which also showed significant GED (Table S4, Fig. 3A, B). The other 97 PED targets that do not show significant mRNA-level GED included ELMO2, PRDM6, RUNX1, VGLL1, CBFB, FGFR3, and NFE2L2 (rho ≤ −0.39) (Table S4). We identified 75 druggable proteins that show significant (rho < −0.25, FDR < 0.05) pan-lineage expression-driven dependency, including SOX10, MYB, GATA1, MYOD1, CCND1, CDK6, CCND2, PAX5, and HNF4A (rho ≤ −0.52) (Table S5).

At the individual tissue level, many of these pan-lineage PED targets also showed high ED within multiple lineages (Fig. 3A). Targets showing PED exclusive for each lineage (rho ≤ −0.83) included PYURF in soft tissue, GTSF1 in central nervous system, PAX5 in hematopoietic and lymphoid tissue, ATG4A in kidney, and TMEM208 in bone (Fig. 3A, C, Table S6). To examine potential actionability of the identified PED proteins, we integrated DGIdb and identified 152 druggable significant (rho < −0.25, FDR < 0.05) lineage-specific PEDs for all lineages; within these, we found a set of very strong lineage-specific targets (rho ≤ −0.77, FDR < 2e-4), including PAX5 in hematopoietic and lymphoid tissue, JMJD6 in central nervous system, IL10RB in upper aerodigestive tract, SERPIND1 in ovary, TSPO in kidney, and SGPL1 in endometrium (Fig. S4E, Table S6). Enrichment analyses with the PED targets yielded 15 pathways enriched in a more lineage-specific pattern than GED results (Fig. 3D, Table S7). DNA-binding transcription activator activity (GO:0140297 and GO:0001228) terms were similarly significantly enriched, showing consistency with the GED results (4 and 3 out of 7 lineages, respectively).

### Concordance between gene and protein expression-driven dependency

**Figure 4:**
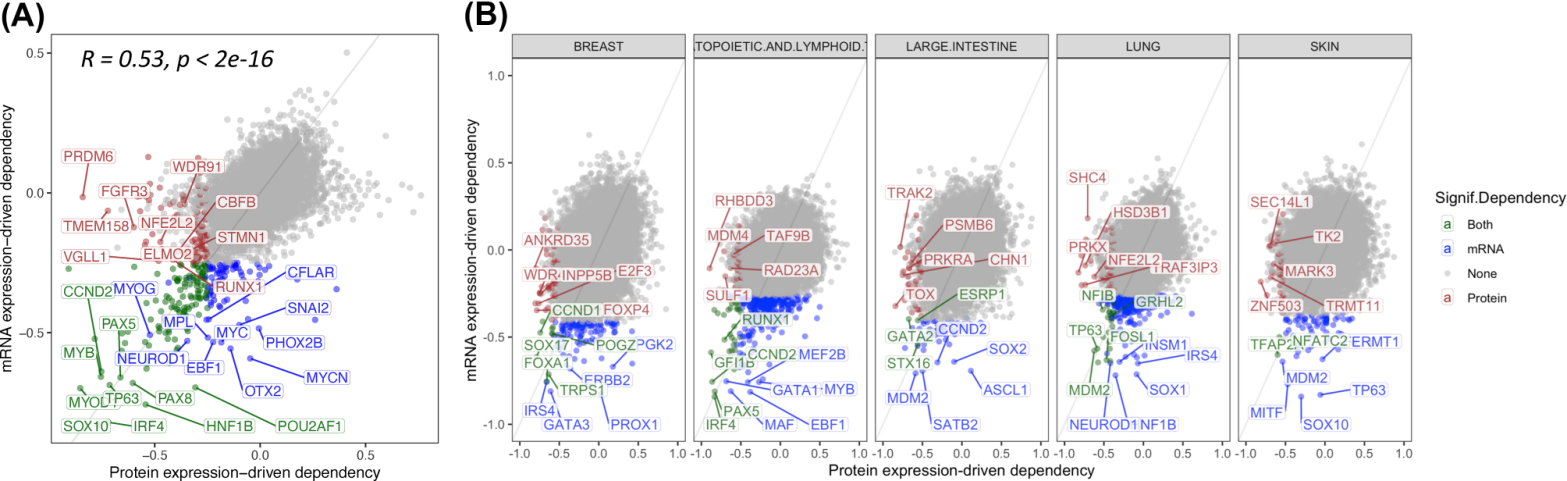
mRNA vs. PED. (A) Scatter plot illustrating the correlation between pan-lineage GEDs and PEDs across genes. Genes with consistent significant pan-lineage dependencies at both mRNA and protein levels are labeled, including top targets *SOX10, TP63, IRF4*, and *MYOD1*. Additional significant pan-lineage GEDs without corresponding PEDs (e.g., *MYCN, FOX2B, SNAI2*) and PEDs without corresponding GEDs (e.g., *PRDM6, TMEM158, FGFR3*) are also indicated. (B) Scatter plots showing the correlation between tissue-level GEDs and PEDs within specific lineages.

Protein expression evidence can validate molecular targets observed at the mRNA level. We thus analyze the concordant and unique gene targets based on their GED and PED correlations. We found 123 genes showing consistently significant pan-lineage expression-driven dependency in both mRNA and protein levels, most notably *SOX10* (rho_RNA_ = −0.83, rho_protein_ = −0.77), *TP63* (rho_RNA_ = −0.69, rho_protein_ = −0.71), *IRF4* (rho_RNA_ = −0.82, rho_protein_ = −0.72), and *MYOD1* (rho_RNA_ = −0.70, rho_protein_ = −0.85) (Fig. 4A, Table S4). The confirmation of both GED and PED demonstrates the robustness of these targets.

Meanwhile, given the moderate correlation between mRNA and protein, PED may also reveal protein aberrations that arise post-transcriptionally. We found 83 genes showing significant (rho < −0.25, FDR < 0.05) pan-lineage GED without a significant PED that may be less robust as potential therapeutic targets, including *MYCN* (rho_RNA_ = −0.59, rho_protein_ = −0.05), *FOX2B* (rho_RNA_ = −0.48, rho_protein_ = −0.006), and *SNAI2* (rho_RNA_ = −0.47, rho_protein_ = −0.01) (Fig. 4A, Table S8). On the other hand, we also found 97 proteins showing significant (rho < −0.25, FDR < 0.05) pan-lineage PED without a significant GED. Some notable targets include *PRDM6* (rho_RNA_ = −0.02, rho_protein_ = −0.85), TMEM158 (rho_RNA_ = −0.06, rho_protein_ = −0.72), *FGFR3* (rho_RNA_ = −0.12, rho_protein_ = −0.6), and WDR91 (rho_RNA_ = −0.04, rho_protein_ = −0.36) (Fig. 4A, Table S4).

We next analyzed the consistency between tissue-level GEDs and PEDs for each lineage (Fig. 4B, Table S9). In total, we found 109 genes showing significant GED and PED (rho < −0.25, FDR < 0.05) within a lineage, which may present as some of the strongest targets identified through BEACON. *FDFT1* showed significant GED and PED (rho < −0.53, FDR < 0.018) in the endometrium and urinary tract lineages. *SOX2* gene showed significant GED and PED (rho < −0.42, FDR < 0.028) in the lung and esophagus lineages. Other top lineage-specific targets showing concordance between GED and PEDs include *PAX5, IRF4, and CCND2/3* in hematopoietic and lymphoid tissue, *FOXA1/TRPS1* in breast, *MECOM/SERPIND1* in ovary, *MDM2/TP63* in lung, and *LIN28B/IRS2* in bone (rho < −0.56, FDR < 0.0015).

### Leveraging expression-driven dependency to enrich for drug targets

**Figure 5:**
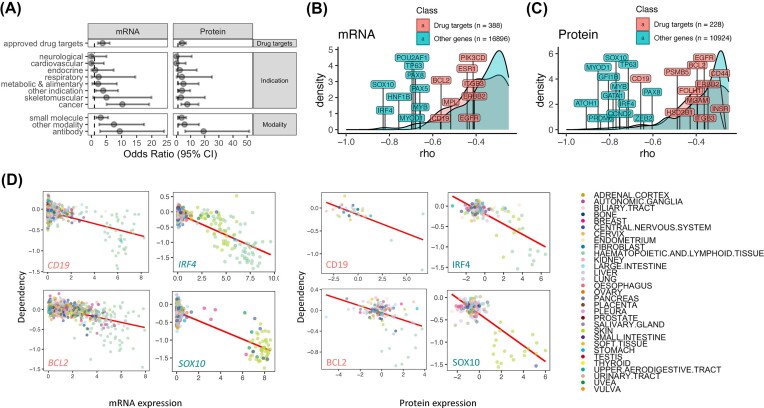
Leveraging expression-driven dependency to enrich for drug targets. (A) Enrichment (Fisher’s exact test) results demonstrating the enrichment of identified GEDs and PEDs in druggable gene lists curated by DrugBank, including all approved drug targets, drug targets by indication, and by drug modality. (B–C) The density plots of ED scores from drug targets (DrugBank approved targets) vs. other genes, highlighting the top significant targets identified at (B) mRNA and (C) protein levels. (D) Scatter plots of expression vs. dependency correlations for top drug targets and other genes, showing significant pan-lineage ED at both mRNA and protein levels (e.g., CD19, BCL2, *IRF4, SOX10*). Data points (cell lines) are colored by tissue type.

Identification of drug targets is a major goal of genomic studies, yet even by using 141,456 human DNA-Seq data in gnomAD without phenotype association, known drug targets only showed a minor difference in constraints for loss-of-function variants compared to other genes [28]. To test whether expression-driven dependency derived by BEACON may represent an effective strategy to identify drug targets, we ascertained whether the identified genes showing GED/PED are enriched for druggable targets from DrugBank and curated by Minikel et al. [28]. We used Fisher’s exact test to evaluate the association between the druggable gene lists and the pan-lineage GEDs/PEDs we identified (see the “Methods” section). The majority (8 out of 15) of druggable gene lists from DrugBank were significantly enriched (Fisher’s exact test, odds ratio > 2, FDR < 0.05) with expression-driven dependency observed at both mRNA and protein levels (Fig. 5A, Table S10). Genes targeted by *Antibody* were the gene set most enriched with GEDs and PEDs (OR_RNA_ = 9.4, OR_protein_ = 19.2), where the higher enrichment in PEDs aligns with the mechanism of action of the antibody directly binding to proteins. These GED/PED genes include Antibody targets (5 out of 36) showing significant levels of both GED/PED (rho < −0.25, FDR < 0.05) such as *CD19* (rho_RNA_ = −0.56, rho_protein_ = −0.66), *EGFR* (rho_RNA_ = −0.43, rho_protein_ = −0.36), *ITGB3* (rho_RNA_ = −0.41, rho_protein_ = −0.36), *ERBB2* (rho_RNA_ = −0.41, rho_protein_ = −0.35), and *PDGFRA* (rho_RNA_ = −0.37, rho_protein_ = −0.26) (Fig. 5B, C, Table S11). These targets also belong to DrugBank’s *Approved drug targets* (OR_RNA_ = 3.7, OR_protein_ = 3.9) and *Oncology* (*Cancer*) (OR_RNA_ = 10.2, OR_protein_ = 7.6) gene lists, both of which were also significantly enriched with GEDs and PEDs. The high fold enrichment for druggable genes in the *Oncology* gene set aligns with our analyses using DepMap cancer cell lines. Well-established targets within *Approved drug targets* and *Oncology* that show strong GED and PED include *BCL2* (for both RNA and protein level EDs, rho < −0.4), *PIK3CD* (rho < −0.34), and *PDGFRB* (rho < −0.3), suggesting that BEACON reliably captures established therapeutic dependencies and provides an effective framework for validating known oncogene addictions. Additionally, among the DrugBank *Oncology* gene set, *PSMB5* (targeted by proteasome inhibitors bortezomib and carfilzomib for hematologic malignancies) and *RXRA* (targeted by bexarotene, an RXR agonist used in the treatment of cutaneous T-cell lymphoma [CTCL]), showing significant levels of GED/PED, were among the Oncology gene list, reinforcing the robustness of our approach in identifying clinically relevant targets.

Notably, in addition to enrichment for known Oncology druggable genes, BEACON-identified GED/PEDs also showed suggestive enrichment for multiple other indication categories, including DrugBank gene sets for skeletomuscular (OR = 4.9, *P* = 0.098 for GEDs; OR = 3.1, *P* = 0.36 for PEDs) and metabolic/alimentary (OR = 2.2, *P* = 0.32 for GEDs; OR = 4.9, *P* = 0.045 for PEDs) diseases. The DrugBank Other Indications category with more targets and statistical power showed significant enrichment for both GEDs (OR = 3.9, FDR = 0.011) and PEDs (OR = 3.9, FDR = 0.02), suggesting there may be a broader utility of these cell-specific targets beyond oncology.

We next characterized whether GED/PEDs identified by BEACON may be more sensitive to identifying genes with specific mode of inheritance or with additional genetic effect properties [28]. GED/PED genes were both enriched for autosomal dominant genes and haploinsufficient genes as determined by ClinGen, but showed no association with autosomal recessive genes (Fig. S5), suggesting these candidates may capture disease genes that are more sensitive to dosage effects. Moreover, GED/PED targets were underrepresented among the common essential genes (684 “Essential In Culture” genes based on 17 genome-wide CRISPR screens [37]), suggesting that BEACON identifies cell-specific vulnerabilities rather than dependencies universally required for cell viability (e.g., housekeeping genes) that could lead to off-target effects. Overall, we identified 36 genes in 10 DrugBank/genetic effect lists that showed significant (rho < −0.25, FDR < 0.05) pan-lineage expression-driven dependency in both mRNA and protein levels (Table S12).

Additional GED/PED targets identified by BEACON that are not currently druggable targets (DrugBank approved) include *SOX10* (rho_RNA_ = −0.83, rho_protein_ = −0.77, also belong to *ClinGen Haploinsufficient* and *Autosomal Dominant* gene sets) and *TP63* (rho_RNA_ = −0.69, rho_protein_ = −0.71, *ClinGen Haploinsufficient*) and the *Autosomal Dominant* genes *GRHL2* (rho_RNA_ = −0.6, rho_protein_ = −0.53) and *HNF4A* (rho_RNA_ = −0.5, rho_protein_ = −0.63) (Fig. 5B, C, Table S11). For genes not in these DrugBank/gene-effect gene lists [28], BEACON identified 87 targets that showed significant ED (rho < −0.25, FDR < 0.05) at both mRNA and protein levels that may represent potential therapeutic targets for further experimental and clinical development, including *IRF4* (for both RNA and protein levels, rho < −0.72), *MYB* (rho < −0.66), *GATA1* (rho < −0.52), *CCND1* (rho < −0.54), *KLF5* (rho < −0.53), *FERMT2* (rho < −0.53), *MYOD1* (rho < −0.7), *PAX5* (rho < −0.66), and *CCND2* (rho < −0.64) (Table S13). For example, *IRF4* knockdown is lethal to multiple myeloma cells [38]. The *IRF4* gene is linked to the BET protein-mediated transcriptional program [39], and its dysregulation is also implicated in lymphoid malignancies during hematopoietic cell differentiation [40]. These suggest a therapeutic hypothesis where *IRF4*-expressing melanoma/lymphoid malignant cells may be accessible through BET inhibitors (BETi).

### Experimental validation of candidate targets showing express-driven dependency

**Figure 6:**
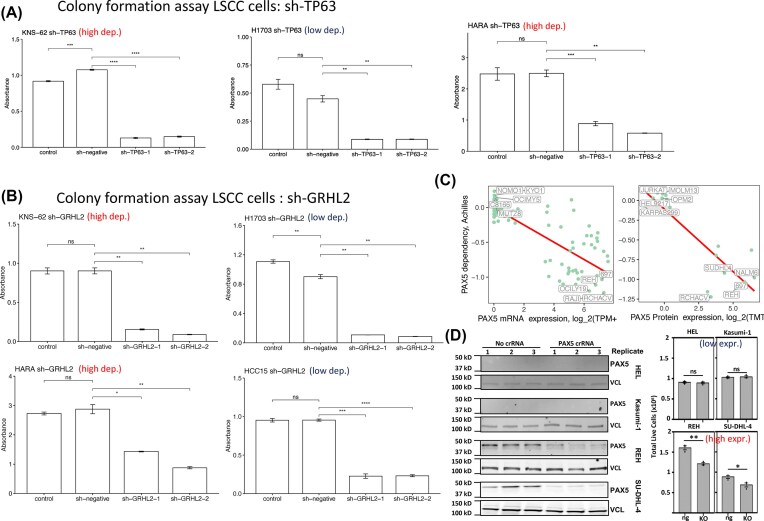
Functional validation of expression-driven dependency targets, *TP63, GRHL2*, and PAX5, in lung squamous cancer cell and hematopoietic cell lines. (A) Colony formation assay in LSCC cell lines (KNS-62, H1703, and HARA) upon knockdown of TP63 using 2 shRNA constructs (sh-TP63-1 and sh-TP63-2). Significant reduction in colony formation was observed compared to sh-negative control cells (*P* < 0.01). ns: non-significance between control and sh-negative cells. Each experiment was performed with 3 replicate wells, where error bars show mean ± standard deviation; this also applies to panel (B). (B) Colony formation assay in LSCC cell lines (KNS-62, H1703, HARA, and HCC15) upon knockdown of GRHL2 using 2 shRNA constructs (sh-GRHL2-1 and sh-GRHL2-2). Significant decrease in colony formation was seen compared to sh-negative control cells (*P* < 0.01). (C) PAX5 mRNA and protein expression levels in myeloid (HEL, Kasumi-1) and B-cell (REH, SU-DHL4) lineage cell lines. PAX5 showed lineage-specific expression-driven dependency. (D) Effect of PAX5 knockout (KO) via CRISPR on cell viability in PAX5-high B-cell lines (REH, SU-DHL4) and PAX5-low myeloid lines (HEL, Kasumi-1). PAX5 KO significantly reduced live cell numbers in REH and SU-DHL4 (*P* < 0.05 and *P* < 0.01, respectively), but not in HEL and Kasumi-1. In (D) left, protein levels were assessed by anti-PAX5 72 h after electroporation. VCL serves as a loading control. In (D) right, cells were electroporated with RNP complexes with (KO) or without (ng) PAX5 crRNA and allowed to recover for 72 h. After recovery, ng and KO cells were reseeded at equal densities, and live cells were counted by trypan blue exclusion after 72 h. Cells were counted in technical triplicate for each biological replicate (*n* = 3).

To experimentally validate GED/PED targets identified by BEACON, we selected 2 types of targets to be tested across 2 lineages: (1) 2 targets showing pan-lineage expression-driven dependency, *GRHL2* and *TP63*, and (2) one target showing lineage-specific expression-driven dependency, *PAX5*. We first confirmed that *TP63* and *GRHL2* mRNA expression were up-regulated in lung squamous cell carcinoma (LSCC) tumor tissue compared to tumor-adjacent normal tissue in TCGA, and chose cultured LSCC cells to conduct validation experiments (see the “Methods” section) (Fig. S6A). We confirmed the inhibition of target gene expression by shRNA using HARA cells with high expression of the candidate genes (Fig. S6B), where qPCR validation showed that the shRNAs reduced TP63 expression to 33% and 17% (for sh1 and sh2, respectively) and GRHL2 expression to 41% and 68% (Fig. S6C). Cell proliferation and colony-forming ability were then measured using 2 types of cells with high dependency (HARA and KNS-62) on candidate genes and 2 types of cells with low dependency (H1703 and HCC15). In KNS-62 and H1703 LSCC cells, the knockdown of *TP63* using 2 shRNA constructs (sh-TP63-1 and sh-TP63-2) resulted in a significant reduction in colony formation and cell viability (reduced proliferation) compared to controls (*P* < 0.01) (Fig. 6A, Fig. S6D). Similarly, *GRHL2* knockdown using sh-GRHL2-1 and sh-GRHL2-2 in both cell lines led to a significant decrease in colony formation and cell viability (*P* < 0.01) (Fig. 6B, Fig. S6E). *TP63* knockdown also resulted in reduced colony formation in HARA cell line (Fig. 6A). The results showed that the knockdown of either gene highly inhibited cell viability and colony formation in LSCC cell lines, regardless of the predicted dependence.

The lineage-specific target, *PAX5*, was evaluated for its role in hematopoietic and lymphoid tissue. Within the lineage, groups of cells with high and low PAX5 expression and low and high PAX5 genetic dependency can be clearly identified by BEACON (Fig. 6C). We chose 2 PAX5-low myeloid lineage cell lines (HEL and Kasumi-1) and 2 PAX5-high (REH and SU-DHL4) B-cell lines to conduct PAX5 knockout (KO) experiments via CRISPR. Upon confirming successful KO via western blots, we showed that PAX5 KO significantly reduced the number of live cells in REH and SU-DHL-4 cell lines compared to controls (*P* < 0.05 and *P* < 0.01, respectively). But PAX5 KO did not significantly inhibit cell survival for HEL and Kasumi-1 (Fig. 6D). Overall, these results show that while *TP63* and *GRHL2* are essential for cell growth across LSCC cells, PAX5 is specifically crucial for the growth of PAX5-high B-cell lymphoma cells. Thus, proteins showing lineage-specific dependencies may present as suitable precision oncology targets in the subset of tumors overexpressing the target gene and protein. However, given the limited scope of our validation of 3 targets, a more systematic validation of GED/PED targets will be required to determine the effectiveness of this target prioritization approach.

## Discussion

This study integrates large-scale CRISPR screen in conjunction with transcriptomic and proteomic data to identify expression-driven dependencies in cancer cells [4, 5], providing a potential new category of targets in precision oncology, particularly against cancer cells without druggable mutations (Fig. 1). Our newly developed Bayesian correlation approach BEACON identified known drug targets and uncovered new candidate genes, demonstrating the utility of expression-driven dependency as a complementary strategy to traditional mutation-driven analyses. Functional experiments demonstrated that targeting genes with high expression levels could reveal potential vulnerabilities within specific cancer types, e.g., PAX5 in lymphoid tumors. We also identified distinct molecular pathways enriched in tissues based on the GED/PEDs, providing insights into the biological processes underpinning cancer progression (Figs 2 and 3). The concept of expression-driven dependency expands the scope of actionable targets by focusing on genes whose high expression levels selectively contribute to cancer cell survival [7–10]. This is particularly relevant in cases where actionable mutations are absent, thereby addressing a significant gap of treatment options in precision oncology [1–3].

By integrating CRISPR/transcriptomic data from CCLE/DepMap [4, 5], and global proteomic analyses [24], we ensured a robust identification of GEDs/PEDs. GEDs and PEDs show significant correlation (*R* = 0.54, *P* < 2e-16) across the cell lines; thus, analyzing the GED/PED can cross-validate the robustness of candidate vulnerability targets (Fig. 4). Our Bayesian approach BEACON further enhanced the reliability of our findings by accommodating variability and limited sample sizes within each tissue lineage (Fig. S2). The identification of GEDs/PEDs has significant implications for drug development and personalized cancer therapy. By targeting genes with high expression levels, new therapeutic avenues can be explored in tumors currently with limited treatment options [1–3]. Although in this study, we emphasized negative associations where higher target expression corresponds to greater dependency, the Bayesian framework is symmetric and can also detect positive correlations, where in rare cases, reduced expression may confer greater vulnerability. This makes BEACON suitable for identifying CYCLOPS-type genes, where reduced expression confers greater vulnerability to perturbation [41]. Notably, the strong enrichment of our identified targets with known druggable gene sets highlights the translational potential of our findings (Fig. 5). At the same time, some of the strongest GED/PED associations revealed by BEACON correspond to lineage-defining TFs. Traditionally, TFs were not easily addressable using small-molecule or antibody-based approaches due to their lack of binding pockets and complex intermolecular interactions. Recent developments in other modalities such as proteolysis-targeting chimeras (PROTACs) will enable testing TFs as potential drug targets [42], particularly in cases where there may be a sufficient therapeutic window in inhibiting these TFs, e.g., to treat adult tumors where the target TFs were only essential in early development and in tumor cells. Importantly, while using large human genomic cohort without phenotypes fails to enrich for drug targets [28], recent human cohort studies demonstrate that genetic evidence provided by genome-wide or Mendelian genetic associations can successfully provide 2- to 5-fold enrichment for drug targets [43, 44]. We note that our approach here, based solely on data from cell line CRISPR screens, provides an orthogonal approach to refine the drug target search space by providing 3.8-fold enrichment for all drug targets and 7- to 10-fold enrichment for oncology targets.

We complemented our computational findings with experimental validation. Knockdown of *TP63* and *GRHL2* genes in lung squamous tumor cell lines demonstrated reduced colony-forming ability, and the *PAX5* knock-out cell lines from hematopoietic and lymphoid tissue samples showed reduced cell growth, reinforcing the functional relevance of the vulnerability targets (Fig. 6). Many GED/PED gene targets are lineage-specific TFs; these agree with recent single-cell studies and synthesis that posited the “developmental constraint model of cancer cell states,” which states that cancer cell states correspond to and may be constrained by the landscape of the “developmental map” [45]. Thus, a cancer cell adopting a specific developmental state may require activation of such TFs and become genetically dependent. While such targets used to be considered undruggable, new drug modalities such as PROTAC are showing strong promise [46–49].

For TP63 and GRHL2, our short-term CCK-8 viability assays showed stronger reductions in proliferation than the effect sizes suggested by published DEMETER shRNA and CERES CRISPR scores. Although the direction of the dependency was fully concordant across all datasets, the magnitude of toxicity differed. Short-term assays can capture acute cellular responses to gene knockdown—such as transient growth delay or stress-induced proliferation defects—that are attenuated or averaged out in the longer-term pooled screens used to generate DEMETER/CERES scores. Furthermore, despite using 2 independent shRNAs for each gene, we cannot entirely exclude minor off-target contributions to the observed effect sizes. Overall, our experiments and DepMap data both support that TP63 and GRHL2 are general functional dependencies in LSCC.

While our study presents a novel approach to identifying cancer dependencies, several limitations warrant discussion. The reliance on cell line models, despite their widespread use, may not fully capture the complexity of tumor heterogeneity and the tumor microenvironment *in vivo*. Future studies should aim to validate these findings in patient-derived xenografts and clinical samples to confirm their translational potential. Moreover, our Bayesian approach BEACON, while robust (Fig. S2), is constrained by the quality and completeness of available data (Fig. S1). Expanding proteomic and transcriptomic datasets that capture the full array of cancer cell heterogeneity across tissue lineages will further improve the reliability of GED/PED identification. It is also important to note that within a given lineage, molecular and clinical subtypes (e.g., ER⁺ vs. ER⁻ breast cancer) may harbor distinct dependencies that could be masked when analyzing at the lineage level. Applying BEACON to subtype-stratified datasets may therefore reveal additional, clinically relevant vulnerabilities. As larger and better-annotated datasets become available, this represents an important direction for future work. Additionally, exploring combination therapies targeting both mutation-driven and expression-driven dependencies could yield synergistic effects, which could be explored in the future. Finally, we acknowledge that the validation experiments we performed employed different perturbation platforms—CRISPR for PAX5 and shRNA knockdown for TP63 and GRHL2. These results should be interpreted as proof of concept rather than direct cross-gene comparisons. Future work using uniform, large-scale perturbation frameworks will be important for fully assessing the robustness and generalizability of BEACON-predicted dependencies.

Overall, our study highlights the potential of expression-driven dependencies as a valuable method for identifying novel therapeutic targets in precision oncology. By integrating multi-omics and CRISPR screen data, we have expanded the repertoire of actionable targets beyond mutated genes for further clinical development, offering new possibilities for cancer treatment.

## Methods

### Data sources

We used the CCLE mRNA expressions data [35] and CCLE quantitative proteomics data [24], and from each dataset we excluded the 26 lineages containing data shared in fewer than 7 cell lines, i.e., adrenal cortex, autonomic ganglia, biliary tract, brain, cervix, colon, eye, fibroblast, melanoma eye(skin), osteosarcoma, placenta, pleura, primary, prostate, salivary gland, skin CJ1(2,3) resistant, skin FV1(2,3) resistant, small intestine, testis, thyroid, and uvea. We used the DepMap Public 22Q2 data release from the DepMap Project [5], which contained the CRISPR knockout screens (Achilles project [25–27]) for 19,221 genes in 1,840 cell lines, including both normal and cancer cell lines, corresponding to 33 primary diseases and 30 lineages. We used the druggable gene lists curated in Minikel et al. [28]. The CRISPR knockout screens and mRNA expressions datasets were downloaded from DepMap portal [50], distributed in the FigShare repository of 22Q2 release [51]. The proteomics datasets were downloaded from Nusinow et al. [24]. The druggable gene lists were downloaded from the corresponding studies given in Minikel et al. [28] and from the DrugBank resource (release 5.1.7).

### mRNA expression-driven dependency (GED)

To measure the expression-driven dependency of targets, we reviewed correlation-based methods utilizing the 2 variables [5, 35, 52], which are adopted to develop a Bayesian approach that we named BEACON. For each gene, BEACON calculated the Bayesian correlation between the gene’s expressions and CERES cancer dependency scores [25] across the pan-lineage cell lines. BEACON modeled expression levels and dependency scores as the bivariate Gaussians and used MCMC sampling to estimate the correlation coefficient *rho* between them. Given the null hypothesis that the uncorrelated expression and dependency of a gene have the 0 *rho* coefficient, we statistically tested each gene’s *rho* estimate obtained from the MCMC simulation as follows. Assume that the MCMC sampling is carried out for a null gene’s expression and dependency, then we expect that the distribution of the *rho* estimate accumulated over the MCMC iterations will be centered at zero. Based on this rationale, we computed the *z*-score of *i*th gene as the deviation of the MCMC estimate of *rho* from the expected (null) value (i.e., zero) in terms of the standard deviation observed in the simulated distribution, i.e., *z*(*i*) = rho_MCMC_(*i*)/SD_MCMC_(*i*). Since the *z*-values, by nature, follow a normal distribution with zero-mean and unit-variance, we computed the *P*-value for each gene’s *rho* estimate as the probability of observing a value as extreme as the computed *z*-value for that gene. We multi-testing corrected the resulting *P*-values using the BH procedure for FDR. Overall, 4,445 genes showed significant pan-lineage expression-driven dependency at the FDR of 0.05. We run the MCMC simulations in R (v4.2: 2022–-04-22) by using packages *rjags* (v4-16) with *JAGS* version (4.3.0), coda (0.19–4.1), and openxlsx (4.2.7.1), with a computing environment on an 8-core processor with 32 GB memory (OS: x86_64-pc-linux-gnu, 64-bit).

Compared to other methods that quantify the relationship between 2 variables, the Bayesian correlation (rho) yielded more intuitive results in the cases with small sample size, while other methods often deviated to spurious correlations imposed by outliers in the data. We benchmarked both methods by simulating expression and dependency datasets at various correlation levels (from −1 to 1, with 0.25 intervals) and sample size (number of cell lines: 5, 7, 10, 20, 30, 60, and 100), with different fractions (0.1, 0.3, 0.5, 0.8, and 1) of samples being outliers (Fig. S2). Through rigorous simulations, we observed that the Bayesian method is better than Pearson correlation for estimating moderate true correlation (|rho| < 0.75) in small sample size (∼10 cell lines or fewer). Bayesian method is also preferable in noisy data (noise level ≥ 0.5, i.e., 50% or more of the samples are corrupted by noise to become outliers), regardless of sample size or true correlation level. For large samples (≥60), both methods have similar performance in all settings. Pearson method is only better at detecting fewer (≤20 cell lines) and highly correlated samples |rho| ≥ 0.75, when there is less noise (≤0.1), while Spearman better captured monotonic non-linear trends, though this advantage largely disappeared in small, noisy cohorts (Fig. S2). Spearman performance degraded substantially under small sample sizes (<10–15 cell lines) or high noise levels (≥30–50% outliers), where rank estimates become unstable. In contrast, BEACON’s Bayesian shrinkage stabilized correlation estimation in precisely these regimes, yielding more accurate estimates for moderate correlations (|ρ| < 0.75) and noisy or limited datasets. Thus, BEACON is most advantageous where lineage-level sample sizes are small, or heterogeneity introduces substantial noise, whereas Spearman remains competitive for larger, cleaner datasets.

We systematically compared BEACON GEDs and PEDs with results from alternative approaches, including Pearson correlation (as used in Project DRIVE and BACON) and Spearman correlation (also used in BACON). Across cancer lineages, BEACON achieved stronger enrichment—measured by higher AUPRC—for known oncogenes and druggable genes, demonstrating that its advantages extend to real data (Fig. S3). We report AUPRC rather than AUROC because AUROC can be misleading under severe class imbalance, where positives (druggable genes) are sparse relative to the large number of negatives. In contrast, AUPRC more appropriately summarizes performance by focusing on the precision–recall tradeoff, making it a more informative metric for evaluating a method’s ability to prioritize true druggable genes among many candidates. The AUPRC values for PED were relatively low across all correlation methods because DRIVE targets are defined using mRNA-based Pearson correlations, whereas PED analysis uses protein expression that is known to only moderately correlate with mRNA levels. Additionally, the smaller number of cell lines with proteomic data requires the BEACON approach to recover known targets in many tissue contexts.

For lineage-wise expression-driven dependency analyses, we stratified by lineage the gene expressions and cancer dependency scores across cell lines, and for each gene, we calculated the Bayesian correlation (and the corresponding P value and FDR as in the pan-lineage case) between the gene’s expressions and cancer dependency scores over the lineage cell lines. In median, 222 GEDs were significant (FDR < 0.05) per lineage. To further identify the lineage-specific targets, we defined a target’s specificity to a given lineage by the difference between the target’s ED score computed within that lineage and the target’s average ED score computed in other lineages.

### Protein expression-driven dependency

We adopted the aforementioned procedures for analyzing the PED. For this, we used the MS proteomics data obtained for 375 cell lines and 22 lineages [24]. For the pan-lineage analysis, we found 872 proteins showing significant expression-driven dependency (FDR < 0.05). For the lineage-wise analyses, we found, in median, 71 proteins per lineage showing significant expression-driven dependency (FDR < 0.05).

### Pathway enrichments from GEDs/PEDs

We used *clusterProfiler* [33] R package (v4.8.3) for functional enrichment analyses of our identified GED and PED sets, and reported the enrichment GO categories at the BH-adjusted *P*-value cutoff of 0.05.

### Association of GEDs/PEDs with drug targets

We tested the association between the drug targets and the genes (proteins) that showed significant (rho < −0.25, FDR < 0.05) pan-lineage ED by using Fisher’s exact test of independence (2-sided). More precisely, given a set of druggable genes, a set of GEDs (PEDs), and the list of total quantified targets in transcriptome (proteome), we calculated the probability of obtaining the observed data and its more extreme deviations in the contingency table consisting of (i) the number of drug targets quantified in the transcriptome (proteome), (ii) the number of GEDs (PEDs) quantified in the transcriptome (proteome), (iii) the number of drug targets quantified in the transcriptome (proteome) that also showed significant ED, and (iv) the remaining number of genes (proteins) that were not drug targets nor showed significant ED, under the null hypothesis that the relative proportions are the same—that the fractions of genes that were drug targets are the same whether the genes show significant ED or not. We found that the pan-lineage GEDs (PEDs) were significantly associated (OR > 2, FDR < 0.05) with 10 (12) of the druggable gene lists in Minikel et al. [28].

## Methods for the experimental validation of *TP63* and *GRHL2*

### Cell culture

The human LSCC cell lines, HARA and KNS62 (The Japanese Cancer Research Resource Bank; JCRB, Osaka, Japan), NCI-H1703 and HCC-15 (kindly provided by Dr. John D. Minna) were used. KNS62 was cultured in E-MEM culture medium (FUJIFILM Wako Pure Chemical Corporation, Osaka, Japan) containing 20% fetal bovine serum (Sigma–Aldrich Japan, Tokyo, Japan) supplemented with 100 U/ml penicillin and streptomycin sulfate (FUJIFILM Wako Pure Chemical Corporation). The other cell lines were cultured in RPMI-1640 culture medium (FUJIFILM Wako Pure Chemical Corporation) containing 10% fetal bovine serum (Sigma–Aldrich Japan) supplemented with 100 U/ml penicillin and streptomycin sulfate (FUJIFILM Wako Pure Chemical Corporation). All cultured cells were incubated at 37°C in a humidified atmosphere of 5% CO2 and maintained in continuous exponential growth by passaging. All cell lines were obtained from the reliable biobanks with authentication (Table S15). Mycoplasma test was performed in regular basis from the first culture of the cells to verify that the cells to be the same as the cells registered.

### Plasmid DNA constructs

The shRNA-targeted sequences were listed in Table S14. For the constructions of plasmids to express shRNA against target genes, double-stranded oligonucleotides were cloned into the pLKO.1-TRC vector (Addgene, #10878). A nonsense scrambled oligonucleotide was used as a negative control. All of the inserted DNA fragments were confirmed by performing DNA sequencing.

### Lentivirus-mediated transient expression of the constructs in LSCCs

HEK293T cells were transfected with the constructed plasmids along with lentiviral packaging plasmids pVSV-G, pMDL/pPRE, and pRSV-REV (Addgene) using a calcium phosphate method. The lentiviral-containing media were collected 72 h after the transfection, filtered through a 0.45 µM filter, then aliquoted and stored at −130°C until use. Cultured LSCC cells were infected with packaged lentiviruses to express shRNA constructs; after 48 h of culture, the cells were treated with 2.5 μg/ml (HCC15) or 5 μg/ml (HARA, KNS-62, H1703) puromycin (Thermo Fisher, # A1113803) and cultured for 24 h (HARA, KNS-62, H1703) or 48 h (HCC15), and used for transient experiments.

### RNA extraction and quantitative PCR analysis

Gene expression levels were examined by quantitative PCR analysis. Briefly, total RNA was isolated from cells using ISOGEN II (Nippon Gene, #311-07361) and purified using RNeasy Mini Kit (Qiagen). Total RNA (500 ng) was reverse transcribed to cDNA using ReverTra Ace™ (TOYOBO, #FSQ-101). Quantitative PCR was performed using primers listed in Table S14, Thunderbird SYBR Green Master Mix (TOYOBO, #QPS-201) and StepOne Plus Real-Time PCR System (Thermo Fisher).

### Cell proliferation and cytotoxic assay

Cell viability was analyzed using the Cell Counting Kit-8 (CCK-8) (Dojindo Laboratories, Kumamoto, Japan: CK04). Cells were seeded 5×103/100 μl per well in 96-well plates. After 1 h incubation at 37°C, 10 μl of CCK-8 solution was added to each well and incubated at 37°C for 2 h. The absorbance was detected at 450 nm using a plate reader (Thermo Fisher Multiskan FC) according to the manufacturer’s instructions. Cell viability was normalized against the sh-negative control after 24 h of transfection, and the data were expressed as a ratio against control after 96 h of transfection.

### Colony formation assay

Cells were seeded 1,300–5,000 cells (5,000 cells for HARA, 1,300 cells for KNS-62, 1,500 cells for H1703, and 2,000 cells for HCC15) per well into 12-well plates (3.8 cm², Corning Japan, Shizuoka, Japan), and cultured for 10 days with the change of culture media every 3 days. The cells were then washed by phosphate-buffered saline (PBS) twice, fixed and stained in 0.2% crystal violet dissolved in 20% ethanol, and incubated for 10 min at room temperature with gentle shaking. After washing by 1 ml of PBS once and by sterilized water 3 times, the plate was air-dried and photographed. To quantify the colony formation, 1 ml of 50% ethanol (pH 4.2, adjusted by hydrochloric acid) was added into each well of 12-well plates, and incubated for 5 min at room temperature with slow shaking, then measured the absorption at 592 nm using a Thermo Fisher Multiskan FC (Thermo Fisher). Each experiment was performed with 3 replicate wells.

### Statistical analysis

Data were analyzed using R version 4.0.3 (The R Foundation for Statistical Computing, Vienna, Austria) in combination with R Studio version 1.2.5033 (R Studio, Boston, MA, USA). Welch 2-sample *t*-test was used to examine the statistical difference between 2 groups.

## Methods for the experimental validation of PAX5

### Tissue culture

All cell lines used in this study were maintained at 37°C with 5% CO^2^. HEL and SU-DHL4 cells were cultured in RPMI 1640 (Corning) supplemented with 10% FetalPlex serum (Gemini), 1% l-glutamine (Corning), and 1% penicillin streptomycin (Gibco). Kasumi-1 cells were cultured in RPMI 1640 (Corning) supplemented with 15% FetalPlex serum (Gemini), 1% l-glutamine (Corning), and 1% penicillin streptomycin (Gibco). REH cells were cultured in IMDM (Gibco) supplemented with 10% heat-inactivated FBS (R&D Systems), 1% l-glutamine (Corning), and 1% penicillin streptomycin (Gibco).

### Genome editing

The CRISPR/Cas9 system was used to genetically engineer cell lines via ribonucleoprotein (RNP) complex delivery as previously described in Layden et al. [53]. Briefly, a crRNA (IDT) targeted to exon 4 of PAX5, CTTTTGTCCGGATGATCCTG, was annealed with tracrRNA (IDT). Control RNP complexes were formed without the crRNA. Annealed gRNAs were incubated with S.p. Cas9 Nuclease (IDT) to form RNP complexes and electroporated into 1.25 million cells per condition using the NEON transfection system (Thermo Fisher). Cells were grown for 72 h, and knockout efficiency was assessed by western blot. Electroporations were performed in biological triplicate for each condition.

### Growth analysis

Cells were allowed to recover for 72 h post-electroporation and then were reseeded to 0.2–0.5 × 10^6^ depending on cell line. Reseeded cells were incubated for 72 h at 37°C with 5% CO2. Cells were mixed with trypan blue (Gibco) and counted with a hemocytometer. Cells were counted in technical triplicate for each biological replicate.

### Western blots

Protein was isolated from cells lysed with RIPA buffer (50 mM Tris pH 8.0, 150 mM NaCl, 1% NP-40, 0.5% sodium deoxycholate, 0.1% SDS) and sonicated before centrifugation. Protein concentration was quantified using the DC Protein Assay kit (Bio-Rad). Equal amounts of protein were boiled in Laemmli buffer and run on SDS-PAGE (sodium dodecyl sulfate-polyacrylamide gel electrophoresis) gels. Proteins were transferred to a PVDF (Polyvinylidene difluoride) membrane, and membranes were blocked in 5% BSA in PBS. Membranes were incubated with the indicated primary antibodies diluted in 5% BSA in PBS-T followed by incubation with IRDye 800CW and 680RD secondary antibodies (LI-COR Biosciences) diluted in PBS-T + 0.01% SDS. Blots were imaged using the Odyssey Imaging System (LI-COR Biosciences). The following primary antibodies were used at a 1:1000 dilution: PAX5 (Santa Cruz; A-11) and VCL (Santa Cruz; 7F9). All secondary antibodies (IRDye 680 Donkey anti-Rabbit and IRDye 800 Donkey anti-Mouse, Licor) were used at a 1:5000 dilution.

### Statistics

Statistical analyses were performed in R (version 4.1.0). Unpaired 2-sample *t*-tests were used to determine significance between conditions.

## Availability of source code and requirements

Project name: BEACON

Project homepage: https://github.com/Huang-lab/BEACON

Operating system: Linux, macOS, or Windows (cross-platform compatible)

Programming language: R (version 4.2.0 or later)

Other requirements: JAGS (Just Another Gibbs Sampler) version 4.x or later; R packages: rjags (4-16), openxlsx (4-2), coda (0.19-4)

License: MIT license

RRID:SCR_027484

## Additional files

**Supplementary Figure S1**. Data overview. (A) Analyses were restricted to lineages with at least 7 cell lines having cancer cell line dependency and corresponding mRNA/protein expression data to ensure statistical robustness. (B) 855 cell lines across 17 lineages were analyzed, sharing cancer cell dependency scores and corresponding mRNA and protein expressions. The limited sample size per cell lineage may lead to spurious correlations, especially for protein expression. (C) The distribution of protein quantification per cell line. Over 12,000 proteins (in total) were quantified across all samples, where a majority of the samples reached a quantification level of over 9,000 proteins [24].

**Supplementary Figure S2**. Benchmarking of BEACON against Pearson and Spearman correlations in simulated data. The performance is measured by mean squared error (MSE, *y*-axis) for the same data sets randomly simulated for various true correlation levels (rho, *x*-axis), under different conditions of noise interference (columns, 0.1, 0.3, 0.5, 0.8, 1) and sample size (rows, 5, 7, 10, 20, 30, 60, 100).

**Supplementary Figure S3**. Systematic benchmarking of BEACON against Pearson and Spearman correlations in real data. (A) AUPRC values for identifying DGIdb druggable genes (*n* = 2,993) (left) and the transcription factors previously identified in Project DRIVE (*n* = 57) (right) based on gene expression–dependency (GED) associations across 23 cancer lineages. (B) AUPRC values for identifying the same druggable gene sets in panel (A) based on protein expression–dependency (PED) associations across 17 lineages.

**Supplementary Figure S4**. Analysis of druggable gene expression dependencies (GEDs) and protein expression dependencies (PEDs). (A) Heatmap illustrating pan-lineage and lineage-specific druggable gene expression-driven dependencies (GEDs) across various cancer types. Each square represents the correlation (rho) between gene expression and dependency (CERES scores) in the respective tissue types. Significant dependencies are highlighted with bold outlines (FDR < 0.05 in black, FDR < 0.15 in gray). Integration of the drug–gene interaction database (DGIdb) identified 81 druggable factors showing pan-lineage GED and 927 tissue-specific druggable targets, in total, showing significant GED across all lineages. (B) Analysis of tissue-specific expression-driven dependencies across tissues revealed 927 significant druggable targets, including 132 for hematopoietic and lymphoid tissue, and 97 for lung. (C) Clustering GED measures of genes across tissue types showed that pancreatic, large intestine, and biliary tract cancer cells share the most similar expression-driven dependency profiles. (D) The breast-specific *ESR1* transcription factor clustered with *IRX5* and *GATA3*, showing strong GED levels in breast tissue cell lines. (E) Integration of DGIdb for PEDs identified 152 significant lineage-specific PEDs, with notable targets including PAX5 in hematopoietic and lymphoid tissue, and JMJD6 in the central nervous system.

**Supplementary Figure S5**. Enrichment (Fisher’s exact test) results demonstrating the enrichment of identified GEDs and PEDs in druggable gene lists based on DrugBank (likely mechanism of action) and genetic effect gene lists as described in Methods.

**Supplementary Figure S6**. Dependencies of *GRHL2* and *TP63* in lung squamous cell carcinoma (LSCC) cell lines. (A) mRNA expression levels of *GRHL2* and *TP63* in TCGA LUSC tumors vs. normal tissues in TCGA, where both genes show elevated expression in LSCC compared to normal adjacent lung tissue. (B) Scatter plots showing the relationship between *TP63* mRNA and protein expression levels vs. gene dependency (CERES score) across various cell lines. (C) qPCR result of shRNA-infected HARA cell line, showing the reduced expression levels of *TP63* and *GRHL2* compared to untreated cells. (D) CCK-8 cell proliferation assays in LSCC cell lines (KNS-62 and H1703) upon knockdown of *TP63* using 2 shRNA constructs (sh-TP63-1 and sh-TP63-2). Proliferation is shown relative to day 1. Data represent mean ± SD from 3 independent replicate wells (*N* = 3), where error bars indicate standard deviation (SD). This also applies to panel (E). (E) CCK-8 cell proliferation assays in LSCC cell lines (KNS-62 and H1703) upon knockdown of *GRHL2* using 2 shRNA constructs (sh-GRHL2-1 and sh-GRHL2-2). Proliferation is shown relative to day 1.

**Supplementary Data**. Spreadsheets containing the supplementary tables (S1–S15), including the related data used for plotting main figures and supplementary figures.

**Supplementary Table S1**. Data availability for all lineage cell lines.

**Supplementary Table S2**. BEACON results of pan-lineage GEDs (tissue-specific GEDs—sheet2).

**Supplementary Table S3**. BEACON results of pan-lineage druggable GEDs (tissue-specific druggable GEDs—sheet2).

**Supplementary Table S4**. Proteins showing significant (rho < –0.25, FDR < 0.05) pan-lineage PED with significant GED (without a significant GED—sheet2).

**Supplementary Table S5**. BEACON results of pan-lineage druggable PEDs.

**Supplementary Table S6**. BEACON results of tissue-specific PEDs (tissue-specific druggable PEDs—sheet2).

**Supplementary Table S7**. Gene ontology enrichment analysis for the tissue-specific GEDs (for the tissue-specific PEDs—sheet2).

**Supplementary Table S8**. Genes showing consistently significant (rho < −0.25, FDR < 0.05) pan-lineage expression-driven dependency in both mRNA and protein levels (in only mRNA levels—sheet2).

**Supplementary Table S9**. Table showing consistency between tissue-level GEDs and PEDs for each lineage.

**Supplementary Table S10**. Results of Fisher’s exact test evaluating the association between the druggable gene lists and the pan-lineage GEDs/PEDs.

**Supplementary Table S11**. GEDs/PEDs enriched for druggable targets curated by the DrugBank.

**Supplementary Table S12**. Druggable genes significantly enriched with expression-driven dependency observed in both mRNA and protein level expressions.

**Supplementary Table S13**. Other genes (not drug targets) significantly enriched with expression-driven dependency observed in both mRNA and protein level expressions.

**Supplementary Table S14**. The shRNA-targeted sequences and primers used for LSCC experiments.

**Supplementary Table S15**. Cell line source table.

## Use of AI tools and technologies in writing

During the preparation of this work, the authors used ChatGPT, Perplexity, and Claude in order to refine language and assist with editing the authors’ originally written content for improved readability. After using this tool/service, the authors reviewed and edited the content as needed and take full responsibility for the content of the publication.

## Supplementary Material

giag011_Supplemental_Files

giag011_Authors_Response_To_Reviewer_Comments_original_submission

giag011_Authors_Response_To_Reviewer_Comments_revision_1

giag011_GIGA-D-25-00147_original_submission

giag011_GIGA-D-25-00147_Revision_1

giag011_GIGA-D-25-00147_Revision_2

giag011_Reviewer_1_Report_original_submission Reviewer 1 -- 6/13/2025

giag011_Reviewer_1_Report_Revision_1 Reviewer 1 -- 10/3/2025

giag011_Reviewer_2_Report_original_submission Reviewer 2 -- 6/30/2025

giag011_Reviewer_2_Report_Revision_1 Reviewer 2 -- 10/25/2025

giag011_Reviewer_3_Report_original_submission Reviewer 3 -- 7/7/2025

giag011_Reviewer_4_Report_original_submission Reviewer 4 -- 7/14/2025

giag011_Reviewer_4_Report_Revision_1 Reviewer 4 -- 10/8/2025

## Data Availability

All additional supporting data are available in the GigaScience repository, GigaDB [54].
