# Supplementary material for: Expression-driven genetic dependency reveals targets for precision oncology
Source: Gigascience. 2026 Jan 29;15:giag011. doi: 10.1093/gigascience/giag011 (PMC12970598; doi:10.1093/gigascience/giag011)
Supplement: giag011_Supplemental_Files [file giag011_supplemental_files.zip › expression_dependency_figures_supp.pdf]

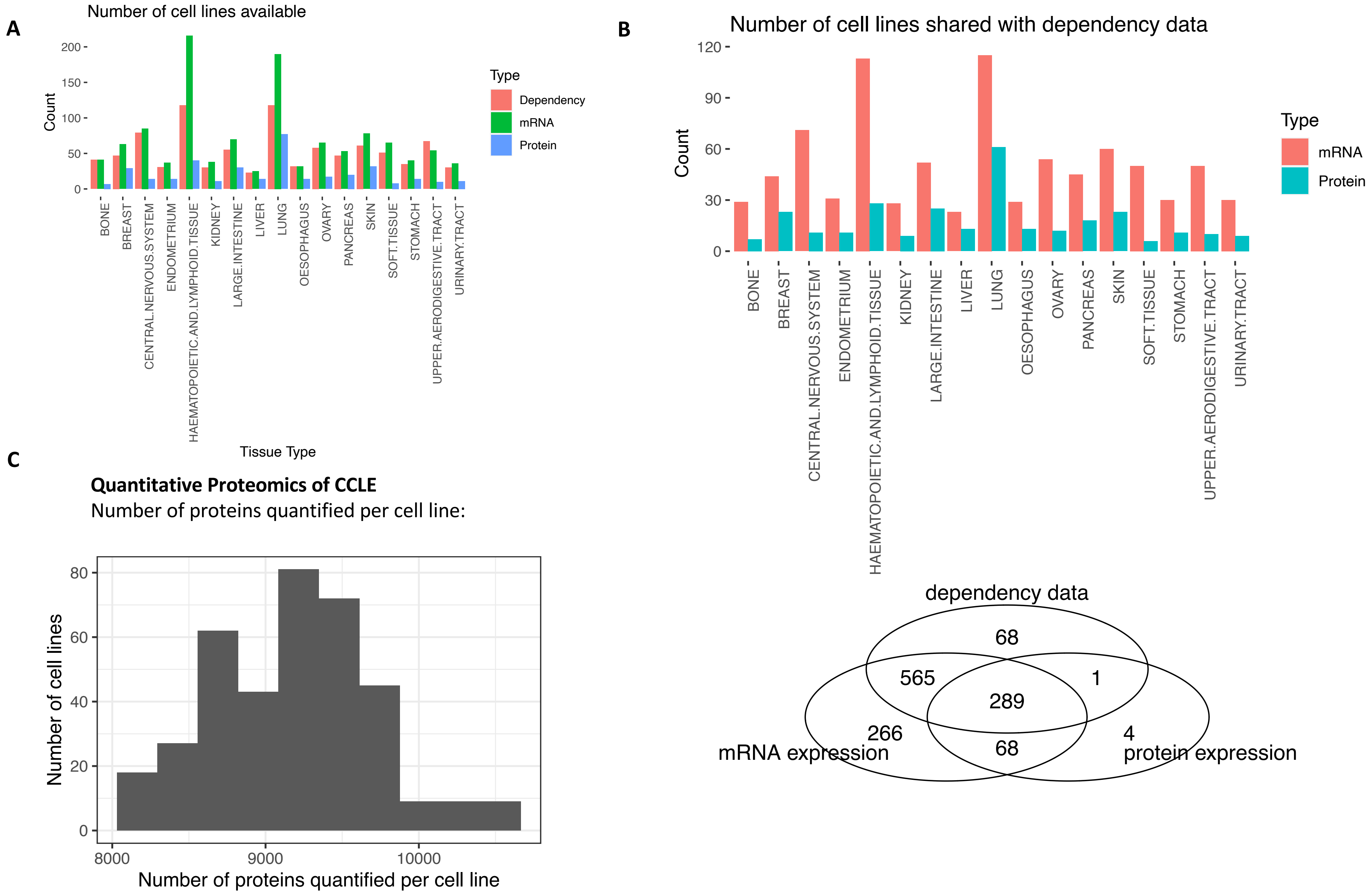

Figure S1. Data overview. (A) Analyses were restricted to lineages with at least 7 cell lines having cancer cell line dependency and corresponding mRNA/protein expression data to ensure statistical robustness. (B) 855 cell lines across 17 lineages were analyzed, sharing cancer cell dependency scores and corresponding mRNA and protein expressions. The limited sample size per cell lineage may lead to spurious correlations, especially for protein expression. (C) The distribution of protein quantification per cell line. Over 12,000 proteins (in total) were quantified across all samples, where a majority of the samples reached a quantification level of over 9,000 proteins.

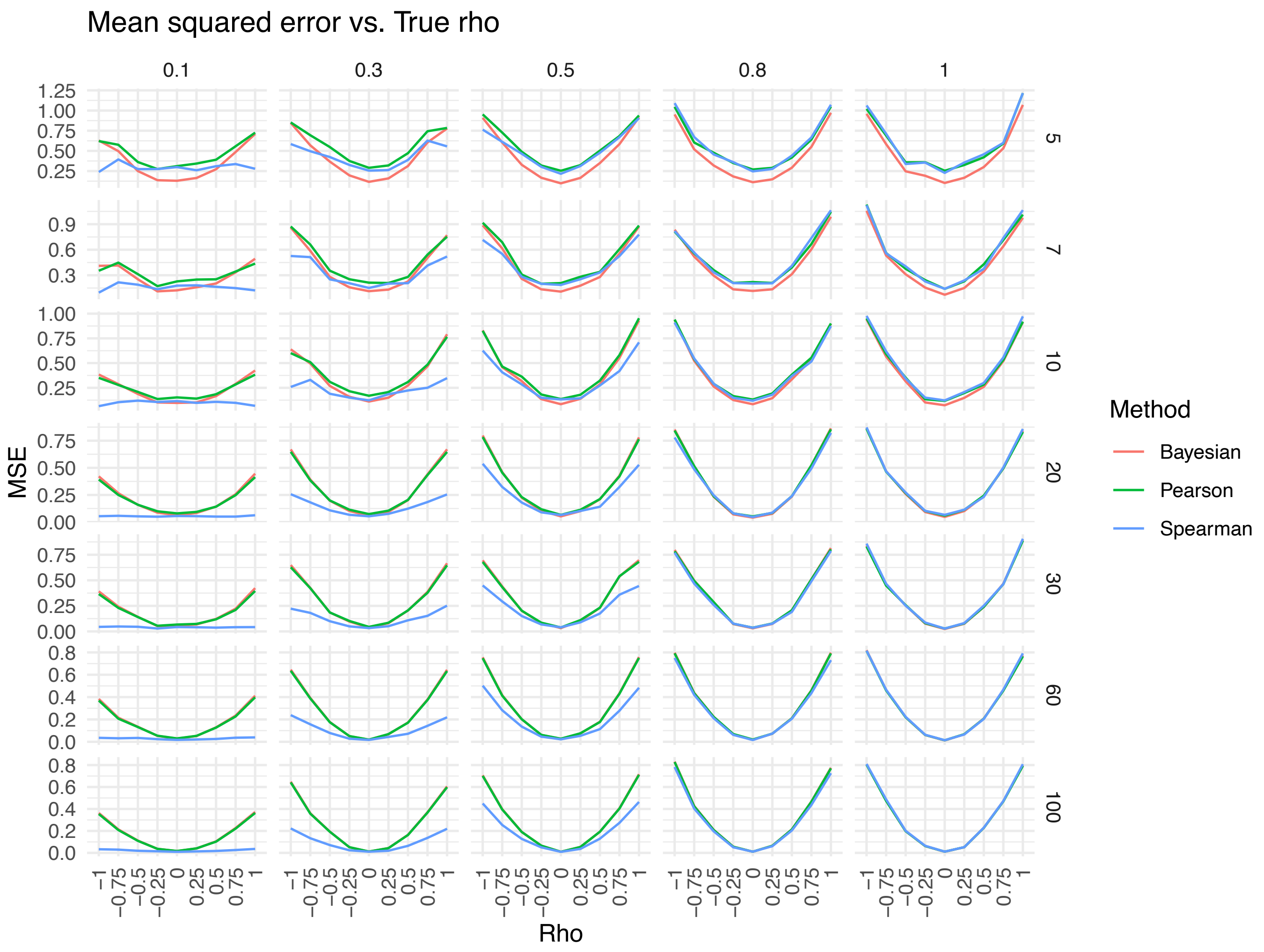

Figure S2. Benchmarking of BEACON against Pearson and Spearman correlations in simulated data. The performance are measured by mean squared-error (MSE, y-axis) for the same data sets randomly simulated for various true correlation levels (rho, x-axis), under different conditions of noise interference (columns, 0.1, 0.3, 0.5, 0.8, 1) and sample size (rows, 5, 7, 10, 20, 30, 60, 100).

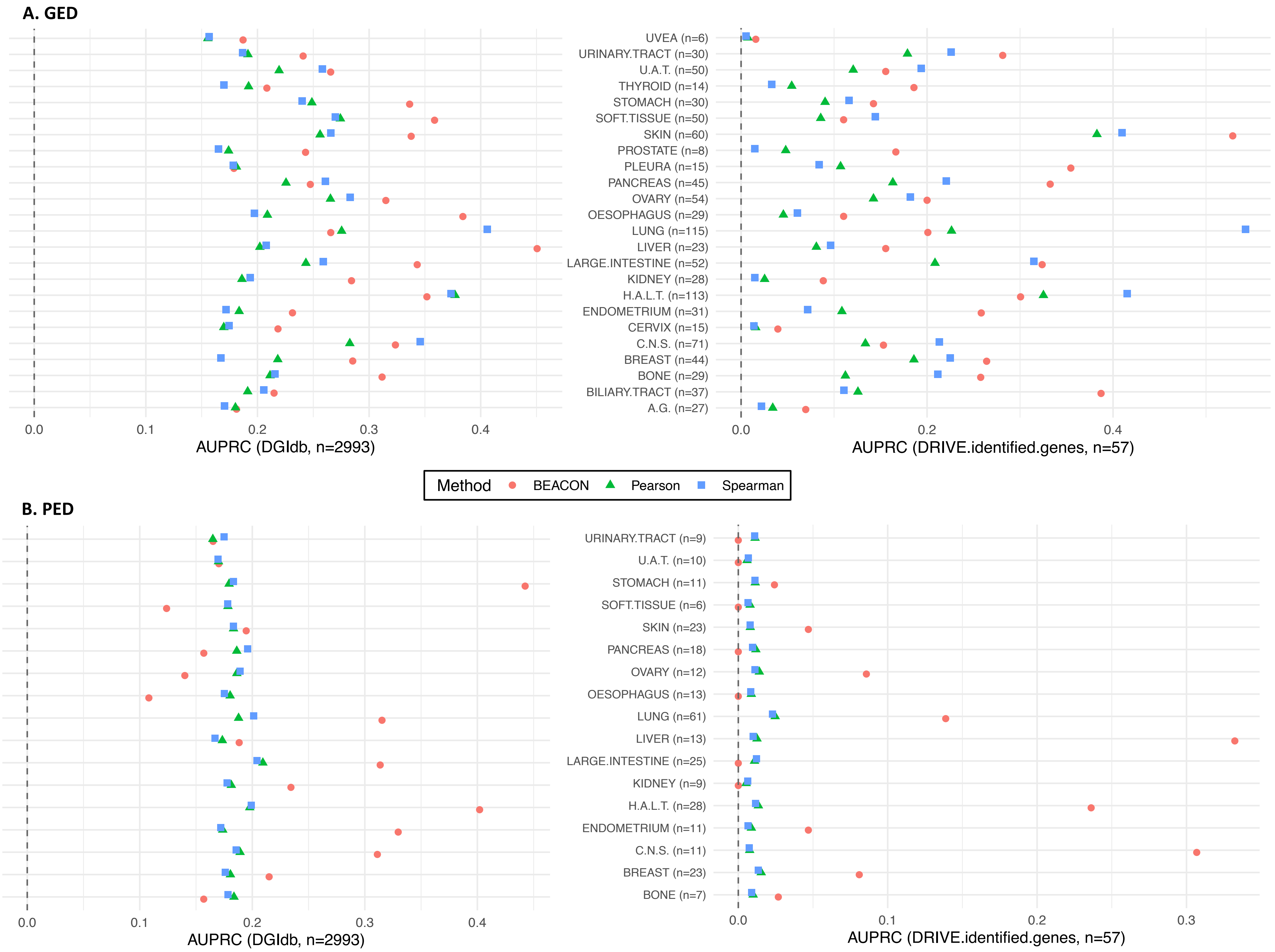

Figure S3. Systematic benchmarking of BEACON against Pearson and Spearman correlations in real data.
(A) AUPRC values for identifying DGIdb druggable genes (n = 2,993) (left) and the transcription factors previously identified in Project DRIVE (n = 57) (right) based on gene expression–dependency (GED) associations across 24 cancer lineages.
(B) AUPRC values for identifying the same druggable gene sets in *panel A* based on protein expression–dependency (PED) associations across 17 lineages.

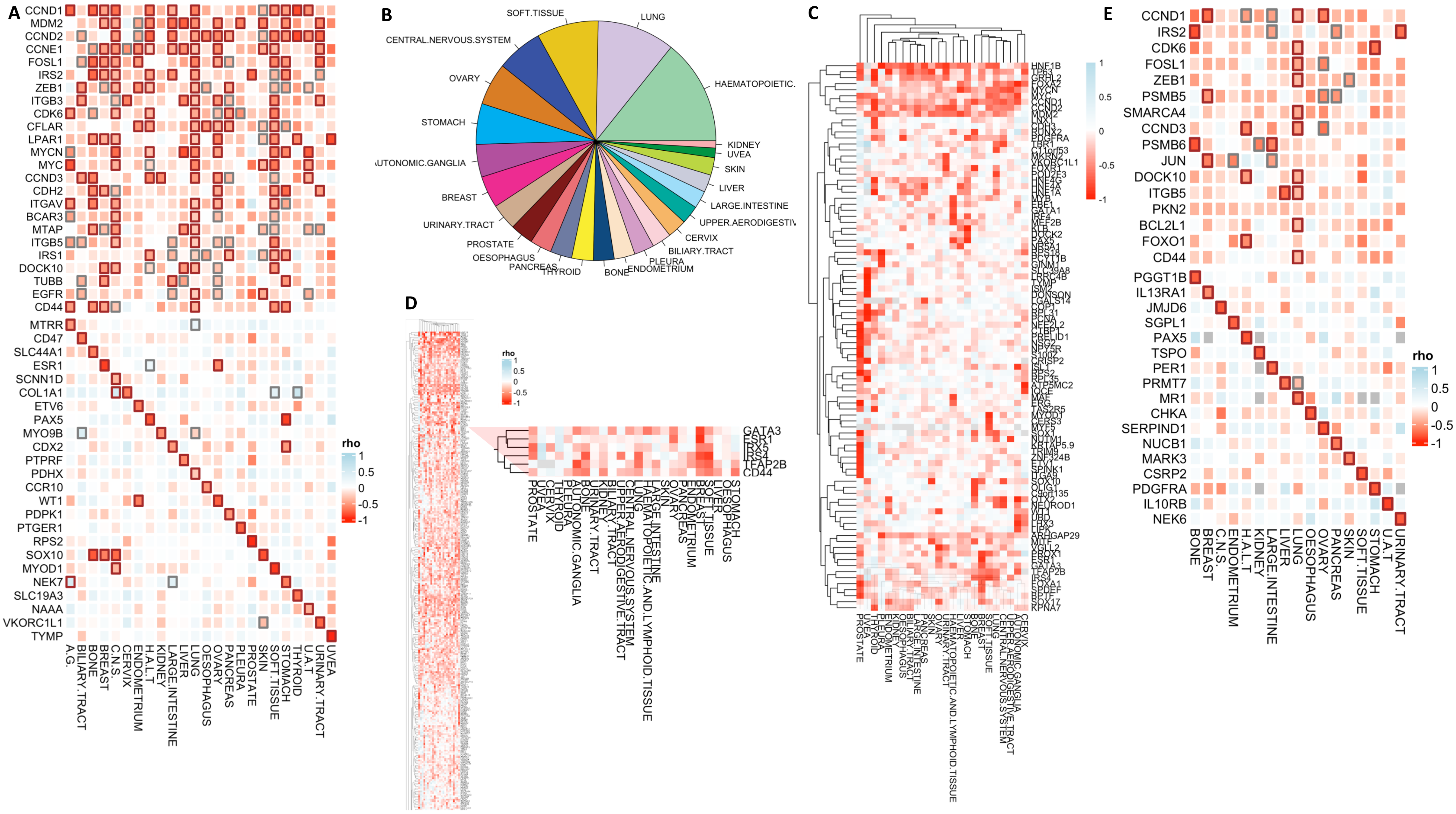

Figure S4. Analysis of Druggable Gene Expression Dependencies (GEDs) and Protein Expression Dependencies (PEDs). (A) Heatmap illustrating pan-lineage and lineage-specific druggable gene expression-driven dependencies (GEDs) across various cancer types. Each square represents the correlation (rho) between gene expression and dependency (CERES scores) in the respective tissue types. Significant dependencies are highlighted with bold outlines (FDR < 0.05 in black, FDR < 0.15 in grey). Integration of the drug-gene interaction database (DGIdb) identified 81 druggable factors showing pan-lineage GED and 927 tissue-specific druggable targets, in total, showing significant GED across all lineages. (B) Analysis of tissue-specific gene expression-driven dependencies across tissues revealed 927 significant druggable targets, including 132 for hematopoietic and lymphoid tissue, and 97 for lung. (C) Clustering GED measures of genes across tissue types showed that pancreatic, large intestine, and biliary tract cancer cells share the most similar expression-driven dependency profiles. (D) The breast-specific *ESR1* transcription factor clustered with IRX5 and GATA3, showing strong GED levels in breast tissue cell lines. (E) Integration of DGIdb for PEDs identified 152 significant lineage-specific PEDs, with notable targets including PAX5 in hematopoietic and lymphoid tissue, and JMJD6 in the central nervous system.

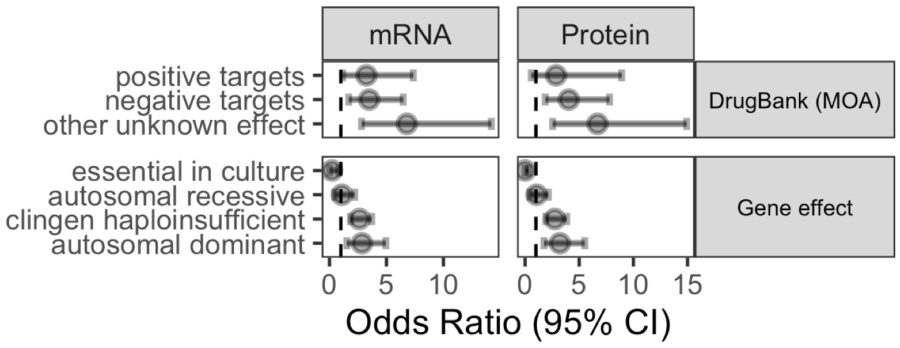

Figure S5. Enrichment (Fisher's exact test) results demonstrating the enrichment of identified GEDs and PEDs in druggable gene lists based on DrugBank (likely mechanism of action) and genetic effect gene lists as described in Methods.

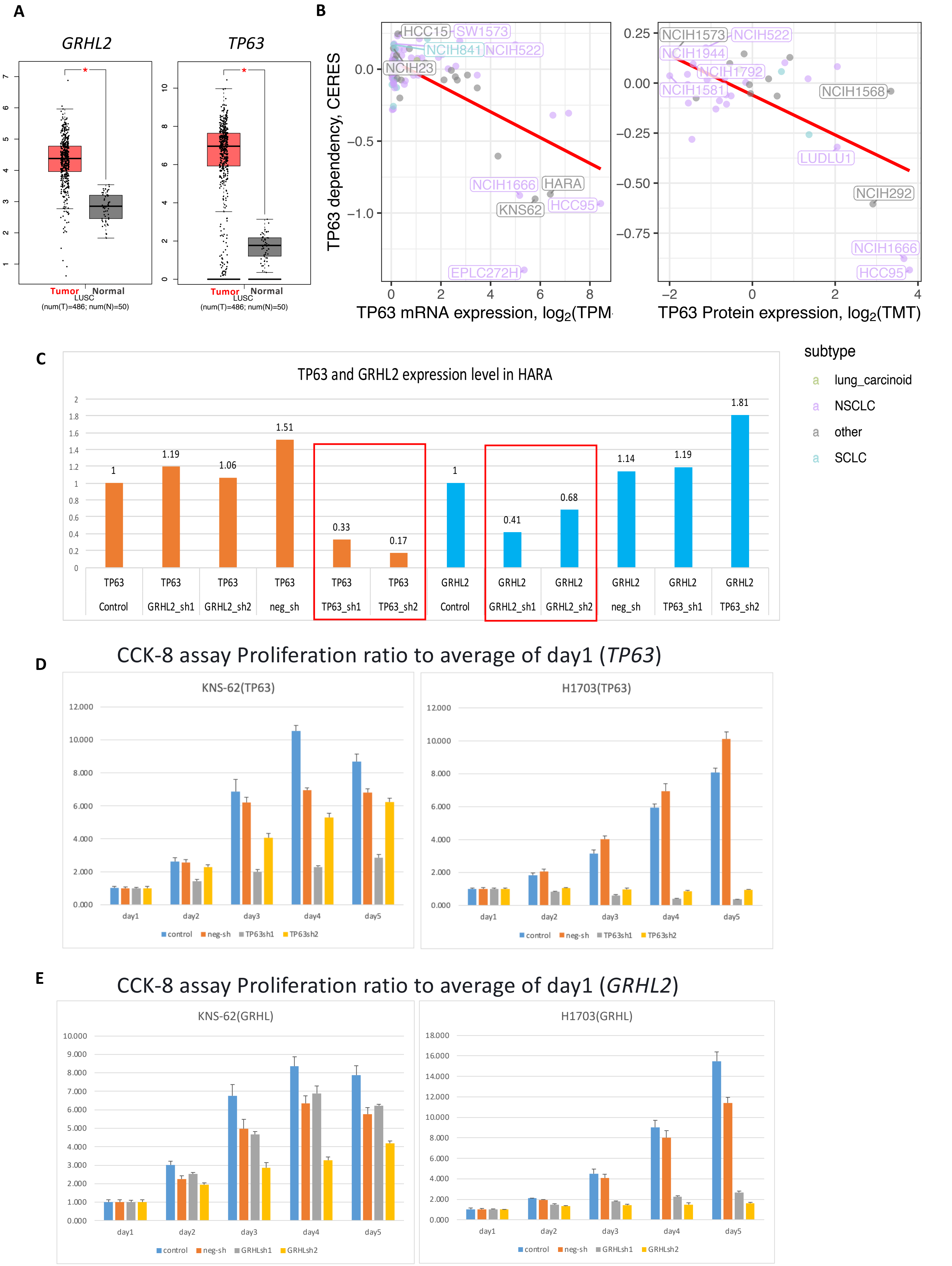

Figure S6. Dependencies of *GRHL2* and *TP63* in lung squamous cell carcinoma (LSCC) cell lines. (A) mRNA expression levels of *GRHL2* and *TP63* in TCGA LUSC tumor vs. normal tissues in TCGA, where both genes show elevated expression in LSCC compared to normal adjacent lung tissue. (B) Scatter plots showing the relationship between *TP63* mRNA and protein expression levels vs. gene dependency (CERES score) across various cell lines. (C) qPCR result of shRNA infected HARA cell line, showing the reduced expression levels of TP63 and GRHL2 compared to untreated cells. (D) CCK-8 cell proliferation assays in LSCC cell lines (KNS-62 and H1703) upon knockdown of *TP63* using two shRNA constructs (sh-TP63-1 and sh-TP63-2). Proliferation is shown relative to day 1. Data represent mean ± SD from three independent replicate wells (N = 3), where error bars indicate standard deviation (SD). This also applies to *panels E*. (E) CCK-8 cell proliferation assays in LSCC cell lines (KNS-62 and H1703) upon knockdown of *GRHL2* using two shRNA constructs (sh-GRHL2-1 and sh-GRHL2-2). Proliferation is shown relative to day 1.
