# Supplementary material for: Expression-driven genetic dependency reveals targets for precision oncology
Source: Gigascience. 2026 Jan 29;15:giag011. doi: 10.1093/gigascience/giag011 (PMC12970598; doi:10.1093/gigascience/giag011)
Supplement: giag011_GIGA-D-25-00147_Revision_1 [file giag011_giga-d-25-00147_revision_1.pdf]

GigaScience

Expression-Driven Genetic Dependency Reveals Targets for Precision Oncology

--Manuscript Draft--

Manuscript Number:	GIGA-D-25-00147R1
Full Title:	Expression-Driven Genetic Dependency Reveals Targets for Precision Oncology
Article Type:	Research
Funding Information:	National Institute of General Medical Sciences (R35GM138113) — Dr Kuan-lin Huang<br>American Cancer Society (RSG-22-115-01-DMC) — Dr Kuan-lin Huang<br>National Institute of General Medical Sciences (2R35GM138113) — Dr Kuan-lin Huang
Abstract:	Background: Cancer cells are heterogeneous, each harboring distinct molecular aberrations and are dependent on different genes for their survival and proliferation. While targeted therapies based on driver DNA mutations have shown success, many tumors lack druggable mutations, limiting treatment options. We hypothesize that new precision oncology targets may be identified through "expression-driven dependency," where cancer cells with high expression of specific genes are more vulnerable to the knockout of those same genes.<br>Results: We developed BEACON, a Bayesian approach to identify expression-driven dependency targets by analyzing global transcriptomic and proteomic profiles alongside genetic dependency data from cancer cell lines across 17 tissue lineages. BEACON successfully identified known druggable genes including BCL2, ERBB2, EGFR, ESR1, and MYC, while revealing novel targets confirmed by both mRNA and protein-expression driven dependency. The identified genes showed a 3.8-fold enrichment for approved drug targets and a 7 to 10-fold enrichment for druggable oncology targets. Experimental validation demonstrated that depletion of GRHL2, TP63, and PAX5 reduced tumor cell growth and survival in their dependent cells.<br>Conclusions: We provide a systematic approach to identify precision oncology targets based on expression-driven dependency patterns. By integrating multi-omics data with genetic dependency screens, BEACON generated a comprehensive catalog of potential therapeutic targets that may expand treatment options for cancer patients lacking druggable mutations. This resource offers new opportunities for precision oncology target discovery beyond mutation-based approaches.
Corresponding Author:	Kuan-lin Huang, PhD<br>Icahn School of Medicine at Mount Sinai<br>New York, NY UNITED STATES
Corresponding Author Secondary Information:	
Corresponding Author's Institution:	Icahn School of Medicine at Mount Sinai
Corresponding Author's Secondary Institution:	
First Author:	Abdulkadir Elmas
First Author Secondary Information:	
Order of Authors:	Abdulkadir Elmas<br>Hillary M. Layden<br>Jacob D. Ellis<br>Luke N. Bartlett<br>Xian Zhao<br>Reika Kawabata-Iwakawa

Powered by Editorial Manager® and ProduXion Manager® from Aries Systems Corporation

	Hideru Obinata
	Scott W. Hiebert
	Kuan-lin Huang, PhD
**Order of Authors Secondary Information:**	
**Response to Reviewers:**	Authors: We sincerely thank the reviewers for the thoughtful and constructive feedback on our manuscript entitled “Expression-Driven Genetic Dependency Reveals Targets for Precision Oncology” (GIGA-D-25-00147). We have carefully considered the comments, particularly regarding the need to benchmark BEACON against established correlation-based approaches and demonstrate its improvements in real data, and have conducted additional systematic benchmarking analyses to address these concerns. We are pleased to submit a revised version of the manuscript for your consideration. Below is a point-by-point response to the reviewers’ comments.<br><br>Reviewer #1: The authors present BEACON, a method for identifying associations between the expression of a gene and sensitivity to the CRISPR knockout of that gene across a panel of cancer cell lines. These 'oncogene like' dependencies represent potential therapeutic targets that might be exploited for the development of new precision medicines in cancer. The issue that BEACON aims to address is the limited sample size (cell line count) in some specific cancer lineages and experimental noise that might result in spurious correlations between expression and CRISPR sensitivity. The authors demonstrate, using a modelling approach, that BEACON is more reliable for estimating correlation than simple Pearson's correlation when there is high-noise in the measurements. The majority of the manuscript focuses on analyses of dependencies systematically identified using the BEACON approach and their enrichment in drug targets and biological pathways. There is some experimental testing of three potential expression driven dependencies presented. The rationale for the overall approach and analyses are clear.<br><br>Authors: We sincerely thank the reviewer for their thoughtful and constructive evaluation of our manuscript. In response, we expanded our benchmarking analyses and show that BEACON consistently outperforms Pearson and Spearman correlations in identifying known druggable targets using real data, particularly in smaller and noisier lineages. We also clarified and consolidated our experimental validation results to ensure transparency.<br><br>Major comments<br><br>- Previous efforts have systematically associated gene/protein expression with CRISPR sensitivity across the same or related datasets (e.g. Pacini et al, Cancer Cell 2024 and Rohde et al, Molecular Systems Biology 2025 using CRISPR; McDonald et al, Cell 2017 and Tsherniak et al, Cell 2017 using RNAi) and so the primary contribution of this paper can be considered the development of the BEACON method. It is thus somewhat surprising that there is no real assessment of the improvements offered by BEACON when compared to simpler methods (Pearson correlation, Spearman correlation) or more more complex recent approaches (Rohde et al's BACON approach). The modelling approach suggests some improvements in specific circumstances (especially high noise) but it is not clear that this leads to improved dependency identification in the real data. Does BEACON identify known oncogene addictions better than these methods? Are the associations identified more reproducible (e.g. across alternative CRISPR screens or RNAi screens)?<br><br>Authors: We thank the reviewer for this validate suggestion; we validated BEACON against real data and incorporated these results in page 5-6:<br>To further validate BEACON on real data, we systematically benchmarked its performance against Pearson and Spearman correlations to identify a curated set of 2,993 druggable genes from DGIdb as the reference standard using this DepMap CRISPR dataset. andard. For each cancer lineage, we calculated the area under the precision–recall curve (AUPRC) for identifying DGIdb genes based on expression–dependency correlation scores. On average across all lineages, BEACON achieved an AUPRC improvement of 25/29% over Pearson and 23/29% over Spearman correlations (Figure S3), based on CRISPR vs. mRNA/protein expression data. Specifically, BEACON was the top-performing method in 17 of 24 lineages for

GED (mRNA) and in 10 of 17 lineages for PED (protein) (Figure S3). The advantage was particularly pronounced in lineages with smaller sample sizes (e.g., Cervix, Oesophagus, Stomach, Endometrium, etc.), where AUPRC gains reached more than two-fold over Pearson/Spearman. An additional benchmarking against the 57 prioritized genes identified by Project DRIVE's expression–dependency model (Pearson-based)29 showed that BEACON achieved average AUPRC gains of 94/530% over Pearson and 123/616% over Spearman correlation (Figure S3). BEACON was also the top-performing method in 18 of 24 lineages for GED (mRNA) and in 11 of 17 lineages for PED (protein). These results demonstrate that BEACON improves over simpler correlation measures and enhances the recovery of biologically validated dependencies.

- The experimental validation and the conclusions drawn from it are somewhat confusing. The authors assess three potential expression associated dependencies - two pan-cancer dependencies (GRHL2 and TP63) and one lineage specific dependency (PAX5 in myeloid cells). Only the lineage-specific dependency validated in the way that might be expected, with higher expression associated with increased dependency, leading the authors to conclude that lineage-specific dependencies may be more suitable targets than pan-cancer ones. Given the numbers analysed (3 genes) this suggestion is not well supported. Moreover the perturbation was performed using distinct approaches - CRISPR for PAX5 and shRNA for the other two genes - and only the knockdown of PAX5 was validated by Western blot. It is very hard to know what phenotypes might be a false positive from off-target shRNA effects or false-negatives from variable shRNA knockdown of the target. The results in S5C suggest that the two shRNAs for each gene cause somewhat discordant phenotypes, suggesting there may be some issues with knockdown efficiency. This could potentially be addressed by adding additional shRNAs for GRHL2 / TP63 or testing them using CRISPR perturbation as was done for PAX5. Validation of the knockdown of the intended target could also shed some light here. The manuscript also mentions experiments in an additional cell line (HCC15) but I cannot see these results presented in the main figures or supplement. It would be useful if all results for these two genes were presented in a single figure, with high and low expressing cell lines clearly marked,

Authors: Our rationale was to evaluate both pan-cancer and lineage-specific dependencies, as BEACON identifies candidates at both levels. While GRHL2 and TP63 appeared as pan-cancer GEDs, their protein-level PED associations were most pronounced in lung cancer, which led us to test them in lung cancer cell lines, whereas PAX5 was pursued as a clear lineage-specific dependency in hematopoietic and lymphoid cells. We acknowledge that the use of different perturbation methods—CRISPR for PAX5 and shRNA for GRHL2 and TP63—complicates direct comparison, but this choice was driven by feasibility and resources with the two collaborative teams at Vanderbilt and Gunma University who specialized in lymphoid and lung cancers, respectively. We also recognize the variability in shRNA efficacy and for transparency have added this to results:
To confirm inhibition of the target genes, qPCR validation in HARA cells showed that knockdown efficiency in the HARA cell line where the expression levels of TP63 and GRHL2 were reduced to 33/17% and 41/68%, respectively.

We now also present all replicate results (including HCC15 and HARA) in a single consolidated figure (Figure 6B) with high- and low-dependency cell lines clearly labeled so that the data can be interpreted transparently. Finally, we added a caveat at the end of this Result section:
However, given the limited scope of our validation of three targets, a more systematic validation of GED/PED targets will be required to determine the effectiveness of this target prioritization approach.

Minor:

- Previous work has established that in some cases lower expression of a gene can make cells more vulnerable to its perturbation (CYCLOPS genes, Nijhawan et al, Cell 2012). While these are not the focus of this manuscript, it would be useful for the authors to comment on the utility of BEACON for their identification.

Powered by Editorial Manager® and ProduXion Manager® from Aries Systems Corporation

Authors: We appreciate this helpful suggestion. Indeed, BEACON can in principle identify not only "negative" correlations (with negative rho, due to dependency score being negative) between expression and dependency (GED/PED, where higher expression is associated with stronger dependency) but also "positive" correlations, such as those characteristic of CYCLOPS genes, where lower expression increases vulnerability to perturbation. We have added a brief comment in the Discussion (page 15, lines 428-432):
Although in this study we emphasized negative associations where higher target expression corresponds to greater dependency, the Bayesian framework is symmetric and can also detect positive correlations. This makes BEACON suitable for identifying CYCLOPS-type genes, where reduced expression confers greater vulnerability to perturbation42.

- p14 "Moreover, GED/PED targets were depleted of genes that were Essential In Culture" - it's not clear what this means or where the data comes from. By definition the gene set analysed are at least somewhat essential in culture

Authors: The “Essential In Culture” gene set was as defined by Hart et al. 2017. We have revised the text to clarify this point and now state (page 12, lines 338-342):
Moreover, GED/PED targets were underrepresented among the common essential genes (N=684 “Essential In Culture” genes based on 17 genome-wide CRISPR screens38), suggesting that BEACON identifies cell-specific vulnerabilities rather than dependencies universally required for cell viability (e.g., house-keeping genes) that could lead to off-target effects.

Reviewer #2: *The authors introduce BEACON, a Bayesian correlation approach designed to identify expression-driven dependency in cancer. Their hypothesis suggests that cancer cells with elevated expression of specific genes demonstrate increased vulnerability to the knockout of those same genes, thereby unveiling a promising new category of targets in precision oncology—particularly valuable for targeting cancer cells lacking druggable mutations.
*BEACON models expression levels and dependency scores as bivariate Gaussians and employs Markov Chain Monte Carlo (MCMC) sampling to estimate the correlation coefficient between them. They then compute p-values followed by rigorous multiple testing correction (BH based FDR correction).
*A notable strength of their approach lies in the integration of mass spectrometry proteomics data alongside transcriptomic and perturbation screening data, enhancing the robustness of their findings.
*Their work highlights some key insights:
- Gene expression-driven dependency (GED) candidates identified across lineages demonstrate enrichment for "DNA-binding transcription activator activity" and "DNA-binding transcription activator activity, RNA polymerase II-specific" pathways.
- The analysis successfully identifies compelling candidates with robust signals in both GED and PED (FERMT2, GRHL2, KLF5, CDK6, and CCND1), which are well-supported by existing drug evidence or established literature
- Clustering analyses reveal that cancer cells from pancreas and biliary tract tissues, as well as kidney and urinary tract tissue lineages, exhibit remarkably similar expression-driven dependency profiles. Additionally, lineage-specific genes such as transcription factors, cluster together in a manner consistent with existing literature
- Through Fisher's exact test, the authors demonstrate significant enrichments of druggable gene lists from DrugBank with expression-driven dependency patterns at both proteomic and transcriptomic levels
- Experimental validation shows that PAX5 is essential for PAX5-high B cell lymphoma cell growth, while TP63 and GRHL2 are essential for LSCC cell growth.

However, I have several principal concerns about the study that should be addressed to demonstrate the robust and superior performance of this proposed approach.

Authors: We thank the reviewer for their thorough summary and recognition of BEACON’s strengths. To address the principal concerns, we expanded our benchmarking analyses to systematically compare BEACON with Pearson and Spearman correlations in real data to identify druggable genes, clarified the validation experiments for TP63 and GRHL2, and refined methodological descriptions. These

Powered by Editorial Manager® and ProduXion Manager® from Aries Systems Corporation

revisions reinforce that BEACON provides robust and practical improvements over existing approaches for uncovering expression-driven dependencies.

Major Comments:

1.Quantitative benchmarking: While the authors present a valuable contribution, the concept of correlating gene dependency scores to expression has been explored previously through approaches like Project DRIVE (E. Robert McDonald, III et al.) and APSIC (Montazeri et al.). BEACON demonstrates strong correlations across multiple lineages, representing broader scope compared to existing methods that appear more lineage-restricted. However, establishing BEACON's comparative advantages requires more rigorous evaluation. Notably, Project DRIVE—a foundational paper in this field—already identified several BEACON candidates in their "Expression Correlation Analysis Identifies Oncogenes and Lineage-Specific Transcription Factors" section, while APSIC characterized many lineage-specific discoveries as tumor effector genes. BEACON's strength lies in integrating proteomic data with transcriptomic and perturbation screens, enabling identification of additional candidates like PAX5 for hematopoietic and lymphoid tissue. To demonstrate the method's impact, I recommend systematic quantitative benchmarking against existing approaches.
Authors: We systematically compared BEACON GED/PEDs with those obtained from other approaches, including Pearson correlation (as used in Project DRIVE and BACON), and Spearman correlation (as additionally implemented in BACON). APSiC was build to identify new drivers via analyzing perturbation (shRNA) vs. mutation/CNVs, quoting https://academic.oup.com/nar/article/49/15/8488/6329117 "We considered the knockdown experiments of p genes across N cell lines. Let be viability of cell line upon knocking down gene and be a binary variable indicating whether a specific genetic alteration (i.e. mutation or copy number alteration) is present in gene j of cell line i." Thus we did not benchmark against it.

Importantly, BEACON utilizes richer/complementary datasets than previous studies. Disentangling contributions of data richness versus methodological innovation would provide valuable insights into whether enhanced performance stems from improved data availability or genuine method improvements.
Authors: To address this, we applied all methods (Pearson, Spearman, and BEACON) to the same dataset consisting of matched mRNA/protein expression and CRISPR dependency data from DepMap. By holding the data constant, differences in performance directly reflect methodological advantages of BEACON rather than data availability.

Overall for benchmarking, the authors are strongly encouraged to utilize any comprehensive datasets that best demonstrate their method's competitive advantage and are not limited to the specific comparisons recommended above.
Authors: We used the DGIdb druggable gene set (n = 2,993) as a comprehensive and lineage-spanning reference standard. This enabled systematic benchmarking across all available lineages at both the mRNA and protein levels, ensuring that BEACON's advantages are broadly generalizable and not restricted to a subset of examples (Figure S3). Combining all these points, we have added the following to Results:
To further validate BEACON on real data, we systematically benchmarked its performance against Pearson and Spearman correlations to identify a curated set of 2,993 druggable genes from DGIdb as the reference standard using this DepMap CRISPR dataset. andard. For each cancer lineage, we calculated the area under the precision–recall curve (AUPRC) for identifying DGIdb genes based on expression–dependency correlation scores. On average across all lineages, BEACON achieved an AUPRC improvement of 25/29% over Pearson and 23/29% over Spearman correlations (Figure S3), based on CRISPR vs. mRNA/protein expression data. Specifically, BEACON was the top-performing method in 17 of 24 lineages for GED (mRNA) and in 10 of 17 lineages for PED (protein) (Figure S3). The advantage was particularly pronounced in lineages with smaller sample sizes (e.g., Cervix, Oesophagus, Stomach, Endometrium, etc.), where AUPRC gains reached more than two-fold over Pearson/Spearman. An additional benchmarking against the 57 prioritized genes identified by Project DRIVE's expression–dependency model (Pearson-based)29 showed that BEACON achieved average AUPRC gains of 94/530% over Pearson and 123/616% over Spearman correlation (Figure S3). BEACON was also the top-performing method in 18 of 24 lineages for GED (mRNA)

and in 11 of 17 lineages for PED (protein). These results demonstrate that BEACON improves over simpler correlation measures and enhances the recovery of biologically validated dependencies.

2.Correlation method comparisons: Figure S2 shows that BEACON exhibits higher MSE at extremes, and the claimed advantage over Pearson for small sample sizes is difficult to quantify from the current visualization. While the theoretical expectation that BEACON should outperform Pearson in small samples is reasonable, the practical significance remains unclear from these simulations. I recommend demonstrating BEACON's advantage using real data by creating a curated list of established GEDs/PEDs and comparing performance between the two methods. This is particularly important since several of BEACON's hits were previously reported by Project DRIVE using simple Pearson correlations.

Authors: As seen in the prior response, we curated two complementary reference sets: (i) DGIdb druggable genes and (ii) the transcription factors previously identified in Project DRIVE. Using AUPRC as the evaluation metric, BEACON consistently outperformed Pearson and Spearman correlations across both benchmarks, at both the GED (mRNA) and PED (protein) levels (Figure S3). The curation of Project DRIVE's gene list was added in addition to DGIdb druggable gene list to resolve this reviewer's concern.

Alternatively, if BEACON's advantage is indeed significant, please elaborate on the simulation results to better justify this claim with clearer quantitative metrics.

Authors: We also expanded our simulation analysis to include additional scenarios with finer resolution at very small sample sizes and under higher noise levels, running 100 replicates for each condition. These extended simulations demonstrate that BEACON consistently yields more accurate and better-calibrated correlation estimates than other methods, with the advantage being most pronounced in small-N settings and at moderate-to-high noise levels (≥50%) (Figure S2).

3.Validation experiments: I'm seeking clarification on the validation experiments for TP63 and GRHL2. These candidates were not sensitive to predicted dependency and the authors say that "pan-lineage targets may represent universal vulnerability and their inhibition may lead to undesired off-target effects on other cells". Are the authors positioning them as weaker candidates to illustrate the superiority of lineage-specific predictions like PAX5? Additionally, why were different experimental approaches used—CRISPR for PAX5 versus shRNA for TP63 and GRHL2? For a method aimed at identifying druggable targets, would drug based experiments be more relevant than knockdown approaches to better demonstrate clinical applicability?

Authors: We thank the reviewer for raising these important points. GRHL2 and TP63 were selected because, while they appeared as pan-lineage GEDs, their strongest protein-level PED associations were observed in lung cancer, which led us to test them in lung squamous carcinoma cell lines. In contrast, PAX5 was chosen as a clear example of a lineage-specific dependency. We have updated the text at the end of Result section given the limited sets of genes we were able to validate:
Thus, proteins showing lineage-specific dependencies may present as suitable precision oncology targets in the subset of tumors overexpressing the target gene and protein. However, given the limited scope of our validation of three targets, a more systematic validation of GED/PED targets will be required to determine the effectiveness of this target prioritization approach.

Regarding methodology, CRISPR-based validation was used for PAX5 while shRNA was used for GRHL2 and TP63 due to the resources available to the two collaborative teams at Vanderbilt and Gunma University who specialized in lymphoid and lung cancers, respectively.

While drug-based experiments would indeed demonstrate greater clinical applicability, our aim here was to provide proof-of-concept validation of BEACON's target predictions, whereas drug-based validation require different experimental design and introduce other complications that may not correspond with how good the target is

(e.g., on-target efficacy and off-target effects of the drugs used).

Minor comments

1. In Figure 4A, the caption refers to the plot as a heatmap, but the visualization appears to be a scatterplot. Please clarify whether the heatmap is missing or modify the caption appropriately. Additionally, I recommend using a different shade of green, as the current color choice makes some gene names difficult to read.

Authors: We thank the reviewer for noting this. Figure 4A is a scatterplot (not a heatmap), and the caption has been corrected. We also adjusted the green color to improve readability of gene names.

2. In Fig S5A, please add a legend for tumor and normal

Authors: We have added a legend to Figure S6A (previously Fig S5A) to clearly distinguish tumor and normal samples.

3. For the TP63 and GRHL2 validation experiments, please include results for all four cell lines. The current manuscript is missing HCC15-shTP63, HCC15-shGRHL2, and HARA-shGRHL2 plots.

Authors: All replicate results are now presented together in a consolidated figure (Figure 6B), including HCC15 and HARA for GRHL2. HCC15 was not used in the TP63 experiments.

4. How many replicates were the experiments performed on? Is it N= 3 for all experiments?

Authors: Each colony formation and proliferation assay was performed with three replicate wells (N=3), as noted in the Methods section and figure legends.

5. Missing some text here - "BEACON offers the unique advantage of utilizing prior distributions that are less susceptible to outliers, especially in multiple lineages where the number of cell lines."

Authors: The sentence has been corrected to read: “BEACON offers the unique advantage of utilizing prior distributions that are less sensitive to outliers, which is particularly beneficial in lineages where the number of available cell lines is small and thus more vulnerable to the influence of outliers.”

Reviewer #3: The authors develop a method for correlating gene and protein expression with cellular dependencies using the resources of DepMap. The innovation appears to be a Bayesian approach to the correlation analysis. They use this approach to identify potential therapeutic targets and evaluate some top candidates using in vitro experiments. The paper is fairly straightforward to follow.

Major comments: 1. Benchmarking - given the non-linear relationships shown in Fig 2, is a comparison with the Pearson method the most appropriate? Would a Spearman's be better?

Authors: We thank the reviewer for this helpful suggestion. In addition to Pearson, we have now benchmarked BEACON directly against Spearman correlation. Using both simulated and real datasets (DGIdb druggable genes and transcription factors identified in Project DRIVE). This is added in Results text:
We benchmarked BEACON’s Bayesian correlation against Pearson correlation, which was used in project DRIVE29, and against both Pearson and Spearman correlation measures, which were employed in BACON30. Simulations were performed on expression and dependency datasets across a range of correlation levels (from -1 to 1, with 0.25 intervals) and sample size (number of cell lines, 5, 7, 10, 20, 30, 60, 100), with different fraction (0.1, 0.3, 0.5, 0.8, 1) of samples corrupted by noise to enable

direct comparison of methodological performance (Figure S2). Based on these simulations, we observed that the Bayesian method is better than Pearson correlation for estimating moderate true correlation (|rho| < 0.75) in small sample size, and preferable in noisy data (noise level ≥ 0.5, i.e., 50% or more of the samples are corrupted by noise to become outliers), regardless of sample size or true correlation level.

To further validate BEACON on real data, we systematically benchmarked its performance against Pearson and Spearman correlations to identify a curated set of 2,993 druggable genes from DGIdb as the reference standard using this DepMap CRISPR dataset. andard. For each cancer lineage, we calculated the area under the precision–recall curve (AUPRC) for identifying DGIdb genes based on expression–dependency correlation scores. On average across all lineages, BEACON achieved an AUPRC improvement of 25/29% over Pearson and 23/29% over Spearman correlations (Figure S3), based on CRISPR vs. mRNA/protein expression data. Specifically, BEACON was the top-performing method in 17 of 24 lineages for GED (mRNA) and in 10 of 17 lineages for PED (protein) (Figure S3). The advantage was particularly pronounced in lineages with smaller sample sizes (e.g., Cervix, Oesophagus, Stomach, Endometrium, etc.), where AUPRC gains reached more than two-fold over Pearson/Spearman. An additional benchmarking against the 57 prioritized genes identified by Project DRIVE's expression–dependency model (Pearson-based)29 showed that BEACON achieved average AUPRC gains of 94/530% over Pearson and 123/616% over Spearman correlation (Figure S3). BEACON was also the top-performing method in 18 of 24 lineages for GED (mRNA) and in 11 of 17 lineages for PED (protein). These results demonstrate that BEACON improves over simpler correlation measures and enhances the recovery of biologically validated dependencies.

2. The analysis identifies dependencies that are proposed as therapeutic targets, however while the proteins can be druggable, what about normal tissue effects? Some of these are likely lineage-defining proteins that could be highly expressed in normal tissues. Is is notable that in Fig 5B, C that the existing drug targets have a lower association strength than other GEDs identified. Does this suggest that the strongest correlations might be lineage-crucial genes that are too important for normal tissue function to make good drug targets? This needs further consideration in the discussion. Are there any pathways differences between these groups (known drug targets vs others)? For example you might expect more tissue lineage Tfs in the "other" category, while the approved drug targets perhaps more cell surface receptors.

Authors: We thank the reviewer for bringing up this very interesting point. As our pathway analyses revealed, some of the strongest GED/PED associations are enriched for DNA-binding transcription activator (Figure 2D, 3D) that are transcription factors (TFs). In our knowledge, many disease-associated TFs have not been drugged not due to their potential lineage essentiality in adults, but also due to their lack of natural binding pockets and complex protein-DNA or protein-protein interactions. Thus, how druggable and effective they may serve as drug targets remains less tested than other protein families more amenable to prior small molecule/antibody-based approaches. Meanwhile, the DrugBank list is known to be enriched for enzymes, transporters, receptors (https://www.proteinatlas.org/humanproteome/tissue/druggable). We have added to the end of this section:

However, many of these top candidates may have more extreme GED/PED rho values than currently druggable genes; it remains to be tested whether that is a confounding with the protein classes more amenable to current drug modalities or there may be a more desirable GED/PED window for prioritizing therapeutic targets.

We have also added text to the Discussion (underlined texts are the additions):

Many GED/PED gene targets are lineage-specific transcription factors (TFs); these agree with recent single-cell studies and synthesis that posited the "developmental constraint model of cancer cell states", which cancer cell states correspond to and may be constrained by the landscape of "developmental map"46. Thus, a cancer cell adopting a specific developmental state may require activation of such transcription factors and become genetically dependent. Traditionally, TFs were not easily addressable using small molecule or antibody-based approaches due to their lack of binding pockets and complex intermolecular interactions. While such targets used to be considered undruggable, new drug modalities such as proteolysis-targeting chimera

Powered by Editorial Manager® and ProduXion Manager® from Aries Systems Corporation

(PROTAC) are showing promises43,47-50, particularly in cases where there may be a sufficient therapeutic window in inhibiting these TFs, e.g., to treat adult tumors where the target TFs were only essential in early development and in tumor cells.
3. The cell assays performed should effectively be replicating the results of the dependencies on which BEACON is based (DepMap), so why do you get different results? Is it because of the different methods used ie shRNA (not seeing the correlation between expression and dependency) vs CRISPR (replicating the correlation)? If you look at older DepMap scores when they used knockdown rather than CRISPR can you replicate your results?
Authors: The discrepancies between our validation assays and DepMap dependency scores reflect both methodological differences and phenotypic readouts. DepMap scores are derived from pooled CRISPR or shRNA screens after many cell passages and barcode sequencing from pooled cell populations, where cell viability is inferred indirectly through barcode representation after cross-gene normalization. Thus, analyses of Broad and Sanger's large-scale CRISPR knockout screens show correlation but indeed variations of the derived gene score (PMID: 31862961). By contrast, our colony formation assays in lung cancer cells capture proliferative potential, integrating effects on cell survival, division, and stress responses. Similarly, our live cell counts in PAX5 B-cell experiments reflect more acute changes in growth kinetics, which may yield different sensitivity profiles. In our focused experiments, we were also able to conduct qPCR and western blots to validate knockdown/knockout efficiency. Thus, while the broad correlation with DepMap is informative, focused assays provide complementary, context-specific insights into how genetic perturbations affect distinct cellular outcomes.
We have added briefly to the Results, "These focused experiments provide complementary validation to the functional data from the DepMap screen because, in our experiments, all functional readouts were collected within four days of the perturbation (Methods), whereas the DepMap CRISPR screen are performed at 14-21 days and scores are inferred indirectly through barcode representation after cross-gene normalization."
We also thank the reviewer for the suggestion to examine DepMap's RNAi data, albeit this screen had a documentation of even further passaging times. In general, DepMap's RNAi dependency & CRISPR dependencies for these assayed genes agree well in these tested cell lines. Based on this result, we added, "We further queried DepMap RNAi data (passaged for 16 doublings, up to 40 days)5 for these genes that showed consistent dependencies with the original DepMap-based predictions (Figure S7), suggesting potential variations due to shRNA constructs or experimental durations."

4. Although mycoplasma testing was done, were the cell lines re-authenticated by STR profiling at any point?

Authors: All cell lines used in this study were obtained from authenticated biobanks (as listed in Table S15), and mycoplasma testing was performed regularly. The Gunman team member (Dr. Reika) who provided the cell lines for lung cancer experiments had left for industry a few years ago and we did not receive a confirmation on this, and thus we did not change texts there.
The Vanderbilt team confirmed all lines used for the PAX5 experiments were confirmed with STR profiling and we have added accordingly to that section of the Methods.

5. QPCR is mentioned n the methods but not provided in the results that I can find. Did this validate gene knockdown by shRNA? Any correlation between % KD and proliferation/colony forming effect?

Authors: qPCR was performed to confirm knockdown of TP63 and GRHL2 following shRNA treatment in the HARA cell lines. We also recognize the variability in shRNA efficacy and for transparency have added this to results:
To confirm inhibition of the target genes, qPCR validation in HARA cells showed that knockdown efficiency in the HARA cell line where the expression levels of TP63 and GRHL2 were reduced to 33/17% and 41/68%, respectively.

In HARA, the more efficacious sh-TP63-2 showed trends of higher reduction in colony formation than sh-TP63-1, but vice versa for the GRHL2 shRNAs. In all cases the

Powered by Editorial Manager® and ProduXion Manager® from Aries Systems Corporation

knockdowns were significantly effective in reducing colony formation. We did not describe the potential correlation between % KD and proliferation/colony forming effect as qPCR was only conducted in HARA but not other cell lines.

6. In the discussion it should be acknowledged that cancer subtypes exist within lineages that are molecularly and clinically distinct and so the method might be missing targets specific for these eg ER+ and ER- breast cancer.

Authors: We agree with the reviewer that molecular and clinical subtypes within a lineage (e.g., ER^+ vs. ER^- breast cancer) may harbor distinct dependencies that could be missed when analyzing at the lineage level. We have added a note in the Discussion:
It is also important to note that within a given lineage, molecular and clinical subtypes (e.g., ER^+ vs. ER^- breast cancer) may harbor distinct dependencies that could be masked when analyzing at the lineage level. Applying BEACON to subtype-stratified datasets may therefore reveal additional, clinically relevant vulnerabilities. As larger and better-annotated datasets become available, this represents an important direction for future work.

Minor comments:
1. Results para 1 "especially in multiple lineages where the number of cell lines." Missing something in this sentence?

Authors: The sentence has been corrected to read: "BEACON offers the unique advantage of utilizing prior distributions that are less sensitive to outliers, which is particularly beneficial in lineages where the number of available cell lines is small and thus more vulnerable to the influence of outliers."

2. Needs some grammar review

Authors: We have carefully reviewed the manuscript for grammar and style and have corrected the relevant sentences for clarity and readability throughout.

3, Please italicise all gene names (when referring to gene, not protein) eg CCNE1 amplification etc
Authors: All gene symbols have now been italicized throughout the manuscript when referring to genes (e.g., CCNE1-amplified), in accordance with standard nomenclature conventions.
4. Fig S5A - legend or axis labels for N and T needed.

Authors: The axis labels "Tumor" and "Normal" has now been added to clearly distinguish those samples in the figure. With the inclusion of an additional panel, this figure is now presented as Figure S6A in the revised manuscript.

5. Fig S5C, D - these are proliferation not colony forming assays as stated in the text.

Authors: The text has been revised to accurately describe Figures S6C and S6D as proliferation assays rather than colony-forming assays. The Results section now states: "In KNS-62 and H1703 LSCC cells, the knockdown of TP63 using two shRNA constructs (sh-TP63-1 and sh-TP63-2) resulted in a significant reduction in colony formation and cell viability (reduced proliferation) compared to controls($p<0.01$) (Figure 6A, Figure S6D)."

6. Please include number of replicates and type of error bars in figure legends for cell assays

Authors: The figure legends for all cell-based assays have been updated to include the number of replicates (N = 3) and the type of error bars (standard deviation).

Reviewer #4: Reproducibility report

1. Summary of the Study The authors developed a Bayesian method called BEACON

	to integrate multi-omics data. The method was tested on cancer cell lines across 17 tissue types to identify expression- driven dependencies. The method recovered known drug targets and identified novel candidates. The study concludes this method provides a systematic approach to identify precision oncology targets.<br><br>2. Scope of reproducibility According to our assessment the primary objective is: to identify expression-driven dependencies across cancer cell lines from multiple lineages enabling the discovery of genes whose expression levels correlate with cancer cell dependency scores. –<br>Outcome: Identification of genes with significant expression-driven dependencies across pan-lineage cancer cell lines.<br>- Analysis method outcome: "BEACON calculated the Bayesian correlation between the gene's expressions and CERES cancer dependency scores 25 across the pan-lineage cell lines. BEACON modeled expression levels and dependency scores as the bivariate Gaussians and used Markov Chain Monte Carlo (MCMC) sampling to estimate the correlation coefficient rho between them. Given the null hypothesis that the uncorrelated expression and dependency of a gene has the 0 rho coefficient, we statistically tested each gene's rho estimate obtained from the MCMC simulation as follows. Assume that the MCMC sampling is carried out for a null gene's expression and dependency, then we expect that the distribution of the rho estimate accumulated over the MCMC iterations will be centered at zero. Based on this rationale, we computed the z-score of i-th gene as the deviation of the MCMC estimate of rho from the expected (null) value (i.e., zero) in terms of the standard deviation observed in the simulated distribution, i.e., z(i) = rhoMCMC(i) / SDMCMC(i). Since the z-values, by nature, follow a normal distribution with zero-mean and unit-variance, then we computed the p- value for each gene's rho estimate as the probability of observing a value as extreme as the computed z-value for that gene. We multi-testing corrected the resulting p-values using the BH procedure for FDR." (page 19 -Methods section / mRNA expression-driven dependency (GED))<br>- Main result: "We first analyzed the pan-lineage GED by using mRNA levels and the corresponding dependency scores from 854 cell lines with available data across 17 lineages and identified 244 genes showing significant association (correlation coefficient, rho < -0.25, FDR < 0.05)" (page 7 - Results section / Cancer vulnerability targets showing gene expression-driven dependency (GED))<br><br>3. Availability of Materials<br>a. Data<br>- Data availability: Open<br>- Data completeness: Complete, all data necessary to reproduce main results are available.<br>- Access Method: Repository<br>- Repository: https://urldefense.proofpoint.com/v2/url?u=https-3A__doi.org_10.6084_m9.figshare.19700056.v2&d=DwIBaQ&c=shNJtf5dKgNcPZ6Yh64b-ALLUrcfR-4CCQkZVKC8w3o&r=88-dBITsh8vXfnQjNN0pRGpahxI_Sccu4B-wNY_gsU4&m=QnwsYAvlqsJ9rZbiozGbBdLREQ59Pq0O2jitd0FjFA1KqZuHTZDAkiy8-tAbmKGL&s=SNkoITd2TWtWYm6mVW11wfiv2qKBRp4piBeZy4Er5Wo&e=<br>-Data quality: Structured<br><br>b. Code<br>- Code availability: Open<br>- Programming Language(s): R<br>- Repository link: https://urldefense.proofpoint.com/v2/url?u=https-3A__github.com_Huang-2Dlab_BEACON&d=DwIBaQ&c=shNJtf5dKgNcPZ6Yh64b-ALLUrcfR-4CCQkZVKC8w3o&r=88-dBITsh8vXfnQjNN0pRGpahxI_Sccu4B-wNY_gsU4&m=QnwsYAvlqsJ9rZbiozGbBdLREQ59Pq0O2jitd0FjFA1KqZuHTZDAkiy8-tAbmKGL&s=ly6kaHNqm3UtC0ImX6NAlr4fBkJv4CgaN3uAfeBNmIE&e= - License: MIT license<br>- Repository status: Public<br>- Documentation: Readme file<br><br>4. Computational environment of reproduction analysis<br>- Operating system for reproduction: MacOS 15.5<br>- Programming Language(s): R<br>- Code implementation approach: Using shared code

- Version environment for reproduction: R version 4.5.0/RStudio 2025.05.1

5. Results
5.1 Original study results
- Results 1: Supplementary table S2 5.2 Steps for reproduction
-> Run the code PanLineageMCMC.R
- Issue 1: File import paths and incorrect file name
-- Resolved: In the original code, there were fixed file paths that only worked on one specific computer. This caused problems when running the code on other computers. To fix this, I recommended to use relative paths, which are based on where the script is located. This way, the code can be run on any computer without needing to change the paths each time.

------------------ Start of script ------------------
```
sam.dep = read.csv(file.path(getwd(), "DepMap_data",
"sample_info.csv"))
```
------------------- End of script -------------------

- Issue 2: Missing function "intsect" at line 162
-- Resolved: The script called a function intsect that was not defined, leading to an error. Upon request, the authors provided the missing function and added it to the main script (PanLineageMCMC.R).

- Issue 3: Output directory not created.
-- Resolved: The script attempted to write output files to a directory that was not created beforehand. This caused errors during the loop execution when trying to save results. A directory check and automatic creation script was added. If the output folder does not exist, it is now created automatically before the loop runs.

------------------ Start of script ------------------
```
dir_path <-
paste0('../out/jags.nadapt',n.adapt,'.update',n.update,'.mcmc
',n.iter,'.simulation_SD_22Q2')
if (!dir.exists(dir_path)) {
  dir.create(dir_path, recursive = TRUE)
}
```
------------------- End of script -------------------

5.3 Statistical comparison Original vs Reproduced results
- Results: Table.mRNA.dependency.Bayesian.pancancer file attached
- Comments: The Bayesian PanCancer analysis was re-run, but only on the 244 significant genes listed in Supplementary Table S2, not on the full set of 17 285 genes. This choice was made due to limited computational resources, as running the full model would have required an estimated 100 hours.
- Errors detected: -
- Statistical Consistency: Among the 244 significant genes originally reported, the reproduced analysis confirmed the statistical significance of these same genes. However, the exact numerical values (Mean, standard deviation, Z value, P-value and adjusted P-value) differed slightly. These discrepancies are expected due to the nature of Bayesian inference, the absence of a random seed, and the relatively low number of MCMC iterations used (n.iter = 500). These settings may not be sufficient to ensure full convergence or reproducibility of posterior estimates and should be interpreted with caution.
We were unable to compare the rho values because they were not available in the provided Supplementary table S2, nor extracted in the R code to be include in the resulting output files.

6. Conclusion
- Summary of the computational reproducibility review
The results of the Supplementary table S2 in the original study was partially reproduced. We were able to confirm the statistical significance of the 244 genes reported in Supplementary Table S2 using the Bayesian PanCancer model in the provided code. However, the numerical results were not always identical. This is expected because Bayesian methods involve random sampling, the original code did

Powered by Editorial Manager® and ProduXion Manager® from Aries Systems Corporation

not set a fixed random seed, and the number of iterations used was relatively low. Furthermore, the rho values were not available for comparison, limiting a full reproducibility assessment.
Several technical issues were also fixed during the reproduction process, such as hardcoded file paths, a missing function, and the absence of output directories, which were resolved to allow the code to run correctly on a different system. Due to computational limitations, running the full model on all 17,285 genes was not performed.

- Recommendations for authors
While the original analysis code was successfully used to confirm the statistical significance of the 244 genes, we recommend several improvements to enhance reproducibility:
-- Code annotation: Adding more detailed comments within the scripts would help users understand the logic behind each step and the purpose of specific commands or operations.
-- Set a random seed: Include set.seed() in all scripts to improve reproducibility across different runs.
-- Specify R and package versions: Provide the R version and exact package versions needed to run the code, via a requirements file for example.
-- Use relative file paths: Ensure that all necessary folders and functions are created or included by default to avoid path issues.
-- Increase MCMC robustness: Use a higher number of iterations and appropriate parameter settings to ensure better convergence and stability of posterior estimates.
-- Inform users about computation time: Clearly indicate in the README or publication the expected runtime of the code, especially if it requires several hours or days to complete.
-- Please also take a moment to check our website at https://www.editorialmanager.com/giga/l.asp?i=208092&l=RU6ZQV4L for any additional comments that were saved as attachments. Please note that as GigaScience has a policy of open peer review, you will be able to see the names of the reviewers.

Authors: We thank the reviewer for carefully evaluating the reproducibility of our work and for identifying areas where our code and documentation could be improved. We are pleased that the reviewer was able to reproduce the statistical significance of the 244 genes reported in Supplementary Table S2 using the provided Bayesian pan-cancer model. We also acknowledge the helpful feedback regarding technical issues, documentation, and computational reproducibility, which we have addressed in the revised submission.

Specifically, we have updated the scripts to use relative file paths instead of hardcoded directories, included the previously missing “intsect” function, and added automatic directory creation so that outputs can be written without error on any system. We also added more detailed comments within the scripts and clarified expected runtime requirements for users, as suggested. Please see the GitHub commits #31494a7 and #3f6e41a at https://github.com/Huang-lab/BEACON/commits/main/.

Regarding the reviewer’s note that rho values were missing, we clarify that the rho estimates were in fact provided under the column labeled “Mean”, which corresponds to the posterior mean correlation coefficient from the MCMC sampling. To avoid ambiguity, we have updated the supplementary tables and documentation to explicitly state that this column represents rho. We also modified the code so that this output is now labeled “rho” rather than “Mean” for clarity.

In addition, we incorporated the reviewer’s suggestions to improve reproducibility by explicitly setting a random seed in all scripts, and by providing the R version and package versions used in a requirements file.

We agree with the reviewer that numerical differences across runs can occur due to the stochastic nature of Bayesian inference, especially with limited iterations. With the above changes, including higher iterations and fixed random seeds, we expect future runs to be more stable and reproducible. Finally, as added to the code availability

	section, this tool has been registered at bio.tools under the identifier biotoolsID: BEACON-x, and at SciCrunch.org under the identifier RRID: SCR_027484.<br><br>Overall, we are grateful for these constructive suggestions, which have helped us improve the clarity, usability, and reproducibility of both our code and manuscript.
Additional Information:	
Question	Response
Are you submitting this manuscript to a special series or article collection?	No
**Experimental design and statistics**<br><br>Full details of the experimental design and statistical methods used should be given in the Methods section, as detailed in our Minimum Standards Reporting Checklist. Information essential to interpreting the data presented should be made available in the figure legends.<br><br>Have you included all the information requested in your manuscript?	Yes
**Resources**<br><br>A description of all resources used, including antibodies, cell lines, animals and software tools, with enough information to allow them to be uniquely identified, should be included in the Methods section. Authors are strongly encouraged to cite Research Resource Identifiers (RRIDs) for antibodies, model organisms and tools, where possible.<br><br>Have you included the information requested as detailed in our Minimum Standards Reporting Checklist?	Yes
**Availability of data and materials**<br><br>All datasets and code on which the conclusions of the paper rely must be either included in your submission or deposited in publicly available repositories (where available and ethically appropriate), referencing such data using	Yes

a unique identifier in the references and in the “Availability of Data and Materials” section of your manuscript.<br><br>Have you have met the above requirement as detailed in our [Minimum Standards Reporting Checklist]?	
GigaScience has policies and guidelines in place for the use of generative AI-writing tools such as ChatGPT. If you have used such writing tools to assist with writing the manuscript this must be declared and cited in the text. Authors should not list AI-writing tools and other AI-assisted technologies as an author or co-author and should acknowledge that they are fully responsible for text generated or refined by AI-writing tools.<p><br><br>A summary of use (particularly in the introduction or among methods) needs to be included at the end of the paper, and the outputs should also be included as a supplementary file hosted in GigaDB or other open repositories. Please read our guidelines for more information. <p><br><br>By submitting to GigaScience, you are aware of the journal's AI-writing tools policy, and if you have declared use of such tools below, you have acknowledged this where appropriate in your manuscript and have made a summary of use and outputs available. <p><br><b>AI-assisted writing tools have been used in the preparation of this manuscript?	Yes

Powered by Editorial Manager® and ProduXion Manager® from Aries Systems Corporation

Manuscript
Click here to access/download;Manuscript;Expression_Dependency_Manuscr

Expression-Driven Genetic Dependency Reveals Targets for Precision Oncology

Abdulkadir Elmas[1], Hillary M. Layden[2], Jacob D. Ellis[2], Luke N. Bartlett[2], Xian Zhao[3], Reika Kawabata-Iwakawa[4], Hideru Obinata[5], Scott W. Hiebert[2,6], Kuan-lin Huang[1]*

[1] Department of Genetics and Genomic Sciences, Department of Artificial Intelligence and Human Health, Center for Transformative Disease Modeling, Tisch Cancer Institute, Icahn Genomics Institute, Icahn School of Medicine at Mount Sinai, New York, NY 10029, USA.
[2] Department of Biochemistry, Vanderbilt University School of Medicine, Nashville, Tennessee 37232, USA
[3] Department of Biochemistry, Gunma University Graduate School of Medicine, Maebashi, Gunma 371-8511, Japan. Current affiliation: Department of Pharmacy, Nanjing Drum Tower Hospital, Affiliated Hospital of Medical School, Nanjing University, 321 Zhongshan Road, Nanjing, Jiangsu, 210008, China.
[4] Division of Integrated Oncology Research, Gunma University Initiative for Advanced Research, Gunma University, Maebashi, Gunma 371-8511, Japan
[5] Education and Research Support Center, Gunma University Graduate School of Medicine, Maebashi, Gunma 371-8511, Japan
[6] Vanderbilt-Ingram Cancer Center, Nashville, Tennessee 37027, USA

*Corresponding Author:
Kuan-lin Huang, Ph.D.
Departments of Genetics and Genomic Sciences & Artificial Intelligence and Human Health
Icahn School of Medicine at Mount Sinai
New York, NY 10029
Email: kuan-lin.huang@mssm.edu

Abstract

Background: Cancer cells are heterogeneous, each harboring distinct molecular aberrations and are dependent on different genes for their survival and proliferation. While targeted therapies based on driver DNA mutations have shown success, many tumors lack druggable mutations, limiting treatment options. We hypothesize that new precision oncology targets may be identified through "expression-driven dependency," where cancer cells with high expression of specific genes are more vulnerable to the knockout of those same genes.

Results: We developed BEACON, a Bayesian approach to identify expression-driven dependency targets by analyzing global transcriptomic and proteomic profiles alongside genetic dependency data from cancer cell lines across 17 tissue lineages. BEACON successfully identified known druggable genes including *BCL2, ERBB2, EGFR, ESR1,* and *MYC*, while revealing novel targets confirmed by both mRNA and protein-expression driven dependency. The identified genes showed a 3.8-fold enrichment for approved drug targets and a 7 to 10-fold enrichment for druggable oncology targets. Experimental validation demonstrated that depletion of *GRHL2, TP63*, and *PAX5* reduced tumor cell growth and survival in their dependent cells.

Conclusions: We provide a systematic approach to identify precision oncology targets based on expression-driven dependency patterns. By integrating multi-omics data with genetic dependency screens, BEACON generated a comprehensive catalog of potential therapeutic targets that may expand treatment options for cancer patients lacking druggable mutations. This resource offers new opportunities for precision oncology target discovery beyond mutation-based approaches.

Keywords Precision oncology, expression-driven dependency, cancer vulnerability, BEACON, Bayesian statistics, proteomics, transcriptomics, cancer cell lines, drug targets, multi-omics

Introduction

Precision oncology requires accurate identification of molecular aberrations in cancer cells that can serve as biomarkers and therapeutic targets. While some tumors harbor genomic mutations predictive of cancer vulnerability, a large fraction of cancer cells lack such actionable mutations[1-3]. Large-scale genetic dependency screens, including the Cancer Cell Line Encyclopedia (CCLE)[4], Cancer Dependency Map (DepMap)[5] and CancerGD[6], have revealed that cancer cells show different vulnerability upon genetic knockdown or knockout. Across diverse types of molecular alterations—including mutations, copy number alterations and expression—gene expression biomarkers have been identified as the top biomarkers of genetic dependency, e.g., in 82% of the 501 DepMap cell lines in a genome-scale RNAi screen[5]. We thus reasoned that precision oncology targets might be identified through “expression-driven dependency”, whereby cancer cells with high expression of the targeted genes are more vulnerable to genetic depletion or therapeutic inhibition.

Multiple studies have used genetic and functional screening data to identify cancer vulnerabilities present in a subset of cancer cells, including aneuploid cancer cells[7,8], pediatric tumor cells[9], and multiple myeloma cells[10]. Notable targets identified include the *WRN* helicase that is essential in cancers with microsatellite instability (MSI)[11,12], PKMYT1 kinase in *CCNE1*-amplified tumors, and *BCAR1* in *KRAS* mutant pancreatic cancer, where the suppression of *BCAR1* and *TUBB3* sensitizes cancer cells to *ERK* inhibition by reducing MYC protein levels[13]. Bondeson et al.[14] identified phosphate dysregulation as a therapeutic vulnerability in ovarian cancer through genome-scale CRISPR-Cas9 screens, highlighting the XPR1–KIDINS220 protein complex as crucial for cancer cell survival. Another study[8] identified the ubiquitin ligase complex UBA6/BIRC6/KCMF1/UBR4 as crucial for the survival of aneuploid epithelial tumors. These studies highlight the potential of developing a systematic approach to identify drug targets by linking subsets of cancer cells to genetic dependency based on their aberrant expression.

Expression analyses focusing on only the transcriptome assume that high gene mRNA expression translates into high protein abundance. However, gene expressions show only

moderate correlations with protein expression in cancer cell lines and primary tumors[15-20], and protein-level analyses may identify new targets[3,21-23]. Notably, global proteomic profiles of 375 cell lines in the CCLE/DepMap were recently generated by global mass spectrometry (MS), quantifying a total of 12,399 proteins using multiplexing quantification methods[24]. The combination of these datasets provides unprecedented opportunities to identify new protein biomarkers and therapeutic targets across cancer types.

Herein, we integrated global proteomic and transcriptomic profiles of 855 cancer cell lines across 17 tissue types from Cancer Dependency Map (DepMap)/Cancer Cell Lines Encyclopedia (CCLE)[24,25], and the corresponding cancer cell dependency scores (Achilles) based on the CRISPR knockout screens[25-27]. By developing a new Bayesian correlation approach, BEACON, we identified the expression-driven cancer cell dependencies (ED) for each tissue type at different molecular layers, and revealed new potentially actionable targets that are strongly-associated with druggable gene lists[28] (**Figure 1**). Our analyses identified the known drug targets *SOX10* and *ESR1* demonstrating strong gene/protein ED linked to their specified cancer type and revealed new potential candidate targets for each cancer type. Experimental validation supported the actionability of the new candidate targets *TP63, GRHL2*, and *PAX5*, exposing potential vulnerability in their dependent cancer cells.

Results

To identify genes showing expression-driven dependency, we first integrated RNA-seq data, global mass spectrometry proteomics data, and the cell dependency data corresponding to the same cell lines in the DepMap project (**Methods**). We restricted our analyses to lineages where at least 7 cell lines with cancer cell line dependency and corresponding mRNA/protein expression data were available to ensure statistical robustness (**Figure S1A**). Overall, 855 cell lines across the 17 lineages shared cancer cell dependency scores and corresponding mRNA and protein expressions (N=854 for mRNA, N=290 for protein, **Figure S1B, Table S1**). Based on this limited sample size per cell lineage (**Figure S1C)**, we noticed that the basic correlation techniques may lead to spurious correlations, particularly for protein expression (**Figure S1B, Figure S2**). Thus,

we developed a Bayesian approach, BEACON (Bayesian EvAluation of expression Correlation-driveN dependency), to model expression levels and dependency scores as the bivariate Gaussians and used Markov Chain Monte Carlo (MCMC) sampling to test the null hypothesis that these two are uncorrelated for each given gene (**Methods**). BEACON offers the unique advantage of utilizing prior distributions that are less sensitive to outliers, which is particularly beneficial in lineages where the number of available cell lines is small and thus more vulnerable to the influence of outliers. We benchmarked BEACON's Bayesian correlation against Pearson correlation, which was used in project DRIVE[29], and against both Pearson and Spearman correlation measures, which were employed in BACON[30]. Simulations were performed on expression and dependency datasets across a range of correlation levels (from -1 to 1, with 0.25 intervals) and sample size (number of cell lines, 5, 7, 10, 20, 30, 60, 100), with different fraction (0.1, 0.3, 0.5, 0.8, 1) of samples corrupted by noise to enable direct comparison of methodological performance (**Figure S2**). Based on these simulations, we observed that the Bayesian method is better than Pearson or Spearman correlation for estimating moderate true correlation ($|rho| < 0.75$) in small sample size, and preferable in noisy data (noise level ≥ 0.5, i.e., 50% or more of the samples are corrupted by noise to become outliers), regardless of sample size or true correlation level.

To further validate BEACON on real data, we systematically benchmarked its performance against Pearson and Spearman correlations to identify a curated set of 2,993 druggable genes from DGIdb as the reference standard using this DepMap CRISPR dataset. For each cancer lineage, we calculated the area under the precision–recall curve (AUPRC) for identifying DGIdb genes based on expression–dependency correlation scores. On average across all lineages, BEACON achieved an AUPRC improvement of 25/29% over Pearson and 23/29% over Spearman correlations (**Figure S3**), based on CRISPR vs. mRNA/protein expression data. Specifically, BEACON was the top-performing method in 17 of 24 lineages for GED (mRNA) and in 10 of 17 lineages for PED (protein) (**Figure S3**). The advantage was particularly pronounced in lineages with smaller sample sizes (e.g., Cervix, Oesophagus, Stomach, Endometrium, etc.), where AUPRC gains reached more than two-fold over Pearson/Spearman. An additional benchmarking against the 57 prioritized genes identified by Project DRIVE's expression–

dependency model (Pearson-based)[29] showed that BEACON achieved average AUPRC gains of 94/530% over Pearson and 123/616% over Spearman correlation (**Figure S3**). BEACON was also the top-performing method in 18 of 24 lineages for GED (mRNA) and in 11 of 17 lineages for PED (protein). These results demonstrate that BEACON improves over simpler correlation measures and enhances the recovery of biologically validated dependencies.

Cancer vulnerability targets showing gene expression-driven dependency (GED)

We first applied BEACON to reveal cancer vulnerabilities that show gene expression-driven dependencies (GED) at the mRNA level. We first analyzed the pan-lineage GED by using mRNA levels and the corresponding dependency scores from 854 cell lines with available data across 17 lineages and identified 244 genes showing significant association (correlation coefficient, rho < -0.25, FDR < 0.05). The notable genes with strong pan-lineage associations (false discovery rate, FDR < 1e-32) include *SOX10* (correlation coefficient, rho = -0.83), *IRF4* (rho = -0.82), *HNF1B* (rho = -0.76), and *MYOD1* (rho = -0.70) (**Table S2**).

Having found many GEDs across cancer cells from different tissue types, we then applied BEACON to identify tissue-specific GEDs within each lineage (**Methods**). As expected, several significant pan-lineage GED targets also showed substantial tissue-level GED in multiple lineages, including *TP63, CCND1, CCND2*, and *KLF5* (rho ≤ -0.61, FDR < 1e-32) (**Figure 2A-B**). *TP63* showed significant (rho < -0.25, FDR < 0.05) GED across 14 out of 24 lineages of the cancer cell lines. *TP63* is a member of the p53-family transcription factors that regulates developmental processes in several organs and tissues, as well as tumorigenesis and tumor progression[31]. Another transcription factor, *KLF5* also showed significant (rho < -0.25, FDR < 0.05) GED frequently across half of the cell lineages (12/24). This could be explained by its role in the development and progression of various types of cancer, as its expression is essential for cell cycle regulation, apoptosis, migration, and differentiation, impacting a wide array of target genes such as cyclin D1, cyclin B, PDGFα, and FGF-BP[32].

Since multiple lineages were dependent on the expression of transcription factors such as *KLF5* and *TP63*, targeting these genes may lead to unintended consequences across

tissue types. To minimize potential off-target effects, we further identified the GED targets showing only lineage-specific expression-driven dependency, i.e., exhibiting low correlation (more negative rho) within a given lineage's cell lines and relatively smaller (near-zero) correlation in other lineages (**Methods**, **Figure 2A**). Among such targets, we found *MYOD1* for soft tissue, *PAX5* for haematopoietic and lymphoid tissue, *SOX10* for skin, and *ESR1* for breast (rho ≤ -0.84, FDR < 1e-32) (**Table S2, Figure 2A, Figure 2C**). We next investigated whether the candidate targets showing GED were enriched in distinct molecular pathways. Enrichment analyses using Gene Ontology (GO)[33] for each lineage GED revealed 38 unique pathways enriched across lineages (**Figure 2D**). Although different pathways showed different levels of enrichment, the two GO terms, (i) "DNA-binding transcription activator activity" (GO:0001216) and (ii) "DNA-binding transcription activator activity, RNA polymerase II-specific" (GO:0001228), were the most frequently-enriched across the lineages (15 out of 18 lineages).

To explore the potential clinical actionability of the identified GEDs, we integrated drug-gene interaction database (DGIdb)[34], and identified 82 druggable factors out of 244 pan-lineage GEDs (**Figure S4A, Table S3**). By analyzing dependencies at each tissue, we identified 951 druggable targets showing significant (rho < -0.25, FDR < 0.05) lineage-specific GEDs, including 132 targets for hematopoietic and lymphoid tissue, 101 for lung, 81 for soft tissue, 61 for central nervous system, 52 for ovary, 49 for stomach, 47 for autonomic ganglia, and 44 for breast (**Figure S4B**). Among these, the most strongly-associated (rho ≤ -0.78, FDR < 1e-32) tissue-specific GED targets include *MYOD1* in soft tissue, *ESR1* in breast, *WT1* in ovary, and *SOX10* in skin (**Table S3)**. The skin-specific GED observed for *SOX10* was consistent with a recent study[35], where the mRNA expression of *SOX10* was found to be associated with *SOX10* hypomethylation and sensitivity to *SOX10* knockdown in melanoma cell lines, while other tissues' cell lines showed limited *SOX10* expression and limited dependency to *SOX10* for survival. The strong *ESR1*-driven dependency in breast cancer cell lines support the established use of SERMs and aromatase inhibitors in ER(+) breast cancers[36]. Several GED genes already have established targeted therapies, and additional genes showing strong lineage-specific expression-driven dependencies may also have therapeutic potential.

Based on a set of the most significant GED targets found within lineages (rho < -0.75, FDR < 1e-10), clustering analyses (**Methods**) showed that cancer cells of the pancreas and biliary tract tissue lineages showed the most similar expression-driven dependency profiles, as well as those of the kidney and urinary tract tissue lineages **(Figure S4C**). We also conducted a clustering analysis to identify GED-nominated drug targets showing similar tissue-specificities across tissue lineages. For example, the breast-specific *ESR1* transcription factor is clustered with the other factors *FOXA1*, *SPDEF*, *TBX3*, and *TRPS1* (**Figure S4D**). These transcription factors showed the strongest GED levels in breast tissue cell lines (rho < -0.6, FDR < 2e-5), where *SPDEF* showed breast-specific GEDs similar to *ESR1*. *FOXA1* and *SPDEF* are the key drivers of ER+ breast cancer risk and have been identified as master regulators of the *FGFR2*-mediated cancer risk[37]. These results identified cross-tissue cancer cells that may share similar targets.

Cancer vulnerability targets showing protein expression-driven dependency (PED)

Given that gene mRNA expressions show only moderate correlations with protein abundance in cancer[15-20], we next sought to expand our analyses to identify targets showing protein expression-driven dependency (PED). We applied BEACON to dependency data and protein expression levels in the subset of 290 cell lines with both types of data (**Methods**). BEACON identified 223 proteins showing significant (rho < -0.25, FDR < 0.05) pan-lineage protein expression-driven dependency (PED). Among the proteins showing pan-lineage PED, just over half (N=123) of the targets also showed significant (rho < -0.25, FDR < 0.05) pan-lineage GED, suggesting general concordance between mRNA and protein while implicating the importance of considering protein expression. ZEB2 was the most strongly-associated PED (rho = -0.64), followed by FERMT2, GRHL2, KLF5, CDK6, and CCND1 (rho ≤ -0.52), all of which also showed significant GED (**Table S4**, **Figure 3A-B**). The other 100 PED targets that do not show significant mRNA-level GED included ELMO2, PRDM6, FGFR3, RUNX1, VGLL1, TMEM158, and CBFB (rho ≤ -0.39) (**Table S4**). We identified 78 druggable proteins that show significant (rho < -0.25, FDR < 0.05) pan-lineage expression-driven dependency,

including SOX10, MYB, GATA1, MYOD1, CDK6, HNF4A, CCND1, and PAX5 (rho ≤ -0.52) (**Table S5**).

At the individual tissue level, many of these pan-lineage PED targets also showed high PED within multiple lineages (**Figure 3A**). Targets showing PED exclusive for each lineage (rho ≤ -0.83) included PYURF in soft tissue, GTSF1 in central nervous system, PAX5 in haematopoietic and lymphoid tissue, TTC7B in kidney, and TMEM208 in bone (**Figure 3A, Figure 3C, Table S6**). To examine potential actionability of the identified PED proteins, we integrated DGIdb and identified 170 druggable significant (rho < -0.25, FDR < 0.05) lineage-specific PEDs for all lineages; within these, we found a set of very strong lineage-specific targets (rho ≤ -0.81), including PAX5 in haematopoietic and lymphoid tissue, LAMP1 in stomach, NEK6 in urinary tract, TSPO in kidney, CHKA in oesophagus, SERPIND1 in ovary, and SOX10 in central nervous system (**Figure S4E**, **Table S6)**. Enrichment analyses with the PED targets yielded 17 pathways enriched in a more lineage-specific pattern than GED results (**Figure 3D, Table S7**). DNA-binding transcription activator activity (GO:0001216 and GO:0001228) terms were similarly significantly enriched showing consistency with the GED results (3 out of 5 lineages).

Concordance between gene and protein expression-driven dependency

Protein expression evidence can validate molecular targets observed at the mRNA level. We analyzed the concordant and unique gene targets based on their GED and PED correlations. We found 123 genes showing consistently significant pan-lineage expression-driven dependency in both mRNA and protein levels, most notably *SOX10* (rho_{RNA} = -0.82, $rho_{protein}$ = -0.77), *TP63* (rho_{RNA} = -0.69, $rho_{protein}$ = -0.72), *IRF4* (rho_{RNA} = -0.82, $rho_{protein}$ = -0.73), and *MYB* (rho_{RNA} = -0.66, $rho_{protein}$ = -0.75) (**Figure 4A, Table S8**). The confirmation of both GED and PED demonstrate the robustness of these targets.

Meanwhile, given the moderate correlation between mRNA and protein expression, protein expression-driven dependency may also reveal protein aberrations that arise post-transcriptionally. We found 85 genes showing significant (rho < -0.25, FDR < 0.05) pan-lineage GED without a significant PED that may be less robust as potential

therapeutic targets, including *MYCN* (rho_{RNA} = -0.59, $rho_{protein}$ = -0.05), *OTX2* (rho_{RNA} = -0.56, $rho_{protein}$ = -0.14), and *EBF1* (rho_{RNA} = -0.53, $rho_{protein}$ = -0.22) (**Figure 4A, Table S8**). On the other hand, we also found 100 proteins showing significant (rho < -0.25, FDR < 0.05) pan-lineage PED without a significant GED. Some notable targets include ELMO2 (rho_{RNA} = -0.2, $rho_{protein}$ = -0.47), PRDM6 (rho_{RNA} = -0.02, $rho_{protein}$ = -0.85), FGFR3 (rho_{RNA} = -0.12, $rho_{protein}$ = -0.6), and RUNX1 (rho_{RNA} = -0.24, $rho_{protein}$ = -0.42) (**Figure 4A, Table S4**).

We next analyzed the consistency between tissue-level GEDs and PEDs for each lineage. (**Figure 4B, Table S9)**. In total, we found 121 genes showing significant GED and PED (rho < -0.25, FDR < 0.05) within a lineage, which may present as some of the strongest targets identified through BEACON. *KLF5* showed significant GED and PED (rho < -0.47, FDR < 0.045) in the endometrium, liver, and pancreas lineages. *SOX2* gene showed significant GED and PED (rho < -0.42, FDR < 0.032) in the lung and oesophagus lineages. Other lineage-specific targets showing concordance between GED and PEDs include *FOXA1* in breast, *PAX5* in haematopoietic and lymphoid tissue, *GATA2* in large intestine, *MDM2/TP63* in lung, and *TFAP2A* in skin.

Leveraging expression-driven dependency to enrich for drug targets

Identification of drug targets is a major goal of genomic studies, yet even by using 141,456 human DNA-Seq data in gnomAD without phenotype association, known drug targets only showed a minor difference in constraints for loss-of-function (LoF) variants compared to other genes[28]. To test whether expression-driven dependency derived by BEACON may represent an effective target identification strategy, we ascertained whether the BEACON-identified genes are enriched for druggable targets from DrugBank and gene lists curated by Minikel et al.[28]. We used Fisher's exact test to evaluate the association between the druggable gene lists and the pan-lineage GEDs/PEDs (**Methods**). The majority (8 out of 15) of druggable gene lists from DrugBank were significantly enriched (Fisher's exact test, odds ratio > 2, FDR < 0.05) with expression-driven dependency observed at both mRNA and protein levels (**Figure 5A, Table S10**). Genes targeted by *Antibody* was the gene set most enriched with GEDs and PEDs (OR_{RNA} = 9.2, $OR_{protein}$ =

18.9), where the higher enrichment in PEDs aligns with the mechanism of action of the antibody directly binding to proteins. These GED/PED genes include Antibody targets (5 out of 23) showing significant levels of both GED/PED (rho < -0.25, FDR < 0.05) such as *CD19* (rho_{RNA} = -0.56, $rho_{protein}$ = -0.66), *EGFR* (rho_{RNA} = -0.43, $rho_{protein}$ = -0.36), *ITGB3* (rho_{RNA} = -0.41, $rho_{protein}$ = -0.36), *ERBB2* (rho_{RNA} = -0.41, $rho_{protein}$ = -0.34), and *PDGFRA* (rho_{RNA} = -0.37, $rho_{protein}$ = -0.26) (**Figure 5B-C, Table S11**). These targets also belong to DrugBank's *Approved drug targets* (enrichment OR_{RNA} = 3.9, $OR_{protein}$ = 3.9) and *Oncology (Cancer)* (enrichment OR_{RNA} = 10.1, $OR_{protein}$ = 7.5) gene lists, both of which were also significantly enriched with GEDs and PEDs. The high fold enrichment for druggable genes in the *Oncology* gene set aligns with our analyses using DepMap cancer cell lines. Other well-established targets within *Approved drug targets* and *Oncology* that show strong GED and PED include *BCL2* (for both RNA and protein level EDs, rho < -0.39), *PIK3CD* (rho < -0.33), and *PDGFRB* (rho < -0.29), suggesting that BEACON reliably captures established therapeutic dependencies and provides an effective framework for validating known oncogene addictions. Additionally, among the Drugbank *Oncology* gene set, *PSMB5* (targeted by proteasome inhibitors bortezomib and carfilzomib for hematologic malignancies) and *RXRA* (targeted by bexarotene, an RXR agonist used in the treatment of cutaneous T-cell lymphoma [CTCL]), showing significant levels of GED/PED, were among the Oncology gene list, reinforcing the robustness of our approach in identifying clinically relevant targets.

Notably, in addition to enrichment for known Oncology druggable genes, BEACON-identified GED/PEDs also showed suggestive enrichment for multiple other indication categories, including DrugBank gene sets for Skeletomuscular (OR = 4.9, p = 0.0693 for GEDs; OR = 3.1, p = 0.29 for PEDs) and Metabolic/Alimentary (OR = 2.2, p = 0.24 for GEDs; OR = 4.80, p = 0.030 for PEDs) diseases. The Drugbank Other Indications category with more targets and statistical power showed significant enrichment for both GEDs (OR = 3.8, FDR =0.022) and PEDs (OR = 3.9, FDR =0.022), suggesting there may be a broader utility of these cell-specific targets beyond oncology.

We next characterized whether GED/PEDs identified by BEACON may be more sensitive to identifying genes with specific mode of inheritance or with additional genetic effect

properties[28]. GED/PED genes were both enriched for Autosomal Dominant genes and haploinsufficient genes as determined by ClinGen, but showed no association with Autosomal Recessive genes (**Figure S5**). Moreover, GED/PED targets were underrepresented among the common essential genes (N=684 "Essential In Culture" genes based on 17 genome-wide CRISPR screens[38]), suggesting that BEACON identifies cell-specific vulnerabilities rather than dependencies universally required for cell viability (e.g., house-keeping genes) that could lead to off-target effects. **Table S12** further highlights 36 genes in 10 DrugBank/genetic effect lists that showed significant (rho < -0.25, FDR < 0.05) pan-lineage expression-driven dependency in both mRNA and protein levels.

Additional GED/PED targets identified by BEACON that are not currently druggable targets (DrugBank) include *SOX10* (rho_{RNA} = -0.83, $rho_{protein}$ = -0.77, also belong to *ClinGen Haploinsufficient* and *Autosomal Dominant* gene sets) and *TP63* (rho_{RNA} = -0.69, $rho_{protein}$ = -0.72, *ClinGen Haploinsufficient*) and the *Autosomal Dominant* genes *GRHL2* (rho_{RNA} = -0.6, $rho_{protein}$ = -0.53) and *HNF4A* (rho_{RNA} = -0.5, $rho_{protein}$ = -0.62) (**Figure 5B**, **Figure 5C, Table S11**). For genes not in these DrugBank/gene-effect gene lists[28], BEACON identified 87 targets that showed significant ED (rho < -0.25, FDR < 0.05) at both mRNA and protein levels that may represent potential therapeutic targets for further experimental and clinical development, including *IRF4* (for both RNA and protein levels, rho < -0.73), *MYB* (rho < -0.66), *GATA1* (rho < -0.52), *FERMT2* (rho < -0.53), *KLF5* (rho < -0.54), *CCND1* (rho < -0.54), *MYOD1* (rho < -0.7), *PAX5* (rho < -0.66), and *CCND2* (rho < -0.64) (**Table S13**). For example, *IRF4* knockdown is lethal to multiple myeloma cells[39]. The *IRF4* gene is linked to BET protein-mediated transcriptional program[40] and its dysregulation is also implicated in lymphoid malignancies during hematopoietic cell differentiation[41]. These suggest a therapeutic hypothesis where *IRF4*-expressing melanoma/lymphoid malignant cells may be accessible through BET inhibitors (BETi). However, many of these top candidates may have more extreme GED/PED rho values than currently druggable genes; it remains to be tested whether that is a confounding with the protein classes more amenable to current drug modalities or there may be a more desirable GED/PED window for prioritizing therapeutic targets.

Experimental validation of candidate targets showing express-driven dependency

To experimentally validate GED/PED targets identified by BEACON, we selected two types of targets to be tested across two lineages: (1) two targets showing pan-lineage expression-driven dependency, *GRHL2* and *TP63*, and (2) one target showing lineage-specific expression-driven dependency, *PAX5*. These focused experiments provide complementary validation to the functional data from the DepMap screen because, in our experiments, all functional readouts were collected within four days of the perturbation (**Methods**), whereas the DepMap CRISPR screen are performed at 14-21 days and scores are inferred indirectly through barcode representation after cross-gene normalization.

We first confirmed that *TP63* and *GRHL2* mRNA expression were up-regulated in lung squamous (LSCC) tumor tissue compared to tumor-adjacent normal tissue in TCGA, and chose cultured LSCC cells to conduct validation experiments (**Methods**)(**Figure S6A-B**). To confirm inhibition of the target genes, qPCR validation in HARA cells showed that the shRNAs reduced gene expression levels of *TP63* and *GRHL2* to 33/17% and 41/68%, respectively (**Figure S6C**). Cell proliferation and colony-forming ability were then measured using two types of cells with high dependency (HARA, KNS-62) on candidate genes and cells with low dependency (H1703, HCC15 [only included in GRHL2 experiments]). In KNS-62 and H1703 LSCC cells, the knockdown of *TP63* using two shRNA constructs (sh-TP63-1 and sh-TP63-2) resulted in a significant reduction in colony formation and cell viability (reduced proliferation) compared to controls ($p<0.01$) (**Figure 6A, Figure S6D**). Similarly, *GRHL2* knockdown using sh-GRHL2-1 and sh-GRHL2-2 in both cell lines led to a significant decrease in colony formation and cell viability ($p<0.01$) (**Figure 6B, Figure S6E**). *TP63* knockdown also resulted in reduced colony formation in HARA cell line (**Figure 6A**). The results showed that the knockdown of either gene highly inhibited cell viability and colony formation in LSCC cell lines, regardless of the predicted dependence. We further queried DepMap RNAi data (passaged for 16 doublings, up to 40 days)[5] for these genes that showed consistent dependencies with the original

DepMap-based predictions (**Figure S7**), suggesting potential variations due to shRNA constructs or experimental durations.

The lineage-specific target, *PAX5*, was evaluated for its role in haematopoietic and lymphoid tissue. Within the lineage, groups of cells with high and low *PAX5* expression and low and high *PAX5* genetic dependency can be clearly identified by BEACON (**Figure 6C**). We chose two *PAX5*-low myeloid lineage cell lines (HEL and Kasumi-1) and two *PAX5*-high (REH and SU-DHL4) B-cell lines to conduct *PAX5* knockout (KO) experiments via CRISPR. Upon confirming successful KO via western blots, we showed that PAX5 KO significantly reduced the number of live cells in REH and SU-DHL-4 cell lines compared to controls ($p<0.05$ and $p<0.01$, respectively). But *PAX5* KO did not significantly inhibit cell survival for HEL and Kasumi-1 (**Figure 6D**). Overall, these results show that while TP63 and GRHL2 are essential for cell growth across LSCC cells, PAX5 is specifically crucial for the growth of PAX5-high B cell lymphoma cells. Thus, proteins showing lineage-specific dependencies may present as suitable precision oncology targets in the subset of tumors overexpressing the target gene and protein. However, given the limited scope of our validation of three targets, a more systematic validation of GED/PED targets will be required to determine the effectiveness of this target prioritization approach.

Discussion

This study integrates large-scale CRISPR screen in conjunction with transcriptomic and proteomic data to identify expression-driven dependencies in cancer cells[4,5], providing a potential new category of targets in precision oncology, particularly against cancer cells without druggable mutations (**Figure 1**). Our newly developed Bayesian correlation approach BEACON identified known drug targets and uncovered new candidate genes, demonstrating the utility of expression-driven dependency as a complementary strategy to traditional mutation-driven analyses. Functional experiments demonstrated that targeting genes with high expression levels could reveal potential vulnerabilities within specific cancer types, e.g., PAX5 in lymphoid tumors. We also identified distinct molecular pathways enriched in tissues based on the GED/PEDs, providing insights into the

biological processes underpinning cancer progression (**Figures 2-3**). The concept of expression-driven dependency expands the scope of actionable targets by focusing on genes whose high expression levels selectively contribute to cancer cell survival[7-10]. This is particularly relevant in cases where actionable mutations are absent, thereby addressing a significant gap of treatment options in precision oncology[1-3].

By integrating CRISPR/transcriptomic data from CCLE/DepMap[4,5], and global proteomic analyses[24], we ensured a robust identification of GED/PEDs. GEDs and PEDs show significant correlation ($R = 0.54$, $p < 2e-16$) across the cell lines; thus, analyzing the GED/PED can cross-validate the robustness of candidate vulnerability targets (**Figure 4**). Our Bayesian approach BEACON further enhanced the reliability of our findings by accommodating variability and limited sample sizes within each tissue lineage (**Figure S2**). The identification of GED/PEDs has significant implications for drug development and personalized cancer therapy. By targeting genes with high expression levels, new therapeutic avenues can be explored in tumors currently with limited treatment options[1-3]. Although in this study we emphasized negative associations where higher target expression corresponds to greater dependency, the Bayesian framework is symmetric and can also detect positive correlations, where in rare cases, reduced expression may confer greater vulnerability. This makes BEACON suitable for identifying CYCLOPS-type genes, where reduced expression confers greater vulnerability to perturbation[42].

The strong enrichment of our identified targets with known druggable gene sets highlights the translational potential of our findings (**Figure 5**). While using large human genomic cohort without phenotypes fail to enrich for drug targets[28], recent human cohort studies demonstrate that genetic evidence provided by genome-wide or mendelian genetic associations can successfully provide 2 to 5 fold enrichment for drug targets[44,45]. We note that our approach here, based solely on data from cell line CRISPR screens, provide an orthogonal approach to refine the drug target search space by providing 3.8 fold enrichment for all drug targets and 7-10 fold enrichment for oncology targets.

We complemented our computational findings with experimental validation. Knockdown of *TP63* and *GRHL2* genes in lung squamous tumor cell lines demonstrated reduced colony-forming ability, and the *PAX5* knock-out cell lines from haematopoietic and lymphoid tissue samples showed reduced cell growth, reinforcing the functional relevance of the vulnerability targets (**Figure 6**). Many GED/PED gene targets are lineage-specific transcription factors (TFs); these agree with recent single-cell studies and synthesis that posited the "developmental constraint model of cancer cell states", which cancer cell states correspond to and may be constrained by the landscape of "developmental map"[46]. Thus, a cancer cell adopting a specific developmental state may require activation of such transcription factors and become genetically dependent. Traditionally, TFs were not easily addressable using small molecule or antibody-based approaches due to their lack of binding pockets and complex intermolecular interactions. While such targets used to be considered undruggable, new drug modalities such as proteolysis-targeting chimera (PROTAC) are showing promises[43,47-50], particularly in cases where there may be a sufficient therapeutic window in inhibiting these TFs, e.g., to treat adult tumors where the target TFs were only essential in early development and in tumor cells.

While our study presents a novel approach to identifying cancer dependencies, several limitations warrant discussion. The reliance on cell line models, despite their widespread use, may not fully capture the complexity of tumor heterogeneity and the tumor microenvironment *in vivo*. Future studies should aim to validate these findings in patient-derived xenografts and clinical samples to confirm their translational potential. Moreover, our Bayesian approach BEACON, while robust (**Figure S2**), is constrained by the quality and completeness of available data (**Figure S1**). Expanding proteomic and transcriptomic datasets that capture the full array of cancer cell heterogeneity across tissue lineages will further improve the reliability of GED/PED identification. It is also important to note that within a given lineage, molecular and clinical subtypes (e.g., ER^+ vs. ER^- breast cancer) may harbor distinct dependencies that could be masked when analyzing at the lineage level. Applying BEACON to subtype-stratified datasets may therefore reveal additional,

clinically relevant vulnerabilities. As larger and better-annotated datasets become available, this represents an important direction for future work. Additionally, exploring combination therapies targeting both mutation-driven and expression-driven dependencies could yield synergistic effects, which could be explored in the future.

Overall, our study highlights the potential of expression-driven dependencies as a valuable method for identifying novel therapeutic targets in precision oncology. By integrating multi-omics and CRISPR screen data, we have expanded the repertoire of actionable targets beyond mutated genes for further clinical development, offering new possibilities for cancer treatment.

Methods

Data Sources

We used the CCLE mRNA expressions data[35] and CCLE quantitative proteomics data[24], and from each dataset we excluded the 26 lineages containing data shared in fewer than 7 cell lines, i.e., Adrenal cortex, Autonomic ganglia, Biliary tract, Brain, Cervix, Colon, Eye, Fibroblast, Melanoma Eye(Skin), Osteosarcoma, Placenta, Pleura, Primary, Prostate, Salivary gland, Skin CJ1(2,3) resistant, Skin FV1(2,3) resistant, Small intestine, Testis, Thyroid, and Uvea. We used the DepMap Public 22Q2 data release from the Cancer Dependency Map Project (DepMap)[5], which contained the CRISPR knockout screens (Achilles project[25-27]) for 19,221 genes in 1840 cell lines, including both normal and cancer cell lines, corresponding to 33 primary diseases and 30 lineages. We used the druggable gene lists curated in Minikel et al.[28] The CRISPR knockout screens and mRNA expressions datasets were downloaded from depmap portal[51,52]. The proteomics datasets were downloaded from Nusinow et al.[24]. The druggable gene lists were downloaded from the corresponding studies given in Minikel et al.,[28] and from the DrugBank resource (release 5.1.7).

mRNA expression-driven dependency (GED)

To measure the expression-driven dependency of targets we reviewed correlation-based methods utilizing the two variables[53-55], which are adopted to develop a Bayesian approach that we named BEACON. For each gene, BEACON calculated the Bayesian correlation between the gene's expressions and CERES cancer dependency scores[25] across the pan-lineage cell lines. BEACON modeled expression levels and dependency scores as the bivariate Gaussians and used Markov Chain Monte Carlo (MCMC) sampling to estimate the correlation coefficient *rho* between them. Given the null hypothesis that the uncorrelated expression and dependency of a gene has the 0 *rho* coefficient, we statistically tested each gene's *rho* estimate obtained from the MCMC simulation as follows. Assume that the MCMC sampling is carried out for a null gene's expression and dependency, then we expect that the distribution of the *rho* estimate accumulated over the MCMC iterations will be centered at zero. Based on this rationale, we computed the z-score of *i*-th gene as the deviation of the MCMC estimate of *rho* from the expected (null) value (i.e., zero) in terms of the standard deviation observed in the simulated distribution, i.e., $z(i) = rho_{MCMC}(i) / SD_{MCMC}(i)$. Since the z-values, by nature, follow a normal distribution with zero-mean and unit-variance, then we computed the p-value for each gene's *rho* estimate as the probability of observing a value as extreme as the computed z-value for that gene. We multi-testing corrected the resulting p-values using the BH procedure for FDR. Overall, 4445 genes showed significant pan-lineage expression-driven dependency at the FDR of 0.05. We run the MCMC simulations in R (v3.6) by using *rjags* package (v4-10) with *JAGS* library (v4.3.0).

Compared to other methods that quantify the relationship between two variables, the Bayesian correlation (rho) yielded more intuitive results in the cases with small sample size, while other methods often deviated to spurious correlations imposed by outliers in the data. We benchmarked both methods by simulating expression and dependency datasets at various correlation levels (from -1 to 1, with 0.25 intervals) and sample size (number of cell lines, 5, 7, 10, 20, 30, 60, 100), with different fraction (0.1, 0.3, 0.5, 0.8,

1) of samples being outliers (**Figure S2**). Through rigorous simulations, we observed that the Bayesian method is better than Pearson correlation for estimating moderate true correlation (|rho| < 0.75) in small sample size (~10 cell lines or fewer). Bayesian method is also preferable in noisy data (noise level ≥ 0.5, i.e., 50% or more of the samples are corrupted by noise to become outliers), regardless of sample size or true correlation level. For large samples (≥60), both methods have similar performance in all settings. Pearson method is only better at detecting fewer (≤20 cell lines) and highly-correlated samples |rho| ≥ 0.75, when there is less noise (≤0.1), while Spearman better captured monotonic non-linear trends, though this advantage largely disappeared in small, noisy cohorts (**Figure S2**).

We systematically compared BEACON GEDs and PEDs with results from alternative approaches, including Pearson correlation (as used in Project DRIVE and BACON) and Spearman correlation (also used in BACON). Across cancer lineages, BEACON achieved stronger enrichment—measured by higher AUPRC—for known oncogenes and druggable genes, demonstrating that its advantages extend to real data (**Figure S3**). We report AUPRC rather than AUROC because AUROC can be misleading under severe class imbalance, where positives (druggable genes) are sparse relative to the large number of negatives, which is common in genome-scale analyses. In contrast, AUPRC more appropriately summarizes performance by focusing on the precision–recall tradeoff, making it a more informative metric for evaluating a method's ability to prioritize true druggable genes among many candidates.

For lineage-wise expression-driven dependency analyses, we stratified by lineage the gene expressions and cancer dependency scores across cell lines, and for each gene we calculated the Bayesian correlation (and the corresponding P value and FDR as in the pan-lineage case) between the gene's expressions and cancer dependency scores over the lineage cell lines. In median, 270 gene expression-driven dependencies were significant (FDR < 0.05) per lineage. To further identify the lineage-specific targets, we defined a target's specificity to a given lineage by the difference between the target's ED

score computed within that lineage and the target's average ED score computed in other lineages.

Protein expression-driven dependency (PED)

We adopted the aforementioned procedures for analyzing the protein expression-driven dependency. For this, we used the MS proteomics data obtained for 375 cell lines and 22 lineages[24]. For the pan-lineage analysis, we found 907 proteins showing significant expression-driven dependency (FDR < 0.05). For the lineage-wise analyses, we found, in median, 70 proteins per lineage showing significant expression-driven dependency (FDR < 0.05).

Pathway enrichments from GEDs/PEDs

We used *clusterProfiler*[33] R package (v4.8.3) for functional enrichment analyses of our identified GED and PED sets, and reported the enrichment GO categories at the BH-adjusted p-value cutoff of 0.05.

Association of GEDs/PEDs with drug targets

We tested the association between the drug targets and the genes (proteins) that showed significant (rho < -0.25, FDR < 0.05) pan-lineage ED by using the Fisher's exact test of independence (two-sided). More precisely, given a set of druggable genes, a set of GEDs (PEDs), and the list of total quantified targets in transcriptome (proteome), we calculated the probability of obtaining the observed data and its more extreme deviations in the contingency table consisting of (i) the number of drug targets quantified in the transcriptome (proteome), (ii) the number of GEDs (PEDs) quantified in the transcriptome (proteome), (iii) the number of drug targets quantified in the transcriptome (proteome) that also showed significant ED, and (iv) the remaining number of genes (proteins) that were not drug targets nor showed significant ED, under the null hypothesis that the relative proportions are the same – that the fractions of genes that were drug targets are the same

whether the genes show significant ED or not. We found that the pan-lineage GEDs (PEDs) were significantly associated (OR > 2, FDR < 0.05) with the 10 (12) of the druggable gene lists in Minikel at al.[28]

Methods for the Experimental Validation of *TP63* and *GRHL2*

Cell culture

The human lung squamous cell carcinoma (LSCC) cell lines, HARA and KNS62 (The Japanese Cancer Research Resource Bank; JCRB, Osaka, Japan), NCI-H1703 and HCC-15 (kindly provided by Dr. John D Minna) were used. KNS62 was cultured in E-MEM culture medium (FUJIFILM Wako Pure Chemical Corporation, Osaka, Japan) containing 20% fetal bovine serum (Sigma-Aldrich Japan, Tokyo, Japan) supplemented with 100 U/mL penicillin and streptomycin sulfate (FUJIFILM Wako Pure Chemical Corporation). The others cell lines were cultured in RPMI-1640 culture medium (FUJIFILM Wako Pure Chemical Corporation) containing 10% fetal bovine serum (Sigma-Aldrich Japan) supplemented with 100 U/mL penicillin and streptomycin sulfate (FUJIFILM Wako Pure Chemical Corporation). All cultured cells were incubated at 37 °C in a humidified atmosphere of 5% CO2 and maintained in continuous exponential growth by passaging. All cell lines were obtained from the reliable biobanks with authentication (**Table S15**). Mycoplasma test was performed in regular basis from the first culture of the cells to verify the cells to be the same as the cells registered.

Plasmid DNA constructs

The shRNA-targeted sequences were listed in **Table S14**. For the constructions of plasmids to express shRNA against target genes, double-stranded oligonucleotides were cloned into the pLKO.1-TRC vector (Addgene, #10878). A nonsense scrambled oligonucleotide was used as a negative control. All of the inserted DNA fragments were confirmed by performing DNA sequencing.

Lentivirus-mediated transient expression of the constructs in LSCCs

HEK293T cells were transfected with the constructed plasmids along with lentiviral packaging plasmids pVSV-G, pMDL/pPRE and pRSV-REV (Addgene) using a calcium phosphate method. The lentiviral-containing media were collected 72 h after the transfection, filtered through a 0.45 µM filter, then aliquoted and stored at -130°C until use. Cultured LSCC cells were infected with packaged lentiviruses to express shRNA constructs; after 48 hours of culture, the cells were treated with 2.5 µg/ml (HCC15) or 5 µg/ml (HARA, KNS-62, H1703) puromycin (Thermo Fisher, # A1113803) and cultured for 24 hours (HARA, KNS-62, H1703) or 48 hours (HCC15), and used for transient experiments.

RNA extraction and Quantitative PCR analysis

Gene expression levels were examined by quantitative PCR analysis. Briefly, total RNA was isolated from cells using ISOGEN II (Nippon Gene, #311-07361) and purified using RNeasy Mini Kit (Qiagen). Total RNA (500 ng) was reverse transcribed to cDNA using ReverTra Ace™ (TOYOBO, #FSQ-101). Quantitative PCR was performed using primers listed in **Table S14**, Thunderbird SYBR Green Master Mix (TOYOBO, #QPS-201) and StepOne Plus Real-Time PCR System (Thermo Fisher).

Cell proliferation and cytotoxic assay

Cell viability was analyzed using the Cell Counting Kit-8 (CCK-8) (Dojindo Laboratories, Kumamoto, Japan: CK04). Cells were seeded 5 x 103/100 µL per well in 96-well plates. After 1 h incubation at 37 °C, 10 µL of CCK-8 solution was added to each well and incubated at 37 °C for 2 h. The absorbance was detected at 450 nm using a plate reader (ThermoFisher Maltiskan FC) according to the manufacturer's instructions. Cell viability was normalized against the sh-negative control after 24 h of transfection and the data expressed as a ratio against control after 96 h of transfection.

Colony formation assay

Cells were seeded 1,300-5,000 cells (5,000 cells for HARA, 1,300 cells for KNS-62, 1,500 cells for H1703, 2,000 cells for HCC15) per well into 12 well plates (3.8 cm^2, Corning Japan, Shizuoka, Japan), and cultured for 10 days with the change of culture media every

three days. The cells were then washed by PBS twice, fixed and stained in 0.2% crystal violet dissolved in 20% ethanol, and incubated for 10 minutes at room temperature with gentle shaking. After washing by 1 mL of PBS once and by sterilized water three times, the plate was air dried and photographed. To quantify the colony formation, 1 mL of 50% ethanol (pH 4.2, adjusted by hydrochloric acid) was added into each well of 12-well plates, and incubated for 5 minutes at room temperature with slow shaking, then measured the absorption at 592 nm using a ThermoFisher Maltiskan FC (ThermoFisher). Each experiment was performed with 3 replicate wells.

Statistical analysis

Data were analyzed using R version 4.0.3 (The R Foundation for Statistical Computing, Vienna, Austria) in combination with R studio version 1.2.5033 (R studio, Boston, MA, USA). Welch two sample t-test was used to examine statistical difference between two groups.

Methods for the Experimental Validation of PAX5

Tissue culture

All cell lines used in this study were maintained at 37°C with 5% CO^2. HEL and SU-DHL4 cells were cultured in RPMI 1640 (Corning) supplemented with 10% FetalPlex serum (Gemini) 1% L-Glutamine (Corning), and 1% Penicillin Streptomycin (Gibco). Kasumi-1 cells were cultured in RPMI 1640 (Corning) supplemented with 15% FetalPlex serum (Gemini) 1% L-Glutamine (Corning), and 1% Penicillin Streptomycin (Gibco). REH cells were cultured in IMDM (Gibco) supplemented with 10% heat inactivated FBS (R&D Systems), 1% L-Glutamine (Corning), and 1% Penicillin Streptomycin (Gibco). All cell lines were confirmed by STR profiling.

Genome Editing

The CRISPR/Cas9 system was used to genetically engineer cell lines via ribonucleoprotein (RNP) complex delivery as previously described (Layden et al., 2021). Briefly, a crRNA (IDT) targeted to exon 4 of PAX5, CTTTTGTCCGGATGATCCTG, was annealed with tracrRNA (IDT). Control RNP complexes were formed without the crRNA.

Annealed gRNA were incubated with S.p. Cas9 Nuclease (IDT) to form RNP complexes and electroporated into 1.25 million cells per condition using the NEON transfection system (ThermoFisher). Cells were grown for 72 hours and knockout efficiency was assessed by western blot. Electroporations were performed in biological triplicate for each condition.

Growth Analysis

Cells were allowed to recover for 72 hours post electroporation and then were reseeded to 0.2-0.5 x 10^6 depending on cell line. Reseeded cells were incubated for 72 hours at 37°C with 5% CO_2. Cells were mixed with Trypan Blue (Gibco) and counted with a hemocytometer. Cells were counted in technical triplicate for each biological replicate.

Western Blots:

Protein was isolated from cells lysed with RIPA buffer (50 mM Tris pH 8.0, 150 mM NaCl, 1% NP-40, 0.5% sodium deoxycholate, 0.1% SDS) and sonicated before centrifugation. Protein concentration was quantified using the DC Protein Assay kit (BioRad). Equal amounts of protein were boiled in Laemmli buffer and run on SDS-PAGE gels. Proteins were transferred to a PVDF membrane and membranes were blocked in 5% BSA in PBS. Membranes were incubated with the indicated primary antibodies diluted in 5% BSA in PBS-T followed by incubation with IRDye 800CW and 680RD secondary antibodies (LI-COR Biosciences) diluted in PBS-T + 0.01% SDS. Blots were imaged using the Odyssey Imaging System (LI-COR Biosciences). The following primary antibodies were used at a 1:1000 dilution: PAX5 (Santa Cruz; A-11) and VCL (Santa Cruz; 7F9). All secondary antibodies (IRDye 680 Donkey anti-Rabbit and IRDye 800 Donkey anti-Mouse, Licor) were used at a 1:5000 dilution.

Statistics

Statistical analyses were performed in R (version 4.1.0). Unpaired two-sample t-tests were used to determine significance between conditions.

FIGURE LEGENDS

Figure 1. Study overview. (A) The integration of global proteomic and transcriptomic profiles from 375 cancer cell lines across 22 tissue types in the Cancer Cell Lines Encyclopedia (CCLE), with cancer cell dependency scores derived from CRISPR knockout screens (Achilles). (B) BEACON identifies expression-driven dependency (ED) by using a Bayesian estimation of the correlation coefficient between gene/protein expression and cancer cell dependency data across the cell lines for a representative gene (e.g., *TP63*). (C) Comparison of gene/protein EDs revealed potential markers showing consistency at different molecular levels or arising post-transcriptionally. (D) Heatmaps showing pan-cancer expression-driven dependencies, GED (above) and PED (below), revealing dependencies that are common across multiple cancer types. (E) Heatmaps illustrating cancer-specific expression-driven dependencies, GED (above) and PED (below), identifying dependencies unique to specific cancer types. (F) Identification of new potentially actionable targets that are strongly associated with druggable gene lists catalogued in DrugBank[28], highlighting their therapeutic potential.

Figure 2. Gene Expression-driven Dependency (GED). (A) Heatmap illustrating pan-lineage and lineage-specific gene expression-driven dependencies (GEDs) across various cancer types. Each square represents the correlation (rho) between gene expression and dependency (CERES scores) in the respective tissue types. Significant dependencies are highlighted with bold outlines (FDR < 0.05 in black, FDR < 0.15 in grey). (B) Scatter plots showing examples of gene expression vs. dependency correlations for selected genes (*TP63, CCND1, CCND2, KLF5*) with significant pan-lineage dependencies. Data points (cell lines) are colored by tissue type. (C) Scatter plots demonstrating lineage-specific dependencies for selected genes (*AUNIP1, CD47, SLC4A1, ESR1*). Data points are colored by tissue type, highlighting lineage-specific associations. (D) Pathway enrichment analysis of lineage-specific GEDs, visualized as a heatmap. Each cell indicates the ED score of a particular pathway gene (column) in a specific tissue type (row), with genes grouped (colored) by functional pathways.

Figure 3. Protein Expression-driven Dependency (PED). (A) Heatmap illustrating pan-lineage and lineage-specific protein expression-driven dependencies (PEDs) across various cancer types. Each square represents the correlation (rho) between protein expression and dependency (CERES scores) in the respective tissue types. Significant dependencies are highlighted with bold outlines (FDR < 0.05 in black, FDR < 0.15 in grey). (B) Scatter plots showing examples of protein expression vs. dependency correlations for selected genes (CCND1, KLF5, ELMO2, IRS2) with significant pan-lineage dependencies. Data points (cell lines) are colored by tissue type. (C) Scatter plots demonstrating lineage-specific dependencies for selected genes (TMEM208, GALNT14, GTSF1, PAX5). Data points are colored by tissue type, highlighting lineage-specific associations. (D) Pathway enrichment analysis of lineage-specific PEDs, visualized as a heatmap. Each cell indicates the ED score of a particular pathway gene (column) in a specific tissue type (row), with genes grouped (colored) by functional pathways.

Figure 4. mRNA vs Protein expression-driven dependency. (A) Scatter plot illustrating the correlation between pan-lineage GEDs and PEDs across genes. Genes with consistent significant pan-lineage dependencies at both mRNA and protein levels are highlighted, including *SOX10, TP63, IRF4,* and *MYB*. Additional significant pan-lineage GEDs without corresponding PEDs (e.g., *MYCN, OTX2, EBF1*) and PEDs without corresponding GEDs (e.g., *ELMO2, PRDM6, FGFR3*) are also indicated. (B) Scatter plots showing the correlation between tissue-level GEDs and PEDs within specific lineages.

Figure 5. Leveraging Expression-Driven Dependency to Enrich for Drug Targets. (A) Enrichment (Fisher's exact test) results demonstrating the enrichment of identified GEDs and PEDs in druggable gene lists curated by DrugBank, including all approved drug targets, drug targets by indication, and by drug modality. (B-C) The density plots of ED scores from drug targets versus other genes, highlighting the top significant targets identified at (B) mRNA and (C) protein levels. (D) Scatter plots of expression vs. dependency correlations for top drug targets and other genes, showing significant pan-

lineage ED at both mRNA and protein levels (e.g., *SOX10, TP63, IRF4, CCND1*). Data points (cell lines) are colored by tissue type.

Figure 6. Functional validation of expression-driven dependency targets, *TP63*, *GRHL2*, and PAX5, in lung squamous cancer cell and hematopoietic cell lines. (A) Colony formation assay in LSCC cell lines (KNS-62, H1703, and HARA) upon knockdown of TP63 using two shRNA constructs (sh-TP63-1 and sh-TP63-2). Significant reduction in colony formation was observed compared to sh-negative control cells ($p<0.01$). ns: non-significance between control and sh-negative cells. Each experiment was performed with 3 replicate wells, where error bars show mean ± standard deviation; this also applies to panel B. (B) Colony formation assay in LSCC cell lines (KNS-62, H1703, HARA, and HCC15) upon knockdown of GRHL2 using two shRNA constructs (sh-GRHL2-1 and sh-GRHL2-2). Significant decrease in colony formation was seen compared to sh-negative control cells ($p<0.01$). (C) PAX5 mRNA and protein expression levels in myeloid (HEL, Kasumi-1) and B-cell (REH, SU-DHL4) lineage cell lines. PAX5 showed lineage-specific expression-driven dependency. (D) Effect of PAX5 knockout (KO) via CRISPR on cell viability in PAX5-high B-cell lines (REH, SU-DHL4) and PAX5-low myeloid lines (HEL, Kasumi-1). PAX5 KO significantly reduced live cell numbers in REH and SU-DHL4 ($p<0.05$ and $p<0.01$, respectively), but not in HEL and Kasumi-1. In (D) left, protein levels were assessed by anti-PAX5 72 hours after electroporation. VCL serves as a loading control. In (D) right, cells were electroporated with RNP complexes with (KO) or without (ng) PAX5 crRNA and allowed to recover for 72 hours. After recovery ng and KO cells were reseeded at equal densities and live cells were counted by trypan blue exclusion after 72 hours. Cells were counted in technical triplicate for each biological replicate (n=3).

Supplementary Figure Legends

Figure S1. Data overview. (A) Analyses were restricted to lineages with at least 7 cell lines having cancer cell line dependency and corresponding mRNA/protein expression data to ensure statistical robustness. (B) 855 cell lines across 17 lineages were analyzed, sharing cancer cell dependency scores and corresponding mRNA and protein expressions. The limited sample size per cell lineage may lead to spurious correlations,

especially for protein expression. (C) The distribution of protein quantification per cell line. Over 12,000 proteins (in total) were quantified across all samples, where a majority of the samples reached a quantification level of over 9,000 proteins[24].

Figure S2. Benchmarking of BEACON against Pearson and Spearman correlations in simulated data. The performance are measured by mean squared-error (MSE, y-axis) for the same data sets randomly simulated for various true correlation levels (rho, x-axis), under different conditions of noise interference (columns, 0.1, 0.3, 0.5, 0.8, 1) and sample size (rows, 5, 7, 10, 20, 30, 60, 100).

Figure S3. Systematic benchmarking of BEACON against Pearson and Spearman correlations in real data. (A) AUPRC values for identifying DGIdb druggable genes (n = 2,993) (left) and the transcription factors previously identified in Project DRIVE (n = 57) (right) based on gene expression–dependency (GED) associations across 23 cancer lineages. (B) AUPRC values for identifying the same druggable gene sets in *panel A* based on protein expression–dependency (PED) associations across 17 lineages.

Figure S4. Analysis of Druggable Gene Expression Dependencies (GEDs) and Protein Expression Dependencies (PEDs). (A) Heatmap illustrating pan-lineage and lineage-specific druggable gene expression-driven dependencies (GEDs) across various cancer types. Each square represents the correlation (rho) between gene expression and dependency (CERES scores) in the respective tissue types. Significant dependencies are highlighted with bold outlines (FDR < 0.05 in black, FDR < 0.15 in grey). Integration of the drug-gene interaction database (DGIdb) identified 82 druggable factors showing pan-lineage GED and 951 tissue-specific druggable targets, in total, showing significant GED across all lineages. (B) Analysis of tissue-specific expression-driven dependencies across tissues revealed 951 significant druggable targets, including 132 for hematopoietic and lymphoid tissue, and 101 for lung. (C) Clustering GED measures of druggable genes across tissue types showed that pancreatic and biliary tract cancer cells, as well as kidney and urinary tract cancer cells, have similar druggable profiles. (D) The breast-specific *ESR1* transcription factor clustered with other factors such as *FOXA1, SPDEF, TBX3,* and *TRPS1*, showing strong GED levels in breast tissue cell lines. (E) Integration of DGIdb for PEDs identified 170 significant lineage-specific PEDs, with notable targets

including PAX5 in hematopoietic and lymphoid tissue, and SOX10 in the central nervous system.

Figure S5. Enrichment (Fisher's exact test) results demonstrating the enrichment of identified GEDs and PEDs in druggable gene lists based on DrugBank (likely mechanism of action) and genetic effect gene lists as described in Methods.

Figure S6. Dependencies of *GRHL2* and *TP63* in lung squamous cell carcinoma (LSCC) cell lines. (A) mRNA expression levels of *GRHL2* and *TP63* in TCGA LUSC tumor vs. normal tissues in TCGA, where both genes show elevated expression in LSCC compared to normal adjacent lung tissue. (B) Scatter plots showing the relationship between *TP63* mRNA and protein expression levels vs. gene dependency (CERES score) across various cell lines. (C) CCK-8 cell proliferation assays in LSCC cell lines (KNS-62 and H1703) upon knockdown of *TP63* using two shRNA constructs (sh-TP63-1 and sh-TP63-2). Proliferation is shown relative to day 1. Data represent mean ± SD from three independent replicate wells (N = 3), where error bars indicate standard deviation (SD). This also applies to *panels D-E*. (D) CCK-8 cell proliferation assays in LSCC cell lines (KNS-62 and H1703) upon knockdown of *GRHL2* using two shRNA constructs (sh-GRHL2-1 and sh-GRHL2-2). Proliferation is shown relative to day 1. (E) qPCR result of shRNA infected HARA cell line, showing the reduced expression levels of *TP63* and *GRHL2* compared to untreated cells.

Figure S7. Dependency scores vs. gene expression levels of (A) *GRHL2, (B) TP63,* and (C) *PAX5* as provided by DepMap RNAi and CRISPR screen data across all cell lines with available data (public 25Q2 data release). The x-axis denotes normalized gene expression log2(TPM+1) of the respective genes and the y-axis denoted the gene knockdown effect from RNAi (DEMETER2 score) (left panels) or the gene knockout effect from CRISPR (Cronos score) (right panels) screens. The cell types that were used in our functional experiments (Figure 6) were labeled.

Supplementary Data. Spreadsheets containing the supplementary tables (S1-S15), including the related data used for plotting main figures and supplementary figures.

Table S1. Data availability for all lineage cell lines.

Table S2. BEACON results of pan-lineage GEDs (tissue-specific GEDs – sheet2).

Table S3. BEACON results of pan-lineage druggable GEDs (tissue-specific druggable GEDs – sheet2).

Table S4. Proteins showing significant (rho < -0.25, FDR < 0.05) pan-lineage PED with significant GED (without a significant GED – sheet2).

Table S5. BEACON results of pan-lineage druggable PEDs.

Table S6. BEACON results of tissue-specific PEDs (tissue-specific druggable PEDs – sheet2).

Table S7. Gene ontology enrichment analysis for the tissue-specific PEDs.

Table S8. Genes showing consistently significant (rho < -0.25, FDR < 0.05) pan-lineage expression-driven dependency in both mRNA and protein levels (in only mRNA levels – sheet2).

Table S9. Table showing consistency between tissue-level GEDs and PEDs for each lineage.

Table S10. Results of Fisher's exact test evaluating the association between the druggable gene lists and the pan-lineage GEDs/PEDs.

Table S11. GEDs/PEDs enriched for druggable targets curated by the DrugBank.

Table S12. Druggable genes significantly enriched with expression-driven dependency observed in both mRNA and protein level expressions.

Table S13. Other genes (not drug targets) significantly enriched with expression-driven dependency observed in both mRNA and protein level expressions

Table S14. The shRNA-targeted sequences and primers used for LSCC experiments.

Table S15. Cell line source table.

DATA AND SOFTWARE AVAILABILITY

Data Availability

Data for DepMap/CCLE genetic screens and mRNA/protein expressions can be found on DepMap data portal: https://depmap.org/portal/.

Code Availability

The source code for BEACON is available at https://github.com/Huang-lab/BEACON. This tool has been registered at bio.tools under the identifier biotoolsID: BEACON-x, and at SciCrunch.org under the identifier RRID: SCR_027484.

ACKNOWLEDGEMENTS

The authors wish to acknowledge data from the Cancer Dependency Map project and its contributors. The authors thank all members of the Huang lab for constructive discussion. This work was supported in part through the computational and data resources and staff expertise provided by Scientific Computing and Data at the Icahn School of Medicine at Mount Sinai and supported by the Clinical and Translational Science Awards (CTSA) grant UL1TR004419 from the National Center for Advancing Translational Sciences. Research reported in this publication was also supported by the Office of Research Infrastructure of the National Institutes of Health under award number S10OD026880 and S10OD030463. The content is solely the responsibility of the authors and does not necessarily represent the official views of the National Institutes of Health. This work was supported by NIH NIGMS R35GM138113 and ACS RSG-22-115-01-DMC to KH.

COMPETING FINANCIAL INTERESTS

K.H. is a co-founder and board member of a non-for-profit organization, Open Box Science, where he does not receive any compensation. All other authors declare no competing interests.

CONTRIBUTIONS

KH conceived the research, and AE and KH designed the approach and computational analyses. AE developed the Bayesian approach and conducted the bioinformatics analyses, which is supervised by KH. HL, JE, LB, XZ, RI, HO, and SH designed and conducted the experiments. AE, HL, SH, and KH wrote the manuscript. All authors read, edited, and approved the manuscript.

Use of AI Tools and Technologies in Writing

During the preparation of this work the authors used ChatGPT, Perplexity, and Claude in order to refine language and assist with editing the authors originally written content for improved readability. After using this tool/service, the authors reviewed and edited the content as needed and take full responsibility for the content of the publication.

REFERENCES

1. Sengupta, S., Sun, S.Q., Huang, K.L., Oh, C., Bailey, M.H., Varghese, R., Wyczalkowski, M.A., Ning, J., Tripathi, P., McMichael, J.F., et al. (2018). Integrative omics analyses broaden treatment targets in human cancer. Genome Med *10*, 60. 10.1186/s13073-018-0564-z.
2. Waarts, M.R., Stonestrom, A.J., Park, Y.C., and Levine, R.L. (2022). Targeting mutations in cancer. J Clin Invest *132*. 10.1172/jci154943.
3. Savage, S.R., Yi, X., Lei, J.T., Wen, B., Zhao, H., Liao, Y., Jaehnig, E.J., Somes, L.K., Shafer, P.W., Lee, T.D., et al. (2024). Pan-cancer proteogenomics expands the landscape of therapeutic targets. Cell. 10.1016/j.cell.2024.05.039.
4. Barretina, J., Caponigro, G., Stransky, N., Venkatesan, K., Margolin, A.A., Kim, S., Wilson, C.J., Lehár, J., Kryukov, G.V., Sonkin, D., et al. (2012). The Cancer Cell Line Encyclopedia enables predictive modelling of anticancer drug sensitivity. Nature *483*, 603-607. 10.1038/nature11003.
5. Tsherniak, A., Vazquez, F., Montgomery, P.G., Weir, B.A., Kryukov, G., Cowley, G.S., Gill, S., Harrington, W.F., Pantel, S., Krill-Burger, J.M., et al. (2017). Defining a Cancer Dependency Map. Cell *170*, 564-576.e516. 10.1016/j.cell.2017.06.010.
6. Bridgett, S., Campbell, J., Lord, C.J., and Ryan, C.J. (2017). CancerGD: A Resource for Identifying and Interpreting Genetic Dependencies in Cancer. Cell Syst *5*, 82-86.e83. 10.1016/j.cels.2017.06.002.
7. Cohen-Sharir, Y., McFarland, J.M., Abdusamad, M., Marquis, C., Bernhard, S.V., Kazachkova, M., Tang, H., Ippolito, M.R., Laue, K., Zerbib, J., et al. (2021). Aneuploidy renders cancer cells vulnerable to mitotic checkpoint inhibition. Nature *590*, 486-491. 10.1038/s41586-020-03114-6.
8. Cervia, L.D., Shibue, T., Borah, A.A., Gaeta, B., He, L., Leung, L., Li, N., Moyer, S.M., Shim, B.H., Dumont, N., et al. (2023). A Ubiquitination Cascade Regulating the Integrated Stress Response and Survival in Carcinomas. Cancer Discov *13*, 766-795. 10.1158/2159-8290.Cd-22-1230.
9. Dharia, N.V., Kugener, G., Guenther, L.M., Malone, C.F., Durbin, A.D., Hong, A.L., Howard, T.P., Bandopadhayay, P., Wechsler, C.S., Fung, I., et al. (2021). A first-generation pediatric cancer dependency map. Nat Genet *53*, 529-538. 10.1038/s41588-021-00819-w.
10. de Matos Simoes, R., Shirasaki, R., Downey-Kopyscinski, S.L., Matthews, G.M., Barwick, B.G., Gupta, V.A., Dupéré-Richer, D., Yamano, S., Hu, Y., Sheffer, M., et al. (2023). Genome-scale functional genomics identify genes preferentially

essential for multiple myeloma cells compared to other neoplasias. Nat Cancer *4*, 754-773. 10.1038/s43018-023-00550-x.

11. Chan, E.M., Shibue, T., McFarland, J.M., Gaeta, B., Ghandi, M., Dumont, N., Gonzalez, A., McPartlan, J.S., Li, T., Zhang, Y., et al. (2019). WRN helicase is a synthetic lethal target in microsatellite unstable cancers. Nature *568*, 551-556. 10.1038/s41586-019-1102-x.

12. Behan, F.M., Iorio, F., Picco, G., Gonçalves, E., Beaver, C.M., Migliardi, G., Santos, R., Rao, Y., Sassi, F., Pinnelli, M., et al. (2019). Prioritization of cancer therapeutic targets using CRISPR-Cas9 screens. Nature *568*, 511-516. 10.1038/s41586-019-1103-9.

13. Waters, A.M., Khatib, T.O., Papke, B., Goodwin, C.M., Hobbs, G.A., Diehl, J.N., Yang, R., Edwards, A.C., Walsh, K.H., Sulahian, R., et al. (2021). Targeting p130Cas- and microtubule-dependent MYC regulation sensitizes pancreatic cancer to ERK MAPK inhibition. Cell Rep *35*, 109291. 10.1016/j.celrep.2021.109291.

14. Bondeson, D.P., Paolella, B.R., Asfaw, A., Rothberg, M.V., Skipper, T.A., Langan, C., Mesa, G., Gonzalez, A., Surface, L.E., Ito, K., et al. (2022). Phosphate dysregulation via the XPR1-KIDINS220 protein complex is a therapeutic vulnerability in ovarian cancer. Nat Cancer *3*, 681-695. 10.1038/s43018-022-00360-7.

15. Mertins, P., Mani, D.R., Ruggles, K.V., Gillette, M.A., Clauser, K.R., Wang, P., Wang, X., Qiao, J.W., Cao, S., Petralia, F., et al. (2016). Proteogenomics connects somatic mutations to signalling in breast cancer. Nature *534*, 55-62. 10.1038/nature18003.

16. Whiteaker, J.R., Halusa, G.N., Hoofnagle, A.N., Sharma, V., MacLean, B., Yan, P., Wrobel, J.A., Kennedy, J., Mani, D.R., Zimmerman, L.J., et al. (2016). Using the CPTAC Assay Portal to Identify and Implement Highly Characterized Targeted Proteomics Assays. Methods Mol Biol *1410*, 223-236. 10.1007/978-1-4939-3524-6_13.

17. Wang, J., Ma, Z., Carr, S.A., Mertins, P., Zhang, H., Zhang, Z., Chan, D.W., Ellis, M.J., Townsend, R.R., Smith, R.D., et al. (2017). Proteome Profiling Outperforms Transcriptome Profiling for Coexpression Based Gene Function Prediction. Mol Cell Proteomics *16*, 121-134. 10.1074/mcp.M116.060301.

18. Zhang, H., Liu, T., Zhang, Z., Payne, S.H., Zhang, B., McDermott, J.E., Zhou, J.Y., Petyuk, V.A., Chen, L., Ray, D., et al. (2016). Integrated Proteogenomic Characterization of Human High-Grade Serous Ovarian Cancer. Cell *166*, 755-765. 10.1016/j.cell.2016.05.069.

19. Rudnick, P.A., Markey, S.P., Roth, J., Mirokhin, Y., Yan, X., Tchekhovskoi, D.V., Edwards, N.J., Thangudu, R.R., Ketchum, K.A., Kinsinger, C.R., et al. (2016). A Description of the Clinical Proteomic Tumor Analysis Consortium (CPTAC) Common Data Analysis Pipeline. J Proteome Res *15*, 1023-1032. 10.1021/acs.jproteome.5b01091.

20. Rodriguez, H., Zenklusen, J.C., Staudt, L.M., Doroshow, J.H., and Lowy, D.R. (2021). The next horizon in precision oncology: Proteogenomics to inform cancer diagnosis and treatment. Cell *184*, 1661-1670. 10.1016/j.cell.2021.02.055.

21. Song, W.M., Elmas, A., Farias, R., Xu, P., Zhou, X., Hopkins, B., Huang, K.L., and Zhang, B. (2023). Multiscale protein networks systematically identify aberrant protein interactions and oncogenic regulators in seven cancer types. J Hematol Oncol *16*, 120. 10.1186/s13045-023-01517-2.
22. Elmas, A., Tharakan, S., Jaladanki, S., Galsky, M.D., Liu, T., and Huang, K.L. (2021). Pan-cancer proteogenomic investigations identify post-transcriptional kinase targets. Commun Biol *4*, 1112. 10.1038/s42003-021-02636-7.
23. Elmas, A., Lujambio, A., and Huang, K.L. (2022). Proteomic Analyses Identify Therapeutic Targets in Hepatocellular Carcinoma. Front Oncol *12*, 814120. 10.3389/fonc.2022.814120.
24. Nusinow, D.P., Szpyt, J., Ghandi, M., Rose, C.M., McDonald, E.R., 3rd, Kalocsay, M., Jané-Valbuena, J., Gelfand, E., Schweppe, D.K., Jedrychowski, M., et al. (2020). Quantitative Proteomics of the Cancer Cell Line Encyclopedia. Cell *180*, 387-402.e316. 10.1016/j.cell.2019.12.023.
25. Meyers, R.M., Bryan, J.G., McFarland, J.M., Weir, B.A., Sizemore, A.E., Xu, H., Dharia, N.V., Montgomery, P.G., Cowley, G.S., Pantel, S., et al. (2017). Computational correction of copy number effect improves specificity of CRISPR-Cas9 essentiality screens in cancer cells. Nat Genet *49*, 1779-1784. 10.1038/ng.3984.
26. Pacini, C., Dempster, J.M., Boyle, I., Gonçalves, E., Najgebauer, H., Karakoc, E., van der Meer, D., Barthorpe, A., Lightfoot, H., Jaaks, P., et al. (2021). Integrated cross-study datasets of genetic dependencies in cancer. Nat Commun *12*, 1661. 10.1038/s41467-021-21898-7.
27. Dempster, J.M., Boyle, I., Vazquez, F., Root, D.E., Boehm, J.S., Hahn, W.C., Tsherniak, A., and McFarland, J.M. (2021). Chronos: a cell population dynamics model of CRISPR experiments that improves inference of gene fitness effects. Genome Biol *22*, 343. 10.1186/s13059-021-02540-7.
28. Minikel, E.V., Karczewski, K.J., Martin, H.C., Cummings, B.B., Whiffin, N., Rhodes, D., Alföldi, J., Trembath, R.C., van Heel, D.A., Daly, M.J., et al. (2020). Evaluating drug targets through human loss-of-function genetic variation. Nature *581*, 459-464. 10.1038/s41586-020-2267-z.
29. McDonald, E.R., de Weck, A., Schlabach, M.R., Billy, E., Mavrakis, K.J., Hoffman, G.R., Belur, D., Castelletti, D., Frias, E., Gampa, K., et al. (2017). Project DRIVE: A Compendium of Cancer Dependencies and Synthetic Lethal Relationships Uncovered by Large-Scale, Deep RNAi Screening. Cell *170*, 577-592.e510. 10.1016/j.cell.2017.07.005.
30. Rohde, T., Demirtas, T.Y., Süsser, S., Shaw, A.H., Kaulich, M., and Billmann, M. (2025). BaCoN (Balanced Correlation Network) improves prediction of gene buffering. Mol Syst Biol *21*, 807-824. 10.1038/s44320-025-00103-7.
31. Mills, A.A. (2006). p63: oncogene or tumor suppressor? Curr Opin Genet Dev *16*, 38-44. 10.1016/j.gde.2005.12.001.
32. Luo, Y., and Chen, C. (2021). The roles and regulation of the KLF5 transcription factor in cancers. Cancer Sci *112*, 2097-2117. 10.1111/cas.14910.
33. Yu, G., Wang, L.G., Han, Y., and He, Q.Y. (2012). clusterProfiler: an R package for comparing biological themes among gene clusters. Omics *16*, 284-287. 10.1089/omi.2011.0118.

34. Cotto, K.C., Wagner, A.H., Feng, Y.Y., Kiwala, S., Coffman, A.C., Spies, G., Wollam, A., Spies, N.C., Griffith, O.L., and Griffith, M. (2018). DGIdb 3.0: a redesign and expansion of the drug-gene interaction database. Nucleic Acids Res *46*, D1068-d1073. 10.1093/nar/gkx1143.
35. Ghandi, M., Huang, F.W., Jané-Valbuena, J., Kryukov, G.V., Lo, C.C., McDonald, E.R., 3rd, Barretina, J., Gelfand, E.T., Bielski, C.M., Li, H., et al. (2019). Next-generation characterization of the Cancer Cell Line Encyclopedia. Nature *569*, 503-508. 10.1038/s41586-019-1186-3.
36. Sharma, D., Kumar, S., and Narasimhan, B. (2018). Estrogen alpha receptor antagonists for the treatment of breast cancer: a review. Chem Cent J *12*, 107. 10.1186/s13065-018-0472-8.
37. Fletcher, M.N.C., Castro, M.A.A., Wang, X., de Santiago, I., O'Reilly, M., Chin, S.-F., Rueda, O.M., Caldas, C., Ponder, B.A.J., Markowetz, F., and Meyer, K.B. (2013). Master regulators of FGFR2 signalling and breast cancer risk. Nature communications *4*, 2464. 10.1038/ncomms3464.
38. Hart, T., Tong, A.H.Y., Chan, K., Van Leeuwen, J., Seetharaman, A., Aregger, M., Chandrashekhar, M., Hustedt, N., Seth, S., Noonan, A., et al. (2017). Evaluation and Design of Genome-Wide CRISPR/SpCas9 Knockout Screens. G3 (Bethesda) *7*, 2719-2727. 10.1534/g3.117.041277.
39. Shaffer, A.L., Emre, N.C., Romesser, P.B., and Staudt, L.M. (2009). IRF4: Immunity. Malignancy! Therapy? Clin Cancer Res *15*, 2954-2961. 10.1158/1078-0432.Ccr-08-1845.
40. Cheung, K.L., Zhang, F., Jaganathan, A., Sharma, R., Zhang, Q., Konuma, T., Shen, T., Lee, J.Y., Ren, C., Chen, C.H., et al. (2017). Distinct Roles of Brd2 and Brd4 in Potentiating the Transcriptional Program for Th17 Cell Differentiation. Mol Cell *65*, 1068-1080.e1065. 10.1016/j.molcel.2016.12.022.
41. Nam, S., and Lim, J.S. (2016). Essential role of interferon regulatory factor 4 (IRF4) in immune cell development. Arch Pharm Res *39*, 1548-1555. 10.1007/s12272-016-0854-1.
42. Nijhawan, D., Zack, Travis I., Ren, Y., Strickland, Matthew R., Lamothe, R., Schumacher, Steven E., Tsherniak, A., Besche, Henrike C., Rosenbluh, J., Shehata, S., et al. (2012). Cancer Vulnerabilities Unveiled by Genomic Loss. Cell *150*, 842-854. 10.1016/j.cell.2012.07.023.
43. Békés, M., Langley, D.R., and Crews, C.M. (2022). PROTAC targeted protein degraders: the past is prologue. Nat Rev Drug Discov *21*, 181-200. 10.1038/s41573-021-00371-6.
44. Minikel, E.V., Painter, J.L., Dong, C.C., and Nelson, M.R. (2024). Refining the impact of genetic evidence on clinical success. Nature *629*, 624-629. 10.1038/s41586-024-07316-0.
45. Nelson, M.R., Tipney, H., Painter, J.L., Shen, J., Nicoletti, P., Shen, Y., Floratos, A., Sham, P.C., Li, M.J., Wang, J., et al. (2015). The support of human genetic evidence for approved drug indications. Nature Genetics *47*, 856-860. 10.1038/ng.3314.
46. Patel, A.S., and Yanai, I. (2024). A developmental constraint model of cancer cell states and tumor heterogeneity. Cell *187*, 2907-2918. 10.1016/j.cell.2024.04.032.

47. Xiao, L., Parolia, A., Qiao, Y., Bawa, P., Eyunni, S., Mannan, R., Carson, S.E., Chang, Y., Wang, X., Zhang, Y., et al. (2022). Targeting SWI/SNF ATPases in enhancer-addicted prostate cancer. Nature *601*, 434-439. 10.1038/s41586-021-04246-z.
48. Bushweller, J.H. (2019). Targeting transcription factors in cancer - from undruggable to reality. Nat Rev Cancer *19*, 611-624. 10.1038/s41568-019-0196-7.
49. Samarasinghe, K.T.G., Jaime-Figueroa, S., Burgess, M., Nalawansha, D.A., Dai, K., Hu, Z., Bebenek, A., Holley, S.A., and Crews, C.M. (2021). Targeted degradation of transcription factors by TRAFTACs: TRAnscription Factor TArgeting Chimeras. Cell Chem Biol *28*, 648-661.e645. 10.1016/j.chembiol.2021.03.011.
50. Samarasinghe, K.T.G., An, E., Genuth, M.A., Chu, L., Holley, S.A., and Crews, C.M. (2022). OligoTRAFTACs: A generalizable method for transcription factor degradation. RSC Chem Biol *3*, 1144-1153. 10.1039/d2cb00138a.
51. DepMap (2021). DepMap: The Cancer Dependency Map Project at Broad Institute. https://depmap.org/portal.
52. DepMap (2022). DepMap 22Q2 Public. figshare. Dataset. https://doi.org/10.6084/m9.figshare.19700056.v2.
53. Tazawa, M. (1968). Motive force of the cytoplasmic streaming in nitella. Protoplasma *65*, 207-222. 10.1007/bf01666379.
54. Kaisary, A.V., and Grant, R.W. (1984). "Beehive on the bladder": an indication of colovesical disease. Br J Urol *56*, 35-37. 10.1111/j.1464-410x.1984.tb07159.x.
55. Jaladanki, S.K., Elmas, A., Malave, G.S., and Huang, K.L. (2021). Genetic dependency of Alzheimer's disease-associated genes across cells and tissue types. Sci Rep *11*, 12107. 10.1038/s41598-021-91713-2.

Figure

Click here to access/download;Figure;expression_dependency_figures.pdf

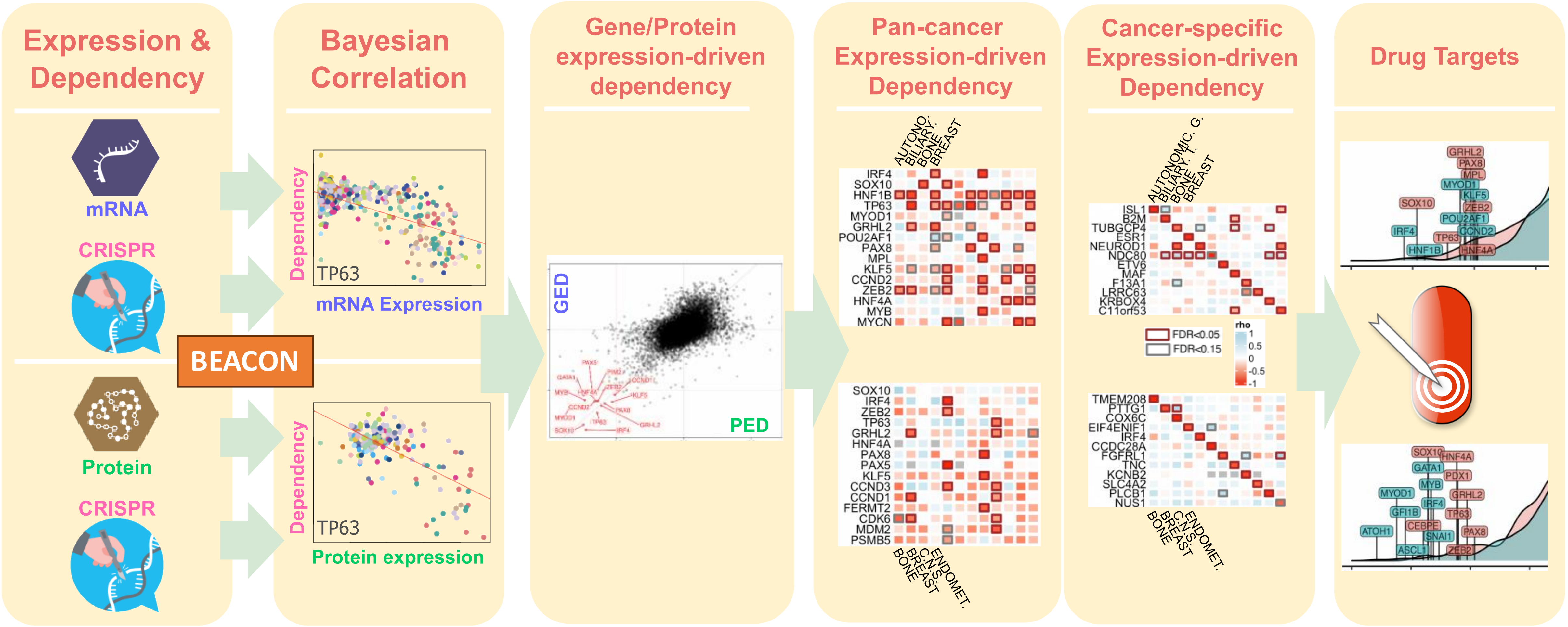

Figure 1. Study overview. (A) The integration of global proteomic and transcriptomic profiles from 375 cancer cell lines across 22 tissue types in the Cancer Cell Lines Encyclopedia (CCLE), with cancer cell dependency scores derived from CRISPR knockout screens (Achilles). (B) BEACON identifies expression-driven dependency (ED) by using a Bayesian estimation of the correlation coefficient between gene/protein expression and cancer cell dependency data across the cell lines for a representative gene (e.g., TP63). (C) Comparison of gene/protein EDs revealed potential markers showing consistency at different molecular levels or arising post-transcriptionally. (D) Heatmaps showing pan-cancer expression-driven dependencies, GED (above) and PED (below), revealing dependencies that are common across multiple cancer types. (E) Heatmaps illustrating cancer-specific expression-driven dependencies, GED (above) and PED (below), identifying dependencies unique to specific cancer types. (F) Identification of new potentially actionable targets that are strongly associated with druggable gene lists catalogued in DrugBank, highlighting their therapeutic potential.

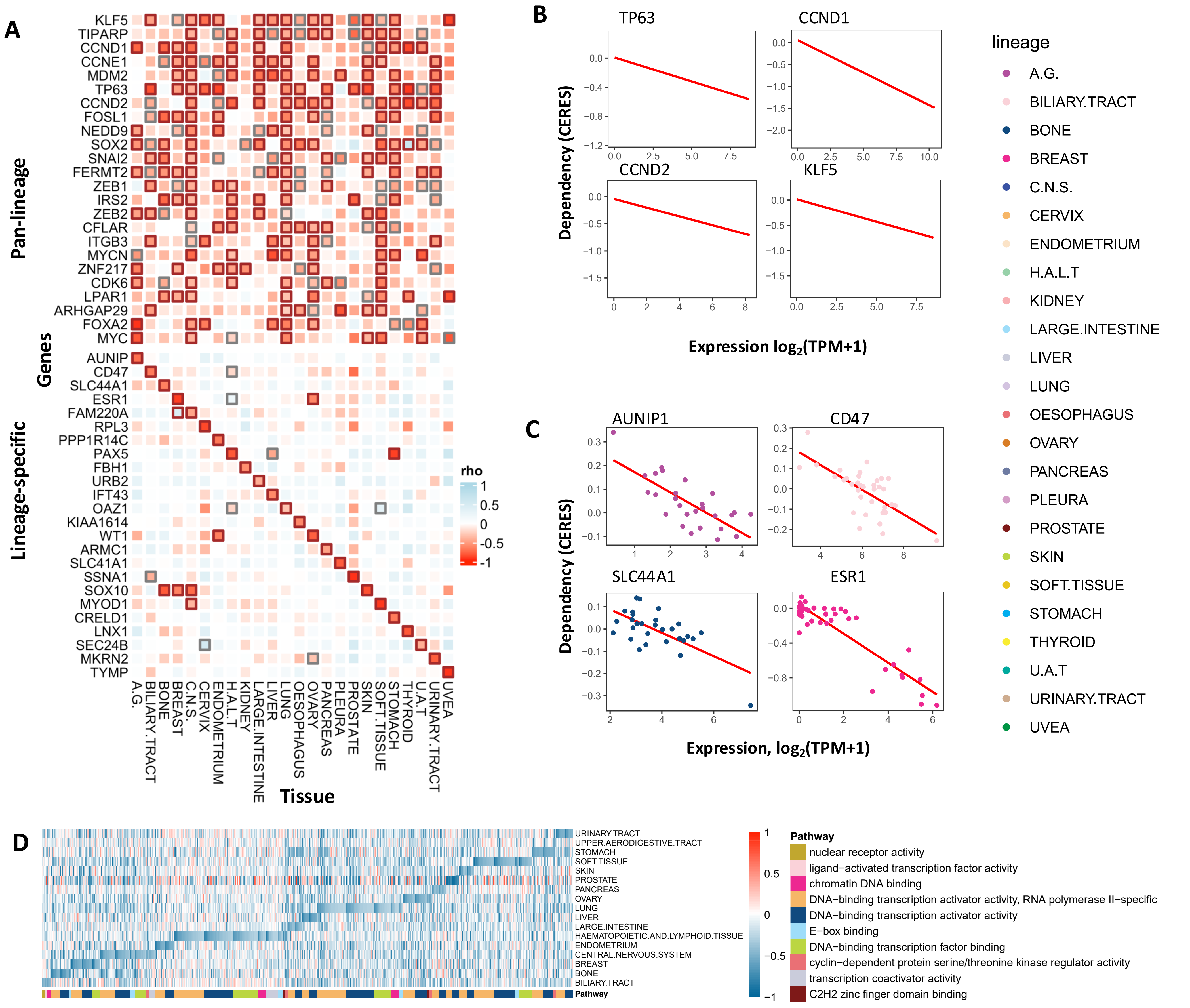

Figure 2. Gene Expression-driven Dependency (GED). (A) Heatmap illustrating pan-lineage and lineage-specific gene expression-driven dependencies (GEDs) across various cancer types. Each square represents the correlation (rho) between gene expression and dependency (CERES scores) in the respective tissue types. Significant dependencies are highlighted with bold outlines (FDR < 0.05 in black, FDR < 0.15 in grey). (B) Scatter plots showing examples of gene expression vs. dependency correlations for selected genes (TP63, CCND1, CCND2, KLF5) with significant pan-lineage dependencies. Data points (cell lines) are colored by tissue type. (C) Scatter plots demonstrating lineage-specific dependencies for selected genes (AUNIP1, CD47, SLC4A1, ESR1). Data points are colored by tissue type, highlighting lineage-specific associations. (D) Pathway enrichment analysis of lineage-specific GEDs, visualized as a heatmap. Each cell indicates the ED score of a particular pathway gene (column) in a specific tissue type (row), with genes grouped (colored) by functional pathways.

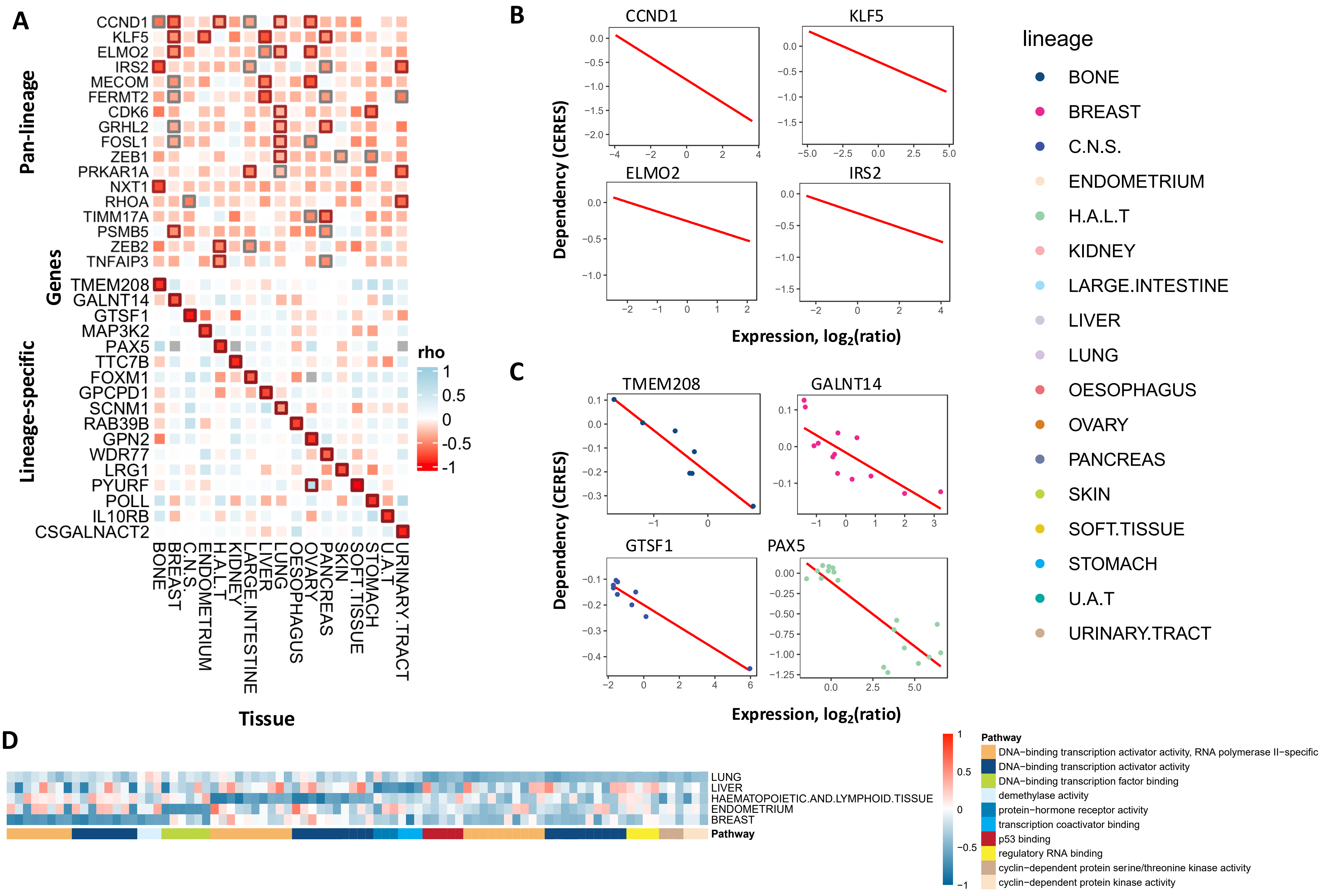

Figure 3. Protein Expression-driven Dependency (PED). (A) Heatmap illustrating pan-lineage and lineage-specific protein expression-driven dependencies (PEDs) across various cancer types. Each square represents the correlation (rho) between protein expression and dependency (CERES scores) in the respective tissue types. Significant dependencies are highlighted with bold outlines (FDR < 0.05 in black, FDR < 0.15 in grey). (B) Scatter plots showing examples of protein expression vs. dependency correlations for selected genes (CCND1, KLF5, ELMO2, IRS2) with significant pan-lineage dependencies. Data points (cell lines) are colored by tissue type. (C) Scatter plots demonstrating lineage-specific dependencies for selected genes (TMEM208, GALNT14, GTSF1, PAX5). Data points are colored by tissue type, highlighting lineage-specific associations. (D) Pathway enrichment analysis of lineage-specific PEDs, visualized as a heatmap. Each cell indicates the ED score of a particular pathway gene (column) in a specific tissue type (row), with genes grouped (colored) by functional pathways.

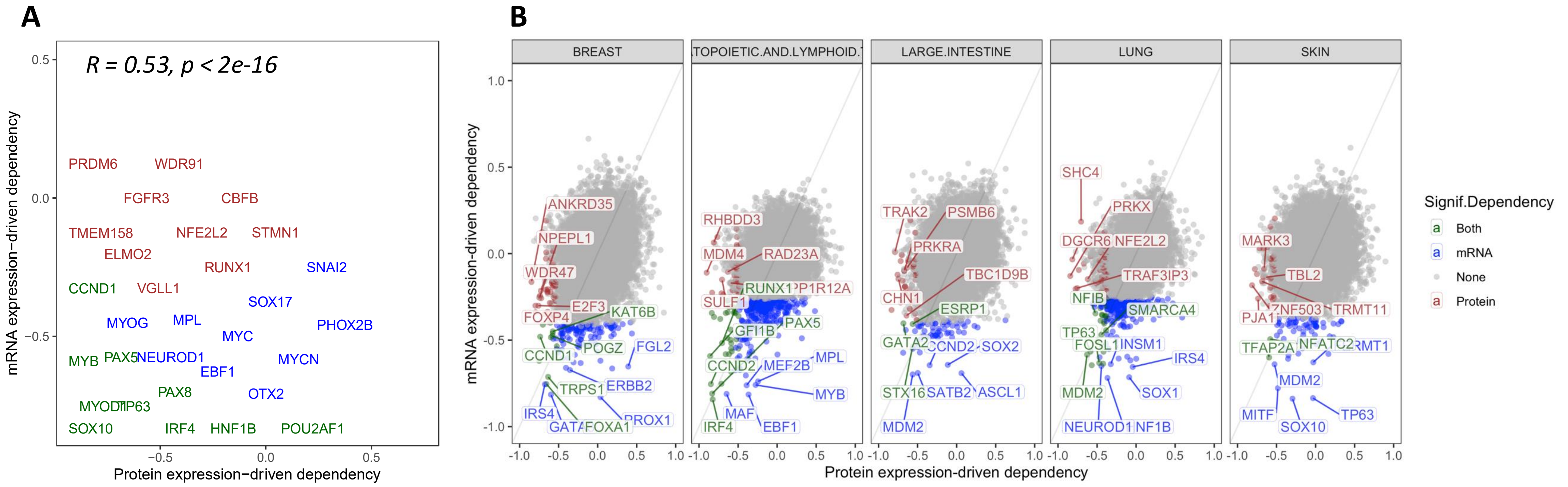

Figure 4. mRNA vs Protein expression-driven dependency. (A) Scatter plot illustrating the correlation between pan-lineage GEDs and PEDs across genes. Genes with consistent significant pan-lineage dependencies at both mRNA and protein levels are highlighted, including SOX10, TP63, IRF4, and MYB. Additional significant pan-lineage GEDs without corresponding PEDs (e.g., MYCN, OTX2, EBF1) and PEDs without corresponding GEDs (e.g., ELMO2, PRDM6, FGFR3) are also indicated. (B) Scatter plots showing the correlation between tissue-level GEDs and PEDs within specific lineages.

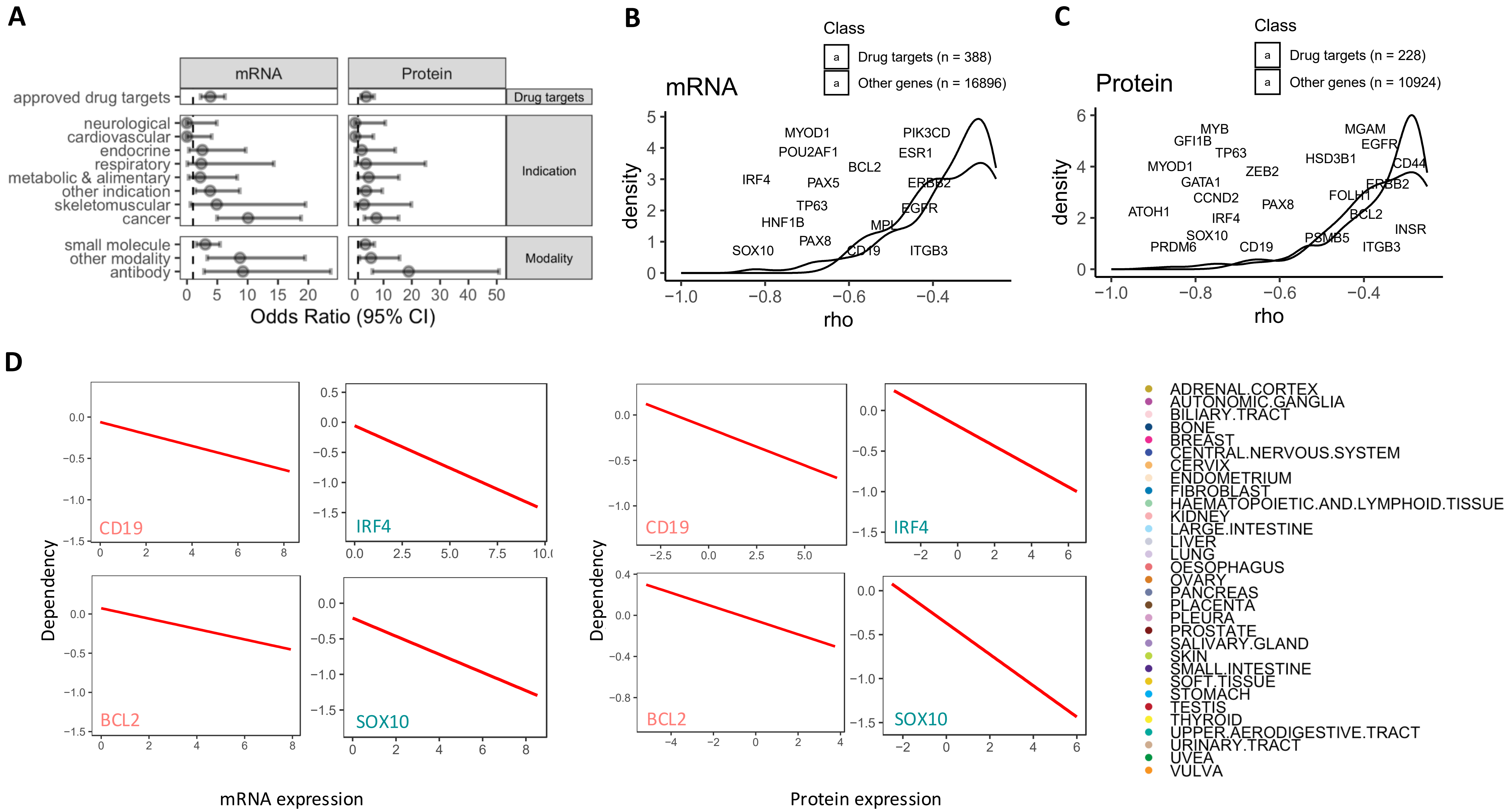

Figure 5. Leveraging Expression-Driven Dependency to Enrich for Drug Targets. (A) Enrichment (Fisher's exact test) results demonstrating the enrichment of identified GEDs and PEDs in druggable gene lists curated by DrugBank, including all approved drug targets, drug targets by indication, and by drug modality. (B-C) The density plots of ED scores from drug targets (DrugBank approved targets) versus other genes, highlighting the top significant targets identified at (B) mRNA and (C) protein levels. (D) Scatter plots of expression vs. dependency correlations for top drug targets and other genes, showing significant pan-lineage ED at both mRNA and protein levels (e.g., SOX10, TP63, IRF4, CCND1). Data points (cell lines) are colored by tissue type.

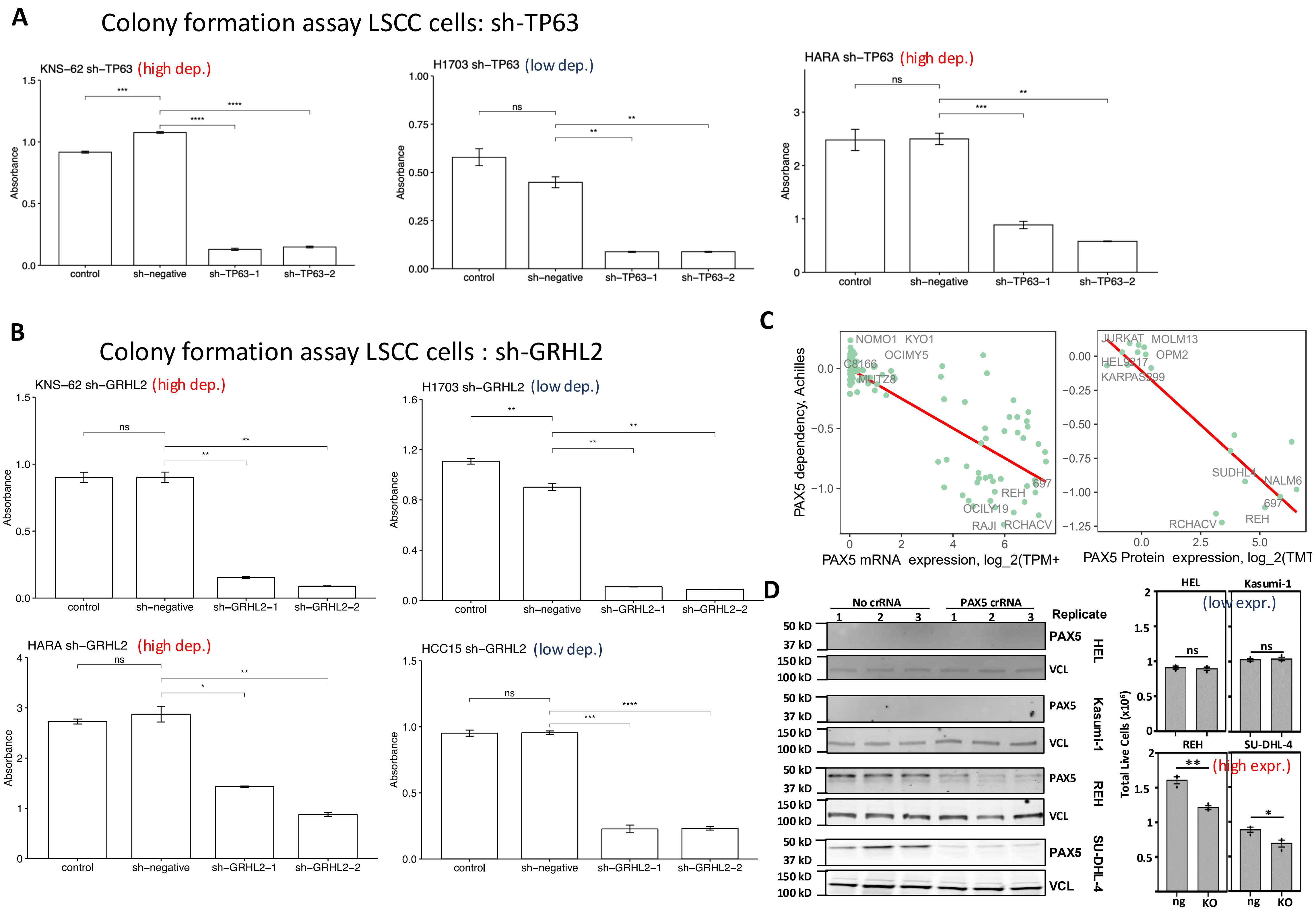

Figure 6. Functional validation of expression-driven dependency targets, TP63, GRHL2, and PAX5, in lung squamous cancer cell and hematopoietic cell lines. (A) Colony formation assay in LSCC cell lines (KNS-62, H1703, and HARA) upon knockdown of TP63 using two shRNA constructs (sh-TP63-1 and sh-TP63-2). Significant reduction in colony formation was observed compared to sh-negative control cells ($p<0.01$). ns: non-significance between control and sh-negative cells. Each experiment was performed with 3 replicate wells, where error bars show mean ± standard deviation; this also applies to *panel B*. (B) Colony formation assay in LSCC cell lines (KNS-62, H1703, HARA, and HCC15) upon knockdown of GRHL2 using two shRNA constructs (sh-GRHL2-1 and sh-GRHL2-2). Significant decrease in colony formation was seen compared to sh-negative control cells ($p<0.01$). (C) PAX5 mRNA and protein expression levels in myeloid (HEL, Kasumi-1) and B-cell (REH, SU-DHL4) lineage cell lines. PAX5 showed lineage-specific expression-driven dependency. (D) Effect of PAX5 knockout (KO) via CRISPR on cell viability in PAX5-high B-cell lines (REH, SU-DHL4) and PAX5-low myeloid lines (HEL, Kasumi-1). PAX5 KO significantly reduced live cell numbers in REH and SU-DHL4 ($p<0.05$ and $p<0.01$, respectively), but not in HEL and Kasumi-1. In (D) left, protein levels were assessed by anti-PAX5 72 hours after electroporation. VCL serves as a loading control. In (D) right, cells were electroporated with RNP complexes with (KO) or without (ng) PAX5 crRNA and allowed to recover for 72 hours. After recovery ng and KO cells were reseeded at equal densities and live cells were counted by trypan blue exclusion after 72 hours. Cells were counted in technical triplicate for each biological replicate (n=3).

Supplementary Figures

Click here to access/download
Supplementary Material
expression_dependency_figures_supp.pdf

Supplementary Table

Click here to access/download
Supplementary Material
TableS1.xlsx

Supplementary Table

Click here to access/download
Supplementary Material
TableS2.xlsx

Supplementary Table

Click here to access/download
Supplementary Material
TableS3.xlsx

Supplementary Table

Click here to access/download
Supplementary Material
TableS4.xlsx

Supplementary Table

Click here to access/download
Supplementary Material
TableS5.xlsx

Supplementary Table

Click here to access/download
Supplementary Material
TableS6.xlsx

Supplementary Table

Click here to access/download
Supplementary Material
TableS7.xlsx

Supplementary Table

Click here to access/download
Supplementary Material
TableS8.xlsx

Supplementary Table

Click here to access/download
Supplementary Material
TableS9.xlsx

Supplementary Table

Click here to access/download
Supplementary Material
TableS10.xlsx

Supplementary Table

Click here to access/download
Supplementary Material
TableS11.xlsx

Supplementary Table

Click here to access/download
Supplementary Material
TableS12.xlsx

Supplementary Table

Click here to access/download
Supplementary Material
TableS13.xlsx

Supplementary Table

Click here to access/download
Supplementary Material
TableS14.xlsx

Tracked changes manuscript for peer review

Click here to access/download
Supplementary Material
Expression_Dependency_Manuscript_revised_tracked.docx

Icahn School of Medicine at Mount Sinai

Kuan-lin Huang, PhD
Associate Professor
Department of Genetics and Genomic Sciences
Department of Artificial Intelligence and Human Health
Center for Transformative Disease Modelling
Icahn School of Medicine at Mount Sinai

1399 Park Avenue (Room 4-420C)
Box 1498
New York, NY 10029
Phone: (212) 824-6134
Email: kuan-lin.huang@mssm.edu
Web: ComputationalOmicsLab.org

Sep 29th 2025

Qing Lan, PhD
GigaScience

Dear Dr. Qing Lan,

We are pleased to submit our revised manuscript entitled "**Expression-Driven Genetic Dependency Reveals Targets for Precision Oncology**", along with a point-by-point response to the reviewers' comments.

Following your suggestion and the reviewers' comments, we have conducted a multitude of analyses that have significantly strengthened the manuscript. Key improvements include:

1. **Rigorous benchmarking**: Head-to-head comparisons of BEACON vs. Pearson/Spearman on DepMap real data using DGIdb druggable genes and Project DRIVE-identified TFs, showing consistent AUPRC gains—especially in small, noisy lineages.
2. **Expanded simulations**: Higher-replicate simulations across sample sizes and noise regimes, demonstrating BEACON's accuracy and calibration advantages in small-N and ≥50% noise.
3. **Validation & reproducibility**: Consolidated TP63/GRHL2/PAX5 results with qPCR and full replicates; clarified figures/captions; standardized N and error bars; and upgraded code for reproducibility (relative paths, fixed seeds, added functions, clearer "rho" outputs, documented environment/runtime).

Additionally, we edited the manuscript for clarity and expanded the discussion (e.g., CYCLOPS-type positives, lineage subtypes, and translational considerations). We have also registered at bio.tools (RRID: SCR_027484) in response to your editorial request and cited that accordingly. We believe the revision addresses all concerns and is suitable for publication.

Sincerely and on behalf of the team,

Kuan-lin Huang, Ph.D.

Icahn School of Medicine at Mount Sinai

Kuan-lin Huang, PhD
Associate Professor
Department of Genetics and Genomic Sciences
Department of Artificial Intelligence and Human Health
Center for Transformative Disease Modelling
Icahn School of Medicine at Mount Sinai

1399 Park Avenue (Room 4-420C)
Box 1498
New York, NY 10029
Phone: (212) 824-6134
Email: kuan-lin.huang@mssm.edu
Web: ComputationalOmicsLab.org

Associate Professor of Genetics and Genomic Sciences & Artificial Intelligence and Human Health
Icahn School of Medicine at Mount Sinai
New York, NY 10029

Authors: We sincerely thank the reviewers for the thoughtful and constructive feedback on our manuscript entitled *"Expression-Driven Genetic Dependency Reveals Targets for Precision Oncology" (GIGA-D-25-00147)*. We have carefully considered the comments, particularly regarding the need to benchmark BEACON against established correlation-based approaches and demonstrate its improvements in real data, and have conducted additional systematic benchmarking analyses to address these concerns. We are pleased to submit a revised version of the manuscript for your consideration. Below is a point-by-point response to the reviewers' comments.

Reviewer #1: The authors present BEACON, a method for identifying associations between the expression of a gene and sensitivity to the CRISPR knockout of that gene across a panel of cancer cell lines. These 'oncogene like' dependencies represent potential therapeutic targets that might be exploited for the development of new precision medicines in cancer. The issue that BEACON aims to address is the limited sample size (cell line count) in some specific cancer lineages and experimental noise that might result in spurious correlations between expression and CRISPR sensitivity. The authors demonstrate, using a modelling approach, that BEACON is more reliable for estimating correlation than simple Pearson's correlation when there is high-noise in the measurements. The majority of the manuscript focuses on analyses of dependencies systematically identified using the BEACON approach and their enrichment in drug targets and biological pathways. There is some experimental testing of three potential expression driven dependencies presented. The rationale for the overall approach and analyses are clear.

Authors: We sincerely thank the reviewer for their thoughtful and constructive evaluation of our manuscript. In response, we expanded our benchmarking analyses and show that BEACON consistently outperforms Pearson and Spearman correlations in identifying known druggable targets using real data, particularly in smaller and noisier lineages. We also clarified and consolidated our experimental validation results to ensure transparency.

Major comments

- Previous efforts have systematically associated gene/protein expression with CRISPR sensitivity across the same or related datasets (e.g. Pacini et al, Cancer Cell 2024 and Rohde et al, Molecular Systems Biology 2025 using CRISPR; McDonald et al, Cell 2017 and Tsherniak et al, Cell 2017 using RNAi) and so the primary contribution of this paper can be considered the development of the BEACON method. It is thus somewhat surprising that there is no real assessment of the improvements offered by BEACON when compared to simpler methods (Pearson correlation, Spearman correlation) or more more complex recent approaches (Rohde et al's BACON approach). The modelling approach suggests some improvements in specific circumstances (especially high noise) but it is not clear that this leads to improved dependency identification in the real data. Does BEACON identify known oncogene addictions better than these methods? Are the associations identified more reproducible (e.g. across alternative CRISPR screens or RNAi screens)?

Authors: We thank the reviewer for this validate suggestion; we validated BEACON against real data and incorporated these results in page 5-6:

To further validate BEACON on real data, we systematically benchmarked its performance against Pearson and Spearman correlations to identify a curated set of 2,993 druggable genes from DGIdb as the reference standard using this DepMap CRISPR dataset. andard. For each cancer lineage, we calculated the area under the precision–recall curve (AUPRC) for identifying DGIdb genes based on expression–dependency correlation scores. On average across all lineages, BEACON achieved an AUPRC improvement of 25/29% over Pearson and 23/29% over Spearman correlations (Figure S3), based on CRISPR vs. mRNA/protein expression data. Specifically, BEACON was the top-performing method in 17 of 24 lineages for GED (mRNA) and in 10 of 17 lineages for PED (protein) (Figure S3). The advantage was particularly pronounced in lineages with smaller sample sizes (e.g., Cervix, Oesophagus, Stomach, Endometrium, etc.), where AUPRC gains reached more than two-fold over Pearson/Spearman. An additional benchmarking against the 57 prioritized genes identified by Project DRIVE's expression–dependency model (Pearson-based)[29] showed that BEACON achieved average AUPRC gains of 94/530% over Pearson and 123/616% over Spearman correlation (Figure S3). BEACON was also the top-performing method in 18 of 24 lineages for GED (mRNA) and in 11 of 17 lineages for PED (protein). These results demonstrate that BEACON improves over simpler correlation measures and enhances the recovery of biologically validated dependencies.

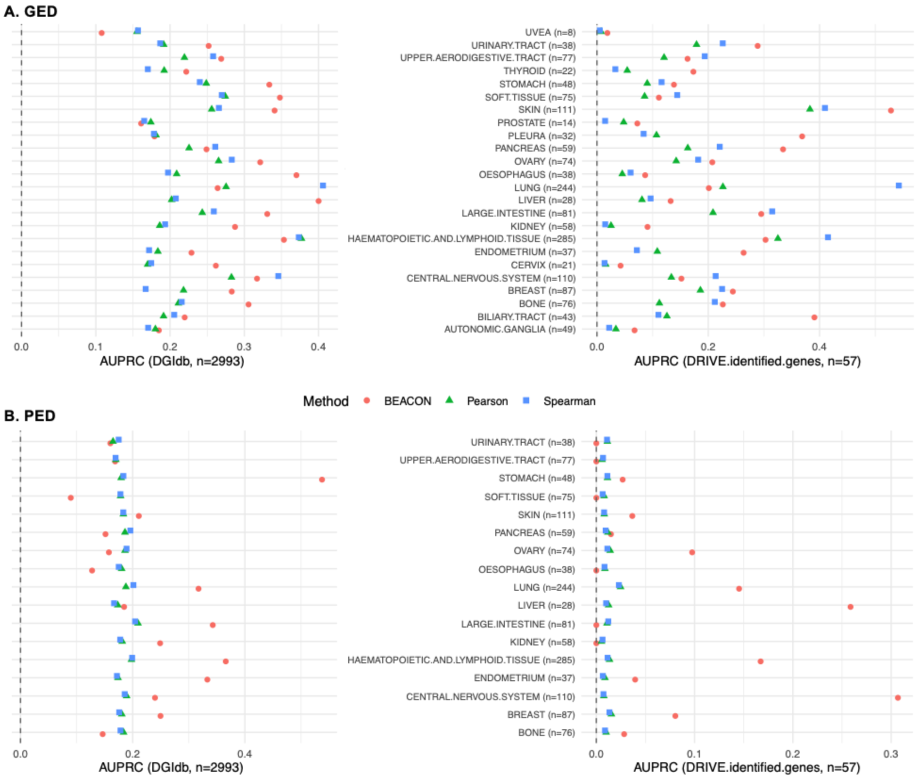

Figure S3. Systematic benchmarking of BEACON against Pearson and Spearman correlations in DepMap data.
(A) AUPRC values for identifying DGIdb druggable genes (n = 2,993) (left) and the transcription factors previously identified in Project DRIVE (n = 57) (right) based on gene expression–dependency (GED) associations across 23 cancer lineages.
(B) AUPRC values for identifying the same druggable gene sets in *panel A* based on protein expression–dependency (PED) associations across 17 lineages.

- The experimental validation and the conclusions drawn from it are somewhat confusing. The authors assess three potential expression associated dependencies - two pan-cancer dependencies (GRHL2 and TP63) and one lineage specific dependency (PAX5 in myeloid cells). Only the lineage-specific dependency validated in the way that might be expected, with higher expression associated with increased dependency, leading the authors to conclude that lineage-specific dependencies may be more suitable targets than pan-cancer ones. Given the numbers analysed (3 genes) this suggestion is not well supported. Moreover the perturbation was performed using distinct approaches - CRISPR for PAX5 and shRNA for the other two genes - and only the knockdown of PAX5 was validated by Western blot. It is very hard to know what phenotypes might be a false positive from off-target shRNA effects or false-negatives from variable shRNA knockdown of the target. The results in S5C suggest that the two shRNAs for each gene cause somewhat discordant phenotypes, suggesting there may be some issues with knockdown efficiency. This could potentially be addressed by adding additional shRNAs for GRHL2 / TP63 or testing them using CRISPR perturbation as was done for PAX5. Validation of the knockdown of the intended target could also shed some light here. The manuscript also mentions experiments in an additional cell line (HCC15) but I cannot see these results presented in the main figures or supplement. It would be useful if all results for these two genes were presented in a single figure, with high and low expressing cell lines clearly marked,

Authors: Our rationale was to evaluate both pan-cancer and lineage-specific dependencies, as BEACON identifies candidates at both levels. While GRHL2 and TP63 appeared as pan-cancer GEDs, their protein-level PED associations were most pronounced in lung cancer, which led us to test them in lung cancer cell lines, whereas PAX5 was pursued as a clear lineage-specific dependency in hematopoietic and lymphoid cells. We acknowledge that the use of different perturbation methods—CRISPR for PAX5 and shRNA for GRHL2 and TP63—complicates direct comparison, but this choice was driven by feasibility and resources with the two collaborative teams at Vanderbilt and Gunma University who specialized in lymphoid and lung cancers, respectively. We also recognize the variability in shRNA efficacy and for transparency have added this to results:

> **To confirm inhibition of the target genes, qPCR validation in HARA cells showed that knockdown efficiency in the HARA cell line where the expression levels of TP63 and GRHL2 were reduced to 33/17% and 41/68%, respectively.**

C

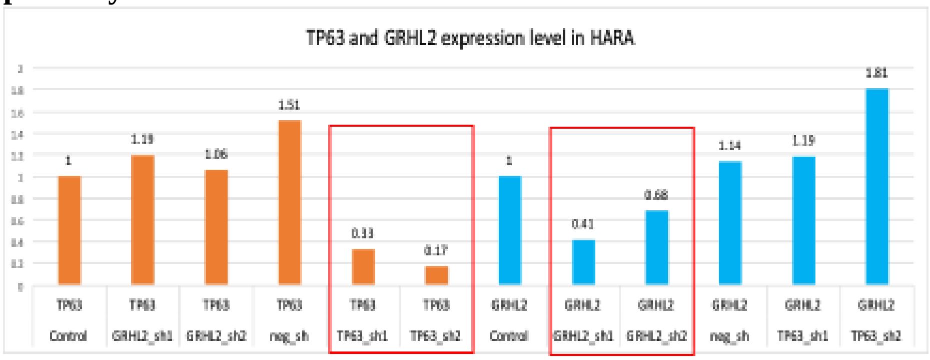

We now also present all replicate results (including HCC15 and HARA) in a single consolidated figure (Figure 6B) with high- and low-dependency cell lines clearly labeled so that the data can be interpreted transparently. Finally, we added a caveat at the end of this Result section:

> **However, given the limited scope of our validation of three targets, a more systematic validation of GED/PED targets will be required to determine the effectiveness of this target prioritization approach.**

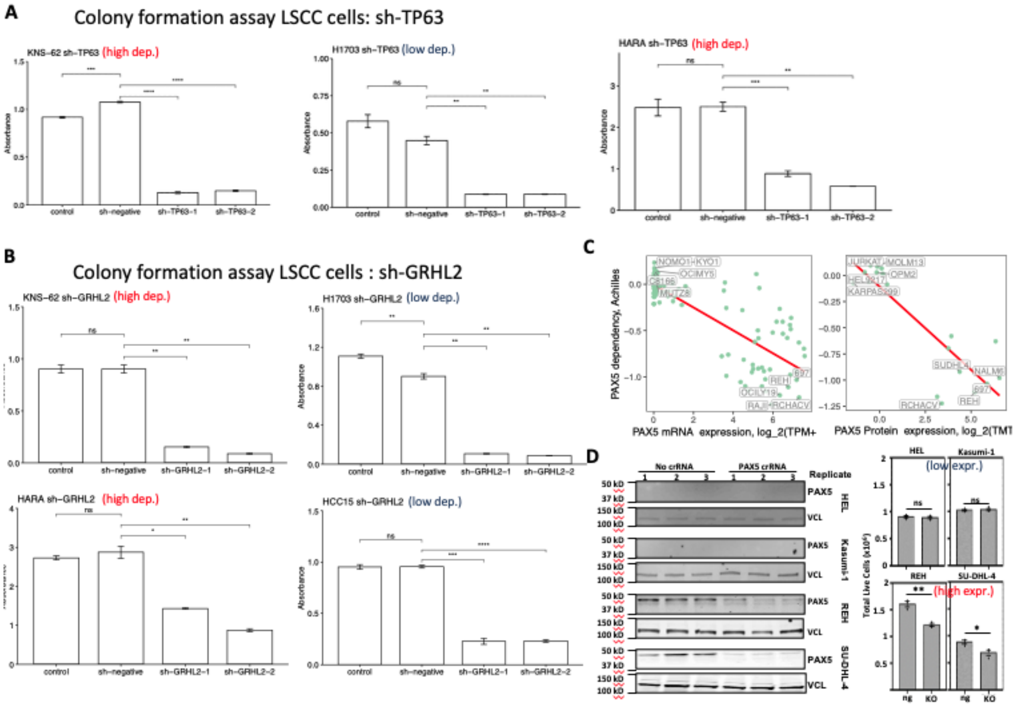

Minor:

- Previous work has established that in some cases lower expression of a gene can make cells more vulnerable to its perturbation (CYCLOPS genes, Nijhawan et al, Cell 2012). While these are not the focus of this manuscript, it would be useful for the authors to comment on the utility of BEACON for their identification.

Authors: We appreciate this helpful suggestion. Indeed, BEACON can in principle identify not only "negative" correlations (with negative rho, due to dependency score being negative) between expression and dependency (GED/PED, where higher expression is associated with stronger dependency) but also "positive" correlations, such as those characteristic of CYCLOPS genes, where lower expression increases vulnerability to perturbation. We have added a brief comment in the Discussion (page 15, lines 428-432):

> **Although in this study we emphasized negative associations where higher target expression corresponds to greater dependency, the Bayesian framework is symmetric and can also detect positive correlations. This makes BEACON suitable for identifying CYCLOPS-type genes, where reduced expression confers greater vulnerability to perturbation[42].**

- p14 "Moreover, GED/PED targets were depleted of genes that were Essential In Culture" - it's not clear what this means or where the data comes from. By definition the gene set analysed are at least somewhat essential in culture

Authors: The "Essential In Culture" gene set was as defined by Hart et al. 2017. We have revised the text to clarify this point and now state (page 12, lines 338-342):

> **Moreover, GED/PED targets were underrepresented among the common essential genes (N=684 "Essential In Culture" genes based on 17 genome-wide CRISPR screens[38]), suggesting that BEACON identifies cell-specific vulnerabilities rather than dependencies universally required for cell viability (e.g., house-keeping genes) that could lead to off-target effects.**

*Reviewer #2: *The authors introduce BEACON, a Bayesian correlation approach designed to identify expression-driven dependency in cancer. Their hypothesis suggests that cancer cells with elevated expression of specific genes demonstrate increased vulnerability to the knockout of those same genes, thereby unveiling a promising new category of targets in precision oncology—particularly valuable for targeting cancer cells lacking druggable mutations.*
**BEACON models expression levels and dependency scores as bivariate Gaussians and employs Markov Chain Monte Carlo (MCMC) sampling to estimate the correlation coefficient between them. They then compute p-values followed by rigorous multiple testing correction (BH based FDR correction).*
**A notable strength of their approach lies in the integration of mass spectrometry proteomics data alongside transcriptomic and perturbation screening data, enhancing the robustness of their findings.*
**Their work highlights some key insights:*
- Gene expression-driven dependency (GED) candidates identified across lineages demonstrate enrichment for "DNA-binding transcription activator activity" and "DNA-binding transcription activator activity, RNA polymerase II-specific" pathways.
- The analysis successfully identifies compelling candidates with robust signals in both GED and PED (FERMT2, GRHL2, KLF5, CDK6, and CCND1), which are well-supported by existing drug evidence or established literature
- Clustering analyses reveal that cancer cells from pancreas and biliary tract tissues, as well as kidney and urinary tract tissue lineages, exhibit remarkably similar expression-driven dependency profiles. Additionally, lineage-specific genes such as transcription factors, cluster together in a manner consistent with existing literature
- Through Fisher's exact test, the authors demonstrate significant enrichments of druggable gene lists from DrugBank with expression-driven dependency patterns at both proteomic and transcriptomic levels
- Experimental validation shows that PAX5 is essential for PAX5-high B cell lymphoma cell growth, while TP63 and GRHL2 are essential for LSCC cell growth.

However, I have several principal concerns about the study that should be addressed to demonstrate the robust and superior performance of this proposed approach.

Authors: We thank the reviewer for their thorough summary and recognition of BEACON's strengths. To address the principal concerns, we expanded our benchmarking

analyses to systematically compare BEACON with Pearson and Spearman correlations in real data to identify druggable genes, clarified the validation experiments for *TP63* and GRHL2, and refined methodological descriptions. These revisions reinforce that BEACON provides robust and practical improvements over existing approaches for uncovering expression-driven dependencies.

Major Comments:

1.Quantitative benchmarking: While the authors present a valuable contribution, the concept of correlating gene dependency scores to expression has been explored previously through approaches like Project DRIVE (E. Robert McDonald, III et al.) and APSIC (Montazeri et al.). BEACON demonstrates strong correlations across multiple lineages, representing broader scope compared to existing methods that appear more lineage-restricted. However, establishing BEACON's comparative advantages requires more rigorous evaluation. Notably, Project DRIVE—a foundational paper in this field—already identified several BEACON candidates in their "Expression Correlation Analysis Identifies Oncogenes and Lineage-Specific Transcription Factors" section, while APSIC characterized many lineage-specific discoveries as tumor effector genes. BEACON's strength lies in integrating proteomic data with transcriptomic and perturbation screens, enabling identification of additional candidates like PAX5 for hematopoietic and lymphoid tissue. To demonstrate the method's impact, I recommend systematic quantitative benchmarking against existing approaches.
Authors: We systematically compared BEACON GED/PEDs with those obtained from other approaches, including Pearson correlation (as used in Project DRIVE and BACON), and Spearman correlation (as additionally implemented in BACON). APSiC was build to identify new drivers via analyzing perturbation (shRNA) vs. mutation/CNVs, quoting https://academic.oup.com/nar/article/49/15/8488/6329117 "We considered the knockdown experiments of p genes across N cell lines. Let be viability of cell line upon knocking down gene and be a binary variable indicating whether a specific genetic alteration (i.e. mutation or copy number alteration) is present in gene j of cell line i." Thus we did not benchmark against it.

Importantly, BEACON utilizes richer/complementary datasets than previous studies. Disentangling contributions of data richness versus methodological innovation would provide valuable insights into whether enhanced performance stems from improved data availability or genuine method improvements.
Authors: To address this, we applied all methods (Pearson, Spearman, and BEACON) to the same dataset consisting of matched mRNA/protein expression and CRISPR dependency data from DepMap. By holding the data constant, differences in performance directly reflect methodological advantages of BEACON rather than data availability.

Overall for benchmarking, the authors are strongly encouraged to utilize any comprehensive datasets that best demonstrate their method's competitive advantage and are not limited to the specific comparisons recommended above.
Authors: We used the DGIdb druggable gene set (n = 2,993) as a comprehensive and lineage-spanning reference standard. This enabled systematic benchmarking across all available lineages at both the mRNA and protein levels, ensuring that BEACON's

advantages are broadly generalizable and not restricted to a subset of examples (Figure S3). Combining all these points, we have added the following to Results:

> To further validate BEACON on real data, we systematically benchmarked its performance against Pearson and Spearman correlations to identify a curated set of 2,993 druggable genes from DGIdb as the reference standard using this DepMap CRISPR dataset. andard. For each cancer lineage, we calculated the area under the precision–recall curve (AUPRC) for identifying DGIdb genes based on expression–dependency correlation scores. On average across all lineages, BEACON achieved an AUPRC improvement of 25/29% over Pearson and 23/29% over Spearman correlations (Figure S3), based on CRISPR vs. mRNA/protein expression data. Specifically, BEACON was the top-performing method in 17 of 24 lineages for GED (mRNA) and in 10 of 17 lineages for PED (protein) (Figure S3). The advantage was particularly pronounced in lineages with smaller sample sizes (e.g., Cervix, Oesophagus, Stomach, Endometrium, etc.), where AUPRC gains reached more than two-fold over Pearson/Spearman. An additional benchmarking against the 57 prioritized genes identified by Project DRIVE's expression–dependency model (Pearson-based)[29] showed that BEACON achieved average AUPRC gains of 94/530% over Pearson and 123/616% over Spearman correlation (Figure S3). BEACON was also the top-performing method in 18 of 24 lineages for GED (mRNA) and in 11 of 17 lineages for PED (protein). These results demonstrate that BEACON improves over simpler correlation measures and enhances the recovery of biologically validated dependencies.

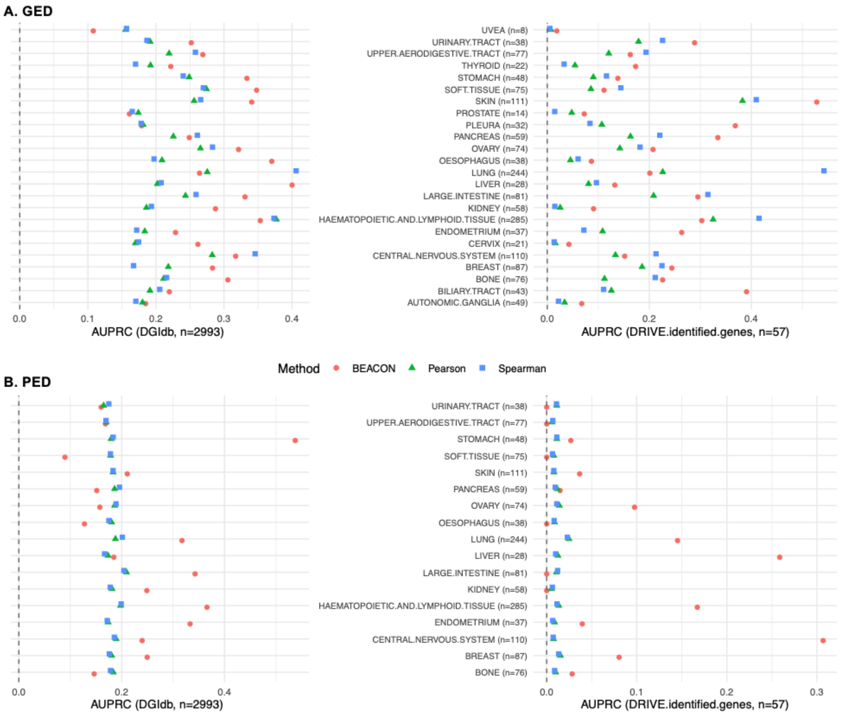

Figure S3. Systematic benchmarking of BEACON against Pearson and Spearman correlations in DepMap data.
(A) AUPRC values for identifying DGIdb druggable genes (n = 2,993) (left) and the transcription factors previously identified in Project DRIVE (n = 57) (right) based on gene expression–dependency (GED) associations across 23 cancer lineages.
(B) AUPRC values for identifying the same druggable gene sets in *panel A* based on protein expression–dependency (PED) associations across 17 lineages.

2.Correlation method comparisons: Figure S2 shows that BEACON exhibits higher MSE at extremes, and the claimed advantage over Pearson for small sample sizes is difficult to quantify from the current visualization. While the theoretical expectation that BEACON should outperform Pearson in small samples is reasonable, the practical significance remains unclear from these simulations. I recommend demonstrating BEACON's advantage using real data by creating a curated list of established GEDs/PEDs and comparing performance between the two methods. This is particularly important since several of BEACON's hits were previously reported by Project DRIVE using simple Pearson correlations.

Authors: As seen in the prior response, we curated two complementary reference sets: (i) DGIdb druggable genes and (ii) the transcription factors previously identified in Project DRIVE. Using AUPRC as the evaluation metric, BEACON consistently outperformed Pearson and Spearman correlations across both benchmarks, at both the GED (mRNA) and PED (protein) levels (Figure S3). The curation of Project DRIVE's gene list was added in addition to DGIdb druggable gene list to resolve this reviewer's concern.

Alternatively, if BEACON's advantage is indeed significant, please elaborate on the simulation results to better justify this claim with clearer quantitative metrics.

Authors: We also expanded our simulation analysis to include additional scenarios with finer resolution at very small sample sizes and under higher noise levels, running 100 replicates for each condition. These extended simulations demonstrate that BEACON consistently yields more accurate and better-calibrated correlation estimates than other methods, with the advantage being most pronounced in small-N settings and at moderate-to-high noise levels (≥50%) (Figure S2).

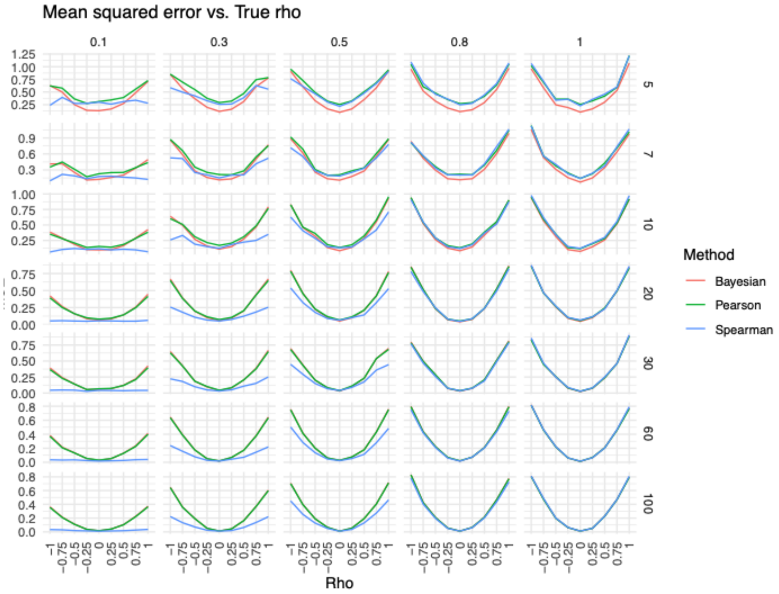

Figure S2. Benchmarking of BEACON against Pearson and Spearman correlations in simulated data. The performance are measured by mean squared-error (MSE, y-axis) for the same data sets randomly simulated for various true correlation levels (rho, x-axis), under different conditions of noise interference (columns, 0.1, 0.3, 0.5, 0.8, 1) and sample size (rows, 5, 7, 10, 20, 30, 60, 100).

3.Validation experiments: I'm seeking clarification on the validation experiments for TP63 and GRHL2. These candidates were not sensitive to predicted dependency and the authors say that "pan-lineage targets may represent universal vulnerability and their inhibition may lead to undesired off-target effects on other cells". Are the authors positioning them as weaker candidates to illustrate the superiority of lineage-specific predictions like PAX5? Additionally, why were different experimental approaches used—CRISPR for PAX5 versus shRNA for TP63 and GRHL2? For a method aimed at identifying druggable targets, would drug based experiments be more relevant than knockdown approaches to better demonstrate clinical applicability?

Authors: We thank the reviewer for raising these important points. GRHL2 and TP63 were selected because, while they appeared as pan-lineage GEDs, their strongest protein-level PED associations were observed in lung cancer, which led us to test them in lung squamous carcinoma cell lines. In contrast, PAX5 was chosen as a clear example of a lineage-specific dependency. We have updated the text at the end of Result section given the limited sets of genes we were able to validate:

> **Thus, proteins showing lineage-specific dependencies may present as suitable precision oncology targets in the subset of tumors overexpressing the target gene and protein. However, given the limited scope of our validation of three targets, a more systematic validation of GED/PED targets will be required to determine the effectiveness of this target prioritization approach.**

Regarding methodology, CRISPR-based validation was used for PAX5 while shRNA was used for GRHL2 and TP63 due to the resources available to the two collaborative teams at Vanderbilt and Gunma University who specialized in lymphoid and lung cancers, respectively.

While drug-based experiments would indeed demonstrate greater clinical applicability, our aim here was to provide proof-of-concept validation of BEACON's target predictions, whereas drug-based validation require different experimental design and introduce other complications that may not correspond with how good the target is (e.g., on-target efficacy and off-target effects of the drugs used).

Minor comments

1. In Figure 4A, the caption refers to the plot as a heatmap, but the visualization appears to be a scatterplot. Please clarify whether the heatmap is missing or modify the caption appropriately. Additionally, I recommend using a different shade of green, as the current color choice makes some gene names difficult to read.

Authors: We thank the reviewer for noting this. Figure 4A is a scatterplot (not a heatmap), and the caption has been corrected. We also adjusted the green color to improve readability of gene names.

2. In Fig S5A, please add a legend for tumor and normal

Authors: We have added a legend to Figure S6A (previously Fig S5A) to clearly distinguish tumor and normal samples.

3. For the TP63 and GRHL2 validation experiments, please include results for all four cell lines. The current manuscript is missing HCC15-shTP63, HCC15-shGRHL2, and HARA-shGRHL2 plots.

Authors: All replicate results are now presented together in a consolidated figure (Figure 6B), including HCC15 and HARA for GRHL2. HCC15 was not used in the TP63 experiments.

4. How many replicates were the experiments performed on? Is it N= 3 for all experiments?

Authors: Each colony formation and proliferation assay was performed with three replicate wells (N=3), as noted in the Methods section and figure legends.

5. Missing some text here - "BEACON offers the unique advantage of utilizing prior distributions that are less susceptible to outliers, especially in multiple lineages where the number of cell lines."

Authors: The sentence has been corrected to read: "BEACON offers the unique advantage of utilizing prior distributions that are less sensitive to outliers, which is particularly beneficial in lineages where the number of available cell lines is small and thus more vulnerable to the influence of outliers."

Reviewer #3: The authors develop a method for correlating gene and protein expression with cellular dependencies using the resources of DepMap. The innovation appears to be a Bayesian approach to the correlation analysis. They use this approach to identify potential therapeutic targets and evaluate some top candidates using in vitro experiments. The paper is fairly straightforward to follow.

Major comments: 1. Benchmarking - given the non-linear relationships shown in Fig 2, is a comparison with the Pearson method the most appropriate? Would a Spearman's be better?

Authors: We thank the reviewer for this helpful suggestion. In addition to Pearson, we have now benchmarked BEACON directly against Spearman correlation. Using both simulated and real datasets (DGIdb druggable genes and transcription factors identified in Project DRIVE). This is added in Results text:

> **We benchmarked BEACON's Bayesian correlation against Pearson correlation, which was used in project DRIVE[29], and against both Pearson and Spearman correlation measures, which were employed in BACON[30]. Simulations were performed on expression and dependency datasets across a range of correlation levels (from -1 to 1, with 0.25 intervals) and sample size (number of cell lines, 5, 7, 10, 20, 30, 60, 100), with different fraction (0.1, 0.3, 0.5, 0.8, 1) of samples corrupted by noise to enable direct comparison of methodological performance (Figure S2). Based on these simulations, we observed that the Bayesian method is better than Pearson correlation for estimating moderate true correlation ($|rho| < 0.75$) in small sample size, and preferable in noisy data (noise level ≥ 0.5, i.e., 50% or more of the samples are corrupted by noise to become outliers), regardless of sample size or true correlation level.**
>
> **To further validate BEACON on real data, we systematically benchmarked its performance against Pearson and Spearman correlations to identify a curated set of 2,993 druggable genes from DGIdb as the reference standard using this DepMap CRISPR dataset. andard. For each cancer lineage, we calculated the area under the precision–recall curve (AUPRC) for identifying DGIdb genes based on expression–dependency correlation scores. On average across all lineages, BEACON achieved an AUPRC improvement of 25/29% over Pearson and 23/29% over Spearman correlations (Figure S3), based on CRISPR vs. mRNA/protein expression data. Specifically, BEACON was the top-performing method in 17 of 24 lineages for GED (mRNA) and in 10 of 17 lineages for PED (protein) (Figure S3). The advantage was particularly pronounced in lineages with smaller sample sizes (e.g., Cervix, Oesophagus, Stomach, Endometrium, etc.), where AUPRC gains reached more than two-fold over Pearson/Spearman. An additional benchmarking against the 57 prioritized genes identified by Project DRIVE's expression–dependency model (Pearson-based)[29] showed that BEACON achieved average AUPRC gains of 94/530% over Pearson and 123/616% over Spearman correlation (Figure S3). BEACON was also the top-performing method in 18 of 24 lineages for GED (mRNA) and in 11 of 17 lineages for PED (protein). These results demonstrate that BEACON improves over**

simpler correlation measures and enhances the recovery of biologically validated dependencies.

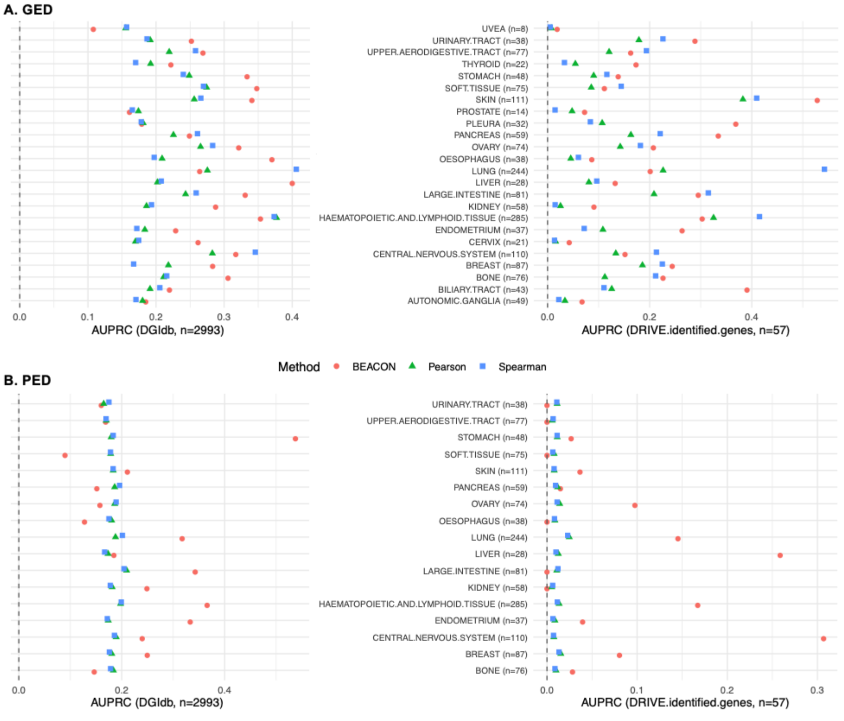

Figure S3. Systematic benchmarking of BEACON against Pearson and Spearman correlations in DepMap data.
(A) AUPRC values for identifying DGIdb druggable genes (n = 2,993) (left) and the transcription factors previously identified in Project DRIVE (n = 57) (right) based on gene expression–dependency (GED) associations across 23 cancer lineages.
(B) AUPRC values for identifying the same druggable gene sets in *panel A* based on protein expression–dependency (PED) associations across 17 lineages.

2. The analysis identifies dependencies that are proposed as therapeutic targets, however while the proteins can be druggable, what about normal tissue effects? Some of these are likely lineage-defining proteins that could be highly expressed in normal tissues. Is is notable that in Fig 5B, C that the existing drug targets have a lower association strength than other GEDs identified. Does this suggest that the strongest correlations might be lineage-crucial genes that are too important for normal tissue function to make good drug targets? This needs further consideration in the discussion. Are there any pathways differences between these groups (known drug targets vs others)? For example you might expect more tissue lineage Tfs in the "other" category, while the approved drug targets perhaps more cell surface receptors.

Authors: We thank the reviewer for bringing up this very interesting point. As our pathway analyses revealed, some of the strongest GED/PED associations are enriched for DNA-binding transcription activator (Figure 2D, 3D) that are transcription factors (TFs). In our knowledge, many disease-associated TFs have not been drugged not due to their potential lineage essentiality in adults, but also due to their lack of natural binding pockets and complex protein-DNA or protein-protein interactions. Thus, how druggable and effective they may serve as drug targets remains less tested than other protein families more amenable to prior small molecule/antibody-based approaches. Meanwhile, the DrugBank list is known to be enriched for enzymes, transporters, receptors (https://www.proteinatlas.org/humanproteome/tissue/druggable). We have added to the end of this section:

> **However, many of these top candidates may have more extreme GED/PED rho values than currently druggable genes; it remains to be tested whether that is a confounding with the protein classes more amenable to current drug modalities or there may be a more desirable GED/PED window for prioritizing therapeutic targets.**

We have also added text to the Discussion (underlined texts are the additions):

> **Many GED/PED gene targets are lineage-specific transcription factors (TFs); these agree with recent single-cell studies and synthesis that posited the "developmental constraint model of cancer cell states", which cancer cell states correspond to and may be constrained by the landscape of "developmental map"[46]. Thus, a cancer cell adopting a specific developmental state may require activation of such transcription factors and become genetically dependent. Traditionally, TFs were not easily addressable using small molecule or antibody-based approaches due to their lack of binding pockets and complex intermolecular interactions. While such targets used to be considered undruggable, new drug modalities such as proteolysis-targeting chimera (PROTAC) are showing promises[43,47-50], particularly in cases where there may be a sufficient therapeutic window in inhibiting these TFs, e.g., to treat adult tumors where the target TFs were only essential in early development and in tumor cells.**

3. The cell assays performed should effectively be replicating the results of the dependencies on which BEACON is based (DepMap), so why do you get different results? Is it because of the different methods used ie shRNA (not seeing the correlation between expression and dependency) vs CRISPR (replicating the correlation)? If you look at older DepMap scores when they used knockdown rather than CRISPR can you replicate your results?

Authors: The discrepancies between our validation assays and DepMap dependency scores reflect both methodological differences and phenotypic readouts. DepMap scores are derived from pooled CRISPR or shRNA screens after many cell passages and barcode sequencing from pooled cell populations, where cell viability is inferred indirectly through barcode representation after cross-gene normalization. Thus, analyses of Broad and Sanger's large-scale CRISPR knockout screens show correlation but indeed variations of the derived gene score (PMID: 31862961). By contrast, our colony formation assays in lung cancer cells capture proliferative potential, integrating effects on cell survival, division, and stress responses. Similarly, our live cell counts in PAX5 B-cell experiments reflect more acute changes in growth kinetics, which may yield different sensitivity profiles. In our focused experiments, we were also able to conduct qPCR and western blots to validate knockdown/knockout efficiency. Thus, while the broad correlation with DepMap is informative, focused assays provide complementary, context-specific insights into how genetic perturbations affect distinct cellular outcomes.

We have added briefly to the Results, "These focused experiments provide complementary validation to the functional data from the DepMap screen because, in our experiments, all

functional readouts were collected within four days of the perturbation (Methods), whereas the DepMap CRISPR screen are performed at 14-21 days and scores are inferred indirectly through barcode representation after cross-gene normalization."

We also thank the reviewer for the suggestion to examine DepMap's RNAi data, albeit this screen had a documentation of even further passaging times. In general, DepMap's RNAi dependency & CRISPR dependencies for these assayed genes agree well in these tested cell lines. Based on this result, we added, "We further queried DepMap RNAi data (passaged for 16 doublings, up to 40 days)[5] for these genes that showed consistent dependencies with the original DepMap-based predictions (Figure S7), suggesting potential variations due to shRNA constructs or experimental durations."

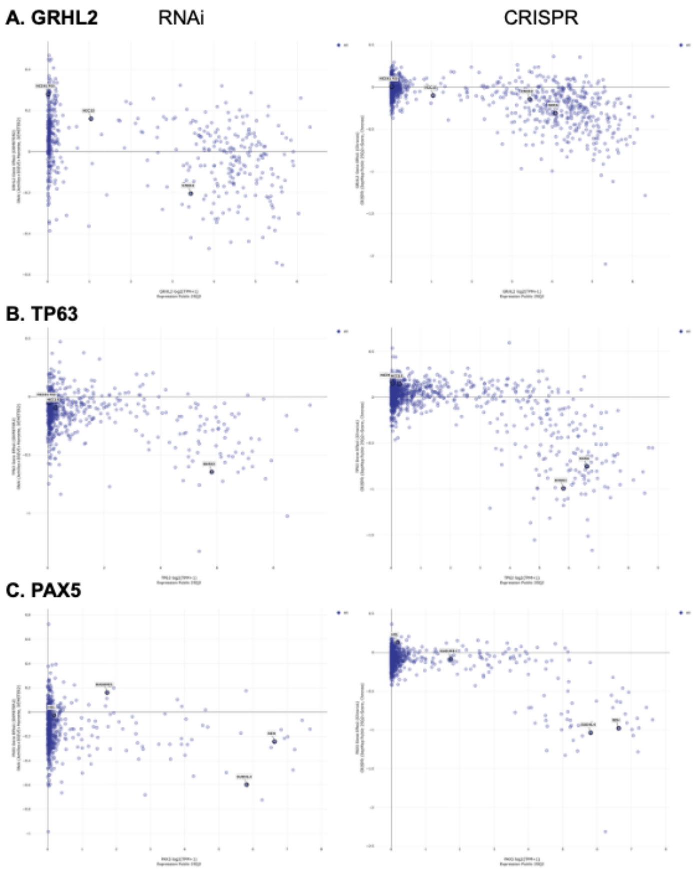

Figure S7. Dependency scores vs. gene expression levels of (A) *GRHL2*, (B) *TP63*, and (C) *PAX5* as provided by DepMap RNAi and CRISPR screen data across all cell lines with available data (public 25Q2 data release). The x-axis denotes normalized gene expression log2(TPM+1) of the respective genes and the y-axis denoted the gene knockdown effect from RNAi (DEMETER2 score) (left panels) or the gene knockout effect from CRISPR (Cronos score) (right panels) screens. The cell types that were used in our functional experiments (Figure 6) were labeled.

4. Although mycoplasma testing was done, were the cell lines re-authenticated by STR profiling at any point?

Authors: All cell lines used in this study were obtained from authenticated biobanks (as listed in Table S15), and mycoplasma testing was performed regularly. The Gunman team member (Dr. Reika) who provided the cell lines for lung cancer experiments had left for industry a few years ago and we did not receive a confirmation on this, and thus we did not change texts there.
The Vanderbilt team confirmed all lines used for the PAX5 experiments were confirmed with STR profiling and we have added accordingly to that section of the Methods.

5. QPCR is mentioned n the methods but not provided in the results that I can find. Did this validate gene knockdown by shRNA? Any correlation between % KD and proliferation/colony forming effect?

Authors: qPCR was performed to confirm knockdown of TP63 and GRHL2 following shRNA treatment in the HARA cell lines. We also recognize the variability in shRNA efficacy and for transparency have added this to results:

To confirm inhibition of the target genes, qPCR validation in HARA cells showed that knockdown efficiency in the HARA cell line where the expression levels of *TP63* and *GRHL2* were reduced to 33/17% and 41/68%, respectively.

C

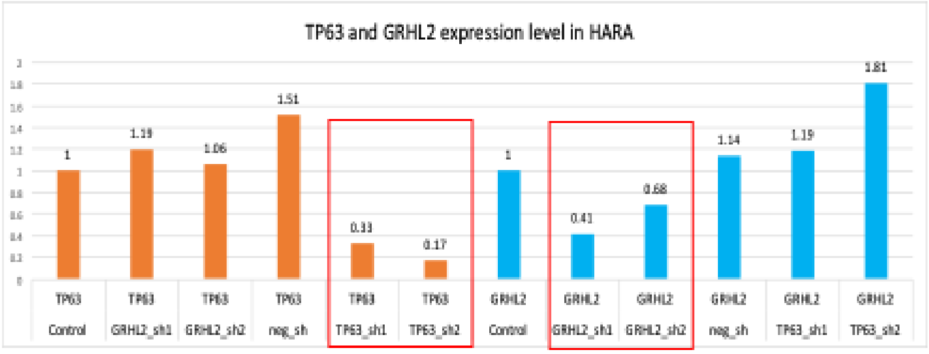

In HARA, the more efficacious sh-TP63-2 showed trends of higher reduction in colony formation than sh-TP63-1, but vice versa for the GRHL2 shRNAs. In all cases the knockdowns were significantly effective in reducing colony formation. We did not describe the potential correlation between % KD and proliferation/colony forming effect as qPCR was only conducted in HARA but not other cell lines.

6. In the discussion it should be acknowledged that cancer subtypes exist within lineages that are molecularly and clinically distinct and so the method might be missing targets specific for these eg ER+ and ER- breast cancer.

Authors: We agree with the reviewer that molecular and clinical subtypes within a lineage (e.g., ER^+ vs. ER^- breast cancer) may harbor distinct dependencies that could be missed when analyzing at the lineage level. We have added a note in the Discussion:

> **It is also important to note that within a given lineage, molecular and clinical subtypes (e.g., ER^+ vs. ER^- breast cancer) may harbor distinct dependencies that could be masked when analyzing at the lineage level. Applying BEACON to subtype-stratified datasets may therefore reveal additional, clinically relevant vulnerabilities. As larger and better-annotated datasets become available, this represents an important direction for future work.**

Minor comments:
1. Results para 1 "especially in multiple lineages where the number of cell lines." Missing something in this sentence?

Authors: The sentence has been corrected to read: "BEACON offers the unique advantage of utilizing prior distributions that are less sensitive to outliers, which is particularly beneficial in lineages where the number of available cell lines is small and thus more vulnerable to the influence of outliers."

2. Needs some grammar review

Authors: We have carefully reviewed the manuscript for grammar and style and have corrected the relevant sentences for clarity and readability throughout.

3, Please italicise all gene names (when referring to gene, not protein) eg CCNE1 amplification etc

Authors: All gene symbols have now been italicized throughout the manuscript when referring to genes (e.g., *CCNE1*-amplified), in accordance with standard nomenclature conventions.

4. Fig S5A - legend or axis labels for N and T needed.

Authors: The axis labels "Tumor" and "Normal" has now been added to clearly distinguish those samples in the figure. With the inclusion of an additional panel, this figure is now presented as Figure S6A in the revised manuscript.

5. Fig S5C, D - these are proliferation not colony forming assays as stated in the text.

Authors: The text has been revised to accurately describe Figures S6C and S6D as proliferation assays rather than colony-forming assays. The Results section now states: *"In KNS-62 and H1703 LSCC cells, the knockdown of TP63 using two shRNA constructs (sh-TP63-1 and sh-TP63-2) resulted in a significant reduction in colony formation and cell viability (reduced proliferation) compared to controls(p<0.01) (Figure 6A, Figure S6D)."*

6. Please include number of replicates and type of error bars in figure legends for cell assays

Authors: The figure legends for all cell-based assays have been updated to include the number of replicates (N = 3) and the type of error bars (standard deviation).

Reviewer #4: Reproducibility report

1. Summary of the Study The authors developed a Bayesian method called BEACON to integrate multi-omics data. The method was tested on cancer cell lines across 17 tissue types to identify expression- driven dependencies. The method recovered known drug targets and identified novel candidates. The study concludes this method provides a systematic approach to identify precision oncology targets.

2. Scope of reproducibility According to our assessment the primary objective is: to identify expression-driven dependencies across cancer cell lines from multiple lineages enabling the discovery of genes whose expression levels correlate with cancer cell dependency scores. – Outcome: Identification of genes with significant expression-driven dependencies across pan-lineage cancer cell lines.
- Analysis method outcome: "BEACON calculated the Bayesian correlation between the gene's expressions and CERES cancer dependency scores 25 across the pan-lineage cell lines.

BEACON modeled expression levels and dependency scores as the bivariate Gaussians and used Markov Chain Monte Carlo (MCMC) sampling to estimate the correlation coefficient rho between them. Given the null hypothesis that the uncorrelated expression and dependency of a gene has the 0 rho coefficient, we statistically tested each gene's rho estimate obtained from the MCMC simulation as follows. Assume that the MCMC sampling is carried out for a null gene's expression and dependency, then we expect that the distribution of the rho estimate accumulated over the MCMC iterations will be centered at zero. Based on this rationale, we computed the z-score of i-th gene as the deviation of the MCMC estimate of rho from the expected (null) value (i.e., zero) in terms of the standard deviation observed in the simulated distribution, i.e., z(i) = rhoMCMC(i) / SDMCMC(i). Since the z-values, by nature, follow a normal distribution with zero-mean and unit-variance, then we computed the p- value for each gene's rho estimate as the probability of observing a value as extreme as the computed z-value for that gene. We multi-testing corrected the resulting p-values using the BH procedure for FDR." (page 19 -Methods section / mRNA expression-driven dependency (GED))
- Main result: "We first analyzed the pan-lineage GED by using mRNA levels and the corresponding dependency scores from 854 cell lines with available data across 17 lineages and identified 244 genes showing significant association (correlation coefficient, rho < -0.25, FDR < 0.05)" (page 7 - Results section / Cancer vulnerability targets showing gene expression-driven dependency (GED))

3. Availability of Materials
a. Data
- Data availability: Open
- Data completeness: Complete, all data necessary to reproduce main results are available.
- Access Method: Repository
- Repository: https://urldefense.proofpoint.com/v2/url?u=https-3A__doi.org_10.6084_m9.figshare.19700056.v2&d=DwIBaQ&c=shNJtf5dKgNcPZ6Yh64b-ALLUrcfR-4CCQkZVKC8w3o&r=88-dBITsh8vXfnQjNN0pRGpahxI_Sccu4B-wNY_gsU4&m=QnwsYAvlqsJ9rZbiozGbBdLREQ59Pq0O2jitd0FjFA1KqZuHTZDAkiy8-tAbmKGL&s=SNkoITd2TWtWYm6mVW11wfiv2qKBRp4piBeZy4Er5Wo&e=
-Data quality: Structured

b. Code
- Code availability: Open
- Programming Language(s): R
- Repository link: https://urldefense.proofpoint.com/v2/url?u=https-3A__github.com_Huang-2Dlab_BEACON&d=DwIBaQ&c=shNJtf5dKgNcPZ6Yh64b-ALLUrcfR-4CCQkZVKC8w3o&r=88-dBITsh8vXfnQjNN0pRGpahxI_Sccu4B-wNY_gsU4&m=QnwsYAvlqsJ9rZbiozGbBdLREQ59Pq0O2jitd0FjFA1KqZuHTZDAkiy8-tAbmKGL&s=ly6kaHNqm3UtC0ImX6NAlr4fBkJv4CgaN3uAfeBNmIE&e= - License: MIT license
- Repository status: Public
- Documentation: Readme file

4. Computational environment of reproduction analysis
- Operating system for reproduction: MacOS 15.5

- Programming Language(s): R
- Code implementation approach: Using shared code
- Version environment for reproduction: R version 4.5.0/RStudio 2025.05.1

5. Results
5.1 Original study results
- Results 1: Supplementary table S2 5.2 Steps for reproduction
-> Run the code PanLineageMCMC.R
- Issue 1: File import paths and incorrect file name
-- Resolved: In the original code, there were fixed file paths that only worked on one specific computer. This caused problems when running the code on other computers. To fix this, I recommended to use relative paths, which are based on where the script is located. This way, the code can be run on any computer without needing to change the paths each time.

```
------------------ Start of script ------------------
sam.dep = read.csv(file.path(getwd(), "DepMap_data",
"sample_info.csv"))
------------------- End of script -------------------
```

- Issue 2: Missing function "intsect" at line 162
-- Resolved: The script called a function intsect that was not defined, leading to an error. Upon request, the authors provided the missing function and added it to the main script (PanLineageMCMC.R).

- Issue 3: Output directory not created.
-- Resolved: The script attempted to write output files to a directory that was not created beforehand. This caused errors during the loop execution when trying to save results. A directory check and automatic creation script was added. If the output folder does not exist, it is now created automatically before the loop runs.

```
------------------ Start of script ------------------
dir_path <-
paste0('../out/jags.nadapt',n.adapt,'.update',n.update,'.mcmc
',n.iter,'.simulation_SD_22Q2')
if (!dir.exists(dir_path)) {
  dir.create(dir_path, recursive = TRUE)
}
------------------- End of script -------------------
```

5.3 Statistical comparison Original vs Reproduced results
- Results: Table.mRNA.dependency.Bayesian.pancancer file attached
- Comments: The Bayesian PanCancer analysis was re-run, but only on the 244 significant genes listed in Supplementary Table S2, not on the full set of 17 285 genes. This choice was made due to limited computational resources, as running the full model would have required an estimated 100 hours.
- Errors detected: -

- Statistical Consistency: Among the 244 significant genes originally reported, the reproduced analysis confirmed the statistical significance of these same genes. However, the exact numerical values (Mean, standard deviation, Z value, P-value and adjusted P-value) differed slightly. These discrepancies are expected due to the nature of Bayesian inference, the absence of a random seed, and the relatively low number of MCMC iterations used (n.iter = 500). These settings may not be sufficient to ensure full convergence or reproducibility of posterior estimates and should be interpreted with caution.
We were unable to compare the rho values because they were not available in the provided Supplementary table S2, nor extracted in the R code to be include in the resulting output files.

6. Conclusion
- Summary of the computational reproducibility review
The results of the Supplementary table S2 in the original study was partially reproduced. We were able to confirm the statistical significance of the 244 genes reported in Supplementary Table S2 using the Bayesian PanCancer model in the provided code. However, the numerical results were not always identical. This is expected because Bayesian methods involve random sampling, the original code did not set a fixed random seed, and the number of iterations used was relatively low. Furthermore, the rho values were not available for comparison, limiting a full reproducibility assessment.
Several technical issues were also fixed during the reproduction process, such as hardcoded file paths, a missing function, and the absence of output directories, which were resolved to allow the code to run correctly on a different system. Due to computational limitations, running the full model on all 17,285 genes was not performed.

- Recommendations for authors
While the original analysis code was successfully used to confirm the statistical significance of the 244 genes, we recommend several improvements to enhance reproducibility:
-- Code annotation: Adding more detailed comments within the scripts would help users understand the logic behind each step and the purpose of specific commands or operations.
-- Set a random seed: Include set.seed() in all scripts to improve reproducibility across different runs.
-- Specify R and package versions: Provide the R version and exact package versions needed to run the code, via a requirements file for example.
-- Use relative file paths: Ensure that all necessary folders and functions are created or included by default to avoid path issues.
-- Increase MCMC robustness: Use a higher number of iterations and appropriate parameter settings to ensure better convergence and stability of posterior estimates.
-- Inform users about computation time: Clearly indicate in the README or publication the expected runtime of the code, especially if it requires several hours or days to complete.
-- Please also take a moment to check our website at https://www.editorialmanager.com/giga/l.asp?i=208092&l=RU6ZQV4L for any additional comments that were saved as attachments. Please note that as GigaScience has a policy of open peer review, you will be able to see the names of the reviewers.

Authors: We thank the reviewer for carefully evaluating the reproducibility of our work and for identifying areas where our code and documentation could be improved. We are pleased that the reviewer was able to reproduce the statistical significance of the 244 genes reported in Supplementary Table S2 using the provided Bayesian pan-cancer model. We also acknowledge the helpful feedback regarding technical issues, documentation, and computational reproducibility, which we have addressed in the revised submission.

Specifically, we have updated the scripts to use relative file paths instead of hardcoded directories, included the previously missing "*intsect*" function, and added automatic directory creation so that outputs can be written without error on any system. We also added more detailed comments within the scripts and clarified expected runtime requirements for users, as suggested. Please see the GitHub commits #31494a7 and #3f6e41a at https://github.com/Huang-lab/BEACON/commits/main/.

Regarding the reviewer's note that rho values were missing, we clarify that the rho estimates were in fact provided under the column labeled "Mean", which corresponds to the posterior mean correlation coefficient from the MCMC sampling. To avoid ambiguity, we have updated the supplementary tables and documentation to explicitly state that this column represents rho. We also modified the code so that this output is now labeled "rho" rather than "Mean" for clarity.

In addition, we incorporated the reviewer's suggestions to improve reproducibility by explicitly setting a random seed in all scripts, and by providing the R version and package versions used in a requirements file.

We agree with the reviewer that numerical differences across runs can occur due to the stochastic nature of Bayesian inference, especially with limited iterations. With the above changes, including higher iterations and fixed random seeds, we expect future runs to be more stable and reproducible. Finally, as added to the code availability section, this tool has been registered at bio.tools under the identifier biotoolsID: BEACON-x, and at SciCrunch.org under the identifier RRID: SCR_027484.

Overall, we are grateful for these constructive suggestions, which have helped us improve the clarity, usability, and reproducibility of both our code and manuscript.
