# Supplementary material for: Expression-driven genetic dependency reveals targets for precision oncology
Source: Gigascience. 2026 Jan 29;15:giag011. doi: 10.1093/gigascience/giag011 (PMC12970598; doi:10.1093/gigascience/giag011)
Supplement: giag011_Authors_Response_To_Reviewer_Comments_original_submission [file giag011_authors_response_to_reviewer_comments_original_submission.pdf]

Author's Response To Reviewer Comments

Authors: We sincerely thank the reviewers for the thoughtful and constructive feedback on our manuscript entitled “Expression-Driven Genetic Dependency Reveals Targets for Precision Oncology” (GIGA-D-25-00147). We have carefully considered the comments, particularly regarding the need to benchmark BEACON against established correlation-based approaches and demonstrate its improvements in real data, and have conducted additional systematic benchmarking analyses to address these concerns. We are pleased to submit a revised version of the manuscript for your consideration. Below is a point-by-point response to the reviewers’ comments.

Reviewer #1: The authors present BEACON, a method for identifying associations between the expression of a gene and sensitivity to the CRISPR knockout of that gene across a panel of cancer cell lines. These 'oncogene like' dependencies represent potential therapeutic targets that might be exploited for the development of new precision medicines in cancer. The issue that BEACON aims to address is the limited sample size (cell line count) in some specific cancer lineages and experimental noise that might result in spurious correlations between expression and CRISPR sensitivity. The authors demonstrate, using a modelling approach, that BEACON is more reliable for estimating correlation than simple Pearson's correlation when there is high-noise in the measurements. The majority of the manuscript focuses on analyses of dependencies systematically identified using the BEACON approach and their enrichment in drug targets and biological pathways. There is some experimental testing of three potential expression driven dependencies presented. The rationale for the overall approach and analyses are clear.

Authors: We sincerely thank the reviewer for their thoughtful and constructive evaluation of our manuscript. In response, we expanded our benchmarking analyses and show that BEACON consistently outperforms Pearson and Spearman correlations in identifying known druggable targets using real data, particularly in smaller and noisier lineages. We also clarified and consolidated our experimental validation results to ensure transparency.

Major comments

- Previous efforts have systematically associated gene/protein expression with CRISPR sensitivity across the same or related datasets (e.g. Pacini et al, Cancer Cell 2024 and Rohde et al, Molecular Systems Biology 2025 using CRISPR; McDonald et al, Cell 2017 and Tsherniak et al, Cell 2017 using RNAi) and so the primary contribution of this paper can be considered the development of the BEACON method. It is thus somewhat surprising that there is no real assessment of the improvements offered by BEACON when compared to simpler methods (Pearson correlation, Spearman correlation) or more more complex recent approaches (Rohde et al's BACON approach). The modelling approach suggests some improvements in specific circumstances (especially high noise) but it is not clear that this leads to improved dependency identification in the real data. Does BEACON identify known oncogene addictions better than these methods? Are the associations identified more reproducible (e.g. across alternative CRISPR screens or RNAi screens)?

Authors: We thank the reviewer for this validate suggestion; we validated BEACON against real data and incorporated these results in page 5-6:
To further validate BEACON on real data, we systematically benchmarked its performance against Pearson and Spearman correlations to identify a curated set of 2,993 druggable genes from DGIdb as the reference standard using this DepMap CRISPR dataset. andard. For each cancer lineage, we calculated the area under the precision–recall curve (AUPRC) for identifying DGIdb genes based on expression–dependency correlation scores. On average across all lineages, BEACON achieved an AUPRC improvement of 25/29% over Pearson and 23/29% over Spearman correlations (Figure S3), based on CRISPR vs. mRNA/protein expression data. Specifically, BEACON was the top-performing method in 17 of 24 lineages for GED (mRNA) and in 10 of 17 lineages for PED (protein) (Figure S3). The advantage was particularly pronounced in lineages with smaller sample sizes (e.g., Cervix, Oesophagus, Stomach, Endometrium, etc.), where AUPRC gains reached more than two-fold over Pearson/Spearman. An additional benchmarking against the 57 prioritized genes identified by Project DRIVE’s expression–dependency model (Pearson-based)29

showed that BEACON achieved average AUPRC gains of 94/530% over Pearson and 123/616% over Spearman correlation (Figure S3). BEACON was also the top-performing method in 18 of 24 lineages for GED (mRNA) and in 11 of 17 lineages for PED (protein). These results demonstrate that BEACON improves over simpler correlation measures and enhances the recovery of biologically validated dependencies.

- The experimental validation and the conclusions drawn from it are somewhat confusing. The authors assess three potential expression associated dependencies - two pan-cancer dependencies (GRHL2 and TP63) and one lineage specific dependency (PAX5 in myeloid cells). Only the lineage-specific dependency validated in the way that might be expected, with higher expression associated with increased dependency, leading the authors to conclude that lineage-specific dependencies may be more suitable targets than pan-cancer ones. Given the numbers analysed (3 genes) this suggestion is not well supported. Moreover the perturbation was performed using distinct approaches - CRISPR for PAX5 and shRNA for the other two genes - and only the knockdown of PAX5 was validated by Western blot. It is very hard to know what phenotypes might be a false positive from off-target shRNA effects or false-negatives from variable shRNA knockdown of the target. The results in S5C suggest that the two shRNAs for each gene cause somewhat discordant phenotypes, suggesting there may be some issues with knockdown efficiency. This could potentially be addressed by adding additional shRNAs for GRHL2 / TP63 or testing them using CRISPR perturbation as was done for PAX5. Validation of the knockdown of the intended target could also shed some light here. The manuscript also mentions experiments in an additional cell line (HCC15) but I cannot see these results presented in the main figures or supplement. It would be useful if all results for these two genes were presented in a single figure, with high and low expressing cell lines clearly marked,

Authors: Our rationale was to evaluate both pan-cancer and lineage-specific dependencies, as BEACON identifies candidates at both levels. While GRHL2 and TP63 appeared as pan-cancer GEDs, their protein-level PED associations were most pronounced in lung cancer, which led us to test them in lung cancer cell lines, whereas PAX5 was pursued as a clear lineage-specific dependency in hematopoietic and lymphoid cells. We acknowledge that the use of different perturbation methods—CRISPR for PAX5 and shRNA for GRHL2 and TP63—complicates direct comparison, but this choice was driven by feasibility and resources with the two collaborative teams at Vanderbilt and Gunma University who specialized in lymphoid and lung cancers, respectively. We also recognize the variability in shRNA efficacy and for transparency have added this to results:
To confirm inhibition of the target genes, qPCR validation in HARA cells showed that knockdown efficiency in the HARA cell line where the expression levels of TP63 and GRHL2 were reduced to 33/17% and 41/68%, respectively.

We now also present all replicate results (including HCC15 and HARA) in a single consolidated figure (Figure 6B) with high- and low-dependency cell lines clearly labeled so that the data can be interpreted transparently. Finally, we added a caveat at the end of this Result section:
However, given the limited scope of our validation of three targets, a more systematic validation of GED/PED targets will be required to determine the effectiveness of this target prioritization approach.

Minor:

- Previous work has established that in some cases lower expression of a gene can make cells more vulnerable to its perturbation (CYCLOPS genes, Nijhawan et al, Cell 2012). While these are not the focus of this manuscript, it would be useful for the authors to comment on the utility of BEACON for their identification.

Authors: We appreciate this helpful suggestion. Indeed, BEACON can in principle identify not only "negative" correlations (with negative rho, due to dependency score being negative) between expression and dependency (GED/PED, where higher expression is associated with stronger dependency) but also "positive" correlations, such as those characteristic of CYCLOPS genes, where lower expression increases vulnerability to perturbation. We have added a brief comment in the Discussion (page 15, lines 428-432):
Although in this study we emphasized negative associations where higher target expression corresponds to greater dependency, the Bayesian framework is symmetric and can also detect positive correlations. This

makes BEACON suitable for identifying CYCLOPS-type genes, where reduced expression confers greater vulnerability to perturbation42.

- p14 "Moreover, GED/PED targets were depleted of genes that were Essential In Culture" - it's not clear what this means or where the data comes from. By definition the gene set analysed are at least somewhat essential in culture

Authors: The “Essential In Culture” gene set was as defined by Hart et al. 2017. We have revised the text to clarify this point and now state (page 12, lines 338-342):
Moreover, GED/PED targets were underrepresented among the common essential genes (N=684 “Essential In Culture” genes based on 17 genome-wide CRISPR screens38), suggesting that BEACON identifies cell-specific vulnerabilities rather than dependencies universally required for cell viability (e.g., house-keeping genes) that could lead to off-target effects.

Reviewer #2: *The authors introduce BEACON, a Bayesian correlation approach designed to identify expression-driven dependency in cancer. Their hypothesis suggests that cancer cells with elevated expression of specific genes demonstrate increased vulnerability to the knockout of those same genes, thereby unveiling a promising new category of targets in precision oncology—particularly valuable for targeting cancer cells lacking druggable mutations.
*BEACON models expression levels and dependency scores as bivariate Gaussians and employs Markov Chain Monte Carlo (MCMC) sampling to estimate the correlation coefficient between them. They then compute p-values followed by rigorous multiple testing correction (BH based FDR correction).
*A notable strength of their approach lies in the integration of mass spectrometry proteomics data alongside transcriptomic and perturbation screening data, enhancing the robustness of their findings.
*Their work highlights some key insights:
- Gene expression-driven dependency (GED) candidates identified across lineages demonstrate enrichment for "DNA-binding transcription activator activity" and "DNA-binding transcription activator activity, RNA polymerase II-specific" pathways.
- The analysis successfully identifies compelling candidates with robust signals in both GED and PED (FERMT2, GRHL2, KLF5, CDK6, and CCND1), which are well-supported by existing drug evidence or established literature
- Clustering analyses reveal that cancer cells from pancreas and biliary tract tissues, as well as kidney and urinary tract tissue lineages, exhibit remarkably similar expression-driven dependency profiles. Additionally, lineage-specific genes such as transcription factors, cluster together in a manner consistent with existing literature
- Through Fisher's exact test, the authors demonstrate significant enrichments of druggable gene lists from DrugBank with expression-driven dependency patterns at both proteomic and transcriptomic levels
- Experimental validation shows that PAX5 is essential for PAX5-high B cell lymphoma cell growth, while TP63 and GRHL2 are essential for LSCC cell growth.

However, I have several principal concerns about the study that should be addressed to demonstrate the robust and superior performance of this proposed approach.

Authors: We thank the reviewer for their thorough summary and recognition of BEACON’s strengths. To address the principal concerns, we expanded our benchmarking analyses to systematically compare BEACON with Pearson and Spearman correlations in real data to identify druggable genes, clarified the validation experiments for TP63 and GRHL2, and refined methodological descriptions. These revisions reinforce that BEACON provides robust and practical improvements over existing approaches for uncovering expression-driven dependencies.

Major Comments:

1.Quantitative benchmarking: While the authors present a valuable contribution, the concept of correlating gene dependency scores to expression has been explored previously through approaches like Project DRIVE (E. Robert McDonald, III et al.) and APSIC (Montazeri et al.). BEACON demonstrates strong correlations across multiple lineages, representing broader scope compared to existing methods that appear more lineage-restricted. However, establishing BEACON's comparative advantages requires more

rigorous evaluation. Notably, Project DRIVE—a foundational paper in this field—already identified several BEACON candidates in their "Expression Correlation Analysis Identifies Oncogenes and Lineage-Specific Transcription Factors" section, while APSIC characterized many lineage-specific discoveries as tumor effector genes. BEACON's strength lies in integrating proteomic data with transcriptomic and perturbation screens, enabling identification of additional candidates like PAX5 for
hematopoietic and lymphoid tissue. To demonstrate the method's impact, I recommend systematic quantitative benchmarking against existing approaches.
Authors: We systematically compared BEACON GED/PEDs with those obtained from other approaches, including Pearson correlation (as used in Project DRIVE and BACON), and Spearman correlation (as additionally implemented in BACON). APSiC was build to identify new drivers via analyzing perturbation (shRNA) vs. mutation/CNVs, quoting https://academic.oup.com/nar/article/49/15/8488/6329117 “We considered the knockdown experiments of p genes across N cell lines. Let be viability of cell line upon knocking down gene and be a binary variable indicating whether a specific genetic alteration (i.e. mutation or copy number alteration) is present in gene j of cell line i.” Thus we did not benchmark against it.

Importantly, BEACON utilizes richer/complementary datasets than previous studies. Disentangling contributions of data richness versus methodological innovation would provide valuable insights into whether enhanced performance stems from improved data availability or genuine method improvements.
Authors: To address this, we applied all methods (Pearson, Spearman, and BEACON) to the same dataset consisting of matched mRNA/protein expression and CRISPR dependency data from DepMap. By holding the data constant, differences in performance directly reflect methodological advantages of BEACON rather than data availability.

Overall for benchmarking, the authors are strongly encouraged to utilize any comprehensive datasets that best demonstrate their method's competitive advantage and are not limited to the specific comparisons recommended above.
Authors: We used the DGIdb druggable gene set (n = 2,993) as a comprehensive and lineage-spanning reference standard. This enabled systematic benchmarking across all available lineages at both the mRNA and protein levels, ensuring that BEACON’s advantages are broadly generalizable and not restricted to a subset of examples (Figure S3). Combining all these points, we have added the following to Results:
To further validate BEACON on real data, we systematically benchmarked its performance against Pearson and Spearman correlations to identify a curated set of 2,993 druggable genes from DGIdb as the reference standard using this DepMap CRISPR dataset. andard. For each cancer lineage, we calculated the area under the precision–recall curve (AUPRC) for identifying DGIdb genes based on expression–dependency correlation scores. On average across all lineages, BEACON achieved an AUPRC improvement of 25/29% over Pearson and 23/29% over Spearman correlations (Figure S3), based on CRISPR vs. mRNA/protein expression data. Specifically, BEACON was the top-performing method in 17 of 24 lineages for GED (mRNA) and in 10 of 17 lineages for PED (protein) (Figure S3). The advantage was particularly pronounced in lineages with smaller sample sizes (e.g., Cervix, Oesophagus, Stomach, Endometrium, etc.), where AUPRC gains reached more than two-fold over Pearson/Spearman. An additional benchmarking against the 57 prioritized genes identified by Project DRIVE’s expression–dependency model (Pearson-based)29 showed that BEACON achieved average AUPRC gains of 94/530% over Pearson and 123/616% over Spearman correlation (Figure S3). BEACON was also the top-performing method in 18 of 24 lineages for GED (mRNA) and in 11 of 17 lineages for PED (protein). These results demonstrate that BEACON improves over simpler correlation measures and enhances the recovery of biologically validated dependencies.

2.Correlation method comparisons: Figure S2 shows that BEACON exhibits higher MSE at extremes, and the claimed advantage over Pearson for small sample sizes is difficult to quantify from the current visualization. While the theoretical expectation that BEACON should outperform Pearson in small samples is reasonable, the practical significance remains unclear from these simulations. I recommend demonstrating BEACON's advantage using real data by creating a curated list of established GEDs/PEDs and comparing performance between the two methods. This is particularly important since several of BEACON's hits were previously reported by Project DRIVE using simple Pearson correlations.

Authors: As seen in the prior response, we curated two complementary reference sets: (i) DGIdb druggable genes and (ii) the transcription factors previously identified in Project DRIVE. Using AUPRC as the

evaluation metric, BEACON consistently outperformed Pearson and Spearman correlations across both benchmarks, at both the GED (mRNA) and PED (protein) levels (Figure S3). The curation of Project DRIVE's gene list was added in addition to DGIdb druggable gene list to resolve this reviewer's concern.

Alternatively, if BEACON's advantage is indeed significant, please elaborate on the simulation results to better justify this claim with clearer quantitative metrics.

Authors: We also expanded our simulation analysis to include additional scenarios with finer resolution at very small sample sizes and under higher noise levels, running 100 replicates for each condition. These extended simulations demonstrate that BEACON consistently yields more accurate and better-calibrated correlation estimates than other methods, with the advantage being most pronounced in small-N settings and at moderate-to-high noise levels (≥50%) (Figure S2).

3.Validation experiments: I'm seeking clarification on the validation experiments for TP63 and GRHL2. These candidates were not sensitive to predicted dependency and the authors say that "pan-lineage targets may represent universal vulnerability and their inhibition may lead to undesired off-target effects on other cells". Are the authors positioning them as weaker candidates to illustrate the superiority of lineage-specific predictions like PAX5? Additionally, why were different experimental approaches used—CRISPR for PAX5 versus shRNA for TP63 and GRHL2? For a method aimed at identifying druggable targets, would drug based experiments be more relevant than knockdown approaches to better demonstrate clinical applicability?

Authors: We thank the reviewer for raising these important points. GRHL2 and TP63 were selected because, while they appeared as pan-lineage GEDs, their strongest protein-level PED associations were observed in lung cancer, which led us to test them in lung squamous carcinoma cell lines. In contrast, PAX5 was chosen as a clear example of a lineage-specific dependency. We have updated the text at the end of Result section given the limited sets of genes we were able to validate:
Thus, proteins showing lineage-specific dependencies may present as suitable precision oncology targets in the subset of tumors overexpressing the target gene and protein. However, given the limited scope of our validation of three targets, a more systematic validation of GED/PED targets will be required to determine the effectiveness of this target prioritization approach.

Regarding methodology, CRISPR-based validation was used for PAX5 while shRNA was used for GRHL2 and TP63 due to the resources available to the two collaborative teams at Vanderbilt and Gunma University who specialized in lymphoid and lung cancers, respectively.

While drug-based experiments would indeed demonstrate greater clinical applicability, our aim here was to provide proof-of-concept validation of BEACON's target predictions, whereas drug-based validation require different experimental design and introduce other complications that may not correspond with how good the target is (e.g., on-target efficacy and off-target effects of the drugs used).

Minor comments

1. In Figure 4A, the caption refers to the plot as a heatmap, but the visualization appears to be a scatterplot. Please clarify whether the heatmap is missing or modify the caption appropriately. Additionally, I recommend using a different shade of green, as the current color choice makes some gene names difficult to read.

Authors: We thank the reviewer for noting this. Figure 4A is a scatterplot (not a heatmap), and the caption has been corrected. We also adjusted the green color to improve readability of gene names.

2. In Fig S5A, please add a legend for tumor and normal

Authors: We have added a legend to Figure S6A (previously Fig S5A) to clearly distinguish tumor and normal samples.

3. For the TP63 and GRHL2 validation experiments, please include results for all four cell lines. The current manuscript is missing HCC15-shTP63, HCC15-shGRHL2, and HARA-shGRHL2 plots.

Authors: All replicate results are now presented together in a consolidated figure (Figure 6B), including HCC15 and HARA for GRHL2. HCC15 was not used in the TP63 experiments.

4. How many replicates were the experiments performed on? Is it N= 3 for all experiments?

Authors: Each colony formation and proliferation assay was performed with three replicate wells (N=3), as noted in the Methods section and figure legends.

5. Missing some text here - "BEACON offers the unique advantage of utilizing prior distributions that are less susceptible to outliers, especially in multiple lineages where the number of cell lines."

Authors: The sentence has been corrected to read: “BEACON offers the unique advantage of utilizing prior distributions that are less sensitive to outliers, which is particularly beneficial in lineages where the number of available cell lines is small and thus more vulnerable to the influence of outliers.”

Reviewer #3: The authors develop a method for correlating gene and protein expression with cellular dependencies using the resources of DepMap. The innovation appears to be a Bayesian approach to the correlation analysis. They use this approach to identify potential therapeutic targets and evaluate some top candidates using in vitro experiments. The paper is fairly straightforward to follow.

Major comments: 1. Benchmarking - given the non-linear relationships shown in Fig 2, is a comparison with the Pearson method the most appropriate? Would a Spearman's be better?

Authors: We thank the reviewer for this helpful suggestion. In addition to Pearson, we have now benchmarked BEACON directly against Spearman correlation. Using both simulated and real datasets (DGIdb druggable genes and transcription factors identified in Project DRIVE). This is added in Results text:
We benchmarked BEACON’s Bayesian correlation against Pearson correlation, which was used in project DRIVE29, and against both Pearson and Spearman correlation measures, which were employed in BACON30. Simulations were performed on expression and dependency datasets across a range of correlation levels (from -1 to 1, with 0.25 intervals) and sample size (number of cell lines, 5, 7, 10, 20, 30, 60, 100), with different fraction (0.1, 0.3, 0.5, 0.8, 1) of samples corrupted by noise to enable direct comparison of methodological performance (Figure S2). Based on these simulations, we observed that the Bayesian method is better than Pearson correlation for estimating moderate true correlation (|rho| < 0.75) in small sample size, and preferable in noisy data (noise level ≥ 0.5, i.e., 50% or more of the samples are corrupted by noise to become outliers), regardless of sample size or true correlation level.
To further validate BEACON on real data, we systematically benchmarked its performance against Pearson and Spearman correlations to identify a curated set of 2,993 druggable genes from DGIdb as the reference standard using this DepMap CRISPR dataset. andard. For each cancer lineage, we calculated the area under the precision–recall curve (AUPRC) for identifying DGIdb genes based on expression–dependency correlation scores. On average across all lineages, BEACON achieved an AUPRC improvement of 25/29% over Pearson and 23/29% over Spearman correlations (Figure S3), based on CRISPR vs. mRNA/protein expression data. Specifically, BEACON was the top-performing method in 17 of 24 lineages for GED (mRNA) and in 10 of 17 lineages for PED (protein) (Figure S3). The advantage was particularly pronounced in lineages with smaller sample sizes (e.g., Cervix, Oesophagus, Stomach, Endometrium, etc.), where AUPRC gains reached more than two-fold over Pearson/Spearman. An additional benchmarking against the 57 prioritized genes identified by Project DRIVE’s expression–dependency model (Pearson-based)29 showed that BEACON achieved average AUPRC gains of 94/530% over Pearson and 123/616% over Spearman correlation (Figure S3). BEACON was also the top-performing method in 18 of 24 lineages for GED (mRNA) and in 11 of 17 lineages for PED (protein). These results demonstrate that BEACON improves over simpler correlation measures and enhances the recovery of biologically validated dependencies.

2. The analysis identifies dependencies that are proposed as therapeutic targets, however while the proteins can be druggable, what about normal tissue effects? Some of these are likely lineage-defining proteins that could be highly expressed in normal tissues. Is is notable that in Fig 5B, C that the existing drug targets have a lower association strength than other GEDs identified. Does this suggest that the strongest correlations might be lineage-crucial genes that are too important for normal tissue function to make good drug targets? This needs further consideration in the discussion. Are there any pathways differences between these groups (known drug targets vs others)? For example you might expect more tissue lineage Tfs in the "other" category, while the approved drug targets perhaps more cell surface receptors.
Authors: We thank the reviewer for bringing up this very interesting point. As our pathway analyses revealed, some of the strongest GED/PED associations are enriched for DNA-binding transcription activator (Figure 2D, 3D) that are transcription factors (TFs). In our knowledge, many disease-associated TFs have not been drugged not due to their potential lineage essentiality in adults, but also due to their lack of natural binding pockets and complex protein-DNA or protein-protein interactions. Thus, how druggable and effective they may serve as drug targets remains less tested than other protein families more amenable to prior small molecule/antibody-based approaches. Meanwhile, the DrugBank list is known to be enriched for enzymes, transporters, receptors (https://www.proteinatlas.org/humanproteome/tissue/druggable). We have added to the end of this section:
However, many of these top candidates may have more extreme GED/PED rho values than currently druggable genes; it remains to be tested whether that is a confounding with the protein classes more amenable to current drug modalities or there may be a more desirable GED/PED window for prioritizing therapeutic targets.
We have also added text to the Discussion (underlined texts are the additions):
Many GED/PED gene targets are lineage-specific transcription factors (TFs); these agree with recent single-cell studies and synthesis that posited the "developmental constraint model of cancer cell states", which cancer cell states correspond to and may be constrained by the landscape of "developmental map"46. Thus, a cancer cell adopting a specific developmental state may require activation of such transcription factors and become genetically dependent. Traditionally, TFs were not easily addressable using small molecule or antibody-based approaches due to their lack of binding pockets and complex intermolecular interactions. While such targets used to be considered undruggable, new drug modalities such as proteolysis-targeting chimera (PROTAC) are showing promises43,47-50, particularly in cases where there may be a sufficient therapeutic window in inhibiting these TFs, e.g., to treat adult tumors where the target TFs were only essential in early development and in tumor cells.
3. The cell assays performed should effectively be replicating the results of the dependencies on which BEACON is based (DepMap), so why do you get different results? Is it because of the different methods used ie shRNA (not seeing the correlation between expression and dependency) vs CRISPR (replicating the correlation)? If you look at older DepMap scores when they used knockdown rather than CRISPR can you replicate your results?
Authors: The discrepancies between our validation assays and DepMap dependency scores reflect both methodological differences and phenotypic readouts. DepMap scores are derived from pooled CRISPR or shRNA screens after many cell passages and barcode sequencing from pooled cell populations, where cell viability is inferred indirectly through barcode representation after cross-gene normalization. Thus, analyses of Broad and Sanger's large-scale CRISPR knockout screens show correlation but indeed variations of the derived gene score (PMID: 31862961). By contrast, our colony formation assays in lung cancer cells capture proliferative potential, integrating effects on cell survival, division, and stress responses. Similarly, our live cell counts in PAX5 B-cell experiments reflect more acute changes in growth kinetics, which may yield different sensitivity profiles. In our focused experiments, we were also able to conduct qPCR and western blots to validate knockdown/knockout efficiency. Thus, while the broad correlation with DepMap is informative, focused assays provide complementary, context-specific insights into how genetic perturbations affect distinct cellular outcomes.
We have added briefly to the Results, "These focused experiments provide complementary validation to the functional data from the DepMap screen because, in our experiments, all functional readouts were collected within four days of the perturbation (Methods), whereas the DepMap CRISPR screen are performed at 14-21 days and scores are inferred indirectly through barcode representation after cross-gene normalization."
We also thank the reviewer for the suggestion to examine DepMap's RNAi data, albeit this screen had a documentation of even further passaging times. In general, DepMap's RNAi dependency & CRISPR dependencies for these assayed genes agree well in these tested cell lines. Based on this result, we added,

“We further queried DepMap RNAi data (passaged for 16 doublings, up to 40 days)5 for these genes that showed consistent dependencies with the original DepMap-based predictions (Figure S7), suggesting potential variations due to shRNA constructs or experimental durations.”

4. Although mycoplasma testing was done, were the cell lines re-authenticated by STR profiling at any point?

Authors: All cell lines used in this study were obtained from authenticated biobanks (as listed in Table S15), and mycoplasma testing was performed regularly. The Gunman team member (Dr. Reika) who provided the cell lines for lung cancer experiments had left for industry a few years ago and we did not receive a confirmation on this, and thus we did not change texts there.
The Vanderbilt team confirmed all lines used for the PAX5 experiments were confirmed with STR profiling and we have added accordingly to that section of the Methods.

5. QPCR is mentioned n the methods but not provided in the results that I can find. Did this validate gene knockdown by shRNA? Any correlation between % KD and proliferation/colony forming effect?

Authors: qPCR was performed to confirm knockdown of TP63 and GRHL2 following shRNA treatment in the HARA cell lines. We also recognize the variability in shRNA efficacy and for transparency have added this to results:
To confirm inhibition of the target genes, qPCR validation in HARA cells showed that knockdown efficiency in the HARA cell line where the expression levels of TP63 and GRHL2 were reduced to 33/17% and 41/68%, respectively.

In HARA, the more efficacious sh-TP63-2 showed trends of higher reduction in colony formation than sh-TP63-1, but vice versa for the GRHL2 shRNAs. In all cases the knockdowns were significantly effective in reducing colony formation. We did not describe the potential correlation between % KD and proliferation/colony forming effect as qPCR was only conducted in HARA but not other cell lines.

6. In the discussion it should be acknowledged that cancer subtypes exist within lineages that are molecularly and clinically distinct and so the method might be missing targets specific for these eg ER+ and ER- breast cancer.

Authors: We agree with the reviewer that molecular and clinical subtypes within a lineage (e.g., ER^+ vs. ER^- breast cancer) may harbor distinct dependencies that could be missed when analyzing at the lineage level. We have added a note in the Discussion:
It is also important to note that within a given lineage, molecular and clinical subtypes (e.g., ER^+ vs. ER^- breast cancer) may harbor distinct dependencies that could be masked when analyzing at the lineage level. Applying BEACON to subtype-stratified datasets may therefore reveal additional, clinically relevant vulnerabilities. As larger and better-annotated datasets become available, this represents an important direction for future work.

Minor comments:
1. Results para 1 "especially in multiple lineages where the number of cell lines." Missing something in this sentence?

Authors: The sentence has been corrected to read: “BEACON offers the unique advantage of utilizing prior distributions that are less sensitive to outliers, which is particularly beneficial in lineages where the number of available cell lines is small and thus more vulnerable to the influence of outliers.”

2. Needs some grammar review

Authors: We have carefully reviewed the manuscript for grammar and style and have corrected the relevant sentences for clarity and readability throughout.

3, Please italicise all gene names (when referring to gene, not protein) eg CCNE1 amplification etc

Authors: All gene symbols have now been italicized throughout the manuscript when referring to genes (e.g., CCNE1-amplified), in accordance with standard nomenclature conventions.
4. Fig S5A - legend or axis labels for N and T needed.

Authors: The axis labels “Tumor” and “Normal” has now been added to clearly distinguish those samples in the figure. With the inclusion of an additional panel, this figure is now presented as Figure S6A in the revised manuscript.

5. Fig S5C, D - these are proliferation not colony forming assays as stated in the text.

Authors: The text has been revised to accurately describe Figures S6C and S6D as proliferation assays rather than colony-forming assays. The Results section now states: “In KNS-62 and H1703 LSCC cells, the knockdown of TP63 using two shRNA constructs (sh-TP63-1 and sh-TP63-2) resulted in a significant reduction in colony formation and cell viability (reduced proliferation) compared to controls($p<0.01$) (Figure 6A, Figure S6D).”

6. Please include number of replicates and type of error bars in figure legends for cell assays

Authors: The figure legends for all cell-based assays have been updated to include the number of replicates (N = 3) and the type of error bars (standard deviation).

Reviewer #4: Reproducibility report

1. Summary of the Study The authors developed a Bayesian method called BEACON to integrate multi-omics data. The method was tested on cancer cell lines across 17 tissue types to identify expression-driven dependencies. The method recovered known drug targets and identified novel candidates. The study concludes this method provides a systematic approach to identify precision oncology targets.

2. Scope of reproducibility According to our assessment the primary objective is: to identify expression-driven dependencies across cancer cell lines from multiple lineages enabling the discovery of genes whose expression levels correlate with cancer cell dependency scores. –
Outcome: Identification of genes with significant expression-driven dependencies across pan-lineage cancer cell lines.
- Analysis method outcome: "BEACON calculated the Bayesian correlation between the gene's expressions and CERES cancer dependency scores 25 across the pan-lineage cell lines. BEACON modeled expression levels and dependency scores as the bivariate Gaussians and used Markov Chain Monte Carlo (MCMC) sampling to estimate the correlation coefficient rho between them. Given the null hypothesis that the uncorrelated expression and dependency of a gene has the 0 rho coefficient, we statistically tested each gene's rho estimate obtained from the MCMC simulation as follows. Assume that the MCMC sampling is carried out for a null gene's expression and dependency, then we expect that the distribution of the rho estimate accumulated over the MCMC iterations will be centered at zero. Based on this rationale, we computed the z-score of i-th gene as the deviation of the MCMC estimate of rho from the expected (null) value (i.e., zero) in terms of the standard deviation observed in the simulated distribution, i.e., z(i) = rhoMCMC(i) / SDMCMC(i). Since the z-values, by nature, follow a normal distribution with zero-mean and unit-variance, then we computed the p- value for each gene's rho estimate as the probability of observing a value as extreme as the computed z-value for that gene. We multi-testing corrected the resulting p-values using the BH procedure for FDR." (page 19 -Methods section / mRNA expression-driven dependency (GED))
- Main result: "We first analyzed the pan-lineage GED by using mRNA levels and the corresponding dependency scores from 854 cell lines with available data across 17 lineages and identified 244 genes showing significant association (correlation coefficient, rho < -0.25, FDR < 0.05)" (page 7 - Results section / Cancer vulnerability targets showing gene expression-driven dependency (GED))

3. Availability of Materials
a. Data

- Data availability: Open
- Data completeness: Complete, all data necessary to reproduce main results are available.
- Access Method: Repository
- Repository: https://urldefense.proofpoint.com/v2/url?u=https-3A__doi.org_10.6084_m9.figshare.19700056.v2&d=DwIBaQ&c=shNJtf5dKgNcPZ6Yh64b-ALLUrcfR-4CCQkZVKC8w3o&r=88-dBITsh8vXfnQjNN0pRGpahxI_Sccu4B-wNY_gsU4&m=QnwsYAvlqsJ9rZbiozGbBdLREQ59Pq0O2jitd0FjFA1KqZuHTZDAkiy8-tAbmKGL&s=SNkoITd2TWtWYm6mVW11wfiv2qKBRp4piBeZy4Er5Wo&e=
-Data quality: Structured

b. Code
- Code availability: Open
- Programming Language(s): R
- Repository link: https://urldefense.proofpoint.com/v2/url?u=https-3A__github.com_Huang-2Dlab_BEACON&d=DwIBaQ&c=shNJtf5dKgNcPZ6Yh64b-ALLUrcfR-4CCQkZVKC8w3o&r=88-dBITsh8vXfnQjNN0pRGpahxI_Sccu4B-wNY_gsU4&m=QnwsYAvlqsJ9rZbiozGbBdLREQ59Pq0O2jitd0FjFA1KqZuHTZDAkiy8-tAbmKGL&s=ly6kaHNqm3UtC0ImX6NAlr4fBkJv4CgaN3uAfeBNmIE&e= - License: MIT license
- Repository status: Public
- Documentation: Readme file

4. Computational environment of reproduction analysis
- Operating system for reproduction: MacOS 15.5
- Programming Language(s): R
- Code implementation approach: Using shared code
- Version environment for reproduction: R version 4.5.0/RStudio 2025.05.1

5. Results
5.1 Original study results
- Results 1: Supplementary table S2 5.2 Steps for reproduction
-> Run the code PanLineageMCMC.R
- Issue 1: File import paths and incorrect file name
-- Resolved: In the original code, there were fixed file paths that only worked on one specific computer. This caused problems when running the code on other computers. To fix this, I recommended to use relative paths, which are based on where the script is located. This way, the code can be run on any computer without needing to change the paths each time.

```
------------------ Start of script ------------------
sam.dep = read.csv(file.path(getwd(), "DepMap_data",
"sample_info.csv"))
------------------- End of script -------------------
```

- Issue 2: Missing function "intsect" at line 162
-- Resolved: The script called a function intsect that was not defined, leading to an error. Upon request, the authors provided the missing function and added it to the main script (PanLineageMCMC.R).

- Issue 3: Output directory not created.
-- Resolved: The script attempted to write output files to a directory that was not created beforehand. This caused errors during the loop execution when trying to save results. A directory check and automatic creation script was added. If the output folder does not exist, it is now created automatically before the loop runs.

```
------------------ Start of script ------------------
dir_path <-
paste0('../out/jags.nadapt',n.adapt,'.update',n.update,'.mcmc
',n.iter,'.simulation_SD_22Q2')
if (!dir.exists(dir_path)) {
```

```
dir.create(dir_path, recursive = TRUE)
}
------------------- End of script -------------------
```

5.3 Statistical comparison Original vs Reproduced results
- Results: Table.mRNA.dependency.Bayesian.pancancer file attached
- Comments: The Bayesian PanCancer analysis was re-run, but only on the 244 significant genes listed in Supplementary Table S2, not on the full set of 17 285 genes. This choice was made due to limited computational resources, as running the full model would have required an estimated 100 hours.
- Errors detected: -
- Statistical Consistency: Among the 244 significant genes originally reported, the reproduced analysis confirmed the statistical significance of these same genes. However, the exact numerical values (Mean, standard deviation, Z value, P-value and adjusted P-value) differed slightly. These discrepancies are expected due to the nature of Bayesian inference, the absence of a random seed, and the relatively low number of MCMC iterations used (n.iter = 500). These settings may not be sufficient to ensure full convergence or reproducibility of posterior estimates and should be interpreted with caution.
We were unable to compare the rho values because they were not available in the provided Supplementary table S2, nor extracted in the R code to be include in the resulting output files.

6. Conclusion
- Summary of the computational reproducibility review
The results of the Supplementary table S2 in the original study was partially reproduced. We were able to confirm the statistical significance of the 244 genes reported in Supplementary Table S2 using the Bayesian PanCancer model in the provided code. However, the numerical results were not always identical. This is expected because Bayesian methods involve random sampling, the original code did not set a fixed random seed, and the number of iterations used was relatively low. Furthermore, the rho values were not available for comparison, limiting a full reproducibility assessment.
Several technical issues were also fixed during the reproduction process, such as hardcoded file paths, a missing function, and the absence of output directories, which were resolved to allow the code to run correctly on a different system. Due to computational limitations, running the full model on all 17,285 genes was not performed.

- Recommendations for authors
While the original analysis code was successfully used to confirm the statistical significance of the 244 genes, we recommend several improvements to enhance reproducibility:
-- Code annotation: Adding more detailed comments within the scripts would help users understand the logic behind each step and the purpose of specific commands or operations.
-- Set a random seed: Include set.seed() in all scripts to improve reproducibility across different runs.
-- Specify R and package versions: Provide the R version and exact package versions needed to run the code, via a requirements file for example.
-- Use relative file paths: Ensure that all necessary folders and functions are created or included by default to avoid path issues.
-- Increase MCMC robustness: Use a higher number of iterations and appropriate parameter settings to ensure better convergence and stability of posterior estimates.
-- Inform users about computation time: Clearly indicate in the README or publication the expected runtime of the code, especially if it requires several hours or days to complete.
-- Please also take a moment to check our website at https://www.editorialmanager.com/giga/l.asp?i=208092&l=RU6ZQV4L for any additional comments that were saved as attachments. Please note that as GigaScience has a policy of open peer review, you will be able to see the names of the reviewers.

Authors: We thank the reviewer for carefully evaluating the reproducibility of our work and for identifying areas where our code and documentation could be improved. We are pleased that the reviewer was able to reproduce the statistical significance of the 244 genes reported in Supplementary Table S2 using the provided Bayesian pan-cancer model. We also acknowledge the helpful feedback regarding technical issues, documentation, and computational reproducibility, which we have addressed in the revised submission.

Specifically, we have updated the scripts to use relative file paths instead of hardcoded directories, included the previously missing “intsect” function, and added automatic directory creation so that outputs can be written without error on any system. We also added more detailed comments within the scripts and clarified expected runtime requirements for users, as suggested. Please see the GitHub commits #31494a7 and #3f6e41a at https://github.com/Huang-lab/BEACON/commits/main/.

Regarding the reviewer’s note that rho values were missing, we clarify that the rho estimates were in fact provided under the column labeled “Mean”, which corresponds to the posterior mean correlation coefficient from the MCMC sampling. To avoid ambiguity, we have updated the supplementary tables and documentation to explicitly state that this column represents rho. We also modified the code so that this output is now labeled “rho” rather than “Mean” for clarity.

In addition, we incorporated the reviewer’s suggestions to improve reproducibility by explicitly setting a random seed in all scripts, and by providing the R version and package versions used in a requirements file.

We agree with the reviewer that numerical differences across runs can occur due to the stochastic nature of Bayesian inference, especially with limited iterations. With the above changes, including higher iterations and fixed random seeds, we expect future runs to be more stable and reproducible. Finally, as added to the code availability section, this tool has been registered at bio.tools under the identifier biotoolsID: BEACON-x, and at SciCrunch.org under the identifier RRID: SCR_027484.

Overall, we are grateful for these constructive suggestions, which have helped us improve the clarity, usability, and reproducibility of both our code and manuscript.

Editorial Manager®

Cookie Preference Center

We use cookies which are necessary to make our site work. We may also use additional cookies to analyze, improve and personalize our content and your digital experience. For more information, see our Cookie Policy.

You may choose not to allow some types of cookies. However, blocking some types may impact your experience of our site and the services we are able to offer. See the different category headings below to find out more or change your settings.

Allow all

Manage Consent Preferences

Strictly Necessary Cookies

Always active

These cookies are necessary for the website to function and cannot be switched off in our systems. They are usually only set in response to actions made by you which amount to a request for services, such as setting your privacy preferences, logging in or filling in forms. You can set your browser to block or alert you about these cookies, but some parts of the site will not then work.

Functional Cookies

These cookies enable the website to provide enhanced functionality and personalisation. They may be set by us or by third party providers whose services we have added to our pages. If you do not allow these cookies then some or all of these services may not function properly.

Performance Cookies

These cookies allow us to count visits and traffic sources so we can measure and improve the performance of our site. They help us to know which pages are the most and least popular and see how visitors move around the site.

Targeting Cookies

These cookies may be set through our site by our advertising partners. They may be used by those companies to build a profile of your interests and show you relevant adverts on other sites. If you do not allow these cookies, you will experience less targeted advertising.

Cookie List

Clear

Apply Cancel

Consent Leg.Interest

Confirm my choices

Powered by onetrust
