# Supplementary material for: Expression-driven genetic dependency reveals targets for precision oncology
Source: Gigascience. 2026 Jan 29;15:giag011. doi: 10.1093/gigascience/giag011 (PMC12970598; doi:10.1093/gigascience/giag011)
Supplement: giag011_Authors_Response_To_Reviewer_Comments_revision_1 [file giag011_authors_response_to_reviewer_comments_revision_1.pdf]

Author's Response To Reviewer Comments

Reviewer #1: The addition of a benchmarking effort comparing the BEACON method against Pearson and Spearman Correlati
significantly strengthens the manuscript. The authors have therefore addressed the majority of my concerns.

The results from the GRHL2 and the TP63 experiments are still a little confusing, especially since the published shRNA (DEME
scores in Figure S7 appear more concordant with the published CRISPR experiments than with the authors' shRNA experimen
The duration of the experiment does not appear to explain these results - the authors see greater toxicity in a 4 day experim
than was observed in the longer term pooled shRNA/CRISPR experiments. One possibility is that these results reflect some of
target effects of GRHL2 / TP63 shRNA in the authors experiments. I am not suggesting additional experiments, I would simpl
suggest that the authors expand a little on the discussion of these results.

Authors: We thank the reviewer for this insightful observation. We agree that the relative toxicity observed in our short-term
8 assays for TP63 and GRHL2 appears stronger than what is reflected by the published pooled shRNA (DEMETER) or CRISPR
CERES scores. As noted, several factors may contribute to these differences without necessarily indicating a biological discrep
Short-term viability assays can be more sensitive to acute perturbation effects—including transient stress responses or early
growth disadvantages—that are averaged out in longer-term pooled shRNA/CRISPR screens. Additionally, although we used t
independent shRNA constructs per gene, we cannot fully exclude the possibility of partial off-target effects contributing to the
observed magnitude of growth inhibition.

We have now expanded the Discussion to acknowledge these considerations and to clarify that the overall directionality of the
results is fully concordant with both DEMETER and CERES scores, while the differences in effect size likely reflect methodolog
distinctions rather than contradictory biology:

“For TP63 and GRHL2, our short-term CCK-8 viability assays showed stronger reductions in proliferation than the effect sizes
suggested by published DEMETER shRNA and CERES CRISPR scores. Although the direction of the dependency was fully
concordant across all datasets, the magnitude of toxicity differed. Short-term assays can capture acute cellular responses to
knockdown—such as transient growth delay or stress-induced proliferation defects—that are attenuated or averaged out in th
longer-term pooled screens used to generate DEMETER/CERES scores. Furthermore, despite using two independent shRNAs f
each gene, we cannot entirely exclude minor off-target contributions to the observed effect sizes. Overall, our experiments ar
DepMap data both support that TP63 and GRHL2 are general functional dependencies in LSCC.”

Reviewer #2: In their revised manuscript, the authors have addressed several concerns raised in my initial review, resulting
notably strengthened contribution. The authors are appreciated for their efforts in conducting additional benchmarking analys
and expanding the simulation studies. However, the newly introduced figures reveal some issues that warrant attention befor
manuscript can be recommended for publication.

- The simulation results seem to suggest that Spearman correlation outperforms BEACON at noise levels ≤ 0.5 and larger san
sizes, while BEACON exhibits performance comparable to Pearson, especially for stronger correlations. Could the authors prov
an explanation for these observed patterns and discuss the conditions under which BEACON offers advantages over these me
in the methods section of the manuscript? The current statement—"...while Spearman better captured monotonic non-linear
trends, though this advantage largely disappeared in small, noisy cohorts"—is limited and would benefit from an expansion to
delineate the scenarios where BEACON's methodology provides meaningful benefits over Spearman correlations.

Authors: We thank the reviewer for their careful reading of the new simulation figures. The reviewer is correct that Spearman
correlation performs well —and in some settings better than BEACON— when sample sizes are sufficiently large and noise lev
are low to moderate. This arises because Spearman is non-parametric and robust to mild deviations from linearity, allowing it
capture monotonic relationships efficiently when enough observations are available. In contrast, BEACON is designed to stabi
correlation estimation under small sample sizes and high-noise regimes, conditions where rank-based methods become unsta
and Pearson is strongly influenced by outliers.

We have expanded the Methods section to explain these performance regimes more explicitly. In brief, BEACON’s main
advantages appear when (i) sample sizes are ≤10–15; (ii) noise/outlier fraction ≥0.3–0.5; and (iii) moderate underlying
correlations (|ρ| < 0.75), where Bayesian shrinkage prevents the inflation or sign-flipping commonly observed with

Pearson/Spearman under limited or noisy data. We have now added text detailing the scenarios in which BEACON has a pract
advantage and situations where Spearman performs comparably or better. These clarifications are now included in the revise
manuscript (in Methods section under "mRNA expression-driven dependency (GED)"):

"Spearman performance degraded substantially under small sample sizes (<10–15 cell lines) or high noise levels (≥30–50%
outliers), where rank estimates become unstable. In contrast, BEACON's Bayesian shrinkage stabilized correlation estimation
precisely these regimes, yielding more accurate estimates for moderate correlations (|ρ| < 0.75) and noisy or limited datase
Thus, BEACON is most advantageous where lineage-level sample sizes are small, or heterogeneity introduces substantial nois
whereas Spearman remains competitive for larger, cleaner datasets."

- The use of different experimental approaches for validation—CRISPR-based methods for PAX5 versus shRNA for GRHL2 and TP63—is understandable given logistical and resource constraints, but represents a limitation that warrants acknowledgemen Given the well-documented variability between CRISPR and shRNA screening platforms in the literature, this inconsistency lin the comparability of experiments. I recommend that the authors add a brief statement in the Discussion section clarifying tha current experiments were designed to provide proof-of-concept validation of BEACON's predictive capabilities, while emphasiz that future work should prioritize large-scale, methodologically uniform validation studies to more rigorously assess the reliab and generalizability of BEACON's predictions.

Authors: We appreciate this important point. We agree that the use of different perturbation platforms (CRISPR for PAX5 vers shRNA for TP63/GRHL2) represents a limitation, given the well-known systematic differences between CRISPR and shRNA screening modalities. Our experiments were intended as proof-of-concept validations of BEACON-predicted dependencies, an resource constraints precluded a uniform experimental framework across all targets. In accordance with the reviewer's sugge we have added a brief statement to the Discussion acknowledging this limitation and emphasizing that future work should fo systematic, platform-consistent perturbation studies to fully assess the reliability and generalizability of BEACON predictions.

"Finally, we acknowledge that the validation experiments we performed employed different perturbation platforms—CRISPR f PAX5 and shRNA knockdown for TP63 and GRHL2. These results should be interpreted as proof-of-concept rather than direct cross-gene comparisons. Future work using uniform, large-scale perturbation frameworks will be important for fully assessin robustness and generalizability of BEACON-predicted dependencies."

- For PED, the AUPRC values for the DRIVE dataset appear notably low across all methods. Furthermore, it is surprising to se Pearson correlation is low in performance, given that DRIVE targets are Pearson-based, and one would expect stronger concordance under these conditions. Is this because of the difference between DRIVE and DepMap datasets? Could the autho provide an explanation for these unexpectedly low AUPRC values?

Authors: The primary reason is that the DRIVE benchmark set was constructed using mRNA-based Pearson correlations, whe our PED analysis is based on protein expression from the Nusinow et al. mass-spectrometry dataset. Because mRNA–protein concordance is generally modest for many genes, the recoverability of DRIVE-defined targets in a protein-based correlation framework is limited, independent of the correlation method. In addition, the number of cell lines with both protein and dependency data is limited (now shown in Fig S3) and thus traditional correlation approaches do not work well. We have now clarified this in the revised Methods section under "mRNA expression-driven dependency (GED)":

"The AUPRC values for PED were relatively low across all correlation methods because DRIVE targets are defined using mRNA based Pearson correlations, whereas PED analysis uses protein expression that is known to only moderately correlate with m levels. Additionally, the smaller number of cell lines with proteomic data requires the BEACON approach to recover known tar in many tissue contexts."

- Please clarify the meaning of the XX/YY% notation to ensure unambiguous interpretation.

Authors: These values refer to the remaining expression levels following knockdown by the two shRNA constructs (sh1 and sh We have clarified this in the revised manuscript:

"qPCR validation showed that the shRNAs reduced TP63 expression to 33% and 17% (for sh1 and sh2, respectively) and GRH expression to 41% and 68%."

Comments on figures:

- The lineage legends in Figures 2C, 3C, and 5D can be reduced to show only the lineages actually depicted in each panel, as

current legends are distracting.

Authors: We have now added legends in the panels indicating which lineage-specific data is displayed, and revised the captio
improve clarity.

- The genes mentioned as "highlighted" in Figure 4 (SOX10, TP63, IRF4, and MYB) are not visually distinguished in the figure
Please add highlighting or revise the text.

Authors: We have revised the text to indicate that these genes are “labeled” rather than “highlighted”.

- Figures 5B-C: Should 'Drug targets' and 'Other genes' be differentiated by color in the legend? This distinction is currently unclear.

Authors: We apologize for this rendering issue — it resulted from a figure conversion error during the submission process. Th
original figures correctly distinguish these groups by color, and the updated figures now display properly.

- Please Italicize gene names in plots to separate genes from proteins.

Authors: All gene names have now been italicized in the figures, legends, and captions.

- Figures in S4 and S6 appear to be missing axis labels. Additionally, Figure S6C appears to have a rendering issue in the title
should be corrected.

Authors: These were caused by figure conversion errors during submission. All axis labels and titles have now been corrected
the updated supplementary figures.

Reviewer #4: Reproducibility report for: Expression-Driven Genetic Dependency Reveals Targets for Precision Oncology
Journal: Gigascience
ID number/DOI: GIGA-D-25-00147
Reviewer(s): Laura Caquelin, Department of Clinical Neuroscience, Karolinska Institutet, Sweden
----

1. Context

This report corresponds to a second assessment of the computational reproducibility of the article GIGA-D-25-00147, followin
revision by the authors after the first round of review.

The scope of the computational reproducibility review is to reproduce the results presented in Table S2 relative to the identifi
of genes with significant expression-driven dependencies across pan-lineage cancer cell lines. The identification used a develo
method called BEACON, based on Bayesian statistics, to find how strongly each gene's expression is correlated to dependency
scores across the pan-lineage cell lines, by modeling the data with Gaussian distributions, estimating correlations using MCMC
sampling, and testing significance with z-scores and FDR correction.

----

2. Changes since the first review

The authors made some changes to the code according to the comments from the first computational reproducibility review. T
text in the manuscript related to Table S2 was slightly different, but the numerical values and observations remained unchan

----

3. Availability of Materials
a. Data

- Data availability: Open
- Data completeness: Complete, all data necessary to reproduce main results are available.
- Access Method: Repository
- Repository: https://urldefense.proofpoint.com/v2/url?u=https-3A__doi.org_10.6084_m9.figshare.19700056.v2&d=DwIBaQ&c=shNJtf5dKgNcPZ6Yh64b-ALLUrcfR-4CCQkZVKC8w3o&r=88-dBITsh8vXfnQjNN0pRGpahxI_Sccu4B-wNY_gsU4&m=DrEuIoE1-sQIFwfRY3Hj36DKsd14DiNAYrTmYK2jpxnYAoVkZp93za8h4GNzjdos&s=xU6O1S4Ycd0v8mGPVJnvlRUTwUuBiGKzIbKum5VCwG[illegible]
-Data quality: Structured

b. Code
- Code availability: Open
- Programming Language(s): R
- Repository link: https://urldefense.proofpoint.com/v2/url?u=https-3A__github.com_Huang-2Dlab_BEACON&d=DwIBaQ&c=shNJtf5dKgNcPZ6Yh64b-ALLUrcfR-4CCQkZVKC8w3o&r=88-dBITsh8vXfnQjNN0pRGpahxI_Scc[illegible]wNY_gsU4&m=DrEuIoE1-sQIFwfRY3Hj36DKsd14DiNAYrTmYK2jpxnYAoVkZp93za8h4GNzjdos&s=szQ7p-kzh5bqrCvYxvHTeGSjRp3TzXonOd72MTpuSCM&e=
- License: MIT license
- Repository status: Public
- Documentation: Readme file

----

4. Computational environment of reproduction analysis

- Operating system for reproduction: Macbook Pro, M4 Max, 36Go, MacOS 15.6.1
- Programming Language(s): R
- Code implementation approach: Using shared code
- Version environment for reproduction: R version 4.5.1/RStudio 2025.05.1

----

5. Results

5.1 Original study results
- Results 1: Supplementary table S2

5.2 Steps for reproduction

-> Set up the environment to run PanLineageMCMC.R
--> Cloned the Github repository named "BEACON-main"
--> Installed JAGS as described in the README
--> Downloaded all data files in the figshare repository (https://urldefense.proofpoint.com/v2/url?u=https-3A__figshare.com_articles_dataset_DepMap-5F22Q2-5FPublic_19700056_2-3Ffile-3D35020903&d=DwIBaQ&c=shNJtf5dKgNcPZ6Yh64b-ALLUrcfR-4CCQkZVKC8w3o&r=88-dBITsh8vXfnQjNN0pRGpahxI_Sccu4[illegible]wNY_gsU4&m=DrEuIoE1-sQIFwfRY3Hj36DKsd14DiNAYrTmYK2jpxnYAoVkZp93za8h4GNzjdos&s=lby7NG0bYyh-K-nJquVredqpVUHBp-fG0nunt1aWoTE&e=), unzip and rename the folder to "DepMap_data". Placed this folder inside "BEACON[illegible]main".
--> Downloaded the data file mmc2 using the link provided in the code PanLineageMCMC.R. Note that the link provided (https://urldefense.proofpoint.com/v2/url?u=https-3A__www.cell.com_cms_10.1016_j.cell.2019.12.023_attachment_3709d[illegible]2D3a01-2D4e1d-2Dab4c-2D82597295c5d2_mmc2.xlsx&d=DwIBaQ&c=shNJtf5dKgNcPZ6Yh64b-ALLUrcfR-4CCQkZVKC8w3o&[illegible]dBITsh8vXfnQjNN0pRGpahxI_Sccu4B-wNY_gsU4&m=DrEuIoE1-sQIFwfRY3Hj36DKsd14DiNAYrTmYK2jpxnYAoVkZp93za8h4GNzjdos&s=bmYEY0wfysrZOL2v9k4RxVj2jIR6RKEvmjcXYSKP5iw&[illegible] does not work. Instead I used this link: https://urldefense.proofpoint.com/v2/url?u=https-3A__www.cell.com_cms_10.1016_j.cell.2019.12.023_attachment_3709dedc-2D3a01-2D4e1d-2Dab4c-2D82597295c5d2&d=DwIBaQ&c=shNJtf5dKgNcPZ6Yh64b-ALLUrcfR-4CCQkZVKC8w3o&r=88-dBITsh8vXfnQjNN0pRGpahxI_Sccu4B-wNY_gsU4&m=DrEuIoE1-sQIFwfRY3Hj36DKsd14DiNAYrTmYK2jpxnYAoVkZp93za8h4GNzjdos&s=LEFqk_XGdrZiXA2cIYwmGDEoRHzCHW0sTBdqrygCAg[illegible]). This was modified in PanLineageMCMC_modifiedLC.R. Placed the file in the "BEACON-main" folder.

-> Run the code PanLineageMCMC.R
- Issue 1: File import paths and incorrect file names
-- Resolved: The authors changed file paths to relative paths, but these paths are not correct because the folder "Huang_lab_
does not exist in my environment. Please clarify how to organize the environment or use real relative paths.
For example, instead of:

```
------------------ Start of script ------------------
depmap_info_path = file.path('..','..','..','..','Huang_lab_data','DepMap_data','sample_info_22Q2.csv')
------------------- End of script -------------------
```

Use:

```
------------------ Start of script ------------------
sam.dep = read.csv(file.path(getwd(), "DepMap_data", "sample_info.csv"))
------------------- End of script -------------------
```

Also, the downloaded DepMap_data files do not contain the suffix "_22Q2.csv". I removed this suffix in the paths in PanLineageMCMC_modifiedLC.R (lines 67-70).

- Issue 2: Files need to be gzip compressed
-- Resolved: Line 68 and 70, files need to be gzip compressed. Please specifiy it in the readme file or at the beginning of the
I compressed the file using the terminal.

```
------------------ Start of script ------------------
cd ~/local path
gzip CCLE_expression.csv
gzip CRISPR_gene_effect.csv
------------------- End of script -------------------
```

- Issue 3: Longer run time than expected.
-- Resolved: Authors mention an expected runtime of 1 or 2 hours for mRNA (8 vCPU / 16 GB RAM; n.iter=500 However, this runtime seems to apply per mRNA gene, not for the whole analysis. Reproducing the 12% subset took about 10 hours. Runni the full dataset is estimated at around 100 hours. Please clarify this in the documentation.
Then to be able to run the analysis we only rerun the code on the 244 significant genes from supplementary table S2 using th code:

```
------------------ Start of script ------------------
##### Filter the 244 significant genes listed in Supplementary Table S2 #####

# genes.query = intersect(gen.dat, gen.dep); i = 0; L = length(genes.query) #**

tableS2 = read.xlsx(file.path(getwd(), "TableS2_R1.xlsx"), sheet = 1, startRow = 2)
genes.query = intersect(gen.dat, gen.dep)
genes.query = genes.query[genes.query %in% tableS2$Gene]
i = 0; L = length(genes.query) #**
------------------- End of script -------------------
```

5.3 Statistical comparison Original vs Reproduced results

- Results: Table.mRNA.dependency.Bayesian.pancancer file attached
- Comments: The Bayesian PanCancer analysis was rerun only on the 244 significant genes from Supplementary Table S2, no full 17,285 genes, due to limited computational resources.
- Errors detected: In the supplementary table S2, the column mean is still here and not change in Rho. Based on this observa and considering that the values in the original Table S2 and the Table S2 in the revised manuscript appear to be identical, it i unclear whether the analysis was actually re-run using the fixed random seed to ensure reproducibility.
- Statistical Consistency: Among these 244 genes, the reproduced analysis confirmed their statistical significance. Numerical values (rho, standard deviation, Z value, p-value, and adjusted p-value) differed slightly, often by less than 10% in absolute. The percentage differences for p-values and adjusted p-values appear very high because the p-values themselves are extrem small. This is expected. These small discrepancies are reasonable given the nature of Bayesian inference.

Note that I ran the analyses twice on my laptop with the set.seed() at the beginning of the code. I did not get the same resul each time. From this, I understand that since JAGS uses its own random number generator, it is necessary to set the seed ins JAGS as well when using the jags.model() function. My comment is based on the documentation here, section Random numb generators:
https://urldefense.proofpoint.com/v2/url?u=https-3A__www.rdocumentation.org_packages_rjags_versions_4-2D17_topics_jags.model&d=DwIBaQ&c=shNJtf5dKgNcPZ6Yh64b-ALLUrcfR-4CCQkZVKC8w3o&r=88-dBITsh8vXfnQjNN0pRGpahxI_Sccu4B-wNY_gsU4&m=DrEuIoE1-sQIFwfRY3Hj36DKsd14DiNAYrTmYK2jpxnYAoVkZp93za8h4GNzjdos&s=j0jNObZlrpzbCB8ibMDIVjp8upRm9z8902kXncTcX7Q&e

----

6. Conclusion
- Summary of the second computational reproducibility review

The results of the Supplementary table were partially reproduced. We confirmed the statistical significance of the 244 genes reported in Supplementary Table S2 using the Bayesian PanCancer model in the provided code. We obtain slight differences i numerical results obtained. This is expected because Bayesian methods involve random sampling.

- Follow-up on previous recommendations:

In the first round of review, we noted several recommendations about:
-- Code annotation. The authors improved code comments, but the mmc2 download link is incorrect as noted above. Please update it and specify all download links clearly at the start of the code to facilitate reproducibility.

Authors: We apologize for the oversight regarding the proteomic data download link. We have now corrected this issue by updating the download link in both LineageMCMC.R and PanLineageMCMC.R scripts to the functional URL and consolidating al download links at the beginning of the script.

The authors clarified that the rho corresponds to the mean of the posterior distribution. This was modified in the code in two places (lines 325 and 357), only one change is sufficient.

Authors: We appreciate the reviewer's attention to this detail. We have removed the redundant variable name change on line retaining only the modification on line 325.

The supplementary table S2 was not updated with the new variable name.

Authors: We have rerun all analyses with the updated scripts and generated a new Supplementary Table S2 with correct vari names.

-- Set a random seed. The authors added a random seed at the beginning of the code, but it is unclear whether the analysis rerun using this seed. The values in Table S2 of the Revision 1 manuscript are identical to the original. Please to allow full reproducibility, add a seed to the jags.model() function as described in the R documentation. Please, re-run the analysis and update the file accordingly.

Authors: To ensure full reproducibility as recommended, we have rerun all analyses with random seeds specified both at the beginning of the R script (set.seed()) and within the JAGS model initialization using .RNG.name and .RNG.seed parameters as described in the rjags documentation. We have accordingly updated all supplementary tables and manuscript text to reflect t results generated with these fixed seeds.

-- Specify R and package versions. No requirements file or version information was provided. This is essential for reproducibil over time. Differences in package versions or computing environments can also cause the differences observed in the results. The authors created this, in an automatic way running the code, but did not provide it with the manuscript or on GitHub. Plea include it.

Authors: We have addressed this critical issue by creating a requirements.txt file in our GitHub repository that explicitly lists version (4.2.0: 2022-04-22), all required packages with their specific versions (rjags: 4-16, coda_0.19-4.1, openxlsx_4.2.7.1 This information is now available both in the manuscript and on the GitHub page (https://github.com/Huang-

lab/BEACON/blob/main/requirements.txt) to ensure long-term reproducibility across different computing environments.

-- Use relative file paths. Although file paths were updated, they are still not true relative paths since "Huang_lab_data" is mi[illegible] in my environment. Use proper relative paths as updated in PanLineageMCMC_modifiedLC.R or provide clear instructions for environment setup and folder naming.

Authors: We have adopted proper relative file paths throughout the LineageMCMC.R and PanLineageMCMC.R scripts, eliminat[illegible] the specific "Huang_lab_data" directory reference. The updated scripts now use working-directory-relative paths (e.g., file.path(getwd(), "DepMap_data", "sample_info.csv")). Additionally, we have provided comprehensive folder structure documentation and setup instructions in the GitHub README, including guidance on data preprocessing steps (e.g., gzip compression of expression and dependency data).

-- Increase MCMC robustness. The parameters currently defined in the code, particularly the number of iterations, are the sa[illegible] in the first version of the code. In their response, the authors stated that they expect more stable and reproducible results wi[illegible] the proposed changes. It is necessary to re-run the analysis with the updated MCMC settings to ensure the results are robust[illegible] reproducible.

Authors: We re-ran the full analysis with increased iterations (n.adapt=200, n.update=200, n.iter=1000) and fixed JAGS see[illegible] (i.e., 1,2, and 3, for the three chains, respectively). Posterior correlation estimates (rho) from the original and updated panlin[illegible] mRNA runs were almost perfectly correlated (e.g. Pearson r ≈ 0.999), with median absolute differences below a small thresh[illegible] (3e-4) and maximum absolute difference of 5e-3. Standard deviations, time-series standard errors, z-scores, and adjusted p-[illegible] values showed similarly minor changes, and all genes highlighted in the manuscript remained significant at the same thresho[illegible] (FDR < 0.05). These results indicate that the updated MCMC settings yield stable and reproducible estimates rather than materially altering the scientific conclusions.

-- Inform users about computation time. The stated expected runtime of 1-2 hours for mRNA is misleading. Re-running the fu[illegible] analysis takes around 100 hours. Please clarify this to inform users.

Authors: We have corrected the computational time documentation to accurately reflect the actual runtime: calculating panlin[illegible] mRNA correlations for 12619 genes takes approximately 50.7 hours (14.4 seconds per gene) on an 8-core processor with 32 [illegible] memory (OS: x86_64-pc-linux-gnu, 64-bit), and it takes 9.3 hours per lineage (on average). This information is now clearly [illegible] in the README to prevent user expectations misalignment.

Editorial Manager®

Cookie Preference Center

We use cookies which are necessary to make our site work. We may also use additional cookies to analyze, improve and personalize our content and your digital experience. For more information, see our Cookie Policy.

You may choose not to allow some types of cookies. However, blocking some types may

impact your experience of our site and the services we are able to offer. See the different category headings below to find out more or change your settings.

Allow all

Manage Consent Preferences

Strictly Necessary Cookies

Always active

These cookies are necessary for the website to function and cannot be switched off in our systems. They are usually only set in response to actions made by you which amount to a request for services, such as setting your privacy preferences, logging in or filling in forms. You can set your browser to block or alert you about these cookies, but some parts of the site will not then work.

Functional Cookies

These cookies enable the website to provide enhanced functionality and personalisation. They may be set by us or by third party providers whose services we have added to our pages. If you do not allow these cookies then some or all of these services may not function properly.

Performance Cookies

These cookies allow us to count visits and traffic sources so we can measure and improve the performance of our site. They help us to know which pages are the most and least popular and see how visitors move around the site.

Targeting Cookies

These cookies may be set through our site by our advertising partners. They may be used by those companies to build a profile of your interests and show you relevant adverts on other sites. If you do not allow these cookies, you will experience less targeted advertising.

Cookie List

Clear

Apply Cancel

Consent Leg.Interest

Confirm my choices

Powered by onetrus
