# Supplementary material for: Expression-driven genetic dependency reveals targets for precision oncology
Source: Gigascience. 2026 Jan 29;15:giag011. doi: 10.1093/gigascience/giag011 (PMC12970598; doi:10.1093/gigascience/giag011)
Supplement: giag011_Reviewer_1_Report_original_submission — Reviewer 1 -- 6/13/2025 [file giag011_reviewer_1_report_original_submission.pdf]

Reviewer Report

Title: Expression-Driven Genetic Dependency Reveals Targets for Precision Oncology

Version: Original Submission **Date:** 6/13/2025

Reviewer name: Reviewer 1

Reviewer Comments to Author:

The authors present BEACON, a method for identifying associations between the expression of a gene and sensitivity to the CRISPR knockout of that gene across a panel of cancer cell lines. These 'oncogene like' dependencies represent potential therapeutic targets that might be exploited for the development of new precision medicines in cancer. The issue that BEACON aims to address is the limited sample size (cell line count) in some specific cancer lineages and experimental noise that might result in spurious correlations between expression and CRISPR sensitivity. The authors demonstrate, using a modelling approach, that BEACON is more reliable for estimating correlation than simple Pearson's correlation when there is high-noise in the measurements. The majority of the manuscript focuses on analyses of dependencies systematically identified using the BEACON approach and their enrichment in drug targets and biological pathways. There is some experimental testing of three potential expression driven dependencies presented. The rationale for the overall approach and analyses are clear.

Major comments

- Previous efforts have systematically associated gene/protein expression with CRISPR sensitivity across the same or related datasets (e.g. Pacini et al, Cancer Cell 2024 and Rohde et al, Molecular Systems Biology 2025 using CRISPR; McDonald et al, Cell 2017 and Tsherniak et al, Cell 2017 using RNAi) and so the primary contribution of this paper can be considered the development of the BEACON method. It is thus somewhat surprising that there is no real assessment of the improvements offered by BEACON when compared to simpler methods (Pearson correlation, Spearman correlation) or more more complex recent approaches (Rohde et al's BACON approach). The modelling approach suggests some improvements in specific circumstances (especially high noise) but it is not clear that this leads to improved dependency identification in the real data. Does BEACON identify known oncogene addictions better than these methods? Are the associations identified more reproducible (e.g. across alternative CRISPR screens or RNAi screens)?
- The experimental validation and the conclusions drawn from it are somewhat confusing. The authors assess three potential expression associated dependencies - two pan-cancer dependencies (GRHL2 and TP63) and one lineage specific dependency (PAX5 in myeloid cells). Only the lineage-specific dependency validated in the way that might be expected, with higher expression associated with increased dependency, leading the authors to conclude that lineage-specific dependencies may be more suitable targets than pan-cancer ones. Given the numbers analysed (3 genes) this suggestion is not well supported. Moreover the perturbation was performed using distinct approaches - CRISPR for PAX5 and shRNA for the other two genes - and only the knockdown of PAX5 was validated by Western blot. It is very hard to know what phenotypes might be a false positive from off-target shRNA effects or false-negatives from variable shRNA knockdown of the target. The results in S5C suggest that the two shRNAs

for each gene cause somewhat discordant phenotypes, suggesting there may be some issues with knockdown efficiency. This could potentially be addressed by adding additional shRNAs for GRHL2 / TP63 or testing them using CRISPR perturbation as was done for PAX5. Validation of the knockdown of the intended target could also shed some light here. The manuscript also mentions experiments in an additional cell line (HCC15) but I cannot see these results presented in the main figures or supplement. It would be useful if all results for these two genes were presented in a single figure, with high and low expressing cell lines clearly marked,

Minor:

- Previous work has established that in some cases lower expression of a gene can make cells more vulnerable to its perturbation (CYCLOPS genes, Nijhawan et al, Cell 2012). While these are not the focus of this manuscript, it would be useful for the authors to comment on the utility of BEACON for their identification.
- p14 "Moreover, GED/PED targets were depleted of genes that were Essential In Culture" - it's not clear what this means or where the data comes from. By definition the gene set analysed are at least somewhat essential in culture

Methods

Are the methods appropriate to the aims of the study, are they well described, and are necessary controls included? Choose an item.

Conclusions

Are the conclusions adequately supported by the data shown? Choose an item.

Reporting Standards

Does the manuscript adhere to the journal's guidelines on minimum standards of reporting? Choose an item.

Choose an item.

Statistics

Are you able to assess all statistics in the manuscript, including the appropriateness of statistical tests used? Choose an item.

Quality of Written English

Please indicate the quality of language in the manuscript: Choose an item.

Declaration of Competing Interests

Please complete a declaration of competing interests, considering the following questions:

- Have you in the past five years received reimbursements, fees, funding, or salary from an organisation that may in any way gain or lose financially from the publication of this manuscript, either now or in the future?
- Do you hold any stocks or shares in an organisation that may in any way gain or lose financially from the publication of this manuscript, either now or in the future?
- Do you hold or are you currently applying for any patents relating to the content of the manuscript?
- Have you received reimbursements, fees, funding, or salary from an organization that holds or has applied for patents relating to the content of the manuscript?
- Do you have any other financial competing interests?
- Do you have any non-financial competing interests in relation to this paper?

If you can answer no to all of the above, write 'I declare that I have no competing interests' below. If your reply is yes to any, please give details below.

I declare that I have no competing interests.

I agree to the open peer review policy of the journal. I understand that my name will be included on my report to the authors and, if the manuscript is accepted for publication, my named report including any attachments I upload will be posted on the website along with the authors' responses. I agree for my report to be made available under an Open Access Creative Commons CC-BY license (http://creativecommons.org/licenses/by/4.0/). I understand that any comments which I do not wish to be included in my named report can be included as confidential comments to the editors, which will not be published.

Choose an item.

To further support our reviewers, we have joined with Publons, where you can gain additional credit to further highlight your hard work (see: https://publons.com/journal/530/gigascience). On publication of this paper, your review will be automatically added to Publons, you can then choose whether or not to claim your Publons credit. I understand this statement.

Yes Choose an item.
