# Supplementary material for: Expression-driven genetic dependency reveals targets for precision oncology
Source: Gigascience. 2026 Jan 29;15:giag011. doi: 10.1093/gigascience/giag011 (PMC12970598; doi:10.1093/gigascience/giag011)
Supplement: giag011_Reviewer_2_Report_original_submission — Reviewer 2 -- 6/30/2025 [file giag011_reviewer_2_report_original_submission.pdf]

Reviewer Report

Title: Expression-Driven Genetic Dependency Reveals Targets for Precision Oncology

Version: Original Submission **Date:** 6/30/2025

Reviewer name: Reviewer 2

Reviewer Comments to Author:

*The authors introduce BEACON, a Bayesian correlation approach designed to identify expression-driven dependency in cancer. Their hypothesis suggests that cancer cells with elevated expression of specific genes demonstrate increased vulnerability to the knockout of those same genes, thereby unveiling a promising new category of targets in precision oncologyâ€"particularly valuable for targeting cancer cells lacking druggable mutations.
*BEACON models expression levels and dependency scores as bivariate Gaussians and employs Markov Chain Monte Carlo (MCMC) sampling to estimate the correlation coefficient between them. They then compute p-values followed by rigorous multiple testing correction (BH based FDR correction).
*A notable strength of their approach lies in the integration of mass spectrometry proteomics data alongside transcriptomic and perturbation screening data, enhancing the robustness of their findings.
*Their work highlights some key insights:
- Gene expression-driven dependency (GED) candidates identified across lineages demonstrate enrichment for "DNA-binding transcription activator activity" and "DNA-binding transcription activator activity, RNA polymerase II-specific" pathways.
- The analysis successfully identifies compelling candidates with robust signals in both GED and PED (FERMT2, GRHL2, KLF5, CDK6, and CCND1), which are well-supported by existing drug evidence or established literature
- Clustering analyses reveal that cancer cells from pancreas and biliary tract tissues, as well as kidney and urinary tract tissue lineages, exhibit remarkably similar expression-driven dependency profiles. Additionally, lineage-specific genes such as transcription factors, cluster together in a manner consistent with existing literature
- Through Fisher's exact test, the authors demonstrate significant enrichments of druggable gene lists from DrugBank with expression-driven dependency patterns at both proteomic and transcriptomic levels
- Experimental validation shows that PAX5 is essential for PAX5-high B cell lymphoma cell growth, while TP63 and GRHL2 are essential for LSCC cell growth.

However, I have several principal concerns about the study that should be addressed to demonstrate the robust and superior performance of this proposed approach.

Major Comments:

1.Quantitative benchmarking: While the authors present a valuable contribution, the concept of correlating gene dependency scores to expression has been explored previously through approaches like Project DRIVE (E. Robert McDonald, III et al.) and APSIC (Montazeri et al.). BEACON demonstrates strong correlations across multiple lineages, representing broader scope compared to existing methods that

appear more lineage-restricted. However, establishing BEACON's comparative advantages requires more rigorous evaluation. Notably, Project DRIVEâ€”a foundational paper in this fieldâ€”already identified several BEACON candidates in their "Expression Correlation Analysis Identifies Oncogenes and Lineage-Specific Transcription Factors" section, while APSIC characterized many lineage-specific discoveries as tumor effector genes. BEACON's strength lies in integrating proteomic data with transcriptomic and perturbation screens, enabling identification of additional candidates like PAX5 for hematopoietic and lymphoid tissue. To demonstrate the method's impact, I recommend systematic quantitative benchmarking against existing approaches.
Importantly, BEACON utilizes richer/complementary datasets than previous studies. Disentangling contributions of data richness versus methodological innovation would provide valuable insights into whether enhanced performance stems from improved data availability or genuine method improvements.
Overall for benchmarking, the authors are strongly encouraged to utilize any comprehensive datasets that best demonstrate their method's competitive advantage and are not limited to the specific comparisons recommended above.
2.Correlation method comparisons: Figure S2 shows that BEACON exhibits higher MSE at extremes, and the claimed advantage over Pearson for small sample sizes is difficult to quantify from the current visualization. While the theoretical expectation that BEACON should outperform Pearson in small samples is reasonable, the practical significance remains unclear from these simulations. I recommend demonstrating BEACON's advantage using real data by creating a curated list of established GEDs/PEDs and comparing performance between the two methods. This is particularly important since several of BEACON's hits were previously reported by Project DRIVE using simple Pearson correlations. Alternatively, if BEACON's advantage is indeed significant, please elaborate on the simulation results to better justify this claim with clearer quantitative metrics.
3.Validation experiments: I'm seeking clarification on the validation experiments for TP63 and GRHL2. These candidates were not sensitive to predicted dependency and the authors say that "pan-lineage targets may represent universal vulnerability and their inhibition may lead to undesired off-target effects on other cells". Are the authors positioning them as weaker candidates to illustrate the superiority of lineage-specific predictions like PAX5? Additionally, why were different experimental approaches usedâ€”CRISPR for PAX5 versus shRNA for TP63 and GRHL2? For a method aimed at identifying druggable targets, would drug based experiments be more relevant than knockdown approaches to better demonstrate clinical applicability?
Minor comments
1. In Figure 4A, the caption refers to the plot as a heatmap, but the visualization appears to be a scatterplot. Please clarify whether the heatmap is missing or modify the caption appropriately. Additionally, I recommend using a different shade of green, as the current color choice makes some gene names difficult to read.
2. In Fig S5A, please add a legend for tumor and normal
3. For the TP63 and GRHL2 validation experiments, please include results for all four cell lines. The current manuscript is missing HCC15-shTP63, HCC15-shGRHL2, and HARA-shGRHL2 plots.
4. How many replicates were the experiments performed on? Is it N= 3 for all experiments?
5. Missing some text here - "BEACON offers the unique advantage of utilizing prior distributions that are

less susceptible to outliers, especially in multiple lineages where the number of cell lines."

Methods

Are the methods appropriate to the aims of the study, are they well described, and are necessary controls included? Choose an item.

Conclusions

Are the conclusions adequately supported by the data shown? Choose an item.

Reporting Standards

Does the manuscript adhere to the journal's guidelines on minimum standards of reporting? Choose an item.

Choose an item.

Statistics

Are you able to assess all statistics in the manuscript, including the appropriateness of statistical tests used? Choose an item.

Quality of Written English

Please indicate the quality of language in the manuscript: Choose an item.

Declaration of Competing Interests

Please complete a declaration of competing interests, considering the following questions:

- Have you in the past five years received reimbursements, fees, funding, or salary from an organisation that may in any way gain or lose financially from the publication of this manuscript, either now or in the future?
- Do you hold any stocks or shares in an organisation that may in any way gain or lose financially from the publication of this manuscript, either now or in the future?
- Do you hold or are you currently applying for any patents relating to the content of the manuscript?
- Have you received reimbursements, fees, funding, or salary from an organization that holds or has applied for patents relating to the content of the manuscript?
- Do you have any other financial competing interests?
- Do you have any non-financial competing interests in relation to this paper?

If you can answer no to all of the above, write 'I declare that I have no competing interests' below. If your reply is yes to any, please give details below.

I declare that I have no competing interests

I agree to the open peer review policy of the journal. I understand that my name will be included on my report to the authors and, if the manuscript is accepted for publication, my named report including any attachments I upload will be posted on the website along with the authors' responses. I agree for my report to be made available under an Open Access Creative Commons CC-BY license (http://creativecommons.org/licenses/by/4.0/). I understand that any comments which I do not wish to be included in my named report can be included as confidential comments to the editors, which will not be published.

Choose an item.

To further support our reviewers, we have joined with Publons, where you can gain additional credit to further highlight your hard work (see: https://publons.com/journal/530/gigascience). On publication of this paper, your review will be automatically added to Publons, you can then choose whether or not to claim your Publons credit. I understand this statement.

Yes Choose an item.
