# Supplementary material for: Expression-driven genetic dependency reveals targets for precision oncology
Source: Gigascience. 2026 Jan 29;15:giag011. doi: 10.1093/gigascience/giag011 (PMC12970598; doi:10.1093/gigascience/giag011)
Supplement: giag011_Reviewer_2_Report_Revision_1 — Reviewer 2 -- 10/25/2025 [file giag011_reviewer_2_report_revision_1.pdf]

**Reviewer Report**

**Title: Expression-Driven Genetic Dependency Reveals Targets for Precision Oncology**

**Version: Revision 1** **Date:** 10/25/2025

**Reviewer name: Reviewer** 2

**Reviewer Comments to Author:**

In their revised manuscript, the authors have addressed several concerns raised in my initial review, resulting in a notably strengthened contribution. The authors are appreciated for their efforts in conducting additional benchmarking analyses and expanding the simulation studies. However, the newly introduced figures reveal some issues that warrant attention before the manuscript can be recommended for publication.

- The simulation results seem to suggest that Spearman correlation outperforms BEACON at noise levels â‰¤ 0.5 and larger sample sizes, while BEACON exhibits performance comparable to Pearson, especially for stronger correlations. Could the authors provide an explanation for these observed patterns and discuss the conditions under which BEACON offers advantages over these methods in the methods section of the manuscript? The current statementâ€""...while Spearman better captured monotonic non-linear trends, though this advantage largely disappeared in small, noisy cohorts"â€"is limited and would benefit from an expansion to delineate the scenarios where BEACON's methodology provides meaningful benefits over Spearman correlations.
- The use of different experimental approaches for validationâ€"CRISPR-based methods for PAX5 versus shRNA for GRHL2 and TP63â€"is understandable given logistical and resource constraints, but represents a limitation that warrants acknowledgement. Given the well-documented variability between CRISPR and shRNA screening platforms in the literature, this inconsistency limits the comparability of experiments. I recommend that the authors add a brief statement in the Discussion section clarifying that the current experiments were designed to provide proof-of-concept validation of BEACON's predictive capabilities, while emphasizing that future work should prioritize large-scale, methodologically uniform validation studies to more rigorously assess the reliability and generalizability of BEACON's predictions.
- For PED, the AUPRC values for the DRIVE dataset appear notably low across all methods. Furthermore, it is surprising to see that Pearson correlation is low in performance, given that DRIVE targets are Pearson-based, and one would expect stronger concordance under these conditions. Is this because of the difference between DRIVE and DepMap datasets? Could the authors provide an explanation for these unexpectedly low AUPRC values?
- Please clarify the meaning of the XX/YY% notation to ensure unambiguous interpretation.

Comments on figures:

- The lineage legends in Figures 2C, 3C, and 5D can be reduced to show only the lineages actually depicted in each panel, as the current legends are distracting.
- The genes mentioned as "highlighted" in Figure 4 (SOX10, TP63, IRF4, and MYB) are not visually distinguished in the figure. Please add highlighting or revise the text.

- Figures 5B-C: Should 'Drug targets' and 'Other genes' be differentiated by color in the legend? This distinction is currently unclear.
- Please Italicize gene names in plots to separate genes from proteins.
- Figures in S4 and S6 appear to be missing axis labels. Additionally, Figure S6C appears to have a rendering issue in the title that should be corrected.

**Methods**

Are the methods appropriate to the aims of the study, are they well described, and are necessary controls included? Choose an item.

**Conclusions**

Are the conclusions adequately supported by the data shown? Choose an item.

**Reporting Standards**

Does the manuscript adhere to the journal's guidelines on [minimum standards of reporting?] Choose an item.

Choose an item.

**Statistics**

Are you able to assess all statistics in the manuscript, including the appropriateness of statistical tests used? Choose an item.

**Quality of Written English**

Please indicate the quality of language in the manuscript: Choose an item.

**Declaration of Competing Interests**

Please complete a declaration of competing interests, considering the following questions:

- Have you in the past five years received reimbursements, fees, funding, or salary from an organisation that may in any way gain or lose financially from the publication of this manuscript, either now or in the future?
- Do you hold any stocks or shares in an organisation that may in any way gain or lose financially from the publication of this manuscript, either now or in the future?
- Do you hold or are you currently applying for any patents relating to the content of the manuscript?
- Have you received reimbursements, fees, funding, or salary from an organization that holds or has applied for patents relating to the content of the manuscript?

- Do you have any other financial competing interests?
- Do you have any non-financial competing interests in relation to this paper?

If you can answer no to all of the above, write 'I declare that I have no competing interests' below. If your reply is yes to any, please give details below.

I declare that I have no competing interests.

I agree to the open peer review policy of the journal. I understand that my name will be included on my report to the authors and, if the manuscript is accepted for publication, my named report including any attachments I upload will be posted on the website along with the authors' responses. I agree for my report to be made available under an Open Access Creative Commons CC-BY license (http://creativecommons.org/licenses/by/4.0/). I understand that any comments which I do not wish to be included in my named report can be included as confidential comments to the editors, which will not be published.

Choose an item.

To further support our reviewers, we have joined with Publons, where you can gain additional credit to further highlight your hard work (see: https://publons.com/journal/530/gigascience). On publication of this paper, your review will be automatically added to Publons, you can then choose whether or not to claim your Publons credit. I understand this statement.

Yes Choose an item.
