# Supplementary material for: Expression-driven genetic dependency reveals targets for precision oncology
Source: Gigascience. 2026 Jan 29;15:giag011. doi: 10.1093/gigascience/giag011 (PMC12970598; doi:10.1093/gigascience/giag011)
Supplement: giag011_Reviewer_3_Report_original_submission — Reviewer 3 -- 7/7/2025 [file giag011_reviewer_3_report_original_submission.pdf]

Reviewer Report

Title: Expression-Driven Genetic Dependency Reveals Targets for Precision Oncology

Version: Original Submission **Date:** 7/7/2025

Reviewer name: Reviewer 3

Reviewer Comments to Author:

The authors develop a method for correlating gene and protein expression with cellular dependencies using the resources of DepMap. The innovation appears to be a Bayesian approach to the correlation analysis. They use this approach to identify potential therapeutic targets and evaluate some top candidates using in vitro experiments. The paper is fairly straightforward to follow.

Major comments:

1. Benchmarking - given the non-linear relationships shown in Fig 2, is a comparison with the Pearson method the most appropriate? Would a Spearman's be better?
2. The analysis identifies dependencies that are proposed as therapeutic targets, however while the proteins can be druggable, what about normal tissue effects? Some of these are likely lineage-defining proteins that could be highly expressed in normal tissues. Is is notable that in Fig 5B, C that the existing drug targets have a lower association strength than other GEDs identified. Does this suggest that the strongest correlations might be lineage-crucial genes that are too important for normal tissue function to make good drug targets? This needs further consideration in the discussion. Are there any pathways differences between these groups (known drug targets vs others)? For example you might expect more tissue lineage Tfs in the "other" category, while the approved drug targets perhaps more cell surface receptors.
3. The cell assays performed should effectively be replicating the results of the dependencies on which BEACON is based (DepMap), so why do you get different results? Is it because of the different methods used ie shRNA (not seeing the correlation between expression and dependency) vs CRISPR (replicating the correlation)? If you look at older DepMap scores when they used knockdown rather than CRISPR can you replicate your results?
4. Although mycoplasma testing was done, were the cell lines re-authenticated by STR profiling at any point?
5. QPCR is mentioned n the methods but not provided in the results that I can find. Did this validate gene knockdown by shRNA? Any correlation between % KD and proliferation/colony forming effect?
6. In the discussion it should be acknowledged that cancer subtypes exist within lineages that are molecularly and clinically distinct and so the method might be missing targets specific for these eg ER+ and ER- breast cancer.

Minor comments:

1. Results para 1 "especially in multiple lineages where the number of cell lines." Missing something in this sentence?
2. Needs some grammar review
3, Please italicise all gene names (when referring to gene, not protein) eg CCNE1 amplification etc

4. Fig S5A - legend or axis labels for N and T needed.
5. Fig S5C, D - these are proliferation not colony forming assays as stated in the text.
6. Please include number of replicates and type of error bars in figure legends for cell assays

Methods

Are the methods appropriate to the aims of the study, are they well described, and are necessary controls included? Choose an item.

Conclusions

Are the conclusions adequately supported by the data shown? Choose an item.

Reporting Standards

Does the manuscript adhere to the journal's guidelines on minimum standards of reporting? Choose an item.

Choose an item.

Statistics

Are you able to assess all statistics in the manuscript, including the appropriateness of statistical tests used? Choose an item.

Quality of Written English

Please indicate the quality of language in the manuscript: Choose an item.

Declaration of Competing Interests

Please complete a declaration of competing interests, considering the following questions:

- Have you in the past five years received reimbursements, fees, funding, or salary from an organisation that may in any way gain or lose financially from the publication of this manuscript, either now or in the future?
- Do you hold any stocks or shares in an organisation that may in any way gain or lose financially from the publication of this manuscript, either now or in the future?
- Do you hold or are you currently applying for any patents relating to the content of the manuscript?
- Have you received reimbursements, fees, funding, or salary from an organization that holds or has applied for patents relating to the content of the manuscript?
- Do you have any other financial competing interests?
- Do you have any non-financial competing interests in relation to this paper?

If you can answer no to all of the above, write 'I declare that I have no competing interests' below. If your reply is yes to any, please give details below.

I declare that I have no competing interests

I agree to the open peer review policy of the journal. I understand that my name will be included on my report to the authors and, if the manuscript is accepted for publication, my named report including any attachments I upload will be posted on the website along with the authors' responses. I agree for my report to be made available under an Open Access Creative Commons CC-BY license (http://creativecommons.org/licenses/by/4.0/). I understand that any comments which I do not wish to be included in my named report can be included as confidential comments to the editors, which will not be published.

Choose an item.

To further support our reviewers, we have joined with Publons, where you can gain additional credit to further highlight your hard work (see: https://publons.com/journal/530/gigascience). On publication of this paper, your review will be automatically added to Publons, you can then choose whether or not to claim your Publons credit. I understand this statement.

Yes Choose an item.
