# Supplementary material for: Expression-driven genetic dependency reveals targets for precision oncology
Source: Gigascience. 2026 Jan 29;15:giag011. doi: 10.1093/gigascience/giag011 (PMC12970598; doi:10.1093/gigascience/giag011)
Supplement: giag011_Reviewer_4_Report_original_submission — Reviewer 4 -- 7/14/2025 [file giag011_reviewer_4_report_original_submission.pdf]

Reviewer Report

Title: Expression-Driven Genetic Dependency Reveals Targets for Precision Oncology

Version: Original Submission **Date:** 7/14/2025

Reviewer name: Reviewer 4

Reviewer Comments to Author:

Reproducibility report for: Expression-Driven Genetic Dependency Reveals Targets for Precision Oncology
Journal: Gigascience
ID number/DOI: GIGA-D-25-00147
Reviewer(s): Laura Caquelin, Department of Clinical Neuroscience, Karolinska Institutet, Sweden
----
1. Summary of the Study
The authors developed a Bayesian method called BEACON to integrate multi-omics data. The method was tested on cancer cell lines across 17 tissue types to identify expression- driven dependencies. The method recovered known drug targets and identified novel candidates. The study concludes this method provides a systematic approach to identify precision oncology targets.
----
2. Scope of reproducibility
According to our assessment the primary objective is: to identify expression-driven dependencies across cancer cell lines from multiple lineages enabling the discovery of genes whose expression levels correlate with cancer cell dependency scores.
- Outcome: Identification of genes with significant expression-driven dependencies across pan-lineage cancer cell lines.
- Analysis method outcome: "BEACON calculated the Bayesian correlation between the gene's expressions and CERES cancer dependency scores 25 across the pan-lineage cell lines. BEACON modeled expression levels and dependency scores as the bivariate Gaussians and used Markov Chain Monte Carlo (MCMC) sampling to estimate the correlation coefficient rho between them. Given the null hypothesis that the uncorrelated expression and dependency of a gene has the 0 rho coefficient, we statistically tested each gene's rho estimate obtained from the MCMC simulation as follows. Assume that the MCMC sampling is carried out for a null gene's expression and dependency, then we expect that the distribution of the rho estimate accumulated over the MCMC iterations will be centered at zero. Based on this rationale, we computed the z-score of i-th gene as the deviation of the MCMC estimate of rho from the expected (null) value (i.e., zero) in terms of the standard deviation observed in the simulated distribution, i.e., z(i) = rhoMCMC(i) / SDMCMC(i). Since the z-values, by nature, follow a normal distribution with zero-mean and unit-variance, then we computed the p- value for each gene's rho estimate as the probability of observing a value as extreme as the computed z-value for that gene. We multi-testing corrected the resulting p-values using the BH procedure for FDR." (page 19 -Methods section / mRNA expression-driven dependency (GED))

- Main result: "We first analyzed the pan-lineage GED by using mRNA levels and the corresponding dependency scores from 854 cell lines with available data across 17 lineages and identified 244 genes showing significant association (correlation coefficient, rho < -0.25, FDR < 0.05)" (page 7 - Results section / Cancer vulnerability targets showing gene expression-driven dependency (GED))
----
3. Availability of Materials
a. Data
- Data availability: Open
- Data completeness: Complete, all data necessary to reproduce main results are available.
- Access Method: Repository
- Repository: https://doi.org/10.6084/m9.figshare.19700056.v2
-Data quality: Structured
b. Code
- Code availability: Open
- Programming Language(s): R
- Repository link: https://github.com/Huang-lab/BEACON - License: MIT license
- Repository status: Public
- Documentation: Readme file
----
4. Computational environment of reproduction analysis
- Operating system for reproduction: MacOS 15.5
- Programming Language(s): R
- Code implementation approach: Using shared code
- Version environment for reproduction: R version 4.5.0/RStudio 2025.05.1
----
5. Results
5.1 Original study results
- Results 1: Supplementary table S2 5.2 Steps for reproduction
-> Run the code PanLineageMCMC.R
- Issue 1: File import paths and incorrect file name
-- Resolved: In the original code, there were fixed file paths that only worked on one specific computer. This caused problems when running the code on other computers. To fix this, I recommended to use relative paths, which are based on where the script is located. This way, the code can be run on any computer without needing to change the paths each time.
------------------ Start of script ------------------
```
sam.dep = read.csv(file.path(getwd(), "DepMap_data",
"sample_info.csv"))
```
------------------- End of script -------------------
- Issue 2: Missing function "intsect" at line 162
-- Resolved: The script called a function intsect that was not defined, leading to an error. Upon request, the authors provided the missing function and added it to the main script (PanLineageMCMC.R).
- Issue 3: Output directory not created.

-- Resolved: The script attempted to write output files to a directory that was not created beforehand. This caused errors during the loop execution when trying to save results. A directory check and automatic creation script was added. If the output folder does not exist, it is now created automatically before the loop runs.

------------------ Start of script ------------------

```
dir_path <-
paste0('../out/jags.nadapt',n.adapt,'.update',n.update,'.mcmc
',n.iter,'.simulation_SD_22Q2')
if (!dir.exists(dir_path)) {
dir.create(dir_path, recursive = TRUE)
}
```

------------------- End of script -------------------

5.3 Statistical comparison Original vs Reproduced results

- Results: Table.mRNA.dependency.Bayesian.pancancer file attached
- Comments: The Bayesian PanCancer analysis was re-run, but only on the 244 significant genes listed in Supplementary Table S2, not on the full set of 17 285 genes. This choice was made due to limited computational resources, as running the full model would have required an estimated 100 hours.
- Errors detected: -
- Statistical Consistency: Among the 244 significant genes originally reported, the reproduced analysis confirmed the statistical significance of these same genes. However, the exact numerical values (Mean, standard deviation, Z value, P-value and adjusted P-value) differed slightly. These discrepancies are expected due to the nature of Bayesian inference, the absence of a random seed, and the relatively low number of MCMC iterations used (n.iter = 500). These settings may not be sufficient to ensure full convergence or reproducibility of posterior estimates and should be interpreted with caution.

We were unable to compare the rho values because they were not available in the provided Supplementary table S2, nor extracted in the R code to be include in the resulting output files.

----

6. Conclusion

- Summary of the computational reproducibility review

The results of the Supplementary table S2 in the original study was partially reproduced. We were able to confirm the statistical significance of the 244 genes reported in Supplementary Table S2 using the Bayesian PanCancer model in the provided code. However, the numerical results were not always identical. This is expected because Bayesian methods involve random sampling, the original code did not set a fixed random seed, and the number of iterations used was relatively low. Furthermore, the rho values were not available for comparison, limiting a full reproducibility assessment.

Several technical issues were also fixed during the reproduction process, such as hardcoded file paths, a missing function, and the absence of output directories, which were resolved to allow the code to run correctly on a different system. Due to computational limitations, running the full model on all 17,285 genes was not performed.

- Recommendations for authors

While the original analysis code was successfully used to confirm the statistical significance of the 244 genes, we recommend several improvements to enhance reproducibility:

-- Code annotation: Adding more detailed comments within the scripts would help users understand the logic behind each step and the purpose of specific commands or operations.
-- Set a random seed: Include set.seed() in all scripts to improve reproducibility across different runs.
-- Specify R and package versions: Provide the R version and exact package versions needed to run the code, via a requirements file for example.
-- Use relative file paths: Ensure that all necessary folders and functions are created or included by default to avoid path issues.
-- Increase MCMC robustness: Use a higher number of iterations and appropriate parameter settings to ensure better convergence and stability of posterior estimates.
-- Inform users about computation time: Clearly indicate in the README or publication the expected runtime of the code, especially if it requires several hours or days to complete.

Methods

Are the methods appropriate to the aims of the study, are they well described, and are necessary controls included? Choose an item.

Conclusions

Are the conclusions adequately supported by the data shown? Choose an item.

Reporting Standards

Does the manuscript adhere to the journal's guidelines on minimum standards of reporting? Choose an item.

Choose an item.

Statistics

Are you able to assess all statistics in the manuscript, including the appropriateness of statistical tests used? Choose an item.

Quality of Written English

Please indicate the quality of language in the manuscript: Choose an item.

Declaration of Competing Interests

Please complete a declaration of competing interests, considering the following questions:

- Have you in the past five years received reimbursements, fees, funding, or salary from an organisation that may in any way gain or lose financially from the publication of this manuscript, either now or in the future?

- Do you hold any stocks or shares in an organisation that may in any way gain or lose financially from the publication of this manuscript, either now or in the future?
- Do you hold or are you currently applying for any patents relating to the content of the manuscript?
- Have you received reimbursements, fees, funding, or salary from an organization that holds or has applied for patents relating to the content of the manuscript?
- Do you have any other financial competing interests?
- Do you have any non-financial competing interests in relation to this paper?

If you can answer no to all of the above, write 'I declare that I have no competing interests' below. If your reply is yes to any, please give details below.

1. No 2. No 3. No 4. No 5. No 6. No

I agree to the open peer review policy of the journal. I understand that my name will be included on my report to the authors and, if the manuscript is accepted for publication, my named report including any attachments I upload will be posted on the website along with the authors' responses. I agree for my report to be made available under an Open Access Creative Commons CC-BY license (http://creativecommons.org/licenses/by/4.0/). I understand that any comments which I do not wish to be included in my named report can be included as confidential comments to the editors, which will not be published.

Choose an item.

To further support our reviewers, we have joined with Publons, where you can gain additional credit to further highlight your hard work (see: https://publons.com/journal/530/gigascience). On publication of this paper, your review will be automatically added to Publons, you can then choose whether or not to claim your Publons credit. I understand this statement.

Yes Choose an item.
