# Supplementary material for: Expression-driven genetic dependency reveals targets for precision oncology
Source: Gigascience. 2026 Jan 29;15:giag011. doi: 10.1093/gigascience/giag011 (PMC12970598; doi:10.1093/gigascience/giag011)
Supplement: giag011_Reviewer_4_Report_Revision_1 — Reviewer 4 -- 10/8/2025 [file giag011_reviewer_4_report_revision_1.pdf]

Reviewer Report

Title: Expression-Driven Genetic Dependency Reveals Targets for Precision Oncology

Version: Revision 1 **Date:** 10/8/2025

Reviewer name: Reviewer 4

Reviewer Comments to Author:

Reproducibility report for: Expression-Driven Genetic Dependency Reveals Targets for Precision Oncology
Journal: Gigascience
ID number/DOI: GIGA-D-25-00147
Reviewer(s): Laura Caquelin, Department of Clinical Neuroscience, Karolinska Institutet, Sweden
----
1. Context
This report corresponds to a second assessment of the computational reproducibility of the article GIGA-D-25-00147, following a revision by the authors after the first round of review.
The scope of the computational reproducibility review is to reproduce the results presented in Table S2 relative to the identification of genes with significant expression-driven dependencies across pan-lineage cancer cell lines. The identification used a developed method called BEACON, based on Bayesian statistics, to find how strongly each gene's expression is correlated to dependency scores across the pan-lineage cell lines, by modeling the data with Gaussian distributions, estimating correlations using MCMC sampling, and testing significance with z-scores and FDR correction.
----
2. Changes since the first review
The authors made some changes to the code according to the comments from the first computational reproducibility review. The text in the manuscript related to Table S2 was slightly different, but the numerical values and observations remained unchanged.
----
3. Availability of Materials
a. Data
- Data availability: Open
- Data completeness: Complete, all data necessary to reproduce main results are available.
- Access Method: Repository
- Repository: https://doi.org/10.6084/m9.figshare.19700056.v2
-Data quality: Structured
b. Code
- Code availability: Open
- Programming Language(s): R
- Repository link: https://github.com/Huang-lab/BEACON
- License: MIT license

- Repository status: Public
- Documentation: Readme file
----
4. Computational environment of reproduction analysis
- Operating system for reproduction: Macbook Pro, M4 Max, 36Go, MacOS 15.6.1
- Programming Language(s): R
- Code implementation approach: Using shared code
- Version environment for reproduction: R version 4.5.1/RStudio 2025.05.1
----
5. Results
5.1 Original study results
- Results 1: Supplementary table S2
5.2 Steps for reproduction
-> Set up the environment to run PanLineageMCMC.R
--> Cloned the Github repository named "BEACON-main"
--> Installed JAGS as described in the README
--> Downloaded all data files in the figshare repository (https://figshare.com/articles/dataset/DepMap_22Q2_Public/19700056/2?file=35020903), unzip and rename the folder to "DepMap_data". Placed this folder inside "BEACON-main".
--> Downloaded the data file mmc2 using the link provided in the code PanLineageMCMC.R. Note that the link provided (https://www.cell.com/cms/10.1016/j.cell.2019.12.023/attachment/3709dedc-3a01-4e1d-ab4c-82597295c5d2/mmc2.xlsx) does not work. Instead I used this link: https://www.cell.com/cms/10.1016/j.cell.2019.12.023/attachment/3709dedc-3a01-4e1d-ab4c-82597295c5d2). This was modified in PanLineageMCMC_modifiedLC.R. Placed the file in the "BEACON-main" folder.
-> Run the code PanLineageMCMC.R
- Issue 1: File import paths and incorrect file names
-- Resolved: The authors changed file paths to relative paths, but these paths are not correct because the folder "Huang_lab_data" does not exist in my environment. Please clarify how to organize the environment or use real relative paths.
For example, instead of:
------------------ Start of script ------------------
depmap_info_path = file.path('..','..','..','..','Huang_lab_data','DepMap_data','sample_info_22Q2.csv')
------------------- End of script -------------------
Use:
------------------ Start of script ------------------
sam.dep = read.csv(file.path(getwd(), "DepMap_data", "sample_info.csv"))
------------------- End of script -------------------
Also, the downloaded DepMap_data files do not contain the suffix "_22Q2.csv". I removed this suffix in the paths in PanLineageMCMC_modifiedLC.R (lines 67-70).
- Issue 2: Files need to be gzip compressed
-- Resolved: Line 68 and 70, files need to be gzip compressed. Please specifiy it in the readme file or at

the beginning of the code. I compressed the file using the terminal.
------------------ Start of script ------------------

```
cd ~/local path
gzip CCLE_expression.csv
gzip CRISPR_gene_effect.csv
```

------------------- End of script -------------------

- Issue 3: Longer run time than expected.

-- Resolved: Authors mention an expected runtime of 1 or 2 hours for mRNA (8 vCPU / 16 GB RAM; n.iter=500 However, this runtime seems to apply per mRNA gene, not for the whole analysis. Reproducing the 12% subset took about 10 hours. Running on the full dataset is estimated at around 100 hours. Please clarify this in the documentation.

Then to be able to run the analysis we only rerun the code on the 244 significant genes from supplementary table S2 using the code:

------------------ Start of script ------------------

```
##### Filter the 244 significant genes listed in Supplementary Table S2 #####
# genes.query = intersect(gen.dat, gen.dep); i = 0; L = length(genes.query) #**
tableS2 = read.xlsx(file.path(getwd(), "TableS2_R1.xlsx"), sheet = 1, startRow = 2)
genes.query = intersect(gen.dat, gen.dep)
genes.query = genes.query[genes.query %in% tableS2$Gene]
i = 0; L = length(genes.query) #**
```

------------------- End of script -------------------

5.3 Statistical comparison Original vs Reproduced results

- Results: Table.mRNA.dependency.Bayesian.pancancer file attached
- Comments: The Bayesian PanCancer analysis was rerun only on the 244 significant genes from Supplementary Table S2, not the full 17,285 genes, due to limited computational resources.
- Errors detected: In the supplementary table S2, the column mean is still here and not change in Rho. Based on this observation, and considering that the values in the original Table S2 and the Table S2 in the revised manuscript appear to be identical, it is unclear whether the analysis was actually re-run using the fixed random seed to ensure reproducibility.
- Statistical Consistency: Among these 244 genes, the reproduced analysis confirmed their statistical significance. Numerical values (rho, standard deviation, Z value, p-value, and adjusted p-value) differed slightly, often by less than 10% in absolute.

The percentage differences for p-values and adjusted p-values appear very high because the p-values themselves are extremely small. This is expected. These small discrepancies are reasonable given the nature of Bayesian inference.

Note that I ran the analyses twice on my laptop with the set.seed() at the beginning of the code. I did not get the same results each time. From this, I understand that since JAGS uses its own random number generator, it is necessary to set the seed inside JAGS as well when using the jags.model() function. My comment is based on the documentation here, section Random number generators: https://www.rdocumentation.org/packages/rjags/versions/4-17/topics/jags.model.

----

6. Conclusion

- Summary of the second computational reproducibility review
The results of the Supplementary table were partially reproduced. We confirmed the statistical significance of the 244 genes reported in Supplementary Table S2 using the Bayesian PanCancer model in the provided code. We obtain slight differences in the numerical results obtained. This is expected because Bayesian methods involve random sampling.
- Follow-up on previous recommendations:
In the first round of review, we noted several recommendations about:
-- Code annotation. The authors improved code comments, but the mmc2 download link is incorrect as noted above. Please update it and specify all download links clearly at the start of the code to facilitate reproducibility.
The authors clarified that the rho corresponds to the mean of the posterior distribution. This was modified in the code in two places (lines 325 and 357), only one change is sufficient. The supplementary table S2 was not updated with the new variable name.
-- Set a random seed. The authors added a random seed at the beginning of the code, but it is unclear whether the analysis was rerun using this seed. The values in Table S2 of the Revision 1 manuscript are identical to the original. Please to allow full reproducibility, add a seed to the jags.model() function as described in the R documentation. Please, re-run the analysis and update the file accordingly.
-- Specify R and package versions. No requirements file or version information was provided. This is essential for reproducibility over time. Differences in package versions or computing environments can also cause the differences observed in the results.
The authors created this, in an automatic way running the code, but did not provide it with the manuscript or on GitHub. Please include it.
-- Use relative file paths. Although file paths were updated, they are still not true relative paths since "Huang_lab_data" is missing in my environment. Use proper relative paths as updated in PanLineageMCMC_modifiedLC.R or provide clear instructions for environment setup and folder naming.
-- Increase MCMC robustness. The parameters currently defined in the code, particularly the number of iterations, are the same as in the first version of the code. In their response, the authors stated that they expect more stable and reproducible results with the proposed changes. It is necessary to re-run the analysis with the updated MCMC settings to ensure the results are robust and reproducible.
-- Inform users about computation time. The stated expected runtime of 1-2 hours for mRNA is misleading. Re-running the full analysis takes around 100 hours. Please clarify this to inform users.

Methods

Are the methods appropriate to the aims of the study, are they well described, and are necessary controls included? Choose an item.

Conclusions

Are the conclusions adequately supported by the data shown? Choose an item.

Reporting Standards

Does the manuscript adhere to the journal's guidelines on minimum standards of reporting? Choose an item.

Choose an item.

Statistics

Are you able to assess all statistics in the manuscript, including the appropriateness of statistical tests used? Choose an item.

Quality of Written English

Please indicate the quality of language in the manuscript: Choose an item.

Declaration of Competing Interests

Please complete a declaration of competing interests, considering the following questions:

- Have you in the past five years received reimbursements, fees, funding, or salary from an organisation that may in any way gain or lose financially from the publication of this manuscript, either now or in the future?
- Do you hold any stocks or shares in an organisation that may in any way gain or lose financially from the publication of this manuscript, either now or in the future?
- Do you hold or are you currently applying for any patents relating to the content of the manuscript?
- Have you received reimbursements, fees, funding, or salary from an organization that holds or has applied for patents relating to the content of the manuscript?
- Do you have any other financial competing interests?
- Do you have any non-financial competing interests in relation to this paper?

If you can answer no to all of the above, write 'I declare that I have no competing interests' below. If your reply is yes to any, please give details below.

1. No 2. No 3. No 4. No 5. No 6. No

I agree to the open peer review policy of the journal. I understand that my name will be included on my report to the authors and, if the manuscript is accepted for publication, my named report including any attachments I upload will be posted on the website along with the authors' responses. I agree for my report to be made available under an Open Access Creative Commons CC-BY license (http://creativecommons.org/licenses/by/4.0/). I understand that any comments which I do not wish to be included in my named report can be included as confidential comments to the editors, which will not be published.

Choose an item.

To further support our reviewers, we have joined with Publons, where you can gain additional credit to further highlight your hard work (see: https://publons.com/journal/530/gigascience). On publication of this paper, your review will be automatically added to Publons, you can then choose whether or not to claim your Publons credit. I understand this statement.

Yes Choose an item.
